# Bithiophene‐Cored, *mono‐*, *bis‐*, and *tris‐*(Trimethylammonium)‐Substituted, *bis‐*Triarylborane Chromophores: Effect of the Number and Position of Charges on Cell Imaging and DNA/RNA Sensing

**DOI:** 10.1002/chem.202102308

**Published:** 2021-08-31

**Authors:** Sarina M. Berger, Jessica Rühe, Johannes Schwarzmann, Alexandra Phillipps, Ann‐Katrin Richard, Matthias Ferger, Ivo Krummenacher, Lidija‐Marija Tumir, Željka Ban, Ivo Crnolatac, Dragomira Majhen, Ivan Barišić, Ivo Piantanida, Domenik Schleier, Stefanie Griesbeck, Alexandra Friedrich, Holger Braunschweig, Todd B. Marder

**Affiliations:** ^1^ Institut für Anorganische Chemie and Institute for Sustainable Chemistry & Catalysis with Boron Julius-Maximilians-Universität Würzburg Am Hubland 97074 Würzburg Germany; ^2^ Division of Organic Chemistry and Biochemistry Ruder Boskovic Institute Bijenicka c. 54 10000 Zagreb Croatia; ^3^ Department of Molecular Biology Laboratory for Cell Biology and Signaling Ruder Boskovic Institute Bijenicka c. 54 10000 Zagreb Croatia; ^4^ Molecular Diagnostics, Center for Health and Bioresources AIT Austrian Institute of Technology GmbH Giefinggasse 4 1210 Wien Austria

**Keywords:** boron, bioimaging, luminescence, nucleic acid, singlet oxygen

## Abstract

The synthesis, photophysical, and electrochemical properties of selectively *mono*‐, *bis*‐ and *tris*‐dimethylamino‐ and trimethylammonium‐substituted *bis*‐triarylborane bithiophene chromophores are presented along with the water solubility and singlet oxygen sensitizing efficiency of the cationic compounds **Cat^1+^**, **Cat^2+^**, **Cat(i)^2+^**, and **Cat^3+^**. Comparison with the *mono*‐triarylboranes reveals the large influence of the bridging unit on the properties of the *bis*‐triarylboranes, especially those of the cationic compounds. Based on these preliminary investigations, the interactions of **Cat^1+^**, **Cat^2+^**, **Cat(i)^2+^**, and **Cat^3+^** with DNA, RNA, and DNApore were investigated in buffered solutions. The same compounds were investigated for their ability to enter and localize within organelles of human lung carcinoma (A549) and normal lung (WI38) cells showing that not only the number of charges but also their distribution over the chromophore influences interactions and staining properties.

## Graphical abstract

**Four selectively** ***mono*** **‐,** ***bis*** **‐ and** ***tris*** **‐trimethylammonium‐substituted**, bithiophene‐bridged *bis*‐triarylboranes were synthesized and their potential biological applications were investigated including live cell imaging and photo‐induced cell death. It is demonstrated that their photophysical and electrochemical properties and their interactions with DNAs, RNA and cells, are strongly influenced by the number and distribution of charges within the compounds.

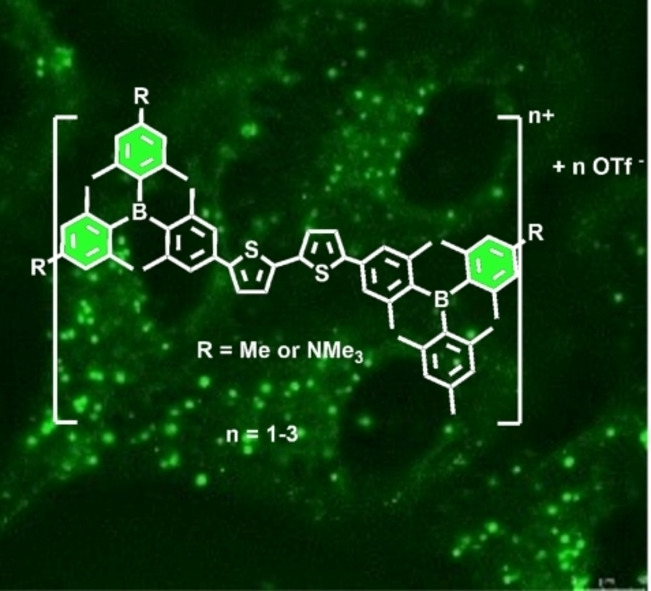

## Introduction

**Figure 1:**
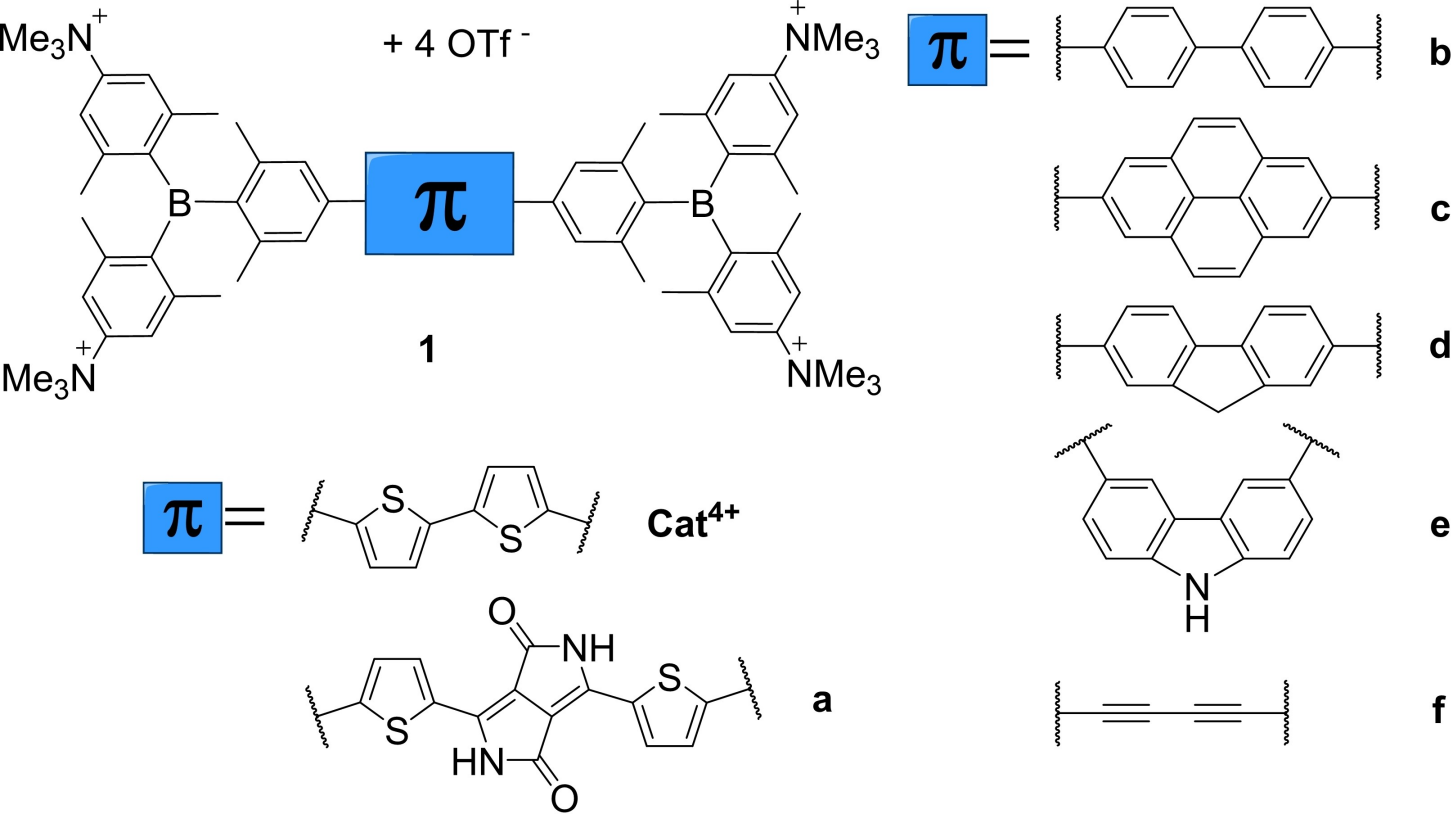
Overview of *tetra*‐cationic, *bis*‐triarylborane‐substituted chromophores reported by Marder and coworkers for biological applications.[^26^, ^29^, ^37^]

Three‐coordinate boron compounds have an empty p‐orbital at the boron center. Hence, such compounds are good π‐acceptors and display interesting photophysical and electrochemical properties, which lead to many different applications,[^1^, ^2^, ^3^, ^4^, ^5^, ^6^, ^7^, ^8^] e. g., optoelectronics,[^9^, ^10^, ^11^] sensors for anions or small molecules,[^12^, ^13^, ^14^, ^15^, ^16^, ^17^] and cell‐imaging agents and biomolecule sensors.[^15^, ^18^, ^19^, ^20^, ^21^, ^22^, ^23^, ^24^, ^25^, ^26^, ^27^, ^28^, ^29^, ^30^, ^31^, ^32^, ^33^] For the latter applications, which were summarized very recently by Berger and Marder,^[34]^ focusing on triarylboranes, water‐stable and soluble compounds are required. In 2009, Gabbaï and coworkers reported water‐soluble triarylboranes bearing at least two trimethylammonium groups at positions *para* to the boron center.^[35]^ Using this basic concept, we have shown that *tetra*‐cationic, *bis*‐triarylborane chromophores (Figure 1) are water‐soluble[^26^, ^29^, ^36^] and water‐stable if they contain at least 5 *ortho*‐methyl groups.^[27]^ Water‐stability can also be obtained upon introduction of other sterically demanding substituents, *e. g*., anthracene^[27]^ or perylene.^[18]^ It was also shown that such *bis*‐triarylborane chromophores can be used to stain organelles in HeLa cells[^29^, ^37^] or NIH 3T3, HEK 293T, and HEPG2–16 cells.^[26]^

**Figure 2:**
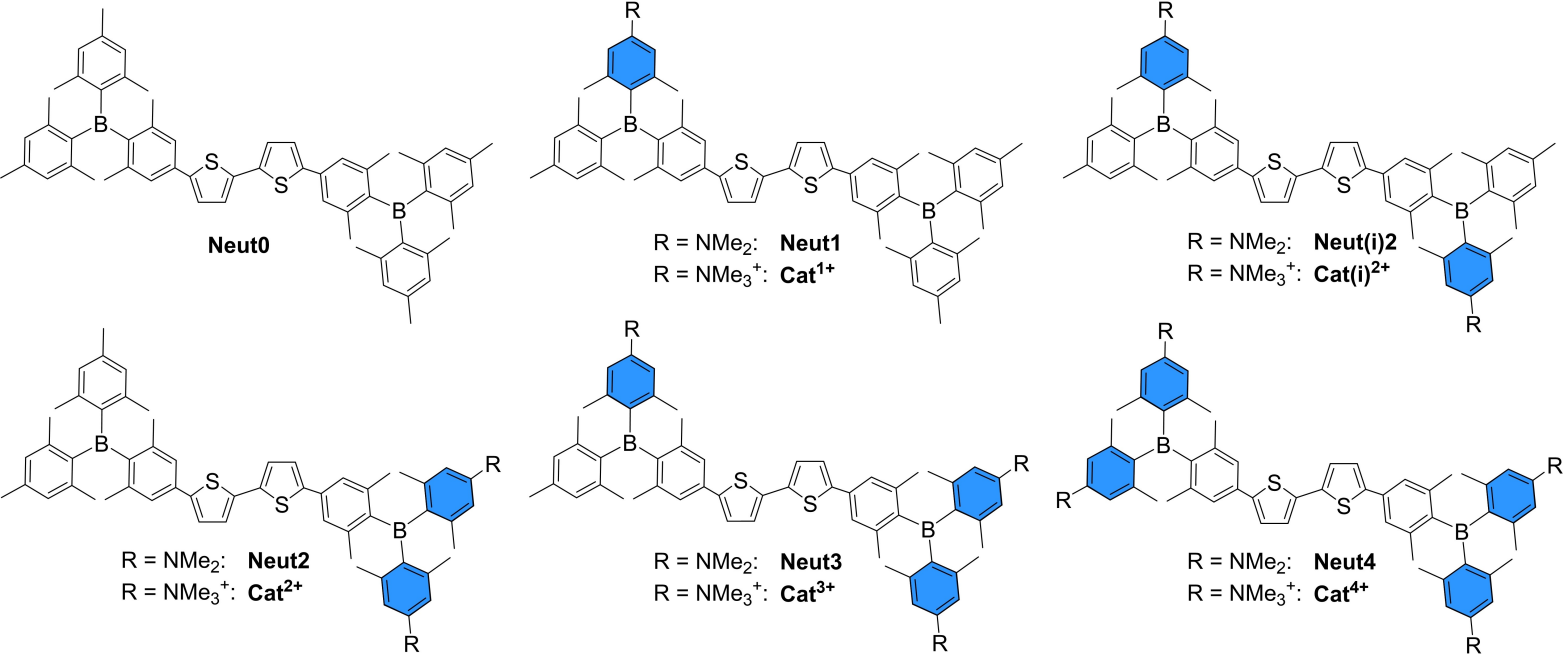
Molecular structures of **Neut0**‐**Neut4** and the selectively charged compounds **Cat^1+^**–**Cat^4+^** as their OTf^‐^ salts.

Combining these cellular experiments with investigations of the compound in buffered solutions in the presence of DNA, RNA and/or proteins, the bithiophene‐bridged model compound **Cat^4+^** (Figure 1) was shown to stain different cell organelles and to interact with proteins.^[38]^ In the present work, we demonstrate the influence of the number and position of positively charged trimethylammonium groups on basic properties such as water‐solubility, singlet oxygen sensitizing efficiency, and photophysical and electrochemical properties of a bithiophene‐bridged, *bis*‐triarylborane chromophore. Additionally, we demonstrate that biomolecule binding and staining selectivity for these compounds depends on the number and distribution of the cationic charges. Thus, the neutral compounds **Neut1**‐**Neut3** and the resulting selectively charged compounds **Cat^1+^**–**Cat^3+^** (Figure 2), which are analogues of the previously studied compounds **Neut4**, and **Cat^4+^**,^[26]^ were synthesized, fully characterized, and their properties were investigated. **Neut0** was synthesized as a reference for the investigation of photophysical and electrochemical properties.

The synthesis of **Neut1**, **Neut(i)2**, and **Neut3** was made possible via a synthetic route to unsymmetrically‐substituted triarylboranes which was previously not well developed,^[39]^ but has been reported very recently by our group.^[40]^

## Results and Discussion

### Synthesis

**Figure 3:**
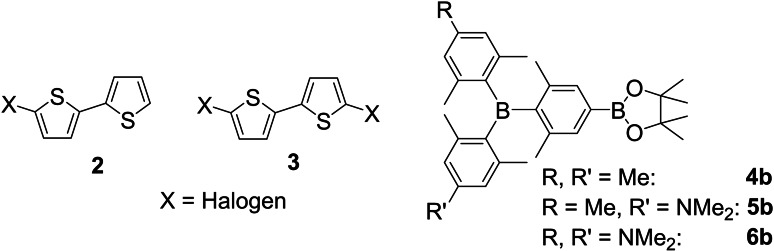
Starting materials for the synthesis of **Neut0**–**Neut3**.

The synthetic strategy is based on Suzuki‐Miyaura cross‐coupling reactions as previously reported for **Neut4** and **Cat^4+^**.^[26]^ Thus, *mono*‐ and *di*‐halogenated bithiophenes (**2**, **3**) as well as three different, borylated triarylboranes **4b**, **5b**, and **6b** were required (Figure 3).

Triarylboranes **6b** ^[26]^ and **5b** ^[40]^ and their *para*‐selective C−H borylated analogues were synthesized according to literature procedures. *Mono*‐[^41^, ^42^] and *di*‐brominated^[42]^ bithiophenes **2** and **3** were synthesized by routes similar to those in the literature.

**Scheme 1:**
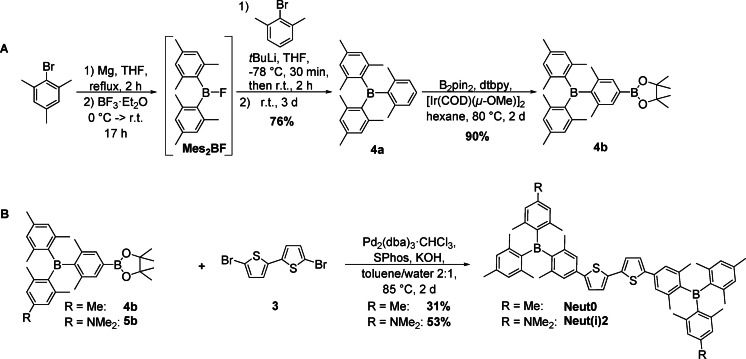
**A)** Synthetic route to **4a** and **4b. B)** Synthetic route to **Neut0** and **Neut(i)2**.

For the synthesis of **Neut0**, **4a** was synthesized from mesityl magnesium bromide (MesMgBr), BF_3_ ⋅ OEt_2_, and 2,6‐dimethylphenyl lithium using the general approach developed by Grisdale and co‐workers.^[43]^ Isolation of **Mes_2_BF** and subsequent reaction with 2,6‐dimethylphenyl lithium gave the triarylborane **4a** in 76 % yield over two steps. C−H borylation with [Ir(COD)(*μ*‐OMe)]_2_ as the precatalyst, 4,4′‐di‐*tert*‐butyl‐2,2′‐dipyridyl (dtbpy) as the ligand, and *bis*‐pinacolato diboron (B_2_pin_2_) gave **4b** in 90 % yield. The ^1^H NMR spectrum of **4b** matches that reported in the literature.^[44]^ Subsequently, Suzuki‐Miyaura cross‐coupling with 5,5’‐dibromo‐2,2’‐bithiophene **3** using Pd_2_(dba)_3_ ⋅ CHCl_3_ as the precatalyst, 2‐dicyclohexylphosphino‐2′,6′‐dimethoxybiphenyl (SPhos) as the ligand, and potassium hydroxide as the base gave **Neut0** in 31 % yield (Scheme 1B). Similarly, **Neut(i)2** was synthesized from **5b** and 5,5’‐dibromo‐2,2’‐bithiophene **3** in 53 % yield.

**Scheme 2:**
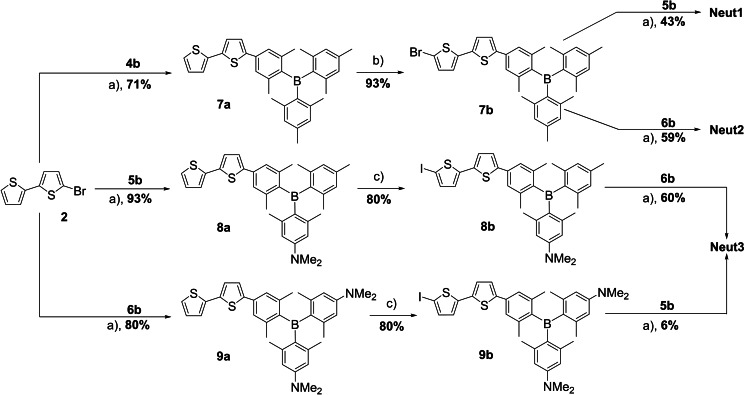
Synthetic routes to **Neut1**, **Neut2**, and **Neut3**. a) Pd_2_(dba)_3_ ⋅ CHCl_3_, SPhos, KOH, toluene/water 2 : 1, 85 °C, 2 d; b) NBS, DMF, 0 °C, 15 min then r.t., 2 h; c) *n*BuLi, THF, −78 °C, 1.5 h, then I_2_, −78 °C ‐> r.t., 18 h.

To obtain **Neut1**, **Neut2**, and **Neut3**, 5‐bromo‐2,2’‐bithiophene **2** was cross‐coupled with **4b**, **5b**, and **6b**, respectively, in Suzuki‐Miyaura reactions using the conditions described above to give **7a**, **8a**, and **9a** in 71 %, 93 %, and 80 % yields, respectively (Scheme 2). Prior to the second Suzuki‐Miyaura cross‐coupling, these intermediates were halogenated at the 5’‐position of the bithiophene. Compound **7a** was brominated at this position in 93 % yield using NBS in DMF. For the aminated compounds **8a** and **9a**, it was not possible to affect bromination with NBS at the desired position due to the more activated positions at the dimethylamino‐substituted arene. Therefore, the bithiophene moiety was lithiated with *n*BuLi and then treated with I_2_. This gave inseparable mixtures containing 80 % of the iodinated compounds **8b** and **9b** and their respective starting materials as determined by ^1^H NMR spectroscopy. As the starting material cannot undergo Suzuki‐Miyaura cross‐coupling, these mixtures were used directly, after minor purification, for the synthesis of **Neut3**. It was possible to isolate **Neut3** from both reaction sequences (**8b**+**6b** and **9b**+**5b**). However, the first reaction sequence gave higher yields in all steps, especially the last one (60 % vs. 6 % yield).

With all uncharged compounds **Neut1**–**3** in hand, their cationic counterparts were synthesized using methyl triflate in CH_2_Cl_2_ similar to previous reports[^26^, ^27^, ^29^, ^35^, ^40^] giving **Cat^1+^**, **Cat^2+^**, **Cat(i)^2+^**, and **Cat^3+^** in 88 %, 69 %, 49 %, and 57 % yields, respectively. The neutral triarylboranes **5a** and **6a** were methylated similarly giving **5c** and **6c** in 89 % and 93 % yields, respectively. With the neutral and cationic triarylboranes and *bis*‐triarylboranes in hand, their photophysical, and electrochemical properties were investigated as well as the singlet oxygen sensitizing efficiency and water‐solubility of **Cat^1+^**–**Cat^3+^** and their DNA/RNA binding affinities in buffered solutions. Additionally, the behavior of the most promising compounds **Cat(i)^2+^** and **Cat^3+^** was investigated in two different cell lines.

### Solubility in water

While **Cat^4+^** is soluble in pure water with a maximum solubility of 1.0 mM,^[26]^ none of the new, *mono*‐, *di*‐ or *tri*‐cationic *bis*‐triarylboranes are soluble in pure water at concentrations of 2.6–3.4 *μ*M (Figure S64, Table S2). However, concentrated solutions of **Cat^1+^**–**Cat^3+^** in acetonitrile can be diluted with water to less than 1 % without precipitation or aggregation. The concentrations of the cationic compounds in these diluted solutions were as high as 15 to 34 μM and are suitable for photophysical studies. Thus, aqueous solutions of the cationic *bis*‐triarylboranes were prepared from concentrated acetonitrile solutions which were diluted with water until the solution contained ≤1 % acetonitrile. For studies of interactions with DNA/RNA in buffered solutions and cells, stock solutions were prepared in DMSO (*c*=1×10^−2^ mol L^−1^) and diluted prior to use with the buffered solutions indicated.

### Photophysical data

**Figure 4:**
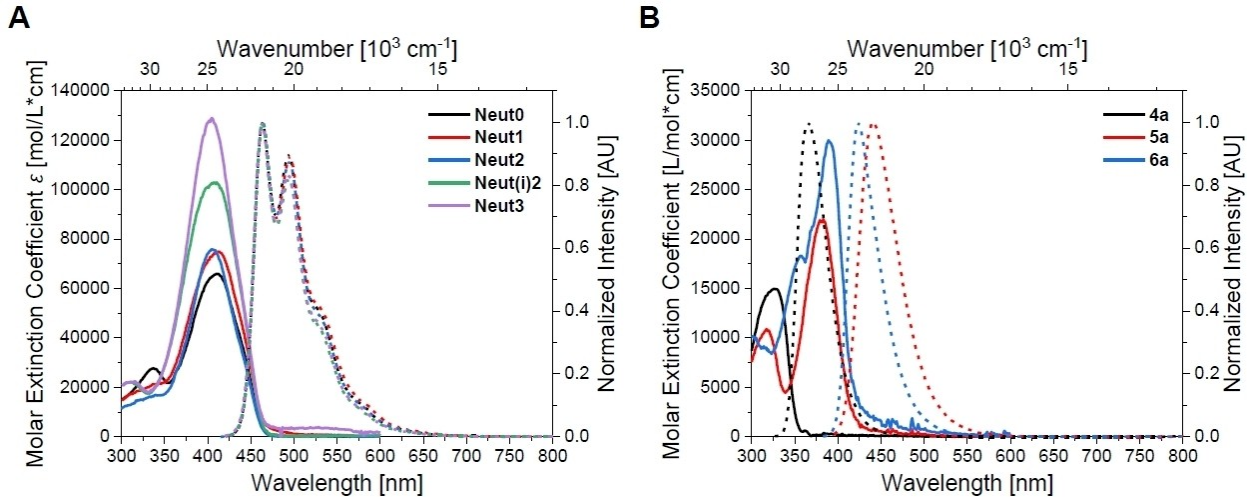
Absorption (solid lines) and emission (dotted lines; excitation at $\lambda_{max}^{abs}$ ) spectra of **A)** the neutral *bis*‐triarylboranes **Neut0**–**Neut3** and **B)** the neutral triarylboranes **4a**–**6a** in hexane.

**Figure 5:**
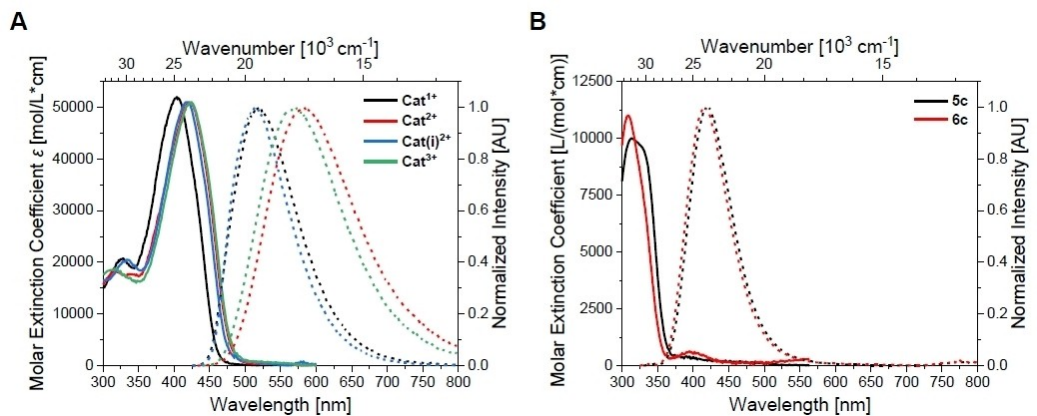
Absorption (solid lines) and emission (dotted lines; excitation at $\lambda_{max}^{abs}$ ) spectra of **A)** the cationic *bis*‐triarylboranes **Cat^1+^**–**Cat^3+^** and **B)** the cationic triarylboranes **5c** and **6c** in acetonitrile.

**Figure 6:**
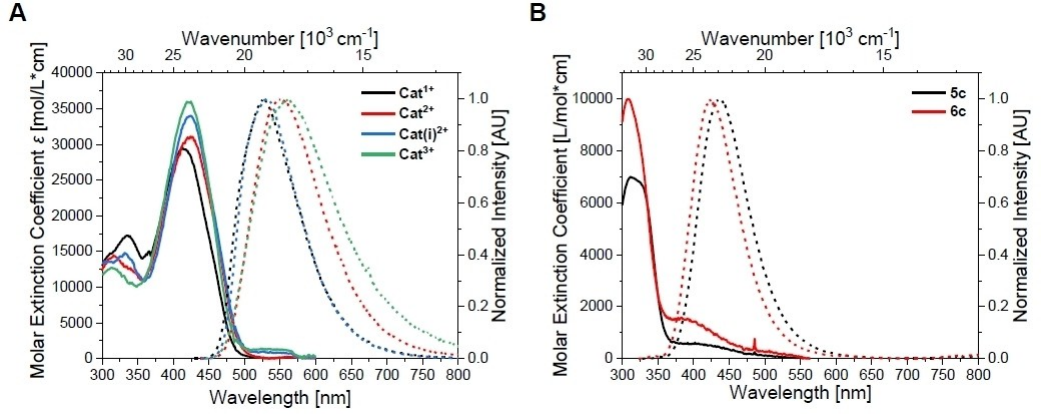
Absorption (solid lines) and emission (dotted lines; excitation at $\lambda_{max}^{abs}$ ) spectra of **A)** the cationic *bis*‐triarylboranes **Cat^1+^**–**Cat^3+^** and **B)** the cationic triarylboranes **5c** and **6c** in 1 % MeCN in water.

Due to large number of compounds which are discussed in the following sections of this paper, an overview of their molecular structures and numbering is given in Scheme S1 in the Supporting Information. The absorption spectra, extinction coefficients, emission spectra, fluorescence lifetimes, and quantum yields of triarylboranes **4a**‐**6a**, **5c**, and **6c** and of the cationic and neutral *bis*‐triarylboranes **Neut0**–**Neut3** and **Cat^1+^**–**Cat^3+^** were measured in solvents of different polarity (Table 1 and Figures S65–S78 in the Supporting Information). The photophysical properties of the triarylboranes and of the neutral *bis*‐triarylboranes are discussed briefly here (Figures 4,5,6) and a more detailed discussion is given in the Supporting Information.

**Table 1:** Selected photophysical properties of the new triarylboranes and *bis*‐triarylboranes.

	Solvent	$\lambda_{max}^{abs}$ [nm]	*ϵ* [L mol^−1^ cm^‐1^]	$\lambda_{max}^{fl}$ [nm]	apparent Stokes shift [cm^−1^]	*τ* [ns]	*Φ*_f_
**4a**	hexane	326	15000	367	3400	1.48	0.10
**5a**	hexane	383	22000	440	3400	5.17	0.34
**6a**	hexane	390	30000	424	2100	1.55	0.13
**5c**	MeCN	314	10000	420	8000	3.37	0.11
	1 % MeCN in water	313	7000	437	9100	7.49	0.19
**6c**	MeCN	309	11000	416	8300	4.40	0.10
	1 % MeCN in water	308	10000	425	8900	6.65	0.11
**Neut0**	hexane	410	66000	464	2800	0.52	0.31
**Neut1**	hexane	412	75000	464	2700	0.54	0.38
**Neut2**	hexane	405	76000	464	3100	0.54	0.32
**Neut(i)2**	hexane	408	103000	464	2900	0.54	0.39
**Neut3**	hexane	404	129000	463	3100	0.52	0.39
**Cat^1+^**	MeCN	414	52000	519	4900	0.82	0.34
	1 % MeCN in water	415	29500	527	5100	0.54 ^[a]^	0.07
**Cat^2+^**	MeCN	420	51000	584	6700	2.98	0.48
	1 % MeCN in water	424	31100	550	5400	0.76 ^[a]^	0.15
**Cat(i)^2+^**	MeCN	418	51000	515	4500	0.75	0.35
	1 % MeCN in water	424	34000	510	4000	0.63 ^[a]^	0.15
**Cat^3+^**	MeCN	424	51000	568	6000	2.41	0.46
	1 % MeCN in water	423	36000	558	5700	0.24 ^[a]^	0.08

[a] More than one lifetime was observed and only the dominant lifetime (>50 %) is listed here. More information is given in the Supporting Information.

The absorption spectra of the triarylboranes display no solvatochromism. The introduction of a strong electron‐donating functionality, in this case dimethylamino group(s), leads to bathochromically shifted absorption and emission maxima. Upon methylation, the positive solvatochromism of the emission is almost lost due to the loss of the charge‐transfer process which was supported by DFT and TD‐DFT calculations using the orbital overlap parameter Λ. This parameter is defined as $\Lambda =\;\frac{\sum_{i,a}c_{i,a}^{2}\left\langle {|\phi }_{a}|||\phi_{i}|\right\rangle }{\sum_{i,a}c_{i,a}^{2}}$ with $\phi_{a}$ and $\phi_{i}$ representing the occupied and unoccupied one‐electron wavefunctions, respectively, |<$\phi_{a}$ |||$\phi_{i}$ >| representing the norm of the one‐electron wavefunction centroid and *c*_*i,a*_ representing the weight of the one‐electron excitation. Λ takes values between 0 and 1, with 0 corresponding to no overlap and 1 to complete overlap.^[45]^ For all *mono*‐triarylboranes, these values are smaller than 0.6, thus suggesting localized orbitals and CT character of the transition.^[45]^ Hence, the photophysical properties of **4a**, **5c**, and **6c** are very similar, as these compounds are electronically similar due to the conversion of the electron‐donating amine to trimethylammonium groups upon methylation. The molar extinction coefficient increases almost linearly with the number of dimethylamino groups in the neutral triarylboranes. For **5c** and **6c**, these values are lower and independent of the number of trimethylammonium groups.

The properties of the neutral *bis*‐triarylboranes are very similar when dissolved in the same solvent, except for the molar extinction coefficients, which increase non‐linearly with the number of amino groups. The emission spectra display charge‐transfer character for **Neut1**‐**Neut3**, but not for **Neut0** as the latter bears no amino groups. DFT calculations show the HOMOs and LUMOs of the neutral *bis*‐triarylboranes to be mainly localized at the bithiophene bridge. Thus, transitions are of π–π* nature. This is supported by TD‐DFT calculations and Λ values resulting therefrom, which are between 0.66 and 0.72 thus reflecting delocalized orbitals over the bridge and locally excited transitions.^[45]^ In contrast, for the cationic *bis*‐triarylboranes, Λ values are between 0.30 and 0.53 reflecting charge‐transfer character of the transitions, and rather localized orbitals. Thus, charge transfer character of the transition of lowest energy is lost upon methylation, which has been shown previously for very similar compounds.^[26]^ The orbitals obtained from DFT calculations suggest that the HOMOs of the cationic *bis*‐triarylboranes are mainly localized at the bithiophene bridge as it is for the neutral compounds. However, the LUMOs of the cationic compounds are mainly localized at the most electron deficient boron center (**5c** for **Cat^1+^**; **6c** for **Cat^2+^**, **Cat^3+^**). Therefore, the transition for most of the cationic *bis*‐triarylboranes is not of π–π* nature but of π‐B_(p)_ nature. In the case of **Cat(i)^2+^** and **Cat^4+^**,^[26]^ the LUMO is delocalized from one boron center to the other over the bithiophene bridge. As a result, the transition is of π–π* nature. Due to the different origins of the main transitions of the cationic *bis‐*triarylboranes, the photophysical properties are not the same for these compounds as they depend to some extent on the number and distribution of the trimethylammonium groups. For example, in acetonitrile, the absorption maxima shift bathochromically with increasing number of trimethylammonium groups. The emission maxima shift bathochromically with increasing dipole moment of the cationic *bis*‐triarylboranes in the order **Cat^1+^** ≈**Cat(i)^2+^** ≪**Cat^3+^** <**Cat^2+^**. Note that the dipole moment of charged compounds is defined relative to its origin. Thus, it is not an observable quantity. However, the term dipole moment will be used herein to describe the distribution of the electron density over the molecules for convenience. The molar extinction coefficient does not change significantly with increasing number of trimethylammonium groups. Similar, but less pronounced trends are found in 1 % MeCN in water and dilutions of DMSO stock solutions with sodium cacodylate buffer solutions (see Supporting Information).

In summary, the absorption and emission maxima are solvent dependent as expected for donor‐acceptor (D‐A) compounds in which the boron center is the electron‐acceptor. The electron‐donor for the neutral compounds is the dimethylamine moiety. In the case of **4a** and **5c**, the mesityl moiety acts as an electron‐donor, whereas for **6c**, only the 2,6‐dimethylphenyl motif serves as the donor, as supported by DFT and TD‐DFT calculations. Upon methylation of the neutral *bis*‐triarylboranes, the bithiophene becomes the electron‐donating motif. The absorption and emission maxima of the cationic *bis*‐triarylboranes **Cat^1+^**, **Cat^2+^**, **Cat(i)^2+^**, and **Cat^3+^** are bathochromically shifted compared to their respective *mono*‐triarylboranes. However, no additive effect from combining the triarylboranes with the bithiophene bridge was observed for the molar extinction coefficient. Thus, it can be concluded that the photophysical properties mainly result from the interaction of the most electron‐poor boron center with the most electron‐rich donating moiety for all compounds investigated.

### Electrochemistry

Electrochemical measurements were performed in the respective solvent (THF or acetonitrile) with [*n*Bu_4_N][PF_6_] as the electrolyte and a scan rate of 250 mV s^‐1^. Each measurement is referenced to the ferrocene/ferrocenium (Fc/Fc^+^) couple. The redox potentials of uncharged and cationic triarylboranes **4a**–**6a**, **5c**, and **6c** were examined as well as those of the neutral and cationic bithiophene *bis*‐triarylboranes **Neut0**–**4** and **Cat^1+^**–**Cat^2+^**. Potentials of the neutral compounds were measured in THF, and those of the cationic compounds in MeCN. All cyclic voltammograms are shown in the Supporting Information (Figures S82–S99) together with an extended discussion thereof. The electrochemical data are summarized in Table 2. No oxidation potentials are expected for **4a**, **5c** and **6c**, within the electrochemical window of the solvent used. In the case of **5c** and **6c**, the presence of ca. 0.5 % starting material was confirmed by ^1^H NMR spectra. Thus, the minor oxidation waves observed for both compounds can be attributed to the presence of non‐ or partially methylated starting materials.

**Table 2:** Reduction and oxidation potentials for neutral compounds **4a**–**6a**, **Neut0**–**Neut4** determined in THF, and cationic compounds **5c, 6c, Cat^1+^**–**Cat^4+^** determined in MeCN.

	*E*_1/2_ (red1) [V]	*E*_1/2_ (red2) [V]	*E*_1/2_ (red3) [V]	*E*_1/2_ (ox1) [V]	*E*_1/2_ (ox2) [V]
**4a**	−2.67				
**5a**	−2.78			0.34	
**6a**	−2.86			0.28	0.51 (irrev.)
**5c**	−2.24				
**6c**	−2.02				
**Neut0**	−2.31	−2.95 (irrev.)			
**Neut1**	−2.34	−3.03 (irrev.)		0.39 (irrev.)	
**Neut2**	−2.33	−2.96 (irrev.)		0.32 (irrev.)	
**Neut(i)2**	−2.39	−3.09 (irrev.)		0.34 (irrev.)	
**Neut3**	−2.40	−3.11 (irrev.)		0.40 (irrev.)	0.49 (irrev.)
**Neut4**	−2.40	−3.03 (irrev.)		0.32 (irrev.)	
**Cat^1+^**	−2.13			0.64	0.98
**Cat^2+^**	−1.94	−2.18		0.66	0.99
**Cat(i)^2+^**	−2.07	−2.76 (irrev.)		0.66	0.98
**Cat^3+^**	−1.94	−2.07	−2.80 (irrev.)	0.67	1.01
**Cat^4+^**	−1.93	−2.78 (irrev.)		0.68	1.10

For the triarylboranes **4a**, **5a**, **6a**, **5c**, and **6c** one partially reversible 1e^−^ reduction potential for the boron center and one 1e^−^ oxidation potential per amino group was found. The reduction potentials decrease with increasing electron density at the boron center which increases in the order **6c**<**5c**<**4a**<**5a**<**6a**. The electrochemical data are in good agreement with values reported by Gabbaï and coworkers for very similar trimethylammonium‐substituted triarylboranes. However, the trend that each trimethylammonium group leads to a decrease of 0.26 V in MeCN^[35]^ was only observed for the addition of the second cationic group (**5c** vs. **6c**) but not for the addition of the first one (**4a** vs. **5c**).

**Figure 7:**
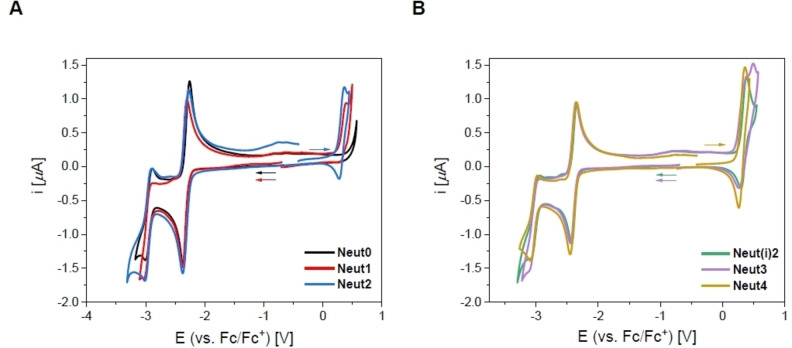
Cyclic voltammograms of **Neut0**–**Neut4** in THF.

For all neutral *bis*‐triarylboranes in THF (Figure 7), one partially reversible reduction at ca. −2.35 V was observed, which consists of two simultaneous 1e^−^ reductions (Table S20) suggesting negligible delocalization between the two boron atoms. Furthermore, a second (irreversible) 1e^−^ reduction at ca. −3.05 V was observed. For all dimethylamino‐substituted *bis*‐triarylboranes, an irreversible oxidation occurred at ca. 0.35 V; only **Neut3** has an additional irreversible oxidation at 0.49 V.

**Figure 8:**
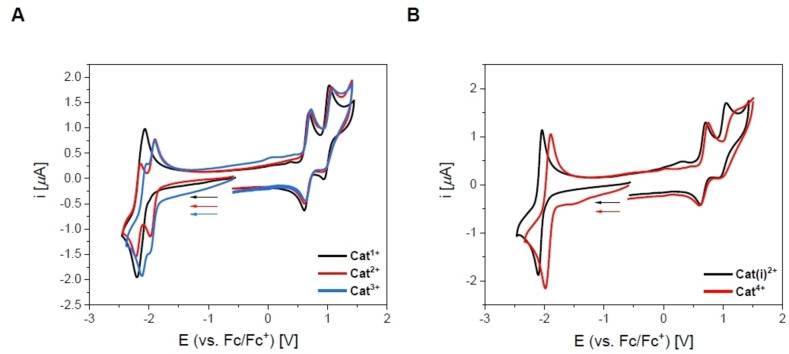
Cyclic voltammograms of **Cat^1+^**–**Cat^4+^** in MeCN.

For all cationic *bis*‐triarylboranes (Figure 8), two fully reversible 1e^−^ oxidation processes were observed at ca. 0.65 V and 1.0 V, respectively, resulting from the bithiophene bridge. However, for **Cat^1+^**, **Cat^2+^**, and **Cat^3+^**, two partially reversible 1e^−^ reductions were observed for each compound whereas **Cat(i)^2+^** and **Cat^4+^** display only one reduction potential. These result from two simultaneous 1e^−^ reductions (Table S20), as the two boron centers in the latter are the same on both sides of the molecule. It was not possible to improve the resolution of the quasi‐reversible reduction waves of **Cat^1+^** by pulse voltammetry experiments (Figure S94D).

### HOMO‐LUMO gaps

HOMO‐LUMO gaps (ΔE_calc_, ΔE_opt_, ΔE_CV_) were obtained from DFT calculations and experimental values. The absorption spectra were used to determine the optical bandgap ΔE_opt_ and ΔE_CV_ was calculated from the HOMO and LUMO energies according to a literature procedure (see Supporting Information).[^46^, ^47^, ^48^, ^49^] The results are summarized in Table 3. ΔE_opt_ does not change significantly within a series of neutral or cationic *bis*‐triarylboranes but decreases by ca. 0.5 eV upon introduction of the strong dimethylamino electron donor in the *mono*‐triarylboranes, due to destabilization of the HOMO more than the LUMO, as supported by DFT calculations. The values of ΔE_opt_ and ΔE_CV_ are of the same magnitude and display the same trends. For the cationic compounds, only the cationic moiety was used in the calculations, so ion‐ion‐interactions were not considered.

**Table 3:** Comparison of HOMO‐LUMO gap energies obtained from DFT calculations (ΔE_calc_), absorption (ΔE_opt_) and CV (ΔE_CV_) measurements.

	ΔE_calc_ [eV]	ΔE_opt_ [eV]	ΔE_CV_ [eV]
**4a**	4.34	3.49	
**5a**	3.66	2.92	3.12
**6a**	3.63	2.92	3.14
**5c**	3.87	3.42	
**6c**	3.72	3.47	
**Neut0**	3.01	2.65	
**Neut1**	2.99	2.65	2.73
**Neut2**	2.99	2.67	2.65
**Neut(i)2**	3.07	2.66	2.73
**Neut3**	3.07	2.66	2.80
**Neut4**	3.10	‐ ^[a]^	2.72
**Cat^1+^**	2.09	2.62	2.77
**Cat^2+^**	1.32	2.56	2.60
**Cat(i)^2+^**	2.58	2.58	2.73
**Cat^3+^**	1.83	2.56	2.61
**Cat^4+^**	–^[b]^	2.55 ^[c]^	2.60

[a] No data were reported for this compound.^[26]^ [b] A different basis set was used.^[26]^ [c] Calculated from reported absorption spectra.^[26]^

### Singlet oxygen sensitization

The ability to sensitize singlet oxygen in O_2_‐saturated acetonitrile solutions was investigated for the triarylboranes **4a**, **6c**, and **5c** as well as for the charged *bis*‐triarylboranes **Cat^1+^**–**Cat^4+^** by monitoring the weak ^1^O_2_ phosphorescence emission at 1275 nm vs. that obtained from a solution of the known sensitizer perinaphthenone^[50]^ with an estimated error of ±0.1.

Triarylboranes **4a**, **6c**, and **5c** sensitize ^1^O_2_ with efficiencies of 0.3, 0.5, and 0.6, respectively, (Figures S79) which increases with increasing number of trimethylammonium substituents (Table S17). The corresponding fluorescence quantum yields in acetonitrile decrease in the same order from 0.34 to 0.11 and 0.10. Thus, the sum of singlet oxygen efficiency *Φ*_Δ_ and fluorescence quantum *Φ*_f_ yield for these compounds is ca. 0.6 (Table S17).

**Figure 9:**
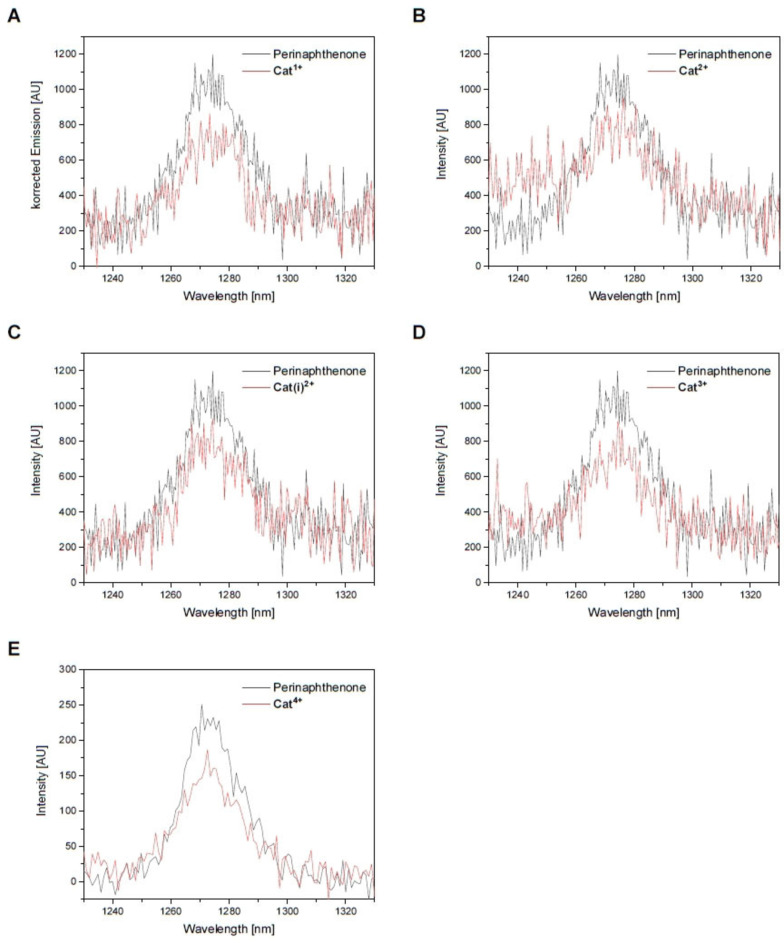
Emission spectra from singlet oxygen sensitization of **A) Cat^1+^**, **B) Cat^2+^**, **C) Cat(i)^2+^**, **D) Cat^3+^**, and **E) Cat^4+^** relative to perinaphthenone in acetonitrile.

The charged *bis*‐triarylboranes sensitize ^1^O_2_ with efficiencies of 0.6, 0.8, 0.7, 0.6, and 0.6 for **Cat^1+^**, **Cat^2+^**, **Cat(i)^2+^**, **Cat^3+^**, and **Cat^4+^**, respectively (Figure 9, Table 4). The values for **Cat^2+^** and **Cat^3+^** may be overestimated due to tailing of the compound's emission into the NIR region of the singlet oxygen emission (Figure S80), which is the reason for the raised baseline between 1230 and 1260 nm in Figures 9B and 9D. Thus, *Φ*_Δ_ of ca. 0.6 is estimated for all charged *bis*‐triarylboranes.

**Table 4:** Summary of singlet oxygen sensitization of **Cat^1+^**–**Cat^4+^** relative to perinaphthenone in acetonitrile.

Compound	*Φ*_Δ_	*Φ*_f_
**Cat^1+^**	0.6	0.34
**Cat^2+^**	0.8 ^[a]^	0.48
**Cat(i)^2+^**	0.7	0.35
**Cat^3+^**	0.6 ^[a]^	0.46
**Cat^4+^**	0.6	0.41^[26]^

[a] Values obtained from singlet oxygen sensitization measurements overestimate the actual value due to tailing of the emission spectrum of the compound between 1230 nm and 1330 nm.

The S_1_ excited states of **Cat^1+^**–**Cat^4+^** thus decay ca. 40 % by fluorescence and ca. 60 % by intersystem crossing to the triplet state, which very efficiently sensitizes ^1^O_2_. Attaching two triarylboranes to a bithiophene motif leads to a more efficient energy transfer to triplet oxygen than for the *mono*‐triarylboranes. Thus, interactions with DNA, RNA and DNApore and cell imaging studies were only examined for the cationic *bis*‐triarylboranes.

### Studies in buffered solutions

**Figure 10:**
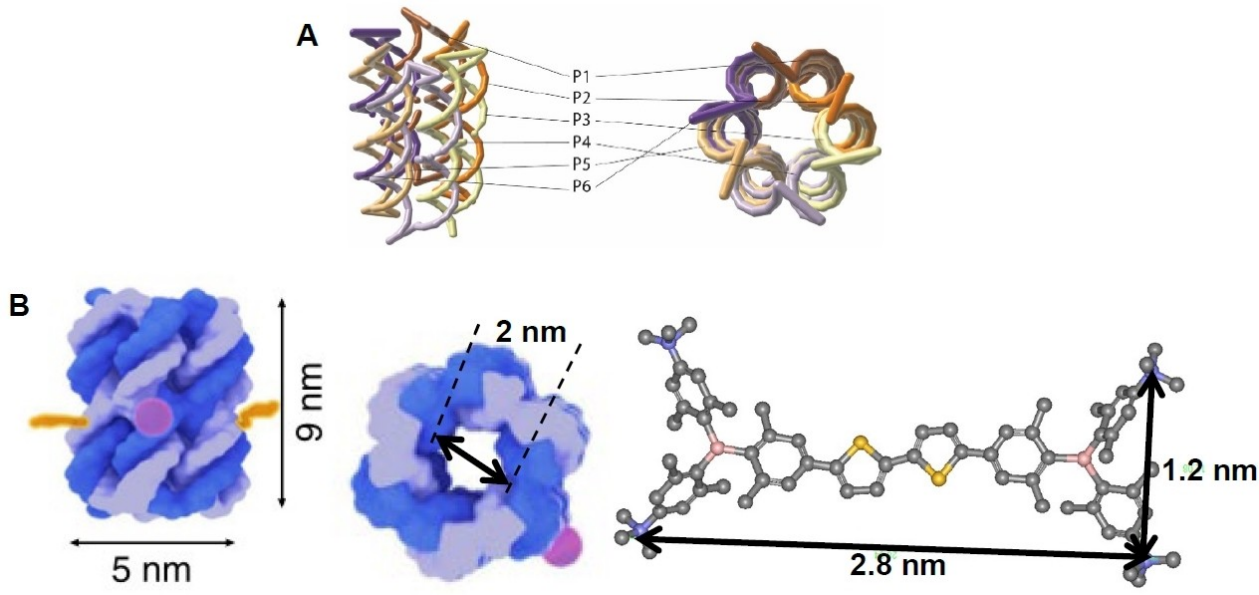
**A)** Schematic presentation of DNApore. The composition of oligonucleotides P1‐P6 is given in Table S21. **B)** Schematic depiction showing that **Cat^4+^** and the analogues **Cat^1+^**–**Cat^3+^** (width ca. 1.2 nm) fit into the cavity of the DNApore (width ca. 2 nm).

Interactions of the cationic *bis*‐triarylboranes **Cat^1+^**–**Cat^3+^** and naturally occurring calf thymus DNA (ctDNA; typical B‐helical structure with a balanced ratio of GC‐(48 %) and AT‐(52 %) base pairs), double stranded (ds) poly rA–poly rU (pApU; A‐helical structure characterized by its major groove being available for binding of bulky small molecules) and DNApore (Figure 10A) were investigated in aqueous buffered solutions. Experimental details and all spectra are given in the Supporting Information. DNApore was chosen as **Cat^1+^**–**Cat^4+^** should fit in the cavity of the structure (Figure 10B).

The stability of **Cat^1+^**–**Cat^3+^** in buffered solutions was confirmed by temperature dependent absorption and emission spectra (Figures S101–S108).

**Figure 11:**
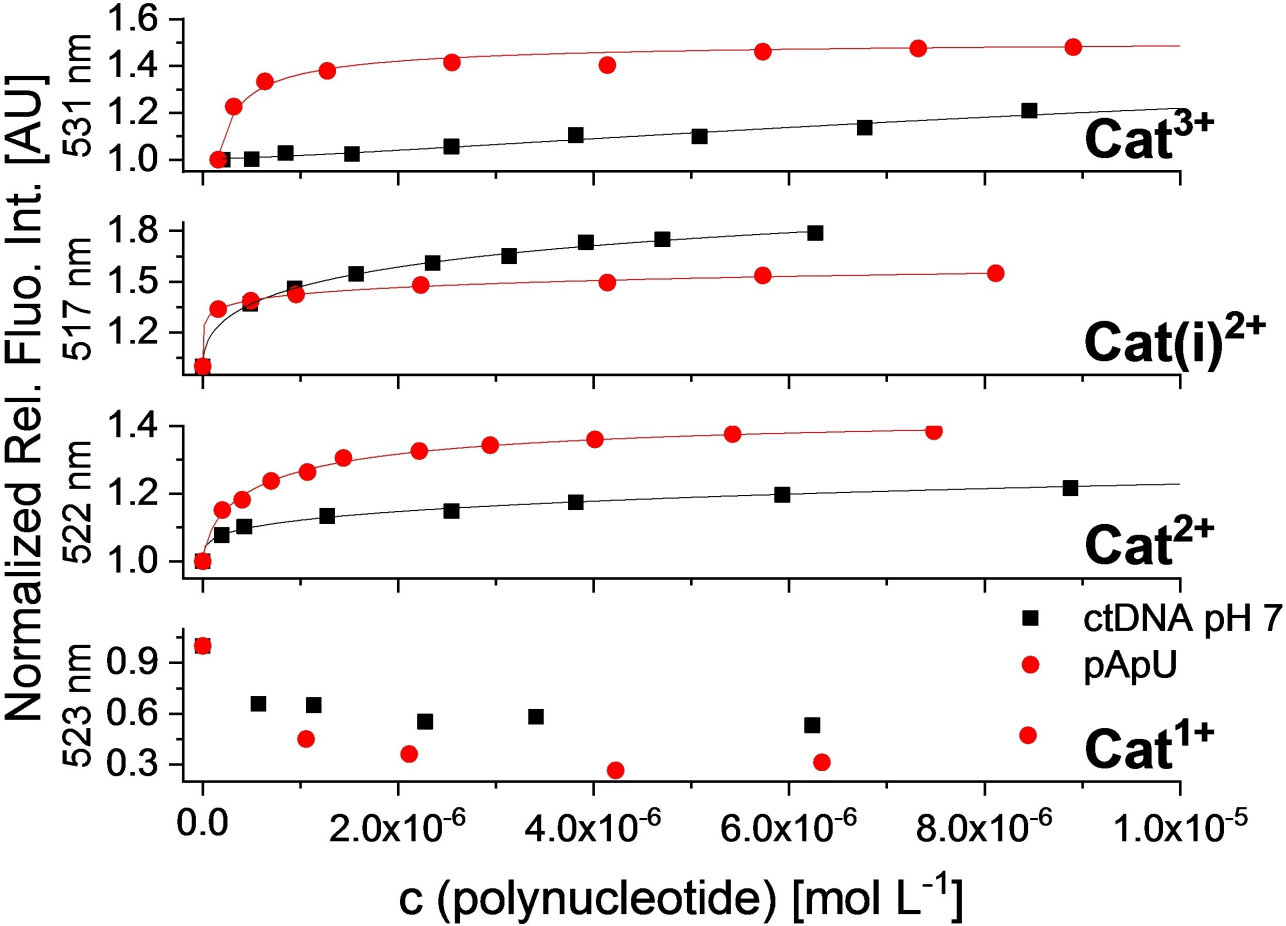
Normalized fluorimetric titration of **Cat^1+^** (c=1×10^−7^ mol L^‐1^; *λ*_exc_=414 nm), **Cat^2+^** (c=1×10^−7^ mol L^‐1^; *λ*_exc_=422 nm), **Cat(i)^2+^** (c=1×10^−7^ mol L^‐1^; *λ*_exc_=417 nm), **Cat^3+^** (c=1×10^−7^ mol L^−1^; *λ*_exc_=421 nm) with ctDNA and pApU at pH 7.

**Figure 12:**
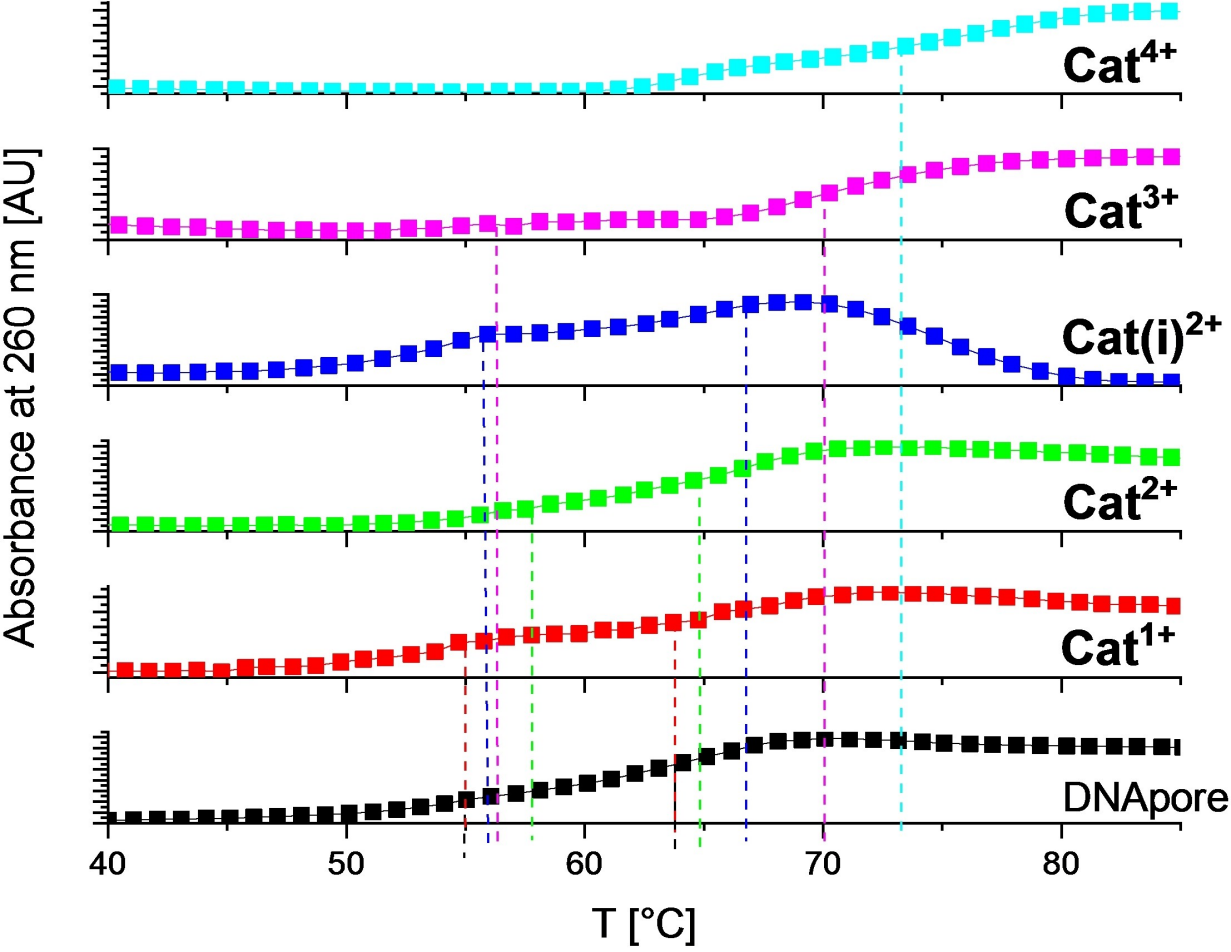
Thermal denaturation curves of DNApore (c=2×10^−5^ M at r_[dye]/[DNApore]_=0.25) at pH 8.0 (15 mM Tris‐HCl, 300 mM KCl) upon addition of **Cat^1+^**–**Cat^4+^**. Error in ΔT_m_ values: ±0.5 °C.

**Figure 13:**
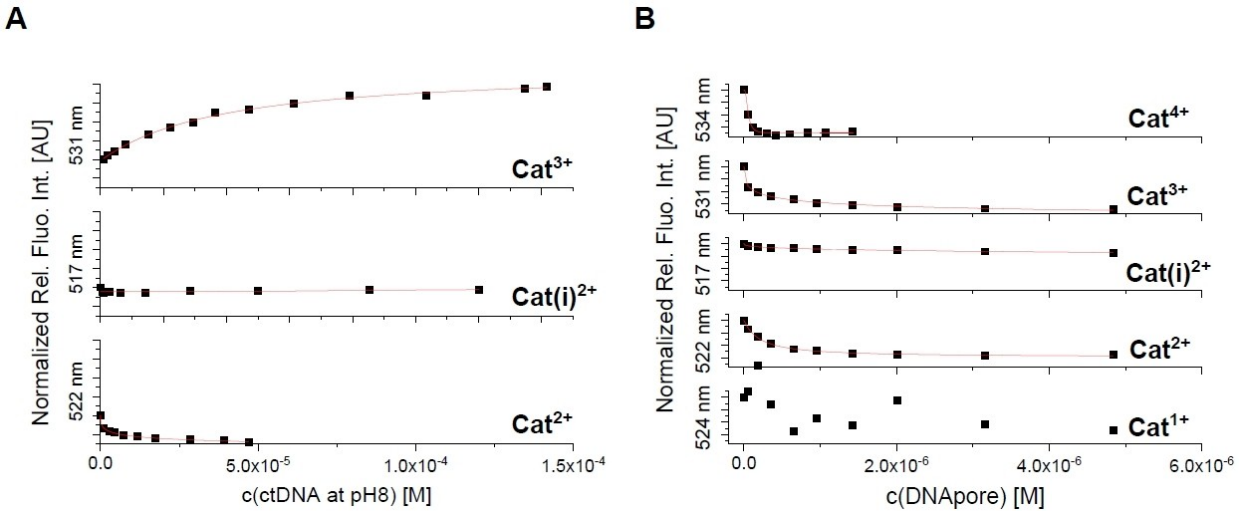
**A)** Normalized fluorimetric titration of **Cat^2+^** (c=5×10^−7^ M; *λ*_exc_=422 nm), **Cat(i)^2+^** (c=5×10^−7^ M; *λ*_exc_=417 nm), and **Cat^3+^** (c=5×10^−7^ M; *λ*_exc_=421 nm) with ctDNA (pH 8 in 15 mM Tris‐HCl, 300 mM KCl) and of **B) Cat^1+^** (c=5×10^−7^ M; *λ*_exc_=414 nm), **Cat^2+^** (c=5×10^‐7^ M; *λ*_exc_=422 nm), **Cat(i)^2+^** (c=5×10^−7^ M; *λ*_exc_=417 nm), **Cat^3+^** (c=5×10^−7^ M; *λ*_exc_=421 nm), and **Cat^4+^** (c=4×10^−7^ M; *λ*_exc_=425 nm) with DNApore (pH 8 in 15 mM Tris‐HCl, 300 mM KCl).

**Figure 14:**
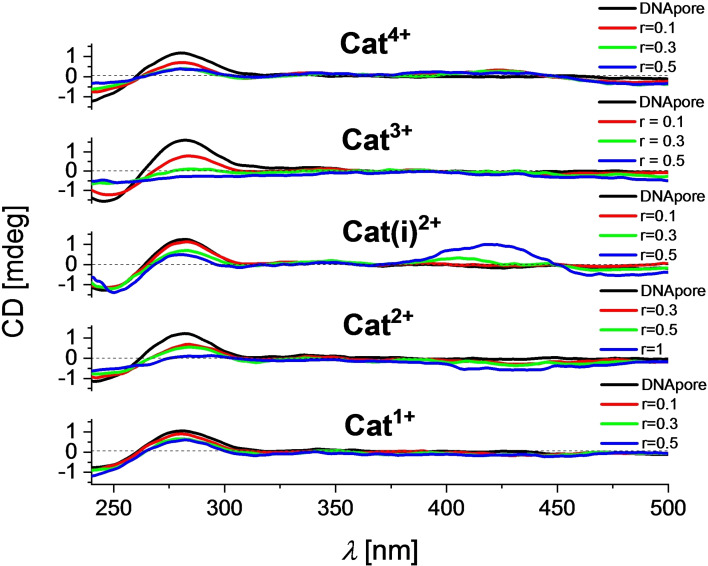
CD titration of DNApore (c=5×10^−5^ M) with **Cat^1+^**–**Cat^4+^** at molar ratios r_[dye]/[polynucleotide]_=0.1–1 in 15 mM Tris‐HCl, 300 mM KCl solutions at pH 8.

Changes in the fluorescence of **Cat^1+^**–**Cat^3+^** upon addition of increasing amounts of DNA, or RNA were studied at pH 7 and/or pH 8. In combination with the results of thermal denaturation (T_m_) and circular dichroism (CD) experiments of the polynucleotides, which are known to be unique for the respective molecule, conclusions about the mode of interaction (intercalation, major or minor groove binding, or external binding) can be drawn.[^51^, ^52^, ^53^, ^54^] For example, increasing denaturation temperatures of polynucleotides in the presence of small molecules, as observed for our *bis*‐triarylboranes **Cat^2+^**–**Cat^3+^**, suggest non‐covalent binding of the latter, characterized by the type of interactions which can increase the stability of DNA/RNA (*e. g*. intercalation, H‐bonding, and/or cation‐anion interactions). However, the emission of **Cat^1+^** showed negligible changes in the presence of any polynucleotide at any pH (Figure 11, Figure 13, Table 6). In some cases, precipitation of **Cat^1+^** was observed. In addition, the denaturation temperatures (Figure 12, Figure S109, Figure S125, Table 5) or the CD spectra (Figures S121, Figure S141, Figure 14) of the polynucleotides were not influenced by the presence of the *mono*‐cation. This behavior is similar to that of neutral analogues investigated previously.^[37]^ Thus, one positive charge is not sufficient for the *bis*‐triarylboranes to provide efficient interaction with polynucleotides.

**Table 5:** ▵T_m_ values ^[a]^ (°C) of polynucleotides upon addition of **Cat** ^1+^–**Cat** ^4+^ at pH indicated.

	r ^[b]^	ctDNA pH 7	polyAU pH 7	ctDNA pH 8	DNA‐pore pH 8
**Cat^1+^**	0.1	0	0	‐	–
	0.2	0	0	–	‐
	0.25	–	–	0.7	0/0
	0.3	0	0	–	–
**Cat^2+^**	0.1	0	0	–	–
	0.2	5.9	2.0	–	–
	0.25	–	–	8.7	3.2/1.2 ^[c]^
	0.3	11.8	11.1	–	–
**Cat(i)^2+^**	0.1	1.7	1.3	–	–
	0.2	2.9	13.0	–	–
	0.25	‐	–	5.5	1.2/3.3 ^[c]^
	0.3	3.8	13.0	–	–
**Cat^3+^**	0.1	3.6	0.6	–	–
	0.2	3.5	6.0	–	–
	0.25	–	–	3.4	1.4/6.6 ^[c]^
	0.3	3.2	5.5	–	–
**Cat^4+^**	0.1	7.3^[38]^	9.5^[38]^	–	–
	0.25	–	–	–	8.0

[a] Error of ▵T_m_=±0.5 °C; [b] r=[compound]/[polynucleotide]; [c] Biphasic transitions.

### Interaction of Cat^2+^–Cat^3+^ with ctDNA, and pApU at pH 7

In the presence of **Cat^2+^**, **Cat(i)^2+^**, and **Cat^3+^**, T_m_ is increased compared to pure ctDNA or pApU at pH 7. Thus, these three compounds efficiently bind to ctDNA and pApU and stabilize these polynucleotides (Table 5; Figure S110–S112). However, the stabilizing effects of **Cat^2+^**, **Cat(i)^2+^**, and **Cat^3+^** are lower than that reported for **Cat^4+^**.^[38]^

Upon addition of ctDNA or pApU, the emission of **Cat^2+^**–**Cat^3+^** increases depending on the structure of the polynucleotide and the number of charges. Using the Scatchard equation^[55]^ and the McGhee, von Hippel formalism,^[56]^ binding constants were obtained, which are very similar for all compounds and polynucleotides investigated (Table 6). Such non‐selective affinity with respect to the secondary structure of the ds‐polynucleotide and the composition of its respective base pairs was reported previously for structurally related compounds,[^36^, ^37^, ^38^] and suggests that **Cat^2+^**, **Cat(i)^2+^**, and **Cat^3+^** bind in the minor groove of DNA or the major groove of RNA.

**Table 6:** Binding constants^[a]^ (log *Ks*) of **Cat^1+^**, **Cat^2+^**, **Cat(i)^2+^**, **Cat^3+^**, and **Cat^4+^** with polynucleotides calculated from fluorimetric titrations according to literature procedures.[^55^, ^56^]

		**Cat^1+^**	**Cat^2+^**	**Cat(i)^2+^**	**Cat^3+^**	**Cat^4+^**
pH 7	ctDNA	^[b]^	6.4	6.9	6.5	7.0 ^[c]^
	pApU	^[b]^	7.0	7.8	7.5	∼7 ^[c]^
pH 8	ctDNA	‐	6.8	^[b]^	5.1	‐
	DNApore	^[b]^	7.5	6.7	7.5	7.8

[a] Analyses of titration data by means of the Scatchard equation^[55]^ with von Hippel formalism^[56]^ gave values of the ratio r=[bound compound]/[polynucleotide]=0.2–0.3; for easier comparison, all log *Ks* values were re‐calculated for fixed r=0.25 (ds‐polynucleotides). Correlation coefficients were >0.99 for all calculated *Ks* values. [b] Negligible emission change. [c] Values reported previously.^[38]^

Upon addition of **Cat^2+^**, **Cat(i)^2+^**, and **Cat^3+^** to ctDNA or pApU, a small decrease of the intensity of the CD spectra between 230 nm and 300 nm (Figures S122–S124) was observed. This can be attributed to an unwinding of the double helix of the polynucleotides upon insertion of the *bis*‐triarylboranes. In contrast to the previously reported tetra‐cationic compound **Cat^4+^**,^[38]^ no ICD bands were observed at 400–500 nm. Thus, **Cat^2+^**, **Cat(i)^2+^**, and **Cat^3+^** might be non‐uniformly oriented with respect to the chiral axis of the polynucleotide, but it was not possible to derive direct structural information. Nevertheless, in analogy with our previous binding studies,[^36^, ^37^, ^38^] groove binding of **Cat^2+^**, **Cat(i)^2+^**, and **Cat^3+^** can be assumed.

### Interaction of Cat^1+^–Cat^3+^ with ctDNA, and DNApore at pH 8

Similarly, as described above for sodium cacodylate solutions, thermal denaturation temperatures of ctDNA and DNApore in Tris‐buffer at pH 8 (15 mmol L^‐1^ Tris‐HCl, 300 mmol L^−1^ KCl) were increased in the presence of **Cat^2+^**, **Cat(i)^2+^**, **Cat^3+^**, and **Cat^4+^** (Table 5, Figure 12). The denaturation curve of DNApore is slightly biphasic, most likely due to the presence of two different conformers. Both transitions were stabilized by interaction with the *bis*‐triarylboranes, with the effect on the melting temperature of DNApore being proportional to the number of cationic charges in the order **Cat^1+^** <**Cat^2+^** ≈**Cat(i)^2+^** <**Cat^3+^** <**Cat^4+^**. The unusual drop of absorbance observed for **Cat(i)^2+^** upon addition of DNA is likely due to precipitation of a **Cat^2+^**/DNA complex upon complete unwinding.

In contrast to the emission increase of **Cat^2+^** and **Cat(i)^2+^** upon addition of ctDNA at pH 7, at pH 8, the emission of these compounds decreases (Figure 13A), while the emission of **Cat^3+^** increases in both solutions. However, upon addition of DNApore, the emission of **Cat^2+^**, **Cat(i)^2+^**, **Cat^3+^**, and **Cat^4+^** decreases (Figure 13B). Binding constants are very similar for the different *bis*‐triarylboranes at pH 8 (Table 6). However, upon changing the pH from 7 to 8, the binding constants of ctDNA are lower, especially for **Cat^3+^**. In contrast, for all compounds, binding constants to the DNApore are larger than those for ctDNA at pH 8. Thus, the chromophores bind more strongly to DNApore than to ctDNA.

Addition of **Cat^1+^** to ctDNA at pH 8 resulted in changes of the CD spectrum at <300 nm which were not observed at pH 7. Thus, a change of the interaction between this *bis*‐triarylborane and ctDNA takes place upon increasing the pH from 7 to 8. For **Cat(i)^2+^** and **Cat^3+^**, a negative band at ca. 425 nm was observed, indicating a perpendicular orientation of the transition moments to the chiral axis of ctDNA at pH 8.

The chiral axis of the DNApore cannot be directly correlated to the usually well‐defined chiral axes of typical ds‐DNAs due to the six intertwined 50‐mer oligonucleotides and, consequently, the tilted angles of the double helices with respect to the central axis of the DNApore. Thus, the following ICD signal assignments do not necessarily match those described in the literature.[^54^, ^57^, ^58^] However, due to the similar structures of the *bis*‐triarylboranes studied herein, and the same experimental procedures applied, the results of these measurements can be used for comparisons within this series of compounds. For **Cat^2+^**, weak, negative ICD bands were observed which is characteristic for a perpendicular orientation of the transition moment of the chromophore with respect to the chiral axis of the DNApore. In contrast to the CD measurements at pH 7, which did not change at wavelengths >300 nm, addition of **Cat(i)^2+^** to DNApore at pH 8 induced strong ICD bands at 400–450 nm (Figure 14). These changes fit the absorption spectra of the corresponding *bis*‐triarylboranes perfectly, which shows that the ICD signals are derived from our chromophores. The positive ICD bands suggest a tilted orientation by ca. 60° to the chiral axis of the DNApore. For **Cat^3+^**, complete disintegration of the CD bands of DNApore suggests strong changes of the secondary structure of the DNApore upon binding.

Thus, **Cat^2+^**, **Cat(i)^2+^**, **Cat^3+^**, and **Cat^4+^** interact with all polynucleotides investigated. Although there are some similarities, *e. g*. interaction with DNApore leads to emission decrease of the compounds, the number and distribution of charges affects the interaction of *bis*‐triarylboranes with different polynucleotides. In particular, the induced CD bands of various derivatives (Figure 14) can be used for fine sensing of the DNApore shape. Thus, **Cat^2+^**, **Cat(i)^2+^**, **Cat^3+^**, and **Cat^4+^** are useful probes for further studies of the DNApore structure and interactions with novel ligands.

### Cell toxicity and imaging studies

Studies were conducted on the cationic *bis*‐triarylboranes to see whether they penetrate cell membranes, stain specific organelles therein, or if they have anti‐proliferative effects to A549 and WI38 cells. Confocal microscopy studies showed that the *mono*‐cationic compound **Cat^1+^** does not penetrate the cells, in contrast to **Cat^2+^**–**Cat^3+^**. Thus, this compound was not further investigated in biological studies. For **Cat^2+^**, only preliminary results were obtained, suggesting localization at the cellular membrane and certain compartments in the cell nucleus. However, due to the low stability of this compound in Dulbecco Modified Eagle's Medium (DMEM) containing 10 % fetal bovine serum (FBS), only the staining patterns of **Cat(i)^2+^** and **Cat^3+^** were further investigated.

The cytotoxicity of **Cat(i)^2+^** and **Cat^3+^** was tested using the MTT assay against human lung carcinoma (A549) and normal lung (WI38) cell lines (Figure S146). In the dark, **Cat(i)^2+^** is not cytotoxic in all concentrations tested (0.1 μM, 1 μM, 10 μM). However, when irradiating the incubated cells of both cell lines in the presence of 1 μM **Cat(i)^2+^** at 400–700 nm for 5 min, the viability of the cells was reduced drastically with increasing concentration of **Cat(i)^2+^**.

In the dark, **Cat^3+^** is not cytotoxic to both cell lines in concentrations of 0.1 μM and 1 μM. However, at 10 μM concentrations, **Cat^3+^** is cytotoxic to A549 cells. This toxicity is even more pronounced for WI38 cells. For both cell lines, the toxicity increases under irradiation (400–700 nm) at 1 μM and 10 μM concentrations.

The increased toxicity of these compounds when irradiated with UV light results most likely from their ability to sensitize singlet oxygen (see above) as the latter is a highly reactive compound leading to cell death.^[59]^ This assumption was confirmed by monitoring cells incubated with **Cat^2+^**, **Cat(i)^2+^**, or **Cat^3+^** by confocal microscopy while irradiating at 405 nm at the full power of the light source (LED). Within 2 min, significant changes in the cell morphology were observed (cell blebbing, cell contraction; Figures S148, S150, S152) suggesting strong cellular damage. Simultaneously, the emission of the dyes bleached rapidly within 30 s to 1 min (Figures S147, S149, S151). The stability of the emission increases with the number of cationic charges. After the emission is quenched completely, no additional cell damage was observed. Control experiments on untreated cells did not show any sensitivity to light exposure. This is in good agreement with the cell toxicity of **Cat(i)^2+^** and **Cat^3+^** under UV irradiation found during the MTT assay.

**Figure 15:**
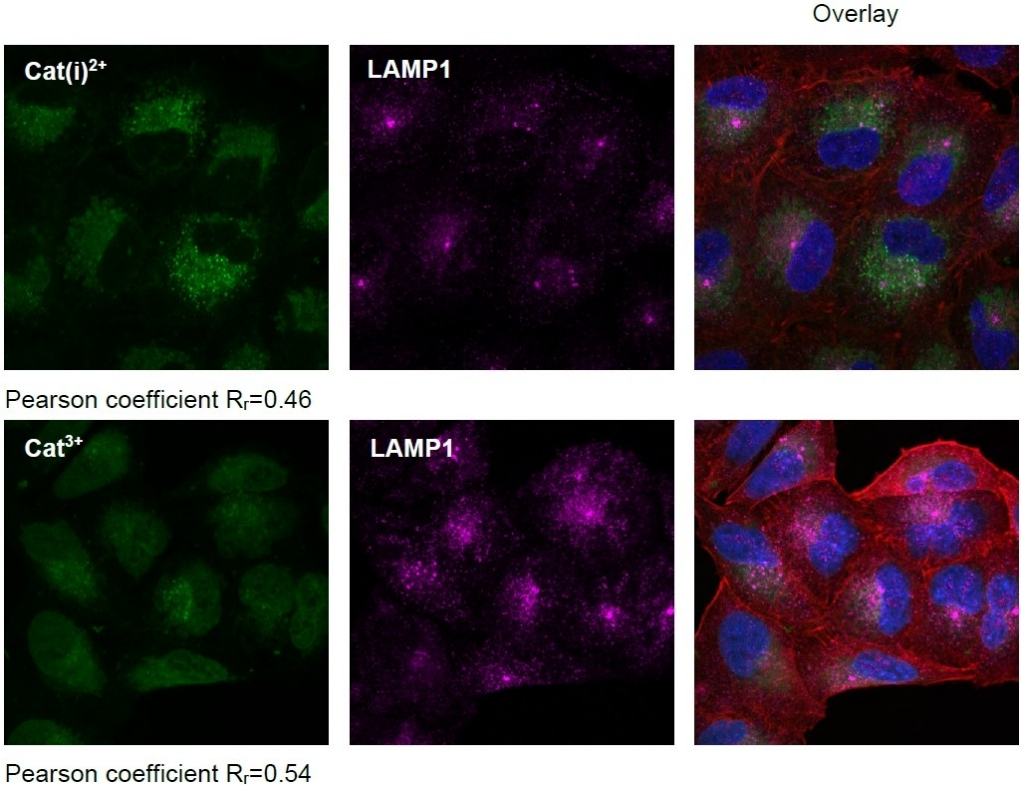
Intracellular localization of **Cat(i)^2+^** and **Cat^3+^** in A549 cells. Co‐localization with lysosomes (LAMP1) was monitored by confocal microscopy. Cells were treated with the *bis‐*triarylboranes at concentrations of 10 μM for 90 min at 37 °C. Nuclei were stained with DAPI. Co‐localization was assessed by determining the Pearson correlation coefficient.

However, when incubating A549 cells for 90 min (37 °C, 5 % CO_2_) with **Cat(i)^2+^**, or **Cat^3+^** and exciting with light (405 nm; LED source), the cells show strong green emission due to the accumulation of the compounds in some organelles in the cytoplasm. The staining patterns of both compounds were further analyzed by co‐localization experiments (Figure 15; Figures S153–S154). The degree of co‐localization was quantified using the Pearson correlation coefficient (R_r_)^[60]^ and corrected by replicate‐based noise correction correlation.^[61]^ For **Cat(i)^2+^** (0.54) and **Cat^3+^** (0.46), the best overlap was obtained for co‐localization with LAMP1, a staining antibody selective for lysosomes. However, the staining pattern of the *bis*‐triarylboranes suggests further interactions with early endosomes and golgi apparati (Figure S153–S154; Tables S23–S24). Thus, these new compounds are not specific for one organelle but are distributed between the organelles mentioned with some selectivity for lysosomes.

## Conclusions

The syntheses of selectively trimethylammonium‐substituted, bithiophene‐bridged, *bis*‐triarylborane chromophores (**Cat^1+^**, **Cat^2+^**, **Cat(i)^2+^**, **Cat^3+^**) and their neutral precursors (**Neut1**, **Neut2**, **Neut(i)2**, **Neut3**) are described. The compounds were isolated in good yields and the influence of different numbers and locations of charges on electrochemical and photophysical properties and cellular imaging was investigated. Photophysical investigations reveal expected behavior for D‐A compounds wherein the boron center is the electron‐acceptor in all compounds with differing electron‐donating moieties, as supported by DFT and TD‐DFT calculations. For the cationic *bis*‐triarylboranes, an increasing hypsochromic shift of the emission spectra with increasing dipole moment was observed in acetonitrile. CV measurements of the neutral and cationic *bis*‐triarylboranes show that the π‐conjugation, and therefore the communication between the boron centers, is improved upon methylation of the dimethylamino groups. Efficient singlet oxygen sensitization was demonstrated for the cationic *bis*‐triarylboranes **Cat^1+^**, **Cat^2+^**, **Cat(i)^2+^**, and **Cat^3+^**. For comparison with the properties of the *bis*‐triarylboranes, selected properties were also investigated for the *mono*‐triarylboranes **4a**‐**6a**, **5c**, and **6c**.

Due to the moderate solubilities of **Cat^1+^**, **Cat^2+^**, **Cat(i)^2+^**, and **Cat^3+^** in pure water, photophysical investigations and biological studies were performed in aqueous environments containing less than 1 % acetonitrile and 0.1 % DMSO, respectively. Studies with DNA and RNA in buffered solutions show negligible binding for the *mono*‐cationic compound **Cat^1+^** but strong binding for **Cat^2+^**, **Cat(i)^2+^**, and **Cat^3+^** within the minor groove of DNA or the major groove of RNA exhibiting a strong fluorescence increase under physiological conditions. The compounds also bind strongly to DNApore, most likely inside the pore. Thus, they could potentially act as a “stopper” of ubiquitous material flow, which DNApore enables when inserted in, *e. g*., bacterial membranes.^[62]^ Due to their induced CD response, **Cat^2+^**, **Cat(i)^2+^**, and **Cat^3+^** might be useful to study the fine structure of DNApore and the interaction with other ligands. Studies with A549 and WI38 cells show all compounds to be cell permeable except for the mono‐cation **Cat^1+^**. Under intense irradiation (400–700 nm), the emission of **Cat^2+^**, **Cat(i)^2+^**, and **Cat^3+^** bleaches rapidly with simultaneous changes of cell morphology, most likely due to in situ formation of singlet oxygen. Thus, the cytotoxicity of the compounds is strongly increased upon irradiation with light (400–700 nm) as demonstrated using the MTT assay. Without irradiation, **Cat(i)^2+^** and **Cat^3+^** are not cytotoxic up to concentrations of 10 and 1 μM, respectively, for both cell lines. Co‐localization experiments of both compounds in A549 cells demonstrate the lysosome as the main but not only accumulation site, since **Cat(i)^2+^** and **Cat^3+^** also localize in other organelles. Thus, the cationic *bis*‐triarylboranes are promising theranostic agents^[63]^ as they combine the potential for photodynamic therapy due to their relatively high singlet‐oxygen sensitizing efficiency with high fluorescence quantum yields for simultaneous imaging of the location of the chromophores in cells or tissues. Furthermore, this study demonstrates that the number of cationic trimethylammonium groups and their distribution influences all properties investigated, including intracellular localization. Thus, when designing new *bis*‐triarylborane chromophores for biological applications, the number and distribution of charges should be considered as well as the bridging unit.

## Crystal structures

“Deposition Number(s) 2072401 (for **4a**) and 2072402 (for **4b**) contain(s) the supplementary crystallographic data for this paper. These data are provided free of charge by the joint Cambridge Crystallographic Data Centre and Fachinformationszentrum Karlsruhe Access Structures service.”

## Conflict of interest

The authors declare no conflict of interest.

## Supporting information

As a service to our authors and readers, this journal provides supporting information supplied by the authors. Such materials are peer reviewed and may be re‐organized for online delivery, but are not copy‐edited or typeset. Technical support issues arising from supporting information (other than missing files) should be addressed to the authors.

Supporting Information
