# Supplementary material for: Bithiophene‐Cored, mono‐, bis‐, and tris‐(Trimethylammonium)‐Substituted, bis‐Triarylborane Chromophores: Effect of the Number and Position of Charges on Cell Imaging and DNA/RNA Sensing
Source: Chemistry. 2021 Aug 31;27(56):14057–72. doi: 10.1002/chem.202102308 (PMC8518794; doi:10.1002/chem.202102308)
Supplement: Supplementary file 1 — Supporting Information [file CHEM-27-14057-s001.pdf]

Chemistry–A European Journal

Supporting Information

Bithiophene-Cored, *mono-*, *bis-*, and *tris-*(Trimethylammonium)-Substituted, *bis*-Triarylborane Chromophores: Effect of the Number and Position of Charges on Cell Imaging and DNA/RNA Sensing

Sarina M. Berger, Jessica Rühe, Johannes Schwarzmann, Alexandra Phillipps, Ann-Katrin Richard, Matthias Ferger, Ivo Krummenacher, Lidija-Marija Tumir, Željka Ban, Ivo Crnolatac, Dragomira Majhen,* Ivan Barišić,* Ivo Piantanida,* Domenik Schleier, Stefanie Griesbeck, Alexandra Friedrich, Holger Braunschweig, and Todd B. Marder*

Electronic Supporting Information

Table of Contents

Overview of Molecular Structures S2
Experimental Section S2
NMR Spectra S31
Crystal Structures S62
Solubility in Water S64
Photophysical Properties S66
  Neutral Triarylboranes S68
  Cationic Triarylboranes S71
  Neutral *bis*-Triarylboranes S73
  Cationic *bis*-Triarylboranes S78
Singlet Oxygen Sensitizing S82
Cyclic Voltammetry S85
  Neutral Triarylboranes S88
  Cationic Triarylboranes S91
  Neutral *bis*-Triarylboranes S93
  Cationic *bis*-Triarylboranes S100
Studies in Buffered Solutions S107
  Physico-hemical Properties of **Cat**$^{1+}$-**Cat**$^{3+}$ in Buffered Solution S107
    Solubility in Sodium Cacodylate at pH 7 .... S107
    Stability of UV/Vis Spectra S107
    Fluorimetric Spectra S112
  Interaction of **Cat**$^{1+}$-**Cat**$^{3+}$ with ctDNA, and pApU at pH 7 .... S116
    Thermal Melting Experiments S116
    Fluorimetric Titrations S118
    Circular Dichroism Experiments S122
  Interaction of **Cat**$^{1+}$-**Cat**$^{4+}$ with ctDNA, and DNApore at pH 8 .... S124
    Thermal Melting Experiments S124
    Fluorimetric Titrations S128
    Circular Dichroism Experiments S133
  Cell Studies S138
    MTT Assay S138
    Photoinduced Cell Damage S139
    Co-localization by Confocal Microscopy S145
DFT and TD-DFT Calculations S147
  Triarylboranes S148
  bis-Triarylboranes S163
References S203

Overview of Molecular Structures

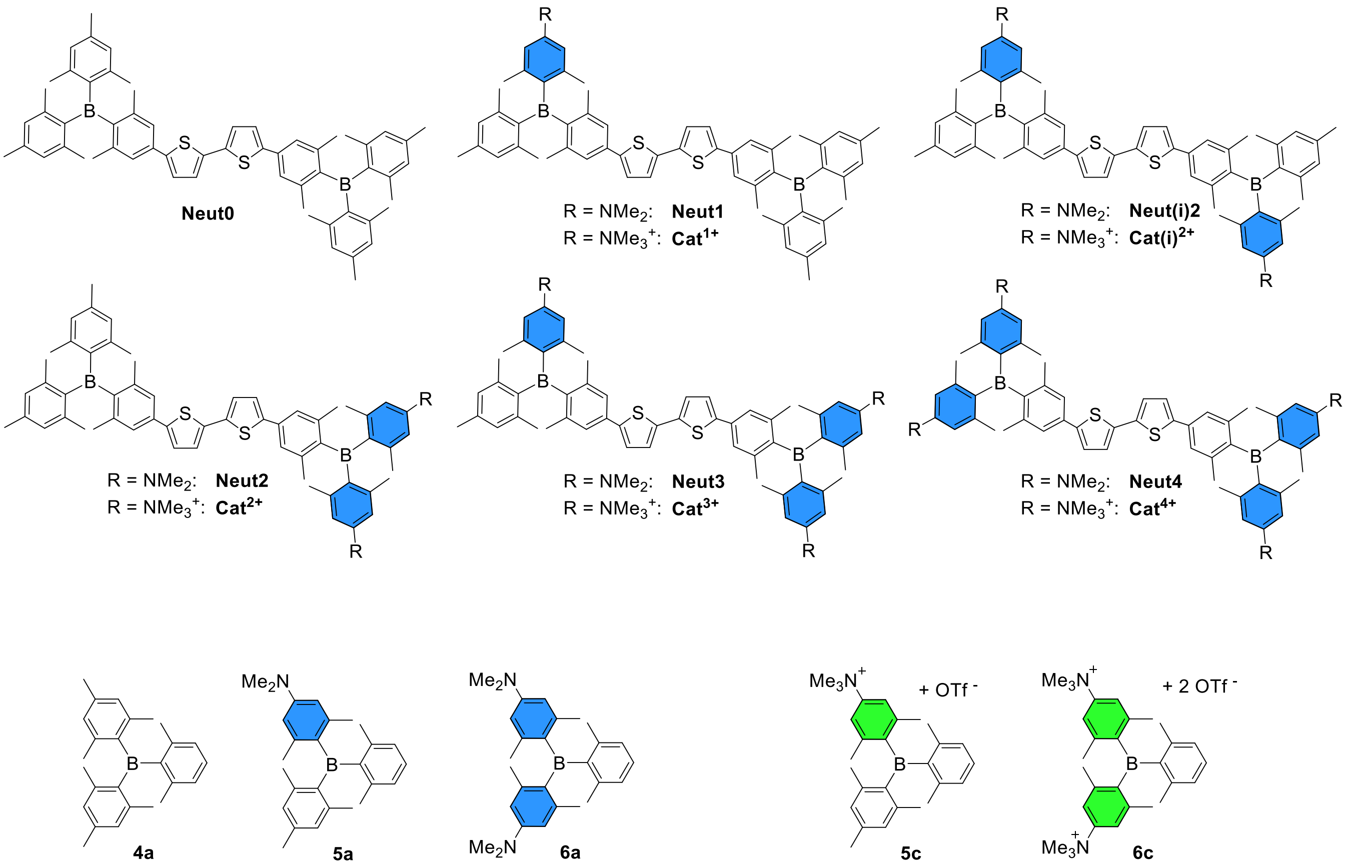

Scheme S1: Molecular structures of investigated compounds.

Experimental Section

General Information

Unless otherwise noted, all reactions were performed under an inert atmosphere of argon using standard Schlenk techniques or glovebox (Innovative Technology Inc.) techniques. For these reactions, oven-dried and additionally flame-dried glassware was used. Unless otherwise stated, every work up procedure described was performed open to the air. Each methylation of **5a**, **6a**, **Neut1**, **Neut2**, **Neut(i)2**, and **Neut3** was done in a new vial with a maximum volume of 5 mL (diameter: 19 mm, height: 40 mm) made from soda-lime glass provided by VWR. Solvents used for reactions under argon (THF, CH_2Cl_2, and Et_2O) were dried, deoxygenated and argon saturated using an Innovative Technology Inc. Pure Solvent Purification System. Deuterated solvents (CD_2Cl_2, CD_3OD, CD_3CN) used for nuclear magnetic resonance spectroscopy were purchased from Sigma Aldrich. *n*-Butyllithium (2.5 M solution in hexane), *t*-butyllithium (1.7 M solution in pentane), 4,4′-di-*tert*-butyl-2,2′-dipyridyl (dtbpy), and methyl triflate were purchased from Sigma Aldrich and used as received. The compound 2-dicyclohexylphosphino-2′,6′-dimethoxybiphenyl (SPhos) was purchased from Oxchem and

used as received. B_2pin_2 was kindly provided by AllylChem Co. Ltd. (Dalian, China). $[Ir(COD)(\mu\text{-}OMe)]_2$,[1] $Pd_2dba_3\cdot CHCl_3$,[2] 5,5'-dibromo-2,2'-bithiophene **3**,[3] *bis*-[4-(*N,N*-dimethylamino)-2,6-dimethylphenyl]-2,6-dimethylphenylborane **6a**,[4] *bis*-[4-(*N,N*-dimethylamino)-2,6-dimethylphenyl]-2,6-dimethyl-4-(4,4,5,5-tetramethyl-1,3,2-dioxaborolan-2-yl)phenylborane **6b**,[4] 5,5′-*bis*-[4-[*bis*-[4-(*N*,*N*-dimethylamino)-2,6-dimethylphenyl]boryl]-3,5-dimethylphenyl]-2,2′-bithiophene **Neut4**,[4] 5,5′-*bis*-[4-[*bis*-[4-(*N*,*N*,*N*-trimethylammonium)-2,6-dimethylphenyl]boryl]-3,5-dimethylphenyl]-2,2′-bithiophene tetratriflate **Cat^{4+}**,[4] [4-(*N,N*-dimethylamino)-2,6-dimethylphenyl]-(mesityl)-(2,6-dimethylphenyl)]borane **5a**,[5] and [4-(*N,N*-dimethylamino)-2,6-dimethylphenyl]-(mesityl)-[4-(4,4,5,5-tetramethyl-1,3,2-dioxaborolan-2-yl)-2,6-dimethylphenyl]borane **5b**[5] were synthesized according to literature procedures. All other chemicals were obtained from commercial sources and used as received.

Reaction progress was monitored by thin layer chromatography (TLC) using plates pre-coated with a layer of either silica (Polygram® Sil G/UV254) with fluorescent indicator UV254 or aluminum oxide, purchased from Marchery-Nagel. Automated flash column chromatography was performed using a Biotage® Isolera Four system on silica gel (Biotage SNAP KP-Sil or NH cartridges indicated with the cartridge size depending on substance mass according to the Biotage handbook), obtained from Biotage. Solvent gradients were applied as indicated. Solvents were generally removed using a rotary evaporator *in vacuo* at a maximum temperature of 50 °C. Preparative column chromatography was performed using silica gel 60 (0.040 – 0.063 mm) or aluminum oxide 90 (basic, activity I) purchased from Macherey-Nagel as the stationary phase and the solvent mixtures indicated.

^1H, $^{13}C\{^1H\}$, $^{11}B\{^1H\}$ **NMR spectra** were obtained, unless otherwise stated, at ambient temperature using a Bruker Avance 300 III (operating at 300 MHz for ^1H, 75 MHz for $^{13}C\{^1H\}$ and 96 MHz for $^{11}B\{^1H\}$), or a Bruker Avance 500 NMR spectrometer (operating at 500 MHz for ^1H, 125 MHz for $^{13}C\{^1H\}$, and 160 MHz for $^{11}B\{^1H\}$). Chemical shifts (δ) were referenced to solvent peaks as follows. ^1H NMR spectra were referenced *via* residual proton resonances of CD_2Cl_2 (5.32 ppm), CD_3OD (3.31 ppm), acetone-d_6 (2.05 ppm), and CD_3CN (1.94 ppm). ^{13}C NMR spectra were referenced to CD_2Cl_2 (53.84 ppm), CD_3OD (49.00 ppm), acetone-d_6 (29.84 ppm), and CD_3CN (1.32 ppm). ^{11}B NMR signals are quoted relative to external $BF_3\cdot OEt_2$.

Elemental analyses were performed on an Elementar vario MICRO cube elemental analyzer. Note that carbon values obtained for most boron-containing samples differ by approximately 1.5% from calculated ones, possibly due to formation of boron carbide.[6] For the analysis of boron-containing compounds, V_2O_5 was added, except for **7a**, **7b**, **9a**, **5b**, **Neut1**, **Neut3**, **Cat^{1+}**, and **Cat^{3+}**.

High-resolution mass spectrometry was performed with a Thermo Fisher Scientific Exactive Plus Orbitrap MS system. ESI measurements were performed with a HESI source at 50 °C. APCI and ASAP measurements were performed with an ACPI source and Corona needle at 400 °C, unless otherwise noted.

Single-crystal X-ray diffraction. Crystals suitable for single-crystal X-ray diffraction were selected, coated in perfluoropolyether oil, and mounted on MiTeGen sample holders. Diffraction data were collected on Bruker X8 Apex II 4-circle diffractometers with CCD area detectors using Mo-K_α radiation monochromated by graphite (**4b**) or multi-layer focusing mirrors (**4a**). The crystals were cooled using an open flow N_2 Bruker Kryoflex II low-temperature device. Data were collected at 100 K. The images were processed and corrected for Lorentz-polarization effects and absorption as implemented in the Bruker software packages. The structures were solved using the intrinsic phasing method (SHELXT)[7a] and Fourier expansion technique. All non-hydrogen atoms were refined in anisotropic approximation, with hydrogen atoms ‘riding’ in idealized positions, by full-matrix least squares against F^2 of all data, using SHELXL[7b] software and the SHELXLE graphical user interface.[8] The crystal of **4b** was a non-merohedral twin with twin domains rotated by 2.8° around reciprocal axis (0.021 1.000 -0.217) and, hence, twin data reduction was performed. However, only the major domain (87%) was used in the refinement of the crystal structure. Diamond[9] software was used for graphical representation. Crystal data and experimental details are listed in Table S1; full structural information has been deposited with the Cambridge Crystallographic Data Centre: CCDC-2072401 (**4a**) and 2072402 (**4b**).

All **photophysical measurements** were carried out at ambient conditions using HPLC grade solvents and standard quartz cuvettes (1 cm x 1 cm cross section). UV/Vis absorption spectra were recorded using an Agilent 1100 diode array UV/Vis spectrophotometer or a Perkin Elmer LAMBDA 465 UV/Vis spectrophotometer. Excitation, emission, lifetime, and quantum yield measurements were recorded using an Edinburgh Instruments FLSP920 spectrometer equipped with a 450 W Xenon arc lamp, double monochromators for the excitation and emission pathways, and a red-sensitive photomultiplier (PMT-R928P) or a near-IR PMT as detectors. The measurements were made in right-angle geometry mode and all spectra were fully corrected for the spectral response of the instrument. To avoid self-absorption, the concentration of all solutions used for photophysical measurements was lower than 10^{-5} M (OD ca. 0.1 at absorption maximum of lowest energy).

Fluorescence quantum yields of the samples were measured using a calibrated integrating sphere (150 mm inner diameter) from Edinburgh Instruments combined with the FLSP920 spectrometer described above. For solution-state measurements, the absorption maximum of

lowest energy of the compound in the respective solvent was chosen for excitation. The emission spectra were measured with dilute samples (ca. 0.1 OD at the excitation wavelength).

Fluorescence lifetime measurements were conducted using the time-correlated single-photon counting method (TCSPC) on the FLSP920 spectrometer equipped with a high-speed photomultiplier tube positioned after a single emission monochromator. Lifetimes shorter than 0.5 ns were recorded at an Edinburgh Instruments FLS 980 fluorescence lifetime spectrometer equipped with a high speed PMT (H10720) detector using the laser diodes defined below. Measurements were made in right-angle geometry mode, and the emission was collected through a polarizer set to the magic angle (54.8°). Solutions were excited with either a 315 nm (pulse width 932.5 ps), 376 nm (pulse width 72.6 ps) or a 418 nm (pulse width 1.5 ns) pulsed diode laser at repetition rates of 1–5 MHz and counts were recorded at the emission maxima. The choice of laser was based on the largest absorption maximum of the respective compound. Decays were recorded to 10 000 counts in the peak channel with a record length of at least 4 000 channels. The band-pass of the monochromator was adjusted to give a signal count rate of <20 KHz. Iterative reconvolution of the IRF with one decay function and nonlinear least-squares analysis were used to analyze the data. The quality of all decay fits was judged to be satisfactory based on the calculated values of the reduced χ^2 and Durbin–Watson parameters and visual inspection of the weighted and autocorrelated residuals.

Singlet oxygen sensitizing efficiency (Φ_Δ) of **4a**, **5c**, **6c**, and **Cat^{1+}** - **Cat^{4+}** was determined in acetonitrile solutions relative to a solution of perinaphthenone in acetonitrile by measuring the weak emission of $^1O_2$ at ca. 1275 nm. Absorption spectra were recorded using the Perkin Elmer LAMBDA 465 UV/Vis spectrophotometer described above. Emission spectra were recorded using the Edinburgh Instruments FLSP920 spectrometer described above. Excitation was performed using the 450 W Xenon arc lamp while detection was performed using the near-IR PMT detector. Emission wavelengths shorter than 850 nm were removed using an optical filter to remove optical refractions of higher orders of the excitation light. Solutions were O_2 saturated by purging the solutions with pure oxygen for 20 min. Concentrations of solutions were adjusted to obtain an intersection of the absorption spectrum of perinaphthenone and the respective compound at an absorbance of ca. 0.15. The intersection of the spectra was at ca. 341 nm for the triarylboranes and at ca. 400 nm for the *bis*-triarylboranes. The values for the respective compounds were calculated relative to perinaphthenone, which is known to have a Φ_Δ of 1 in acetonitrile,[10] by integration of the respective emission spectra between ca. 1240 nm and 1300 nm.

Molar extinction coefficients (ε) were determined from at least three dilutions of two independently prepared stock solutions (c = 6.3 x 10^{-4} – 5.0 x 10^{-6} M) in the solvent(s) indicated and are average values from these measurements.

Solubility in water was determined for **Cat1+**, **Cat2+**, **Cat(i)2+**, and **Cat3+** by measuring the absorbance of solutions in Millipore water (c = 3.4 M to 2.6 M) which were shaken in the dark for 3 d using a Janke & Kunkel IKA® VIBRAX® VXR. Absorption of the resulting "solutions" was measured using the Perkin Elmer LAMBDA 465 UV/Vis spectrophotometer described above. Solutions were filtered using CLEAR PA-45/13 syringe filters with polyamide (Nylon) membrane and a pore size of 0.45 µm.

Cyclic voltammetry experiments were performed using a Gamry Instruments Reference 600 potentiostat. A standard three-electrode cell configuration was employed using a platinum disk working electrode, a platinum wire as counter electrode, and a silver wire, separated by a *Vycor* tip, serving as the reference electrode. Formal redox potentials are referenced to the ferrocene/ferrocenium ($[Cp_2Fe]^{+/0}$) redox couple as an internal standard. Tetra-*n*-butylammonium hexafluorophosphate ($[n\mathrm{Bu}_4\mathrm{N}][\mathrm{PF}_6]$) was used as supporting electrolyte. Compensation for resistive losses (*iR* drop) was employed for all measurements.

DFT and TD-DFT calculations were carried out with the Gaussian 09 (Rev. E.01) program package[11] and were performed on a parallel cluster system. GaussView 5.0.9 was used to visualize the results, and to measure calculated structural parameters. Mulitwfn[12] was used to plot orbital surfaces (isovalue: ± 0.030 $[ea_0^{-3}]^{1/2}$). The ground-state geometries were optimized using the B3LYP functional[13-15] in combination with the 6-31G(d) basis set.[16-17] The optimized geometries were confirmed to be local minima by performing frequency calculations. Based on these optimized structures, the lowest-energy gas-phase vertical transitions were calculated (singlets, 10 states) by TD-DFT, using the Coulomb-attenuated functional CAM-B3LYP[18] in combination with the 6-31G(d,p) basis set.

Optical properties in sodium cacodylate. UV/Vis absorption spectra were recorded on a Varian Cary 100 Bio spectrometer; excitation and emission spectra were recorded on a Varian Cary Eclipse fluorimeter

Study of interactions with DNA and RNA. Polynucleotides were purchased as noted: poly A – poly U (Sigma), calf thymus (ct)-DNA (Aldrich) and dissolved in sodium cacodylate buffer, (I = 0.05 M, pH 7). The ctDNA was additionally sonicated and filtered through a 0.45 mm filter to obtain mostly short (ca. 100 base pairs) rod-like β-helical DNA fragments.[19] The polynucleotide concentration was determined spectroscopically[20] as the concentration of the phosphates, which corresponds to the concentration of the nucleobase.

Thermal melting experiments were performed on a Varian Cary 100 Bio spectrometer in quartz cuvettes (diameter: 1 cm). The measurements were carried out in aqueous buffer solution at pH 7 (sodium cacodylate buffer I = 0.05 M) or pH 8 (15 mM Tris-HCl, 300 mM KCl), as indicated. Thermal melting curves for ds-DNA, ds-RNA, and their complexes with **Cat1+**,

Cat^{2+}, **Cat(i)$^{2+}$**, **Cat^{3+}**, and **Cat^{4+}** were determined by following the absorption change at 260 nm as a function of temperature.[21] T_m values are the midpoints of the transition curves determined from the maximum of the first derivative and checked graphically by the tangent method. The ΔT_m values were calculated subtracting T_m of the free nucleic acid from T_m of the complex. Every ΔT_m value reported here was the average of at least two measurements. The error in ΔT_m is ±0.5 °C.

Fluorimetric titrations were performed in quartz cuvettes (diameter: 1 cm) on a Varian Cary Eclipse fluorimeter by adding portions of polynucleotide solution into the solution of the compound studied and excitation wavelengths of λ_{exc} >300 nm were used to avoid absorption of excitation light by added polynucleotides. After mixing the polynucleotides with the compound investigated, equilibrium was reached in less than 120 s. Fluorescence spectra were analyzed at an excess of DNA/RNA (r[dye]/[DNA] <0.2) to assure one dominant binding mode. To obtain binding constants (K*s*), titration data were processed by means of non-linear fitting to the Scatchard equation[22] using the McGhee, von Hippel formalism[23] which gave values of the ratio of [bound compound]/[polynucleotide] in the range 0.1–0.3. For easier comparison, all K*s* values were recalculated for the fixed r = 0.25 (for ds-DNA/RNA). Calculated values for K*s* have satisfactory correlation coefficients (>0.99).

Circular dichroism (CD) spectra were recorded on a JASCO J-815 spectropolarimeter at room temperature using quartz cuvettes (diameter: 1 cm) with a scan speed of 200 nm/min. A background spectrum of the buffer was subtracted from each spectrum and each spectrum was the result of three accumulations. CD experiments were performed by adding portions of a stock solution of the compound into the solution of polynucleotide ($c = 2 \times 10^{-5}$ M).

Cells. Experiments were performed using two human cell lines, epithelial human lung adenocarcinoma A549 (ATCC® CCL-185™) and human normal lung fibroblast WI-38 (ATCC® CCL-75™). Both cell lines adhere to plastic and glass surfaces and are maintained in the culture under same conditions. Cells were grown in Dulbecco Modified Eagle's Medium (DMEM, Sigma Aldrich, USA) supplemented with 10% of fetal bovine serum (FBS, Sigma Aldrich, USA) at 37 °C and 5% CO_2 in a humidified atmosphere. Cells were passaged twice per week to retain maximum confluence of 70-80%. Cells exhibiting normal morphology without any contamination signs were kept in culture and used in all further experiments. Two biological replicates were performed for all experiments.

Cytotoxicity assay. Cytotoxic effect of the compounds was analyzed by the MTT assay.[24] The compounds were dissolved in an appropriate volume of dimethyl sulfoxide solution (DMSO) under sterile conditions, to obtain a stock solution of 10 mM concentration. Solutions were kept in the dark and stored at +4 °C to prevent degradation. Prior to each assay, fresh

working solutions were prepared from stock solutions by diluting it with DMEM. Cells were seeded on 96 well plate at a concentration of 7 × 10^3 cells/well in 100 µL of DMEM (10% FBS) and left in the incubator overnight (37 °C, 5% CO_2). The next day, 100 µL of the working solution was added to the wells. The final concentration of compounds was obtained in the total volume of 200 µL/well. All conditions were tested in quadruplicates. Cells treated with the same dilutions of DMSO represented the control, while cells treated only with DMEM (10% FBS) represented the negative control. The plate was then incubated for the next 72 h (37 °C, 5% CO_2). After the incubation, the medium was removed, and 40 µL of MTT solution was added to each well. The plate was incubated for 3 h, allowing the formazan crystals to form. After 3 h, 170 µL of DMSO was added in each well and the system was shaken for 20 min, allowing the crystals to dissolve. The absorbance of MTT-formazan product was measured with a microplate reader at 600 nm. The absorbance value directly correlates with the cell survival. For irradiation experiments, cells in MTT-prepared plates (see above), treated with the compounds studied, were irradiated for 5 min in a Luzchem reactor with visible light (400-700 nm, 8 lamps, total ≈8 W, dose: 50.6 mW m^{-2}) at ca. 18 cm distance between the lamps and the cell-plate, 24, 48 and 72 h after starting the MTT test.

Co-localization assay. A549 cells were seeded on glass slides in 24 well plates at a concentration of 3 × 10^4 cells/well and incubated in DMEM (37 °C, 5% CO_2) for 48 h, allowing cells to attach to the glass surface and to multiply. Then, cells were treated with a 10 µM solution of the compound and incubated for 90 min at 37 °C. After incubation, cells were fixed with 2% paraformaldehyde (PFA, 12 min, r.t.), washed with PBS (3 x), permeabilized with 0.1% Triton/PBS (2 min, r.t.), washed with PBS (3 x), blocked in 3% BSA (30 min, r.t.), and incubated with primary antibody against early endosome antigen 1 (EEA1, Cell Signaling Technology, #2411, rabbit, 1:100 in 5% BSA), Golgi Apparatus (GM130, Cell Signaling Technology, #12480, rabbit, 1:2500 in 5% BSA) or Lysosome Associated Membrane Protein 1 (LAMP-1, abcam, ab2417, rabbit, 1:250 in 5% BSA). After incubation with primary antibody, cells were washed with PBS, and then incubated in fluorescently labeled anti-rabbit secondary antibody (AF647) (Cell Signaling Technology, #4414, 1:1000 in 5% BSA). Slides were incorporated in DAPI containing mounting medium. Actin filaments were labeled with Alexa Fluor® 555 Phalloidin (Cell Signaling Technology, #8953). Prepared slides were kept in the dark and visualized using a Leica SP8 X confocal microscope (Leica Microsystems). Co-localization was assessed by determining the Pearson correlation coefficient.

Live cell imaging. Live imaging of the cells treated with the compounds was performed on the A549 cell line. Cells were seeded in Ibidi imaging cell chambers (Ibidi®) in 500 µL of medium, at a concentration of 5 × 10^4 cells/well, and left in the cell incubator for 48 h (37 °C, 5% CO_2).

After two days, cells were treated with 10 μM solution of the compound to be studied and left in the cell incubator for 90 min to allow the compound to enter the cells.

Synthesis

Preparation of DNA nanopores

Oligonucleotides (P1, P2, P3, P4, P5, P6; 50% G-C pairs; exact composition of DNApore: Table S21) were dissolved in Tris-HCl buffer, pH 8 (15 mM Tris-HCl, 300 mM KCl), $c(P_i)$ = 2 x 10^{-2} M. All concentrations are expressed as the concentration of phosphates (same as nucleobases). Equimolar concentrations of each oligonucleotide (5 x 10^{-4} M) were mixed and heated in a UV/Vis thermo-block at 95 °C for 15 min, then slowly cooled (0.5 °C per minute) to 10 °C. The overall DNA nanopore concentration c(P1-6) = 3 x 10^{-3} M. Nanopore formation was confirmed by thermal melting experiments (UV/Vis-monitored and DSC-monitored), showing clear and reproducible denaturation profiles.

Dimesityl-2,6-dimethylphenylborane 4a

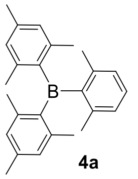

4a

The Grignard reagent was freshly prepared from magnesium (1.94 g, 79.7 mmol) and 2-bromomesitylene (12.0 mL, 79.7 mmol) in dry THF (40 mL) and, after cooling to 0 °C for 30 min, boron trifluoride etherate (4.3 mL, 35.0 mmol) was added dropwise. The solution was stirred at room temperature for 17 h. Afterwards, the solvent was removed *in vacuo* and the resulting solid was extracted with dry, hot hexane (4 x 40 mL). Removal of the solvent *in vacuo* yielded dimesitylfluoroborane **Mes_2BF** as a colorless solid which was used without further purification in the following step. The NMR spectra recorded match those reported in the literature.[25]

In a second flask, 1-bromo-2,6-dimethylbenzene (4.6 mL, 34.6 mmol) was dissolved in dry THF (30 mL), purged with argon for 5 min and cooled to −78 °C, and *tert*-butyl lithium (41 mL, c = 1.7 mol/L, 70 mmol) was added slowly. The solution was stirred for 15 min at −78 °C, warmed to room temperature and stirred for 2 h. The previously prepared dimesitylfluoroborane was dissolved in dry THF (15 mL) and added slowly to the solution of 2,6-xylyl lithium. The reaction was stirred at room temperature for 3 d and then water (40 mL) was added. The aqueous phase was extracted with hexane (3 x 40 mL). The combined organic phases were dried over magnesium sulfate and the solvent was removed *in vacuo*. The crude product was recrystallized from hexane two times yielding the product as clear colorless crystals (26.3 mmol, 9.33 g, 76%). For photophysical measurements, a small quantity was additionally purified *via* filtration through a silica gel plug using hexane.

^{1}H NMR (500 MHz, CD_2Cl_2) δ = 7.14 (dd, J = 7.5 Hz, 1 H, CH), 6.92 (d, J = 7.5 Hz, 2 H, CH), 6.78 (s, 4 H, CH), 2.28 (s, 6 H, CH_3), 2.02 (s, 6 H, CH_3), 2.00 (s, 6 H, CH_3), 1.98 (s, 6 H, CH_3) ppm.

^{11}B{^{1}H} NMR (160 MHz, CD_2Cl_2) δ = 77 (br) ppm.

^{13}C{^{1}H} NMR (125 MHz, CD_2Cl_2) δ = 147.8, 144.4, 141.0, 140.9, 140.6, 139.8, 129.6, 129.0, 129.0, 128.0, 23.0, 23.0, 22.9, 21.4 ppm.

HRMS (EI$^-$) *m/z*: $[M+H]^+$ found: 355.2587; calc. for $[C_{26}H_{31}B]$: 355.2592 (|Δ| = 1.4 ppm).

Elem. Anal. Calc. (%) for $C_{26}H_{31}B$: C 88.13, H 8.82; found: C 88.12, H 8.91.

Dimesityl-4-(4,4,5,5-tetramethyl-1,3,2-dioxaborolan-2-yl)-2,6-dimethylphenylborane 4b

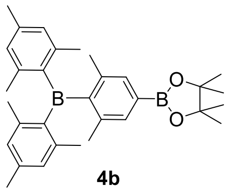

4b

Dimesityl-2,6-dimethylphenylborane **4a** (500 mg, 1.41 mmol), B_2pin_2 (431 mg, 1.70 mmol), dtbpy (8 mg, 29.4 µmol) and [Ir(COD)(µ-OMe)]$_2$ (10 mg, 14.8 µmol) were dissolved in dry, degassed THF (17 mL) and stirred at 80 °C for 23 h. After cooling to room temperature and removing the solvent, the resulting brown oil was purified by automated flash column chromatography (silica gel, hexane/CH_2Cl_2 1:1) yielding the product (612 mg, 1.27 mmol, 90%) as a colorless solid. The ^{1}H NMR spectrum matches that reported in the literature.[26]

^{1}H NMR (200 MHz, CD_2Cl_2) δ = 7.29 (s, 2 H, C*H*), 6.75 (s, 4 H, C*H*), 2.26 (s, 6 H, C*H*$_3$), 2.01 (s, 6 H, C*H*$_3$), 1.97 (s, 6 H, C*H*$_3$), 1.93 (s, 6 H, C*H*$_3$), 1.32 (s, 12 H, C*H*$_3$) ppm.

5-Bromo-2,2'-bithiophene 2

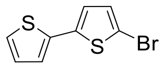

2

Similarly to literature procedures,[27-28] 2,2'-bithiophene (4.14 g, 24.9 mmol) was dissolved in DMF (140 mL) and cooled to 0 °C, and then NBS (4.33 g, 24.3 mmol) was added portion wise. After stirring for 15 min at 0 °C, the reaction mixture was allowed to warm to room temperature while stirring for 1 h. After addition of water (100 mL), the aqueous phase was extracted with $CHCl_3$ (2 x 80 mL). The combined organic phases were washed with water (1 x 80 mL), dried over magnesium sulfate and the solvent was removed *in vacuo*. The resulting green solid was further purified *via* a short way distillation yielding the product (3.62 g, 14.7 mmol, 59%) as a green solid. The ^{1}H NMR spectrum matches that reported in the literature.[29]

^{1}H NMR (300 MHz, CD_2Cl_2) δ = 7.26 (dd, J = 1 Hz, J = 5 Hz, 1 H, C*H*), 7.14 (ddd, J = 0.2 Hz, J = 1 Hz, J = 3.5 Hz, 1 H, C*H*), 7.02 (dd, J = 3.5 Hz, J = 5 Hz, 1 H, C*H*), 7.00 (d, J = 4 Hz, 1 H, C*H*), 6.94 (d, J = 4 Hz, 1 H, C*H*) ppm.

5-[4-(3,5-dimethylphenyl)-dimesitylboryl]-2,2'-bithiophene 7a

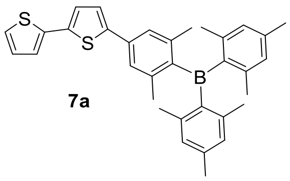

7a

5-Bromo-2,2'-bithiophene **2** (200 mg, 814 µmol), dimesityl-4-(4,4,5,5-tetramethyl-1,3,2-dioxaborolan-2-yl)-2,6-dimethylphenylborane **4b** (430 mg, 895 µmol) and KOH (275 mg, 4.89 mmol) were dissolved in toluene (6 mL) and water (3 mL). After purging the solution with argon for 4 min, $Pd_2(dba)_3 \cdot CHCl_3$ (37.5 mg, 40.8 µmol) and SPhos (70.0 mg, 171 µmol) were added, and the mixture was stirred at 85 °C for 22 h. After cooling to room temperature, the phases were separated, and the aqueous phase was extracted with hexane (4 x 20 mL). The combined organic phases were dried over magnesium sulfate. After removing the solvent *in vacuo*, the crude product was purified *via* flash column chromatography (silica gel, 0–100% ethyl acetate in hexane) yielding the product (298 mg, 575 µmol, 71%) as a yellow solid.

^{1}H NMR (500 MHz, CD_2Cl_2) δ = 7.30 (d, J = 4 Hz, 1 H, C*H*), 7.25 (dd, J = 1.0 Hz, J = 5 Hz, 1 H, C*H*), 7.23 (dd, J = 1 Hz, J = 4 Hz, 1 H, C*H*), 7.22 (s, 2 H, C*H*), 7.17 (d, J = 4 Hz, 1 H, C*H*), 7.05 (dd, J = 4 Hz, J = 5 Hz, 1 H, C*H*), 6.79 (2 br s, 2 H, C*H*), 2.29 (s, 6 H, CH_3), 2.06 (s, 6 H, CH_3), 2.03 (s, 6 H, CH_3), 2.01 (s, 6 H, CH_3) ppm.

^{11}B{^{1}H} NMR (160 MHz, CD_2Cl_2) δ = 78 (br) ppm.

^{13}C{^{1}H} NMR (125 MHz, CD_2Cl_2) δ = 147.6, 1443, 143.6, 141.7, 141.0, 140.9, 139.9, 137.8, 136.8, 129.0, 128.3, 125.0, 124.9, 124.8, 124.1, 124.0, 23.1, 23.1, 23.0, 21.4 ppm.

HRMS (EI^-) *m/z*: [M^-] 518.2281; calc. for [$C_{34}H_{35}BS_2$] 518.2279 (|Δ| = 0.4 ppm).

Elem. Anal. Calc. (%) for $C_{34}H_{35}BS_2$: C 78.75, H 6.80, S 12.36; found: C 78.68, H 6.87, S 12.40.

5’-Bromo-5-[4-(3,5-dimethylphenyl)-dimesitylboryl]-2,2’-bithiophene 7b

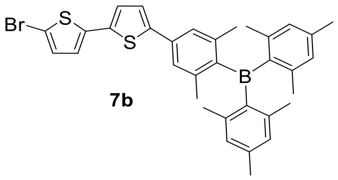

7b

Under ambient conditions, 5-[4-(3,5-dimethylphenyl)-dimesitylboryl]-2,2’-bithiophene **7a** (200 mg, 386 µmol) was dissolved in DMF (5.0 mL) and cooled to 0 °C. After portion wise addition of NBS (69 mg, 386 µmol), the reaction mixture was stirred at 0 °C for 15 min. After warming to room temperature, the solution was stirred for an additional 3 h. As GC-MS showed consumption of NBS, water (10 mL) was added. The resulting yellow precipitate was collected by filtration and washed with water (25 mL). Subsequently, the solid was dissolved in CH_2Cl_2, washed with water, the organic phase was separated and dried over magnesium sulfate. Removal of the solvent *in vacuo* yielded the product (214 mg, 358 µmol, 93%) as a yellow solid.

^{1}H NMR (500 MHz, CD_2Cl_2) δ = 7.28 (d, J = 4 Hz, 1 H, C*H*), 7.20 (s, 2 H, C*H*), 7.11 (d, J = 4 Hz, 1 H, C*H*), 7.01 (d, J = 4 Hz, 1 H, C*H*), 6.97 (d, J = 4 Hz, 1 H, C*H*), 6.78 (s, 4 H, C*H*), 2.28 (s, 6 H, CH_3), 2.05 (s, 6 H, CH_3), 2.02 (s, 6 H, CH_3), 2.00 (s, 6 H, CH_3) ppm.

^{11}B{^{1}H} NMR (160 MHz, CD_2Cl_2) δ = 77 (br) ppm.

^{13}C{^{1}H} NMR (125 MHz, CD_2Cl_2) δ = 147.8, 144.2, 144.2, 141.7, 141.1, 140.9, 139.9, 139.5, 135.6, 134.5, 131.3, 129.0, 125.3, 124.9, 124.1, 111.1, 23.1, 23.0, 23.0, 21.4 ppm.

HRMS (EI^-) *m/z*: [M^- -H^+] 595.1316; calc. for [$C_{34}H_{33}BBrS_2$] 595.1306 (|Δ| = 1.7 ppm).

Elem. Anal. Calc. (%) for $C_{34}H_{34}BBrS_2$: C 68.35, H 5.74, S 10.73; found: C 68.00, H 5.92, S 10.47.

5-[4-(2,6-Dimethylphenyl)-(4-(*N*,*N*-dimethylamino)-2,6-dimethylphenyl)-mesitylboryl]-2,2'-bithiophene 8a

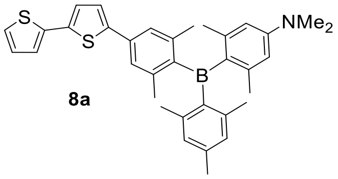

8a

The compound 5-bromo-2,2'-bithiophene **2** (700 mg, 2.86 mmol), [(*N*,*N*-dimethylamino)-2,6-dimethylphenyl]-(mesityl)-[4-(4,4,5,5-tetramethyl-1,3,2-dioxaborolan-2-yl)-2,6-dimethylphenyl]borane **5b** (1.60 g, 3.14 mmol) and KOH (978 mg, 17.4 mmol) were dissolved in toluene (24 mL) and water (12 mL). After purging the solution with argon for 5 min, $Pd_2dba_3 \cdot CHCl_3$ (138 mg, 151 µmol) and SPhos (240 mg, 585 µmol) were added in one portion. The reaction mixture was stirred at 85 °C for 2 d. After cooling to room temperature, the phases were separated, and the aqueous phase was extracted with hexane (3 x 25 mL). The combined organic phases were dried over magnesium sulfate and the solvent was removed *in vacuo*. The resulting crude product was purified *via* preparative column chromatography (silica gel, 10% ethyl acetate in hexane). The product was isolated as a yellow foam (1.46 g, 2.67 mmol, 93%). A portion of this foam was further purified by preparative thin-layer chromatography (PSC plates (silica gel, 2 mm), 5% ethyl acetate in hexane) to yield pure product as a yellow solid.

^{1}H NMR (500 MHz, CD_2Cl_2) δ = 7.29 (m, 1 H, C*H*), 7.25 (m, 1 H, C*H*), 7.22 (m, 1 H, C*H*), 7.20 (s, 2 H, C*H*), 7.16 (m, 1 H, C*H*), 7.04 (m, 1 H, C*H*), 6.76 (s, 2 H, C*H*), 6.32 (s, 2 H, C*H*), 2.97 (s, 6 H, CH_3) 2.27 (s, 3 H, CH_3), 2.15 – 1.95 (m, 18 H, CH_3) ppm.

^{11}B{^{1}H} NMR (160 MHz, CD_2Cl_2) δ = 75 (br) ppm.

^{13}C{^{1}H} NMR (125 MHz, CD_2Cl_2) δ = 152.1, 148.8, 145.3, 143.9, 143.7, 143.6, 139.1, 137.9, 136.5, 135.1, 134.1, 128.8, 128.3, 125.0, 124.7, 124.7, 123.9, 123.8, 111.9, 111.8, 40.1, 24.1, 24.0, 21.3 ppm.

HRMS (EI^-) *m/z*: $[M+H]^+$ found: 548.2604; calc. for $[C_{35}H_{39}BNS_2]$: 548.2611 (|Δ| = 1.3 ppm).

Elem. Anal. Calc. (%) for $C_{35}H_{38}BNS_2$: C 76.76, H 6.99, N 2.56, S 11.71; found: C 77.10, H 7.03, N 2.74, S 11.32.

5-Iodo-5'-[4-(2,6-dimethylphenyl)-(4-(*N*,*N*-dimethylamino)-2,6-dimethylphenyl)-mesitylboryl]-2,2'-bithiophene 8b

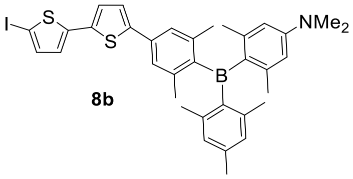

8b

At −78 °C, *n*-butyl lithium (0.35 mL, c = 2.5 M, 880 µmol) was added dropwise to a solution of 5-[4-(2,6-dimethylphenyl)-(4-(*N*,*N*-dimethylamino)-2,6-dimethylphenyl)-mesitylboryl]-2,2'-bithiophene **8a** (399 mg, 730 µmol) in dry, degassed THF (3 mL). After stirring at −78 °C for 1.5 h, iodine (388 mg, 1.53 mmol) was added in one portion and the solution was allowed to warm to room temperature and then stirred for 18 h. The reaction was quenched by addition of water (5 mL) and diethyl ether (5 mL). The aqueous phase was extracted with diethyl ether (3 x 3 mL). The combined organic phase was washed with an aqueous, saturated sodium thiosulfate solution (1 x 10 mL), separated, and dried over magnesium sulfate and the solvent was removed *in vacuo* yielding a mixture of starting material and product (438 mg, purity ≈80%) which was used without further purification for further reaction.

^{1}H NMR (500 MHz, CD_2Cl_2) δ = 7.27 (d, J = 4 Hz, 1 H, C*H*), 7.20 – 7.17 (m, 3 H, C*H*), 7.11 (d, J = 4 Hz, 1 H, C*H*), 6.89 (d, J = 4 Hz, 1 H, C*H*), 6.75 (s, 2 H, C*H*), 6.32 (s, 2 H, C*H*), 2.96 (s, 6 H, CH_3), 2.27 (s, 3 H, CH_3), 2.13 – 1.94 (m, 18 H, CH_3) ppm.

HRMS (EI$^-$) *m/z*: $[M+H]^+$ found: 674.1565; calc. for $[C_{35}H_{38}BINS_2]$: 674.1578 (|Δ| = 1.9 ppm).

5-[4-*Bis*-[4-(*N*,*N*-dimethylamino)-2,6-dimethylphenyl]-3,5-dimethylphenylboryl]-2,2'-bithiophene 9a

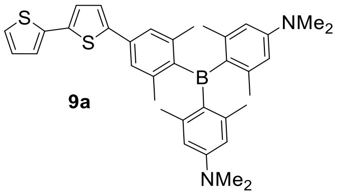

9a

The compounds 5-bromo-2,2'-bithiophene **2** (526 mg, 2.04 mmol), *bis*-[4-(*N*,*N*-dimethylamino)-2,6-dimethylphenyl]-2,6-dimethyl-4-(4,4,5,5-tetramethyl-1,3,2-dioxaborolan-2-yl)phenylborane **6b** (1.21 g, 2.24 mmol) and KOH (638 mg, 11.4 mmol) were dissolved in a mixture of toluene (20 mL) and water (10 mL). After purging the solution with argon for 5 min, SPhos (185 mg, 451 µmol) and $Pd_2(dba)_3{\cdot}CHCl_3$ (95 mg, 104 µmol) were added in one portion. The reaction mixture was stirred at 85 °C for 41 h. After cooling to room temperature, the phases were separated, and the aqueous phase was extracted with hexane (5 x 15 mL). The combined organic phase was dried over magnesium sulfate and the solvent was removed *in vacuo*. The resulting crude product was purified *via* flash column chromatography (silica gel, 0–10% ethyl acetate in hexane) yielding the product (937 mg, 1.62 mmol, 80%) as a yellow solid.

^{1}H NMR (500 MHz, CD_2Cl_2) δ = 7.29 (d, *J* = 4 Hz, 1 H, C*H*), 7.24 (dd, *J* = 1 Hz, *J* = 5 Hz, 1 H, CH), 7.22 (dd, *J* = 1 Hz, *J* = 4 Hz, 1 H, C*H*), 7.20 (s, 2 H, C*H*), 7.17 (d, *J* = 4 Hz, 1 H, C*H*), 7.05 (dd, *J* = 4 Hz, *J* = 5 Hz, 1 H, C*H*), 6.34 (s, 4 H, C*H*), 6.33 (s, 4 H, C*H*), 2.96 (s, 12 H, CH_3), 2.09 (s, 6 H, CH_3), 2.04 (s, 6 H, CH_3), 1.99 (s, 6 H, CH_3) ppm.

^{11}B{^{1}H} NMR (160 MHz, CD_2Cl_2) δ = 74 (br) ppm.

^{13}C{^{1}H} NMR (125 MHz, CD_2Cl_2) δ = 151.7, 149.8, 144.1, 143.3, 142.9, 141.4, 138.0, 136.4, 136.1, 133.8, 128.3, 125.0, 124.7, 124.6, 123.9, 123.6, 111.9, 111.8, 40.2, 21.0, 23.9, 23.0 ppm.

HRMS (EI^+) m/z: $[M+H]^+$ 577.2874; calc. for $[C_{36}H_{42}BN_2S_2]$ 577.2877 (|Δ| = 0.5 ppm).

Elem. Anal. Calc. (%) for $C_{36}H_{41}BN_2S_2$: C 74.98, H 7.17, N 4.86, S 11.12; found: C 74.98, H 7.53, N 5.03, S 10.77.

5’-Iodo-5-[4-*bis*-[4-(*N*,*N*-dimethylamino)-2,6-dimethylphenyl]-3,5-dimethylphenylboryl]-2,2’-bithiophene 9b

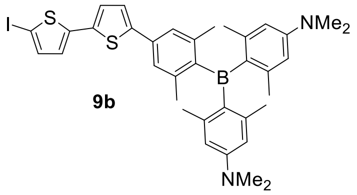

9b

At −78 °C, *n*-butyl lithium (0.35 mL, c = 2.5 M, 880 µmol) was added dropwise to a solution of 5-[4-(4-*bis*-[4-(*N*,*N*-dimethylamino)-2,6-dimethylphenyl]-3,5-dimethylphenylboryl]-2,2’-bithiophene **9a** (400 mg, 690 µmol) in dry, degassed THF (3 mL). After stirring at −78 °C for 1.5 h, iodine (359 mg, 1.41 mmol) was added in one portion and the solution was stirred at room temperature for 18 h. The reaction was quenched by addition of water (5 mL) and diethyl ether (5 mL). The aqueous phase was extracted with diethyl ether (3 x 3 mL). The combined organic phase was washed with an aqueous, saturated sodium thiosulfate solution (10 mL), dried over magnesium sulfate and the solvent was removed *in vacuo* yielding a mixture of starting material and product (460 mg, purity ≈80%) which was used without further purification for further reaction.

^{1}H NMR (500 MHz, CD_2Cl_2) δ = 7.26 (d, *J* = 4 Hz, 1 H, C*H*), 7.20 – 7.17 (m, 3 H, C*H*), 7.11 (d, *J* = 4 Hz, 1 H, C*H*), 6.89 (d, *J* = 4 Hz, 1 H, C*H*), 6.32 (s, 4 H, C*H*), 2.95 (s, 12 H, CH_3), 2.09 – 1.94 (m, 18 H, CH_3) ppm.

HRMS (EI^-) *m/z*: $[M+H]^+$ found: 703.1836; calc. for $[C_{36}H_{41}BIN_2S_2]$: 703.1843 (|Δ| = 1.0 ppm).

5,5'-*Bis*-[4-(2,6-dimethylphenyl)-dimesitylboryl]-2,2'-bithiophene Neut0

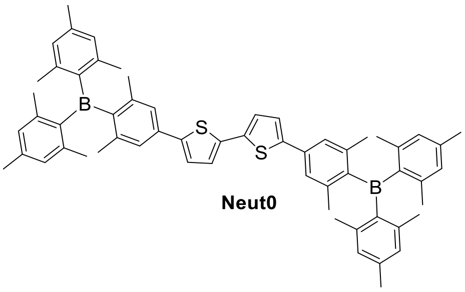

Neut0

The compounds 5,5'-dibromo-2,2'-bithiophene **2** (337 mg, 1.04 mmol), dimesityl-4-(4,4,5,5-tetramethyl-1,3,2-dioxaborolan-2-yl)-2,6-dimethylphenylborane **4b** (500 mg, 1.04 mmol), and KOH (350 mg, 6.24 mmol) were dissolved in toluene (15 mL) and water (5 mL). After purging the solution with argon for 10 min, $Pd_2dba_3 \cdot CHCl_3$ (67 mg, 73.2 µmol) and SPhos (85 mg, 207 µmol) were added in one portion. The reaction mixture was stirred at 85 °C for 36 h. After cooling to room temperature, the phases were separated, and the aqueous phase was extracted with hexane (5 x 20 mL). The combined organic phase was washed with brine (3 x 30 mL), dried over magnesium sulfate and the solvent was removed *in vacuo*. The resulting crude product was purified *via* column chromatography (silica gel, 4% ethyl acetate in hexane). The resulting solid was further purified by precipitation from a saturated diethyl ether solution with methanol, yielding the product as a yellow solid (180 mg, 200 µmol, 31%). Prior to photophysical measurements, a small quantity was further purified by column chromatography (silica gel, 0-8% ethyl acetate in hexane) and by precipitation from a saturated diethyl ether solution with methanol.

^{1}H NMR (500 MHz, CD_2Cl_2): δ = 7.31 (d, J = 4 Hz, 2 H, CH), 7.21 – 7.20 (m, 4 H, CH), 7.19 (d, J = 4 Hz, 2 H, CH), 6.77 (s, 4 H, CH), 6.77 (s, 4 H, CH), 2.27 (s, 12 H, CH_3), 2.05 (s, 12 H, CH_3), 2.01 (s, 12 H, CH_3), 1.99 (s, 12 H, CH_3) ppm.

^{13}C{^{1}H} NMR (125 MHz, CD_2Cl_2): δ = 143.9, 143.2, 141.3, 140.6, 140.5, 136.5, 134.3, 128.6, 124.5, 124.4, 123.7, 22.6, 22.6, 22.5, 20.9 ppm.

It was not possible to obtain a **^{11}B{^{1}H} NMR** spectrum (160 MHz) due to the low solubility of **Neut0** in CD_2Cl_2 and acetone-d_6.

HRMS (EI^-) *m/z*: $[M+H]^+$ found: 871.4701; calc. for $[C_{60}H_{65}B_2NS_2]$: 871.4708 (|Δ| = 0.8 ppm).

Elem. Anal. Calc. (%) for $C_{60}H_{64}B_2NS_2$: C 82.75, H 7.41, N 1.56, S 7.36; found: C 81.75, H 7.49, N 1.39, S 7.07.

5’-[4-(2,6-Dimethylphenyl)-(4-(*N*,*N*-dimethylamino)-2,6-dimethylphenyl)-mesitylboryl]-5-[4-(2,6-dimethylphenyl)-dimesitylboryl]-2,2’-bithiophene Neut1

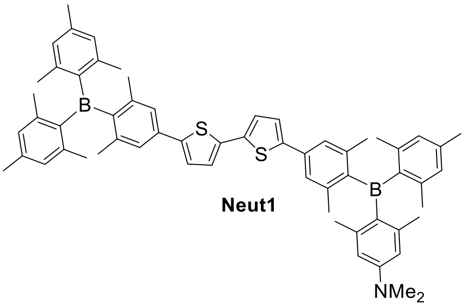

The compounds 5’-bromo-5-[4-(3,5-dimethylphenyl)-dimesitylboryl]-2,2’-bithiophene **7b** (276 mg, 462 µmol), [4-(*N*,*N*-dimethylamino)-2,6-dimethylphenyl]-(mesityl)-[4-(4,4,5,5-tetramethyl-1,3,2-dioxaborolan-2-yl)2,6-dimethylphenyl]borane **5b** (258 mg, 506 µmol) and KOH (167 mg, 2.98 mmol) were dissolved in toluene (4 mL) and water (2 mL). After purging the solution with argon for 10 min, $Pd_2dba_3{\cdot}CHCl_3$ (22 mg, 24.0 µmol) and SPhos (40 mg, 97.4 µmol) were added in one portion. The reaction mixture was stirred at 85 °C for 2 d. After cooling to room temperature, the phases were separated, and the aqueous phase was extracted with hexane (5 x 20 mL). The combined organic phase was washed with brine (3 x 30 mL), dried over magnesium sulfate and the solvent was removed *in vacuo*. The resulting crude product was purified *via* preparative thin layer chromatography (PSC plates (silica gel, 2 mm), 3% ethyl acetate in hexane). The resulting solid was further purified by precipitation from a saturated diethyl ether solution with methanol, yielding the product as an orange yellow solid (180 mg, 200 µmol, 43%).

^{1}H NMR (500 MHz, CD_2Cl_2) δ = 7.31 (d, J = 4 Hz, 1 H, CH), 7.30 (d, J = 4 Hz, 1 H, CH), 7.23 (s, 2 H, CH), 7.21 (br s, 2 H, CH), 7.19 (d, J = 4 Hz, 2 H, CH), 6.79 (s, 4 H, CH), 6.77 (br s, 2 H, CH), 6.33 (s, 2 H, CH), 2.97 (s, 6 H, CH_3), 2.28 (s, 6 H, CH_3), 2.28 (s, 3 H, CH_3), 2.07 – 1.97 (m, 36 H, CH_3) ppm.

^{11}B{^{1}H} NMR (160 MHz, CD_2Cl_2) δ = 78 (br) ppm.

^{13}C{^{1}H} NMR (125 MHz, CD_2Cl_2) δ = 152.1, 148.9, 147.6, 145.3, 144.3, 143.9, 143.8, 143.6, 143.5, 141.7, 141.0, 140.9, 139.9, 139.1, 137.0, 136.7, 135.1, 134.7, 134.2, 129.0, 128.8, 124.9, 124.8, 124.7, 124.2, 123.9, 111.9, 111.9, 40.1, 24.1, 24.0, 23.1, 23.1, 23.0, 21.4, 21.3 ppm.

HRMS (EI$^-$) *m/z*: [M+H]$^+$ found: 900.4960; calc. for [$C_{61}H_{67}B_2NS_2$]: 900.4974 (|Δ| = 1.6 ppm).

Elem. Anal. Calc. (%) for $C_{61}H_{67}B_2NS_2$: C 81.41, H 7.50, N 1.56, S 7.12; found: C 80.87, H 7.51, N 1.39, S 7.26.

5'-[4-(2,6-Dimethylphenyl)-*bis*-(4-(*N*,*N*-dimethylamino)-2,6-dimethylphenyl)-boryl]-5-[4-(2,6-dimethylphenyl)-dimesitylboryl]-2,2'-bithiophene Neut2

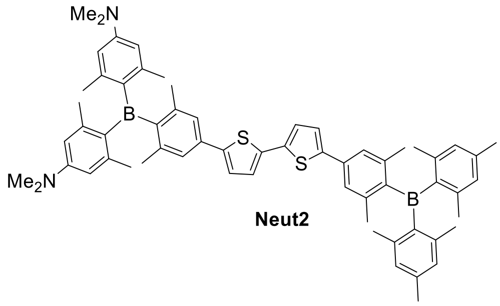

The compounds 5'-bromo-5-[4-(3,5-dimethylphenyl)-dimesitylboryl]-2,2'-bithiophene **7b** (350 mg, 586 µmol), *bis*-[4-(*N*,*N*-dimethylamino)-2,6-dimethylphenyl]-2,6-dimethyl-4-(4,4,5,5-tetramethyl-1,3,2dioxaborolan-2-yl)-phenylborane **6b** (353 mg, 644 µmol) and KOH (205 mg, 3.65 mmol) were dissolved in toluene (14 mL) and water (7 mL). After purging the solution with argon for 10 min, $Pd_2(dba)_3{\cdot}CHCl_3$ (27 mg, 29.5 µmol) and SPhos (53 mg, 129 µmol) were added. The resulting solution was stirred at 85 °C for 42 h. After cooling to room temperature, the phases were separated, and the aqueous phase was extracted with hexane (4 x 20 mL). The combined organic phase was dried over magnesium sulfate. After removing the solvent *in vacuo*, the crude product was further purified *via* flash column chromatography (silica gel, 0–100% ethyl acetate in hexane). The resulting orange oil was further purified by precipitation from a saturated diethyl ether solution with methanol, yielding the product (320 mg, 344 µmol, 59%) as a yellow fine powder.

^{1}H NMR (500 MHz, CD_2Cl_2) δ = 7.31 (d, J = 4 Hz, 1 H, C*H*), 7.30 (d, J = 4 Hz, 1 H, C*H*), 7.23 (br s, 2 H, C*H*), 7.20 (br s, 2 H, C*H*), 7.19 (d, J = 4 Hz, 2 H, C*H*), 6.79 (2 br s, 2 H, C*H*), 6.34 (br s, 2 H, C*H*), 6.33 (br s, 2 H, C*H*), 2.96 (s, 12 H, CH_3), 2.29 (s, 6 H, CH_3), 2.09 (s, 6 H, CH_3), 2.07 (s, 6 H, CH_3), 2.03 (br s, 12 H, CH_3), 2.01 (s, 6 H, CH_3), 2.00 (br s, 6 H, CH_3) ppm.

^{11}B{^{1}H} NMR (160 MHz, CD_2Cl_2) δ = 77 (br) ppm.

^{13}C{^{1}H} NMR (125 MHz, CD_2Cl_2) δ = 151.7, 149.9, 147.6, 144.3, 144.2, 143.4, 143.3, 142.9, 141.7, 141.4, 141.0, 140.9, 139.9, 137.1, 136.5, 136.1, 134.8, 133.7, 129.0, 124.9, 124.8, 124.6, 124.2, 123.8, 111.9, 111.8, 40.2, 24.1, 23.9, 23.1, 23.1, 23.1, 23.0, 21.4 ppm.

HRMS (EI^-) *m/z*: $[M+H]^+$ 929.5224; calc. for $[C_{62}H_{71}B_2N_2S_2]$ 929.5239 (|Δ| = 1.6 ppm).

Elem. Anal. Calc. (%) for $C_{62}H_{70}B_2N_2S_2$: C 80.16, H 7.60, N 3.02, S 6.90; found: C 80.36, H 7.64, N 3.31, S 6.55.

5,5'-*Bis*-[4-(2,6-Dimethlyphenyl)-(4-(*N*,*N*-dimethylamino)-2,6-dimethylphenyl)-mesitylboryl]-2,2'-bithiophene Neut(i)2

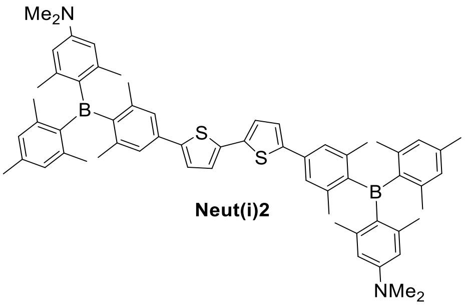

Neut(i)2

The compounds 5,5'-dibromo-2,2'-bithiophene **2** (164 mg, 505 µmol), [(*N*,*N*-dimethylamino)-2,6-dimethylphenyl]-(mesityl)-[4-(4,4,5,5-tetramethyl-1,3,2-dioxaborolan-2-yl)-2,6-dimethylphenyl]borane **5b** (566 mg, 1.11 mmol) and KOH (195 mg, 3.48 mmol) were dissolved in toluene (4 mL) and water (2 mL). After purging the solution with argon for 5 min, $Pd_2dba_3 \cdot CHCl_3$ (23 mg, 25 µmol) and SPhos (43 mg, 105 µmol) were added in one portion. The reaction mixture was stirred at 85 °C for 44 h. After cooling to room temperature, the phases were separated, and the aqueous phase was extracted with hexane (6 x 15 mL). The combined organic phase was dried over magnesium sulfate and the solvent was removed *in vacuo*. The resulting crude product was purified *via* preparative column chromatography (Alox 90, basic, 5% ethyl acetate in hexane). The resulting solid was further purified by precipitation from a saturated diethyl ether solution with methanol, yielding the product as a yellow-orange solid (250 mg, 269 µmol, 53%).

^{1}H NMR (500 MHz, CD_2Cl_2) δ = 7.30 (d, J = 4 Hz, 2 H, C*H*), 7.20 (br s, 4 H, C*H*), 7.18 (d, J = 4 Hz, 2 H, C*H*), 6.76 (br s, 4 H, C*H*), 6.32 (br s, 4 H, C*H*), 2.97 (s, 12 H, CH_3), 2.27 (s, 6 H, CH_3), 2.12 – 1.95 (m, 36 H, CH_3) ppm.

^{11}B{^{1}H} NMR (160 MHz, CD_2Cl_2) δ = 75 (br) ppm.

^{13}C{^{1}H} NMR (125 MHz, CD_2Cl_2) δ = 152.1, 148.8, 145.3, 143.8, 143.7, 143.6, 139.1, 136.7, 135.1, 134.1, 128.8, 124.8, 124.7, 123.9, 111.9, 111.8, 40.1, 24.1, 24.0, 23.2, 23.1, 22.9, 21.3 ppm.

HRMS (EI$^-$) *m/z*: $[M+H]^+$ found: 929.5219; calc. for $[C_{62}H_{70}B_2N_2S_2]$: 929.5239 (|Δ| = 2.2 ppm).

Elem. Anal. Calc. (%) for $C_{62}H_{70}B_2N_2S_2$: C 80.16, H 7.60, N 3.02, S 6.90; found: C 79.62, H 7.85, N 2.90, S 7.48.

5’-[4-(2,6-Dimethylphenyl)-(4-(*N*,*N*-dimethylamino)-2,6-dimethylphenyl)-mesitylboryl]-5-[4-(2,6-dimethylphenyl)-*bis*-(4-(*N*,*N*-dimethylamino)-2,6-dimethylphenyl)boryl]-2,2’-bithiophene Neut3

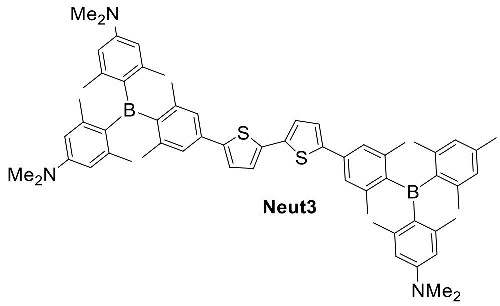

Neut3

Entry A: 5’-Iodo-5-[4-(3,5-dimethylphenyl)-*bis*-(4-(*N*,*N*-dimethylamino)-2,6-dimethylphenyl)-boryl]-2,2’-bithiophene **9b** (460 mg, 655 µmol, purity ≈80%), [4-(*N*,*N*-dimethylamino)-2,6-dimethylphenyl]-(mesityl)-[4-(4,4,5,5-tetramethyl-1,3,2-dioxaborolan-2-yl)-2,6-dimethylphenyl]borane **5b** (367 mg, 720 µmol) and KOH (285 mg, 5.08 mmol) were dissolved in toluene (6 mL) and water (3 mL). After purging the solution with argon for 4 min, $Pd_2dba_3 \cdot CHCl_3$ (30 mg, 33 µmol) and SPhos (60 mg, 146 µmol) were added in one portion. The reaction mixture was stirred at 85 °C for 44 h. After cooling to room temperature, the aqueous phase was extracted with hexane (3 x 20 mL). The combined organic phase was dried over magnesium sulfate and the solvent was removed *in vacuo*. The resulting crude product was purified *via* column chromatography (silica gel, 10% ethyl acetate in hexane). The resulting solid was further purified by precipitation from a saturated diethyl ether solution with methanol, yielding the product as a yellow-orange solid (31 µmol, 30 mg, 6%).

Entry B: 5-Iodo-5’-[4-(2,6-dimethylphenyl)-(4-(*N*,*N*-dimethylamino)-2,6-dimethylphenyl)-mesitylboryl]-2,2’-bithiophene **8b** (438 mg, 650 µmol, purity ≈80%), [*bis*-(4-(*N*,*N*-dimethylamino)-2,6-dimethylphenyl)]-[4-(4,4,5,5-tetramethyl-1,3,2-dioxaborolan-2-yl)-2,6-dimethylphenyl]borane **6b** (385 mg, 715 µmol) and KOH (295 mg, 5.26 mmol) were dissolved in toluene (6 mL) and water (3 mL). After purging the solution with argon for 4 min, $Pd_2dba_3 \cdot CHCl_3$ (30 mg, 32.8 µmol) and SPhos (60 mg, 146 µmol) were added in one portion. The reaction mixture was stirred at 85 °C for 44 h. After cooling to room temperature, the phases were separated, and the aqueous phase was extracted with hexane (3 x 20 mL). The combined organic phase was dried over magnesium sulfate and the solvent was removed *in vacuo*. The resulting crude product was purified *via* column chromatography (silica gel, 10% ethyl acetate in hexane). The resulting solid was further purified by precipitation from a saturated diethyl ether solution with methanol, yielding the product as a yellow-orange solid (300 mg, 120 µmol, 60%).

^{1}H NMR (500 MHz, CD_2Cl_2) δ = 7.31 – 7.28 (m, 2 H, C*H*), 7.21 – 7.17 (m, 6 H, C*H*), 6.76 (s, 2 H, C*H*), 6.32 (s, 6 H, C*H*), 2.97 (s, 6 H, CH_3), 2.95 (s, 12 H, CH_3), 2.27 (s, 3 H, CH_3), 2.08 – 1.95 (m, 36 H, CH_3) ppm.

^{11}B{^{1}H} NMR (160 MHz, CD_2Cl_2) δ = 75 (br) ppm.

^{13}C{^{1}H} NMR (125 MHz, CD_2Cl_2) δ = 152.1, 151.7, 149.9, 148.8, 145.3, 144.0, 143.7, 143.7, 143.6, 143.3, 142.9, 141.4, 139.1, 136.8, 136.5, 136.1, 135.1, 134.1, 133.7, 128.8, 124.8, 124.7, 124.5, 123.9, 123.7, 111.9, 111.8, 40.2, 40.1, 24.1, 24.0, 24.0, 23.8, 23.0, 21.3 ppm.

HRMS (EI^-) *m/z*: $[M+H]^+$ found: 958.5473; calc. for $[C_{63}H_{74}B_2N_3S_2]$: 958.5505 (|Δ| = 3.3 ppm).

Elem. Anal. Calc. (%) for $C_{63}H_{73}B_2N_3S_2$: C 78.98, H 7.68, N 4.39, S 6.69; found: C 78.69, H 7.70, N 4.33, S 6.68.

5'-[4-(2,6-Dimethylphenyl)-(4-(*N*,*N*,*N*-trimethylammonium)-2,6-dimethylphenyl)-mesitylboryl]-5-[4-(2,6-dimethylphenyl)-dimesitylboryl]-2,2'-bithiophene triflate Cat^{1+}

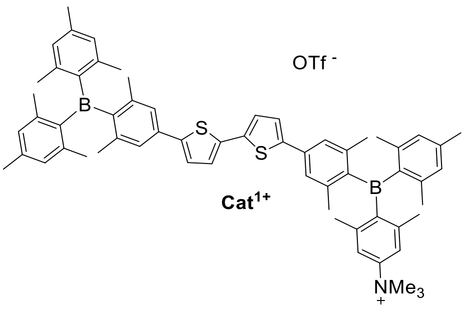

The compound 5'-[4-(2,6-dimethylphenyl)-(4-(*N*,*N*-dimethylamino)-2,6-dimethylphenyl)-mesitylboryl]-5-[4-(2,6-dimethylphenyl)-dimesitylboryl]-2,2'-bithiophene **Neut1** (15 mg, 16.7 µmol) was dissolved in dry, degassed CH_2Cl_2 (2 mL). After addition of methyl triflate (15.0 µL, 133 µmol), the reaction mixture was stirred in the dark at room temperature for 3 d. Subsequently, hexane (7 mL) was added. The resulting solid was collected by filtration and washed with hexane (8 mL) yielding the product as an orange yellow solid (15 mg, 14.8 µmol, 88%).

^{1}H NMR (500 MHz, CD_2Cl_2) δ = 7.51 (s, 2 H, C*H*), 7.41 (d, J = 4 Hz, 1 H, C*H*), 7.37 (d, J = 4 Hz, 1 H, C*H*), 7.30 (s, 1 H, C*H*), 7.27 (s, 1 H, C*H*), 7.25 – 7.23 (m, 2 H, C*H*), 7.23 (s, 2 H, C*H*), 6.85 (s, 1 H, C*H*), 6.82 (s, 2 H, C*H*), 6.78 (s, 4 H, C*H*), 3.65 (s, 9 H, CH_3), 2.29 (s, 3 H, CH_3), 2.27 (s, 6 H, CH_3), 2.20 (s, 3 H, CH_3), 2.17 (s, 3 H, CH_3), 2.10 (s, 3 H, CH_3), 2.07 (s, 3 H, CH_3), 2.05 (s, 6 H, CH_3), 2.03 (s, 3 H, CH_3), 2.01 (s, 6 H, CH_3), 1.98 (s, 6 H, CH_3), 1.96 (s, 3 H, CH_3) ppm.

^{13}C{^{1}H} NMR (125 MHz, CD_2Cl_2) δ = 150.9, 149.1, 148.5, 146.9, 145.1, 144.6, 144.6, 44.5, 144.0, 143.8, 143.2, 142.6, 142.4, 142.3, 142.1, 141.9, 141.7, 141.6, 140.9, 138.3, 137.6, 137.0, 136.0, 130.2, 130.1, 129.8, 129.8, 125.9, 125.8, 125.8, 125.7, 126.6, 125.2, 121.8 (q, $^{1}J_{CF}$ = 316 Hz), 119.6, 57.5, 23.5, 23.4, 23.4, 23.3, 23.2, 23.2, 23.1, 21.3, 23.3 ppm.

HRMS (ESI^{+}) *m/z*: [M−OTf]$^{+}$ found: 914.5115; calc. for [$C_{62}H_{70}B_2NS_2$]: 914.5130 (|Δ| = 1.6 ppm).

Elem. Anal. Calc. (%) for $C_{63}H_{70}B_2F_3NO_3S_3$: C 71.11, H 6.63, N 1.32, S 9.04; found: C 67.93, H 6.63, N 1.28, S 8.78.

5’-[4-(2,6-Dimethylphenyl)-*bis*-(4-(*N*,*N*,*N*-trimethylammonium)-2,6-dimethylphenyl)-boryl]-5-[4-(2,6-dimethylphenyl)-dimesitylboryl]-2,2’-bithiophene *bis*-triflate Cat^{2+}

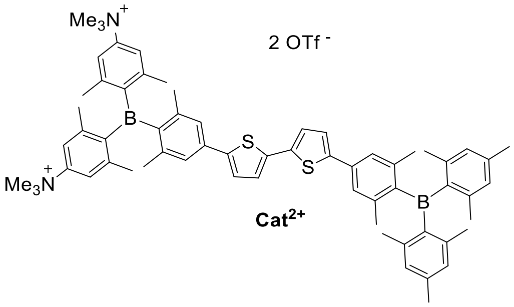

The compound 5’-[4-(2,6-dimethylphenyl)-*bis*-(4-(*N*,*N*-dimethylamino)-2,6-dimethylphenyl)-boryl]-5-[4-(2,6-dimethylphenyl)-dimesitylboryl]-2,2’-bithiophene **Neut2** (15 mg, 16.1 µmol) was dissolved in dry, degassed CH_2Cl_2 (2.0 mL). After addition of methyl triflate (15.0 µL, 132 µmol), the reaction mixture was stirred in the dark at room temperature for 3 d. Subsequently, hexane (7 mL) was added. The resulting solid was collected by filtration and washed with diethyl ether (10 mL) yielding the product (14 mg, 11.1 µmol, 69%) as a yellow orange solid.

^{1}H NMR (500 MHz, CD_3OD) δ = 7.58 (br s, 2 H, C*H*), 7.57 (br s, 2 H, C*H*), 7.46 (d, *J* = 4 Hz, 1 H, C*H*), 7.39 (d, *J* = 4 Hz, 1 H, C*H*), 7.34 (br s, 2 H, C*H*), 7.27 (d, *J* = 4 Hz, 1 H, C*H*), 7.26 (d, *J* = 4 Hz, 1 H, C*H*), 7.24 (br s, 2 H, C*H*), 3.66 (s, 18 H, CH_3), 2.27 (s, 6 H, CH_3), 2.25 (br s, 6 H, CH_3), 2.16 (br s, 6 H, CH_3), 2.08 (s, 6 H, CH_3), 2.05 (s, 6 H, CH_3), 2.02 (s, 6 H, CH_3), 1.98 (s, 6 H, CH_3) ppm.

^{13}C{^{1}H} NMR (125 MHz, CD_3OD) δ = 149.6, 149.4, 148.6, 145.6, 145.1, 144.8, 144.6, 144.5, 143.3, 143.1, 142.5, 141.8, 141.6, 140.6, 138.7, 137.8, 137.5, 135.9, 129.8, 129.8, 126.1, 126.0, 126.0, 125.8, 125.6, 125.2, 121.8 (q, $^1J_{CF}$ = 318 Hz) 120.1, 120.0, 57.5, 23.7, 23.5, 23.4, 23.3, 23.3, 23.2, 21.4 ppm.

HRMS (EI$^+$) *m/z*: [M−2OTf]$^+$ 479.2803; calc. for [$C_{64}H_{76}B_2N_2S_2$] 479.2813 (|Δ| = 2.1 ppm).

Elem. Anal. Calc. (%) for $C_{66}H_{76}B_2F_6N_2O_6S_2$: C 63.06, H 6.09, N 2.23, S 10.20; found: C 60.69, H 6.68, N 1.95, S 10.86.

5,5'-*Bis*-[4-(2,6-Dimethylphenyl)-(4-(*N*,*N*-dimethylammonium)-2,6-dimethylphenyl)-mesitylboryl]-2,2'-bithiophene *bis*-triflate Cat(i)$^{2+}$

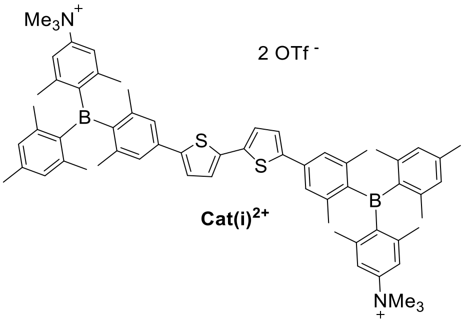

Cat(i)$^{2+}$

The compound 5,5'-*bis*-[4-(2,6-dimethylphenyl)-(4-(*N*,*N*-dimethylamino)-2,6-dimethylphenyl)-mesitylboryl]-2,2'-bithiophene **Neut(i)2** (15 mg, 16.1 µmol) was dissolved in dry CH_2Cl_2 (2 mL). After addition of methyl triflate (15.0 µL, 129 µmol), the reaction mixture was stirred in the dark at room temperature for 17 h. The resulting solid was collected by filtration and washed with diethyl ether (3 x 2 mL) yielding the product as a yellow solid (10 mg, 7.95 µmol, 49%).

^{1}H NMR (500 MHz, CD_3OD) δ = 7.51 (s, 4 H, C*H*), 7.42 (d, *J* = 4 Hz, 2 H, C*H*), 7.30 (m, 4 H, C*H*), 7.26 (d, *J* = 4 Hz, 2 H, C*H*), 6.84 (m, 4 H, C*H*), 3.65 (s, 18 H, C*H₃*), 2.29 (s, 6 H, C*H₃*), 2.20 (s, 6 H, C*H₃*), 2.17 (s, 6 H, C*H₃*), 2.10 (s, 6 H, C*H₃*), 2.07 (s, 6 H, C*H₃*), 2.04 (s, 6 H, C*H₃*), 1.97 (s, 6 H, C*H₃*) ppm.

^{13}C{^{1}H} NMR (125 MHz, CD_3OD) δ = 150.8, 149.1, 147.0, 144.6, 144.5, 144.0, 143.2, 142.5, 142.3, 142.2, 141.7, 138.1, 136.9, 130.2, 130.1, 125.9, 125.9, 125.8, 125.7, 121.8 (q, $^1J_{CF}$ = 318 Hz), 119.6, 57.5, 23.4, 23.4, 23.3, 23.3, 23.2, 21.3 ppm.

HRMS (ESI$^+$) *m/z*: [M−2OTf]$^+$ found: 479.2818; calc. for [$C_{64}H_{76}B_2N_2S_2$]: 479.2813 (|Δ| = 1.0 ppm).

Elem. Anal. Calc. (%) for $C_{66}H_{76}B_2F_6N_2O_6S_4$: C 63.06, H 6.09, N 2.23, S 10.20; found: C 61.50, H 6.13, N 2.12, S 9.74.

5'-[4-(2,6-Dimethylphenyl)-(4-(*N,N,N*-trimethylammonium)-2,6-dimethylphenyl)-mesitylboryl]-5-[4-(2,6-dimethylphenyl)-*bis*-(4-(*N,N,N*-trimethylammonium)-2,6-dimethylphenyl)boryl]-2,2'-bithiophene *tris*-triflate Cat^{3+}

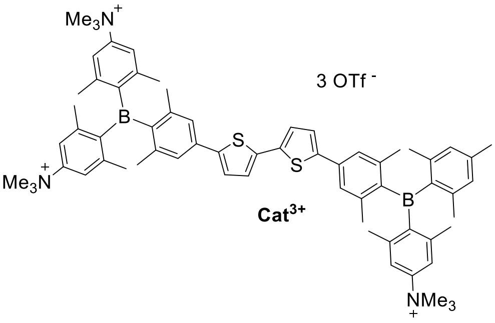

The compound 5'-[4-(2,6-dimethylphenyl)-(4-(*N*,*N*-dimethylamino)-2,6-dimethylphenyl)-mesitylboryl]-5-[4-(2,6-dimethylphenyl)-*bis*-(4-(*N*,*N*-dimethylamino)-2,6-dimethylphenyl)boryl]-2,2'-bithiophene **Neut3** (15 mg, 15.6 µmol) was dissolved in dry CH_2Cl_2 (2 mL). After addition of methyl triflate (21.3 µL, 188 µmol), the reaction mixture was stirred in the dark at room temperature for 21 h. The resulting solid was collected by filtration and washed with diethyl ether (4 x 2 mL) yielding the product as a yellow solid (13 mg, 8.96 µmol, 57%).

^{1}H NMR (500 MHz, CD_3OD) δ = 7.57 (s, 4 H, C*H*), 7.51 (s, 2 H, C*H*), 7.46 (d, *J* = 4 Hz, 1 H, C*H*), 7.42 (d, *J* = 4 Hz, 1 H, C*H*), 7.34 (s, 2 H, C*H*), 7.31 – 7.28 (m, 2 H, C*H*), 7.28 – 7.26 (m, 2 H, C*H*), 6.83 (m, 2 H, C*H*), 3.66 (s, 18 H, CH_3), 3.65 (s, 9 H, CH_3), 2.28 (s, 3 H, CH_3), 2.25 (s, 6 H, CH_3), 2.20 (s, 3 H, CH_3), 2.16 (s, 3 H, CH_3), 2.15 (s, 6 H, CH_3), 2.10 (s, 3 H, CH_3), 2.08 (s, 6 H, CH_3), 2.07 (s, 3 H, CH_3), 2.03 (s, 3 H, CH_3), 1.96 (s, 3 H, CH_3) ppm.

^{13}C{^{1}H} NMR (125 MHz, CD_3OD) δ = 150.8, 149.7, 149.4, 149.1, 147.1, 145.7, 144.9, 144.6, 144.5, 144.4, 144.2, 144.0, 143.5, 143.2, 143.1, 142.5, 142.2, 142.1, 141.8, 138.5, 138.0, 137.8, 136.8, 130.1, 130.1, 126.2, 126.7, 126.0, 125.9, 125.8, 125.7, 121.8 (q, $^{1}J_{CF}$ = 318 Hz), 120.1, 120.1, 119.6, 57.5, 23.6, 23.4, 23.4, 23.4, 23.4, 23.3, 23.2, 21.3 ppm.

HRMS (ESI$^+$) *m/z*: $[M-3OTf]^{3+}$ found: 334.2039; calc. for $[C_{66}H_{82}B_2N_3S_2]$: 334.2040 (|Δ| = 0.30 ppm).

Elem. Anal. Calc. (%) for $C_{69}H_{82}B_2F_9N_3O_9S_5$: C 57.14, H 5.70, N 2.90, S 9.93; found: C 52.95, H 5.69, N 2.81, S 10.95.

[4-(*N*,*N,N*-Trimethylammonium)-2,6-dimethylphenyl]-(mesityl)-2,6-dimethylphenylborane triflate 5c

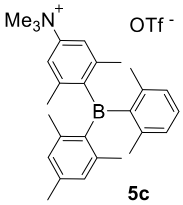

5c

[4-(*N*,*N*-Dimethylamino)-2,6-dimethylphenyl]-(mesityl)-2,6-dimethylphenylborane **5a** (15 mg, 39.1 µmol) was dissolved in CH_2Cl_2 (2 mL). After addition of methyl triflate (17.5 µL, 156 µmol), the reaction mixture was stirred in the dark at room temperature for 17 h. Subsequently, hexane (7 mL) was added. The resulting solid was collected by filtration and washed with hexane (5 mL) yielding the product as a colorless solid (19 mg, 34.7 µmol, 89%). ^{1}H NMR spectroscopy in combination with absorption and emission spectroscopy revealed an impurity of 0.5% starting material. To obtain pure product for spectroscopy, parts of the product were further purified by washing with hexane (20 mL) and drying *in vacuo*.

^{1}H NMR (500 MHz, CD_3OD) δ = 7.49 (s, 2 H, C*H*), 7.20 - 7.17 (m, 1 H, C*H*), 6.99 - 6.93 (m, 2 H, C*H*), 6.84 - 6.79 (m, 2 H, C*H*), 3.64 (s, 9 H, C*H3*), 2.28 (s, 3 H, C*H3*), 2.15 (s, 3 H, C*H3*), 2.13 (s, 3 H, C*H3*), 2.04 (s, 3 H, C*H3*), 2.00 (s, 3 H, C*H3*), 1.97 (s, 3 H, C*H3*), 1.95 (s, 3 H, C*H3*) ppm.

^{11}B{^{1}H} NMR (160 MHz, CD_3OD) δ = 78 (br) ppm.

^{13}C{^{1}H} NMR (125 MHz, CD_3OD) δ = 150.9, 149.1, 147.2, 144.6, 144.5, 144.0, 142.3, 142.1, 141.9, 141.7, 141.1, 131.4, 130.2, 130.0, 129.2, 129.1, 121.8 (q, $^1J_{CF}$ = 319 Hz), 119.6, 119.6, 57.4, 23.4, 23.2, 23.2, 23.2, 23.1, 21.3 ppm.

HRMS (ESI$^+$) *m/z*: [M-OTf]$^+$ found: 398.3011; calc. for [$C_{28}H_{37}BN$]: 398.3014 (|Δ| = 0.8 ppm).

Elem. Anal. Calc. (%) for $C_{29}H_{37}BF_3NO_3S$: C 63.62, H 6.81, N 2.56, S 5.86; found: C 62.21, H 6.86, N 2.72, S 5.79.

Bis-[4-(trimethylammonium)-2,6-dimethylphenyl]-2,6-dimethylphenylborane _bis_-triflate 6c

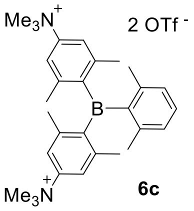

6c

Bis-[4-(*N,N*-dimethylamino)-2,6-dimethylphenyl]-2,6-dimethylphenylborane **6a** (70 mg, 0.17 mmol) was dissolved in dry, degassed CH_2Cl_2 (6.0 mL) and Et_2O (2.0 mL). After addition of methyl triflate (96.0 µL, 0.85 mmol), the reaction mixture was stirred at room temperature for 20 h. The resulting precipitate was collected by filtration and washed with Et_2O yielding the product as a colorless solid (117 mg, 93%).

^{1}H NMR (500 MHz, acetone-d$_6$): δ = 7.79 – 7.74 (m, 4 H, C*H*), 7.29 – 7.25 (m, 1 H, C*H*), 7.06 – 7.02 (m, 2 H, C*H*), 3.86 (s, 18 H, CH_3), 2.18 (s, 6 H, CH_3), 2.15 (s, 6 H, CH_3), 2.05 (s, 6 H, CH_3) ppm.

^{11}B{^{1}H} NMR (160 MHz, acetone-d$_6$): δ = 78 (br) ppm.

^{13}C{^{1}H} NMR (125 MHz, acetone-d$_6$): δ = 149.3, 144.0, 143.8, 141.4, 132.0, 129.2, 122.2 (q, $J_{C\text{-}F}$ = 322 Hz), 120.2, 120.0, 57.6, 23.4, 23.3 ppm.

HRMS (ESI$^+$): m/z found: [M-OTf $^-$]$^+$ 590.30737; calc. for $[C_{31}H_{43}{}^{10}BF_3N_2O_3S]^+$ 590.30704 (|Δ| = 0.6 ppm).

Elem. Anal. Calc. (%) for $C_{32}H_{43}BF_6N_2O_6S_2$: C 51.89, H 5.85, N 3.78, S 8.66; found: C 52.10, H 5.84, N 3.88, S 8.39.

NMR Spectra

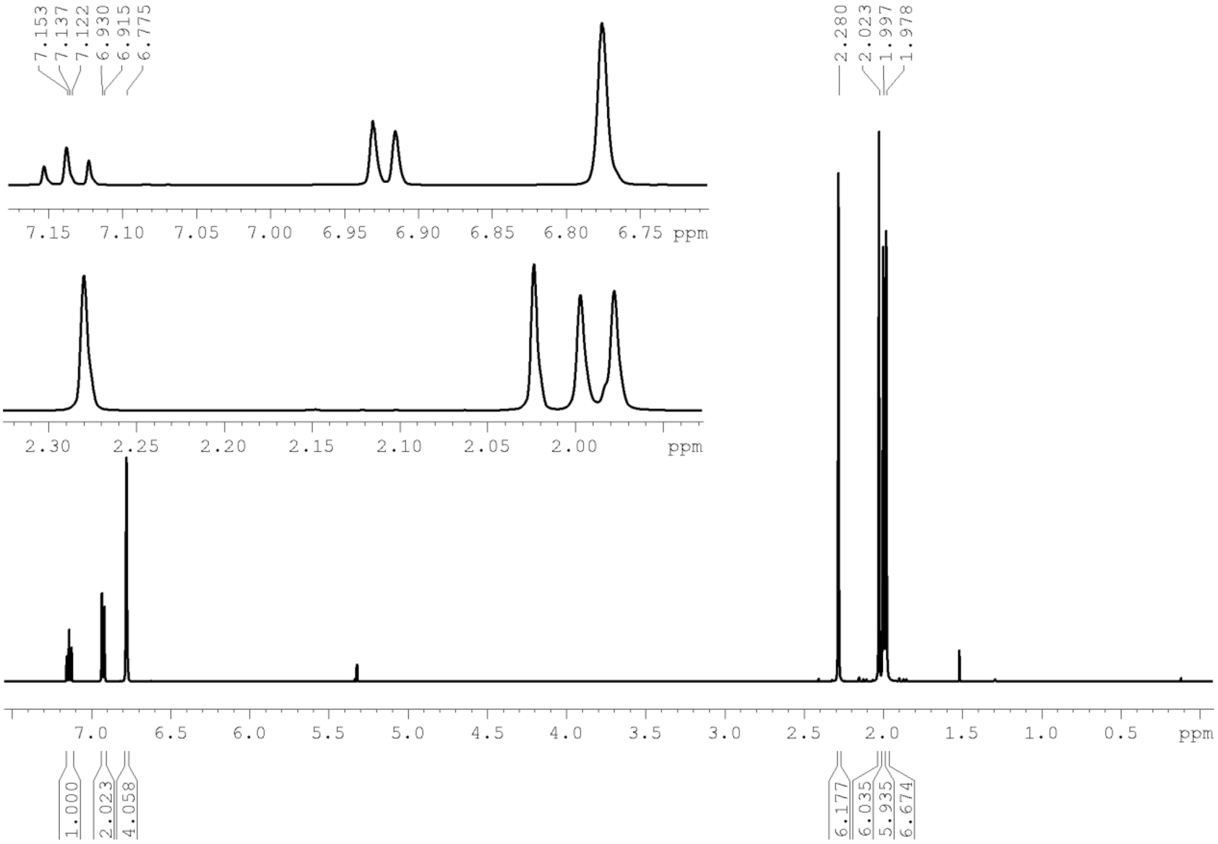

Figure S1: ^{1}H NMR spectrum of compound **4a** recorded in CD_2Cl_2 at 500 MHz.

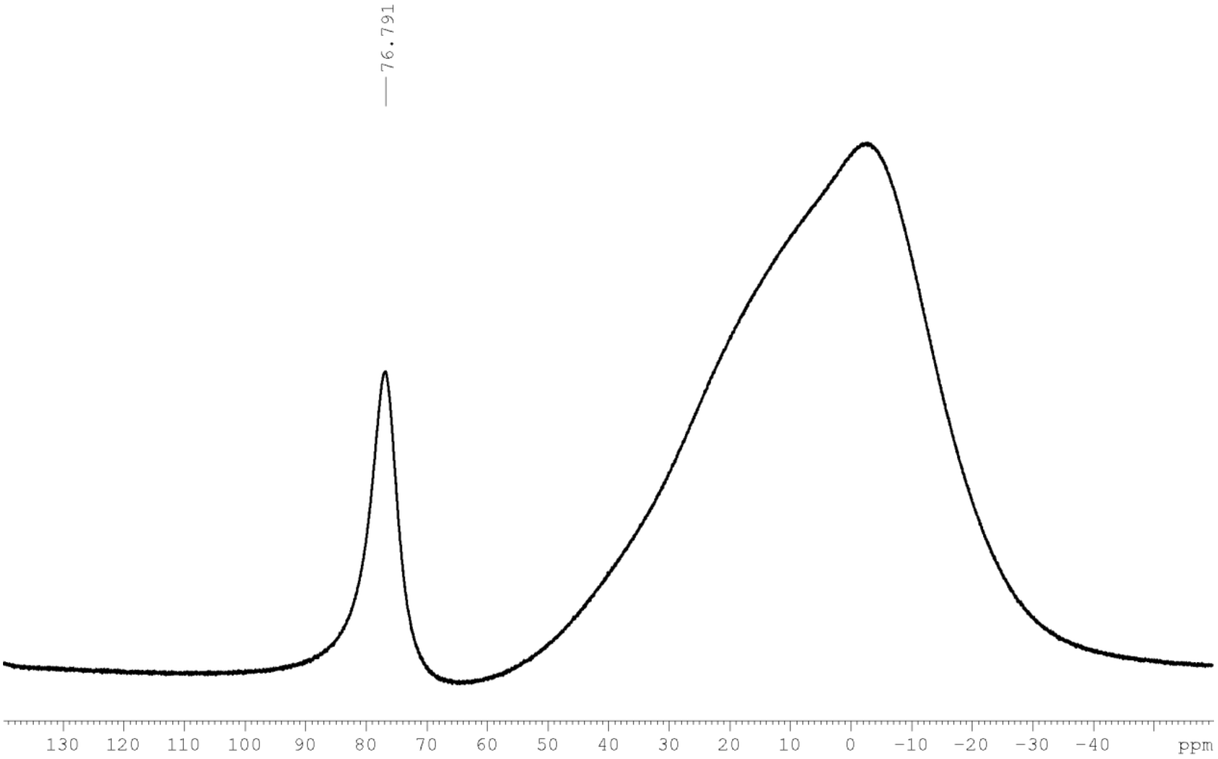

Figure S2: $^{11}B\{^{1}H\}$ NMR spectrum of compound **4a** recorded in CD_2Cl_2 at 160 MHz.

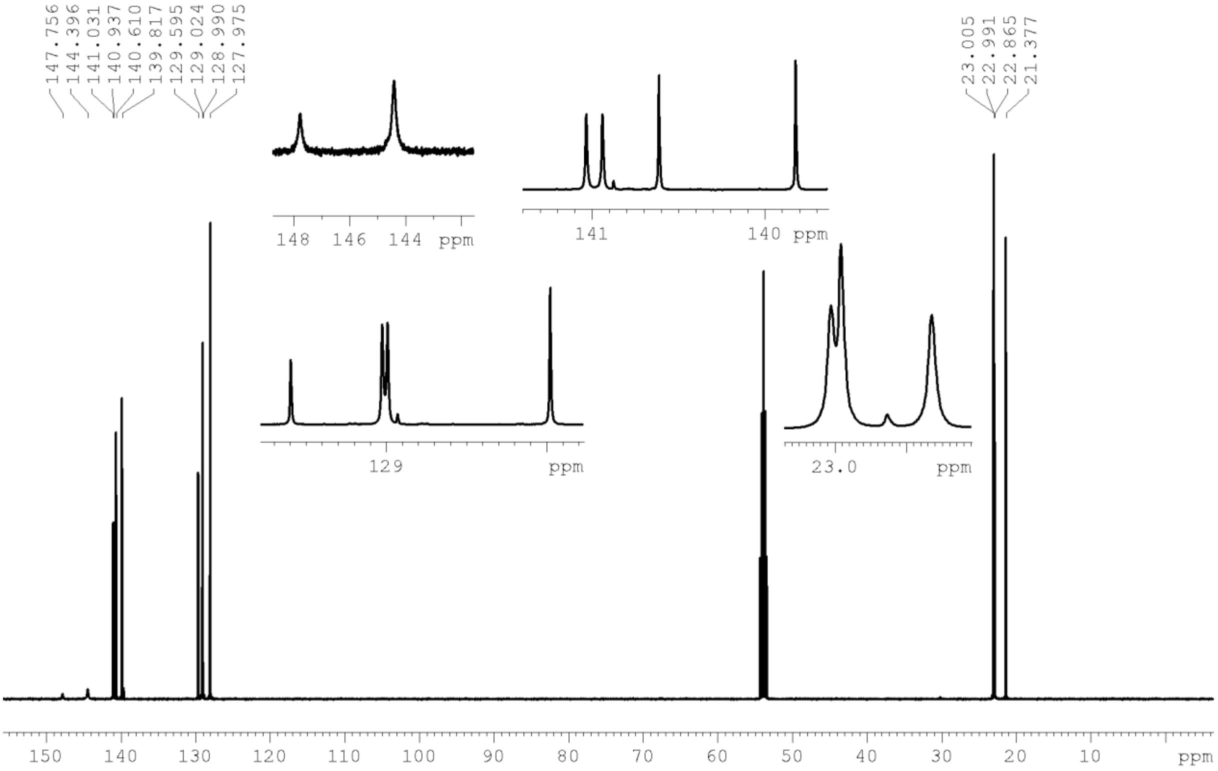

Figure S3: $^{13}C\{^{1}H\}$ NMR spectrum of compound **4a** recorded in CD_2Cl_2 at 125 MHz.

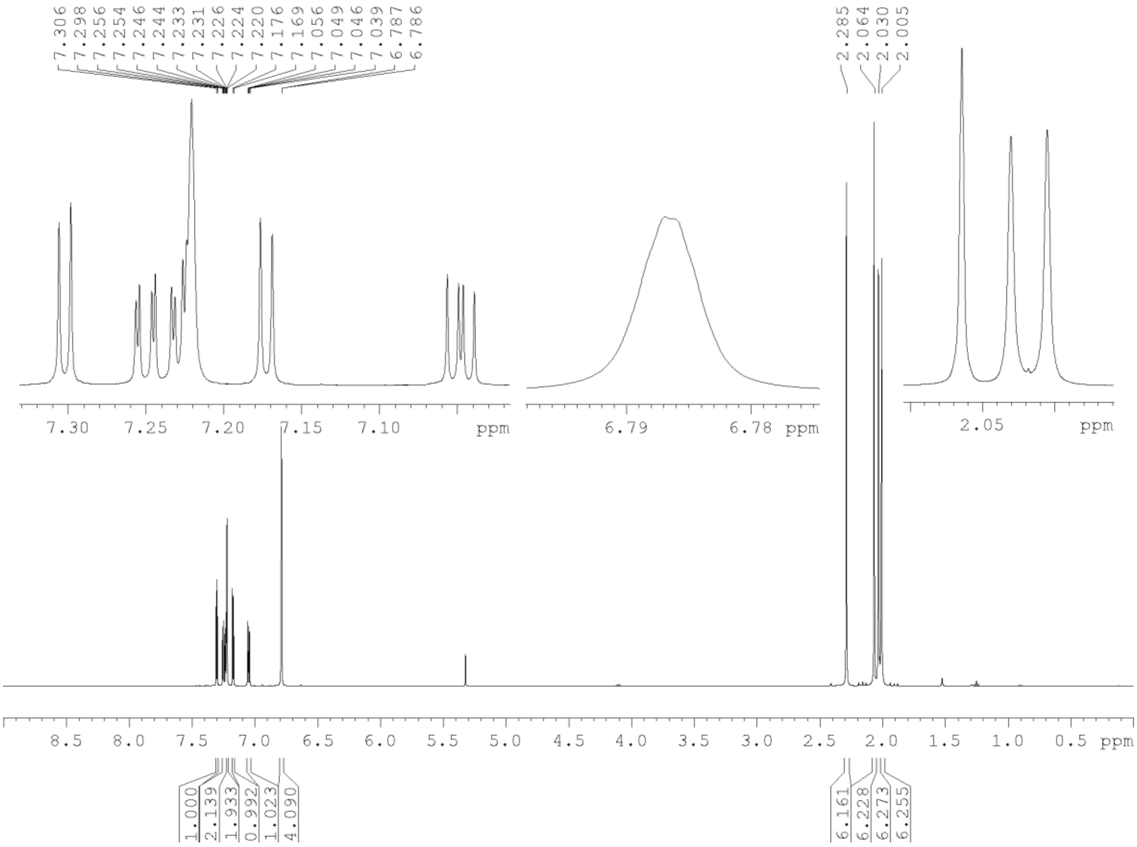

Figure S4: ^{1}H NMR spectrum of **7a** recorded in CD_2Cl_2 at 500 MHz.

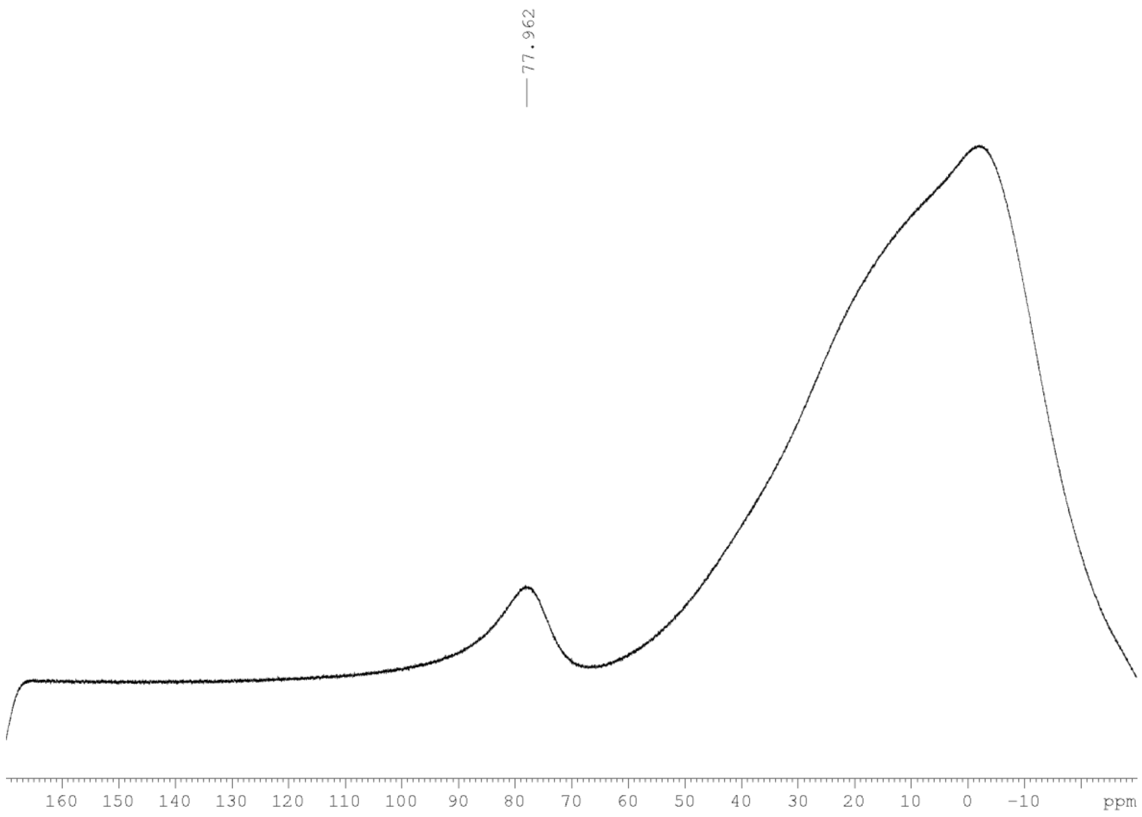

Figure S5: $^{11}B\{^{1}H\}$ NMR spectrum of **7a** recorded in CD_2Cl_2 at 160 MHz.

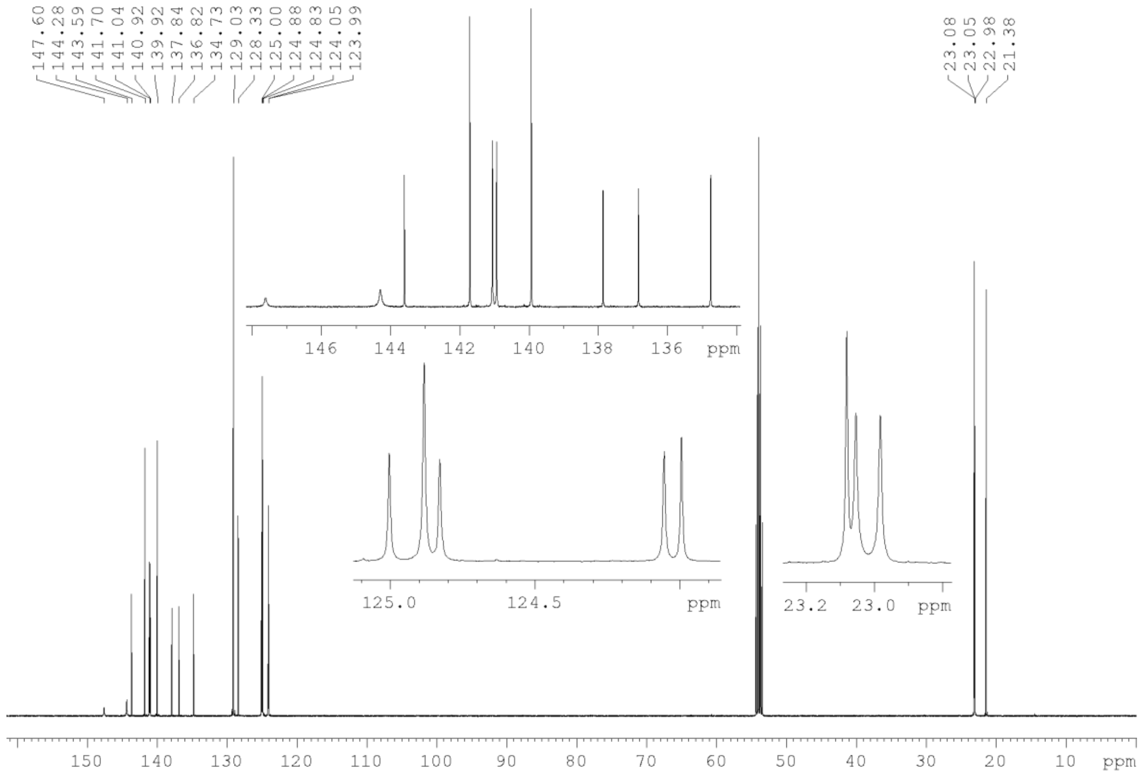

Figure S6: $^{13}C\{^{1}H\}$ NMR spectrum of **7a** recorded in CD_2Cl_2 at 125 MHz.

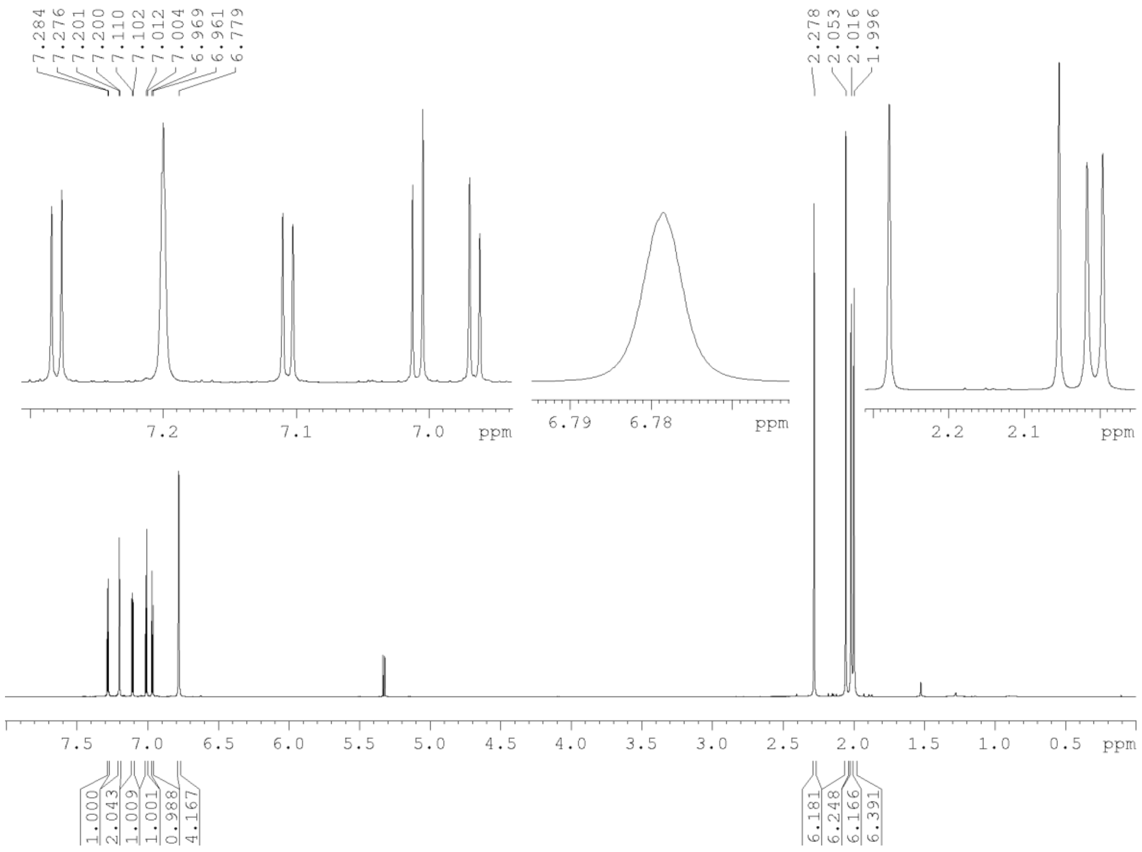

Figure S7: ^{1}H NMR spectrum of **7b** recorded in CD_2Cl_2 at 500 MHz.

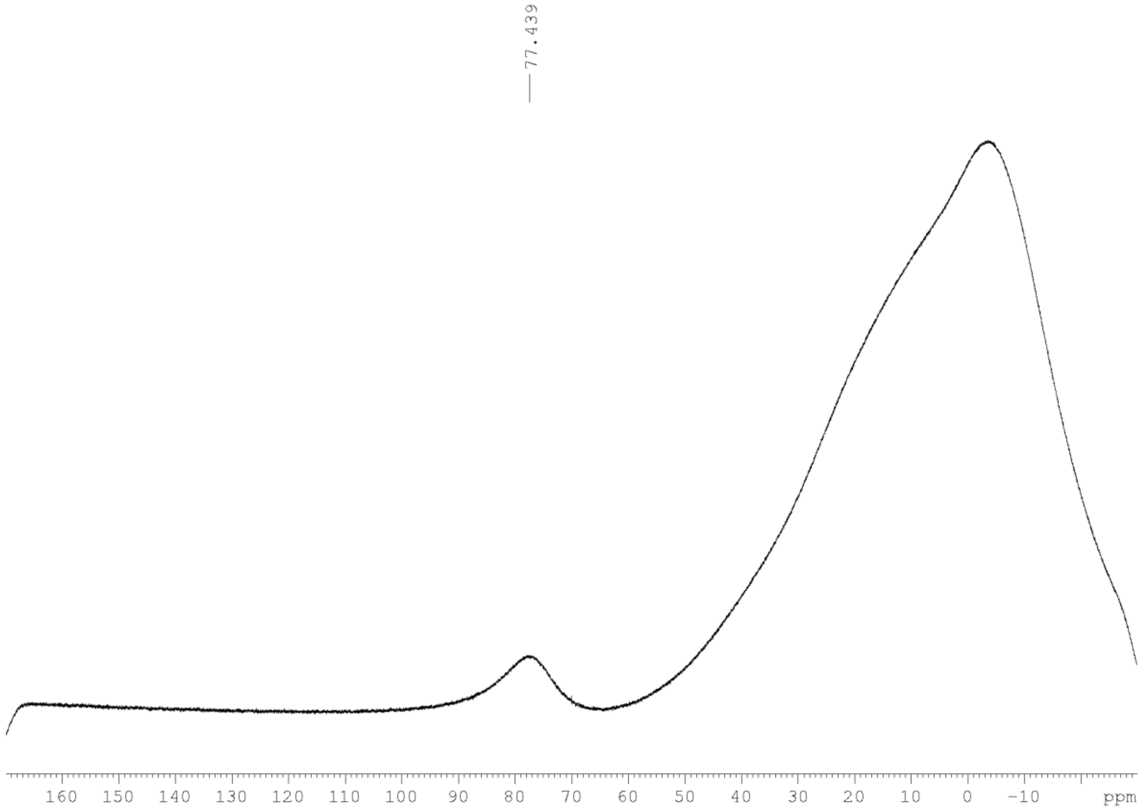

Figure S8: $^{11}B\{^{1}H\}$ NMR spectrum of **7b** recorded in CD_2Cl_2 at 160 MHz.

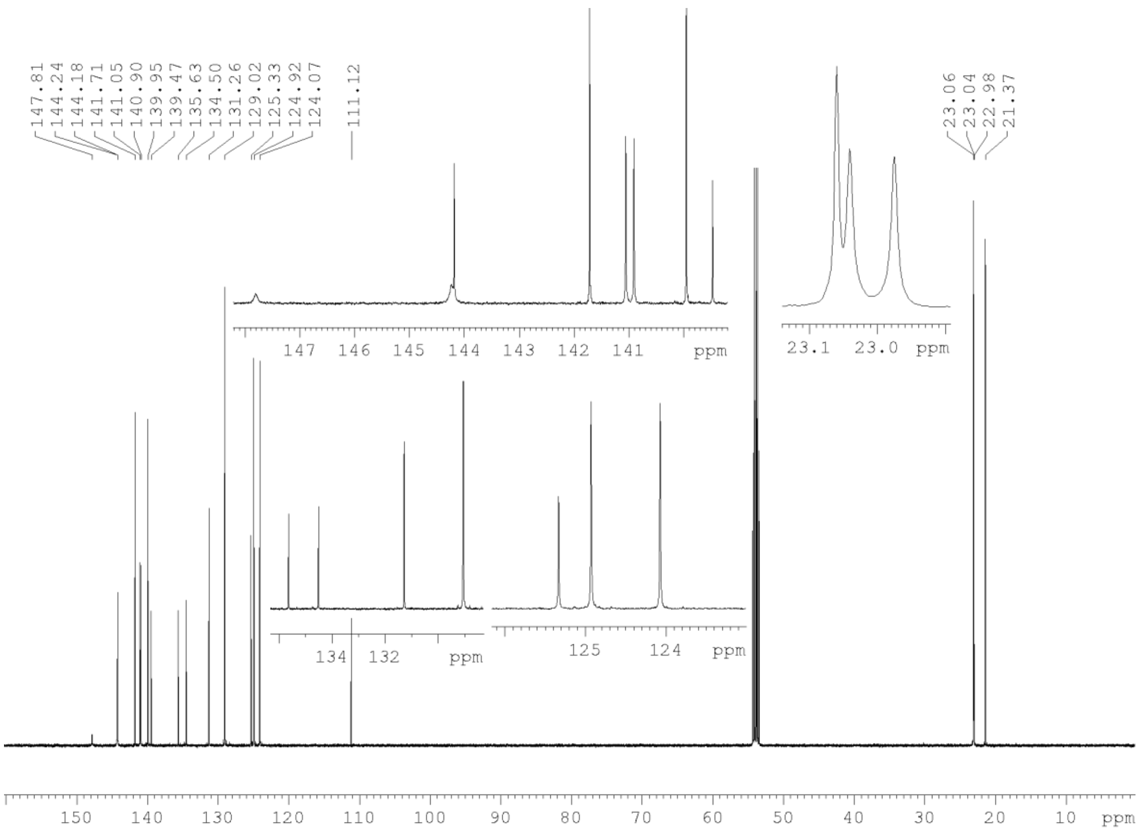

Figure S9: $^{13}C\{^1H\}$ NMR spectrum of **7b** recorded in CD_2Cl_2 at 125 MHz.

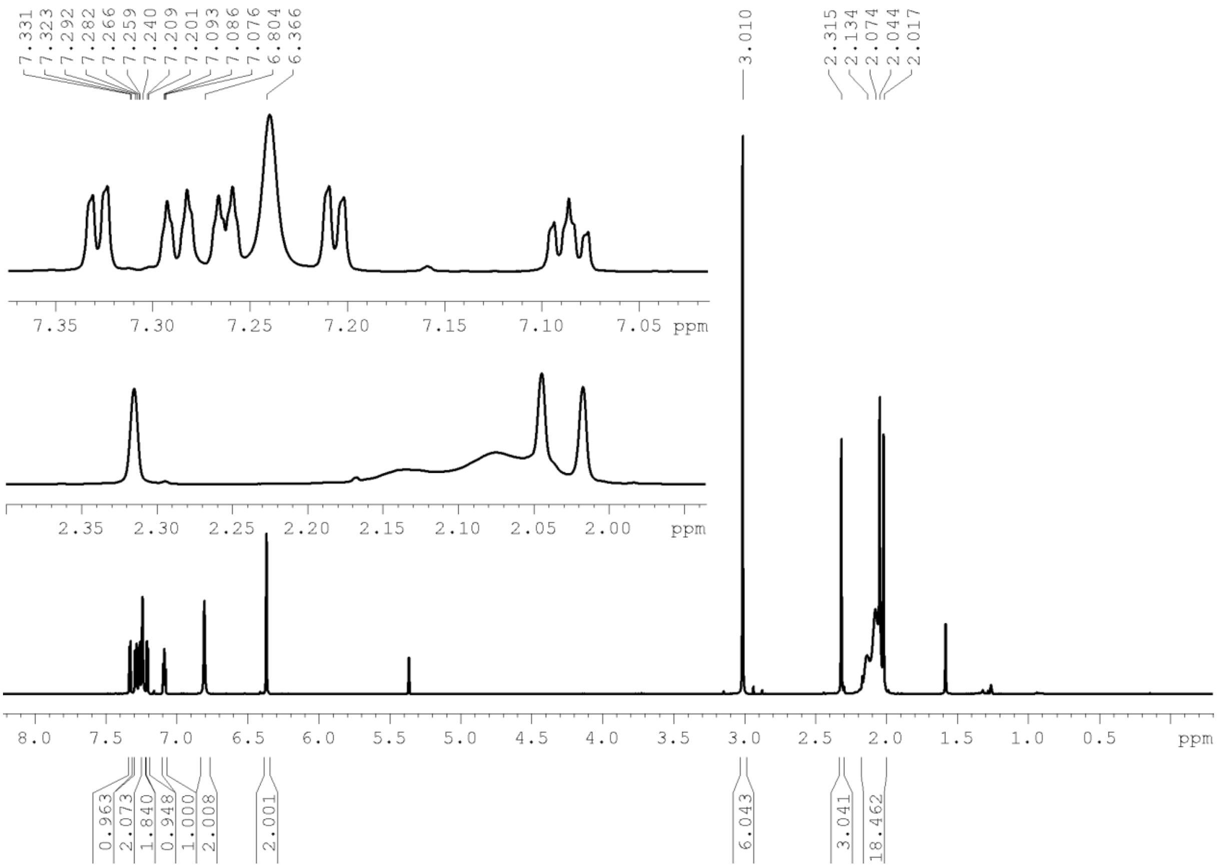

Figure S10: ^1H NMR spectrum of **8a** recorded in CD_2Cl_2 at 500 MHz.

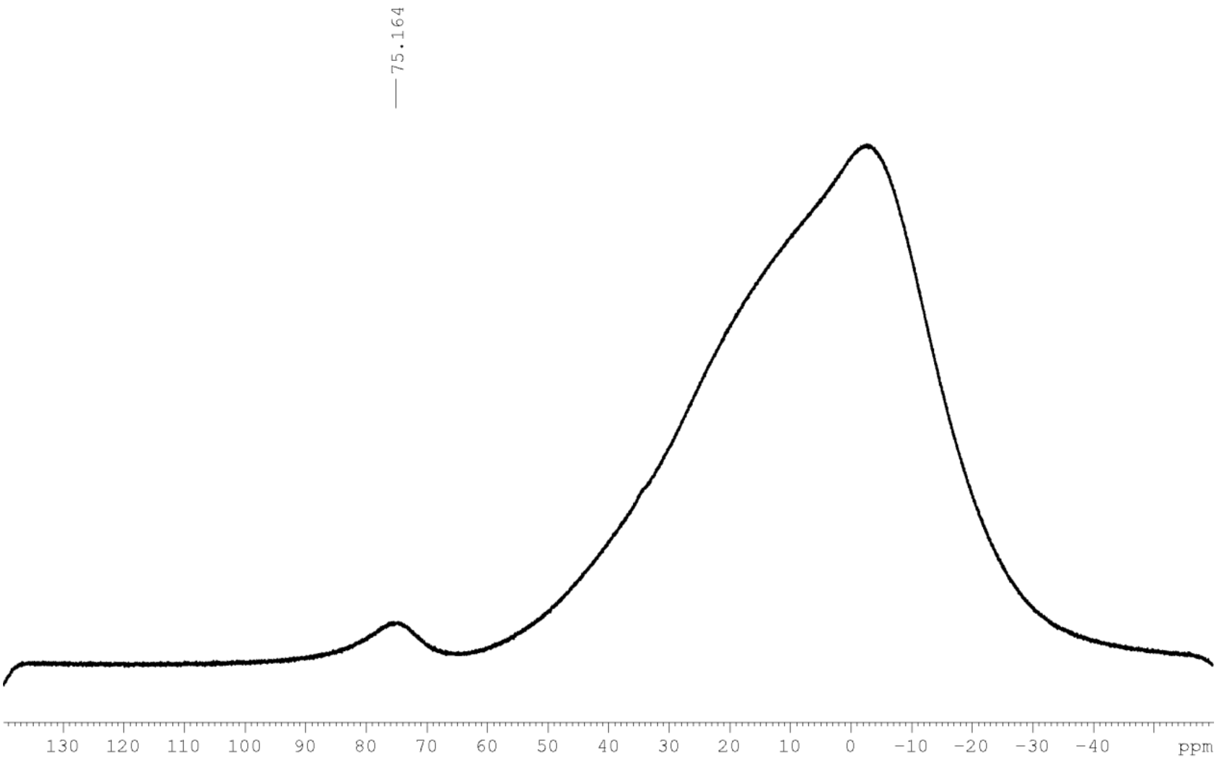

Figure S11: $^{11}B\{^{1}H\}$ NMR spectrum of **8a** recorded in CD_2Cl_2 at 160 MHz.

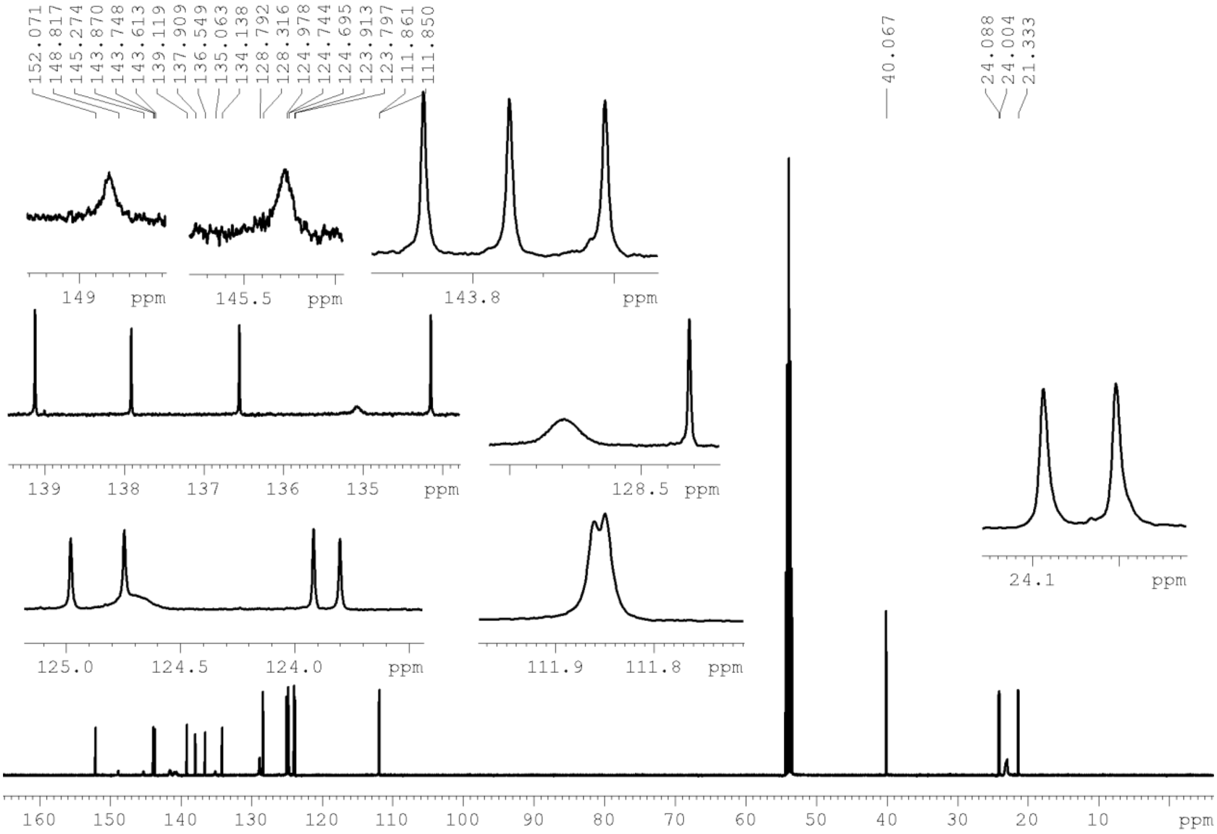

Figure S12: $^{13}C\{^{1}H\}$ NMR spectrum of **8a** recorded in CD_2Cl_2 at 125 MHz.

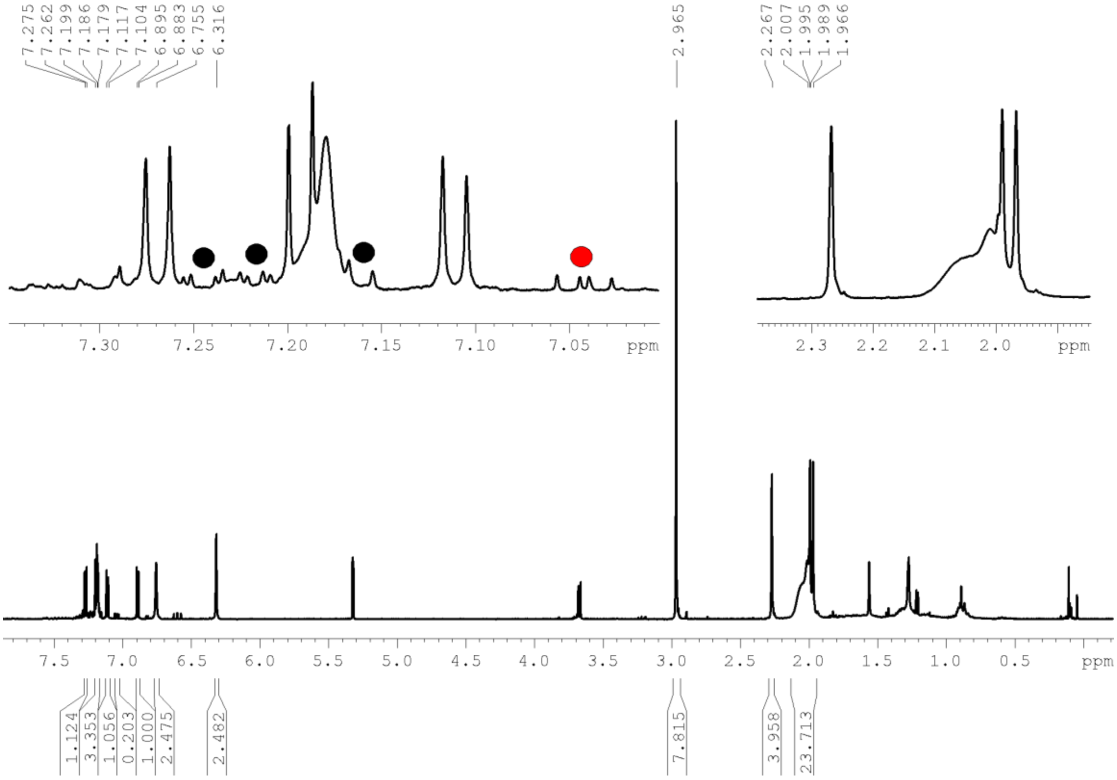

Figure S13: ^{1}H NMR spectrum of compound **8b** recorded in CD_2Cl_2 at 300 MHz. Dots mark remaining starting material. Red dot marks signal which was used for estimation of ratio of starting material to product.

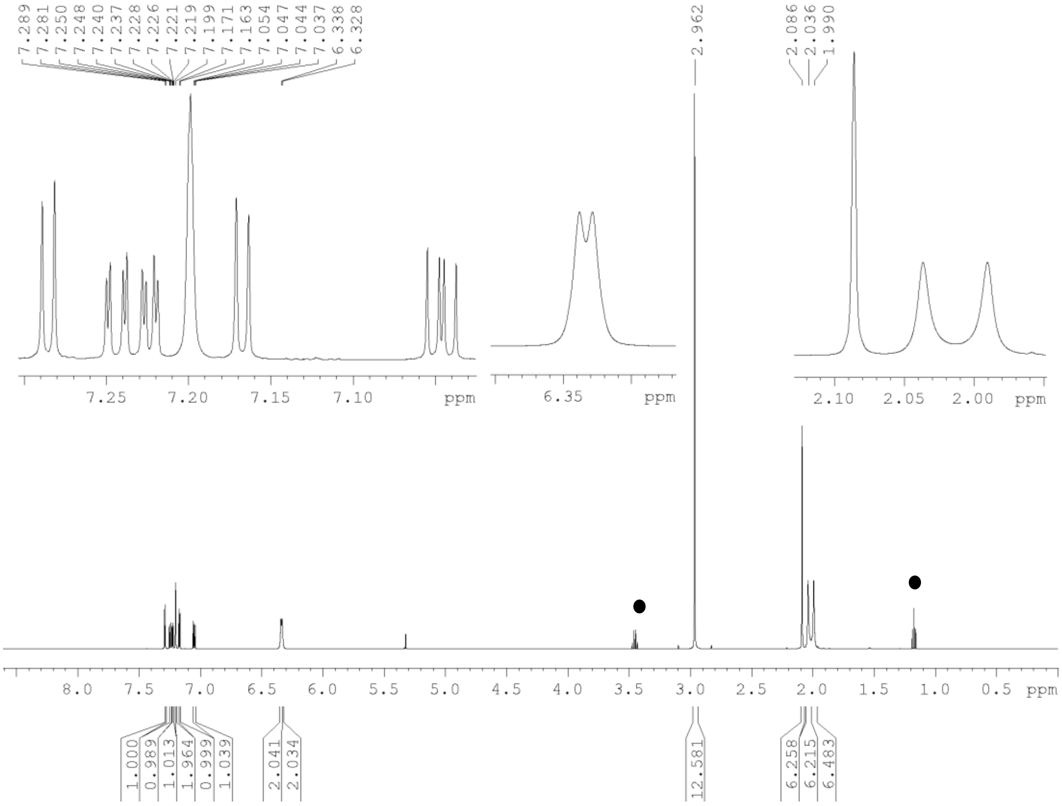

Figure S14: ^{1}H NMR spectrum of **9a** recorded in CD_2Cl_2 at 500 MHz. Dots mark remaining diethyl ether.

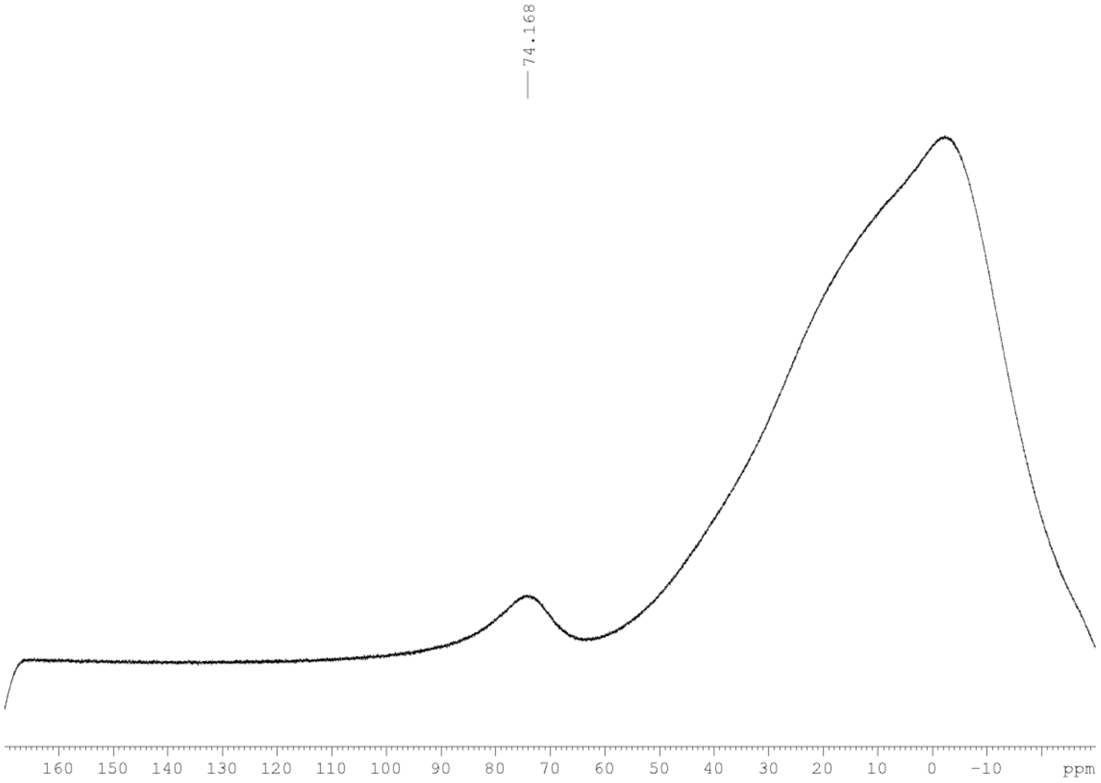

Figure S15: ^{11}B{^{1}H} NMR spectrum of **9a** recorded in CD_2Cl_2 at 160 MHz.

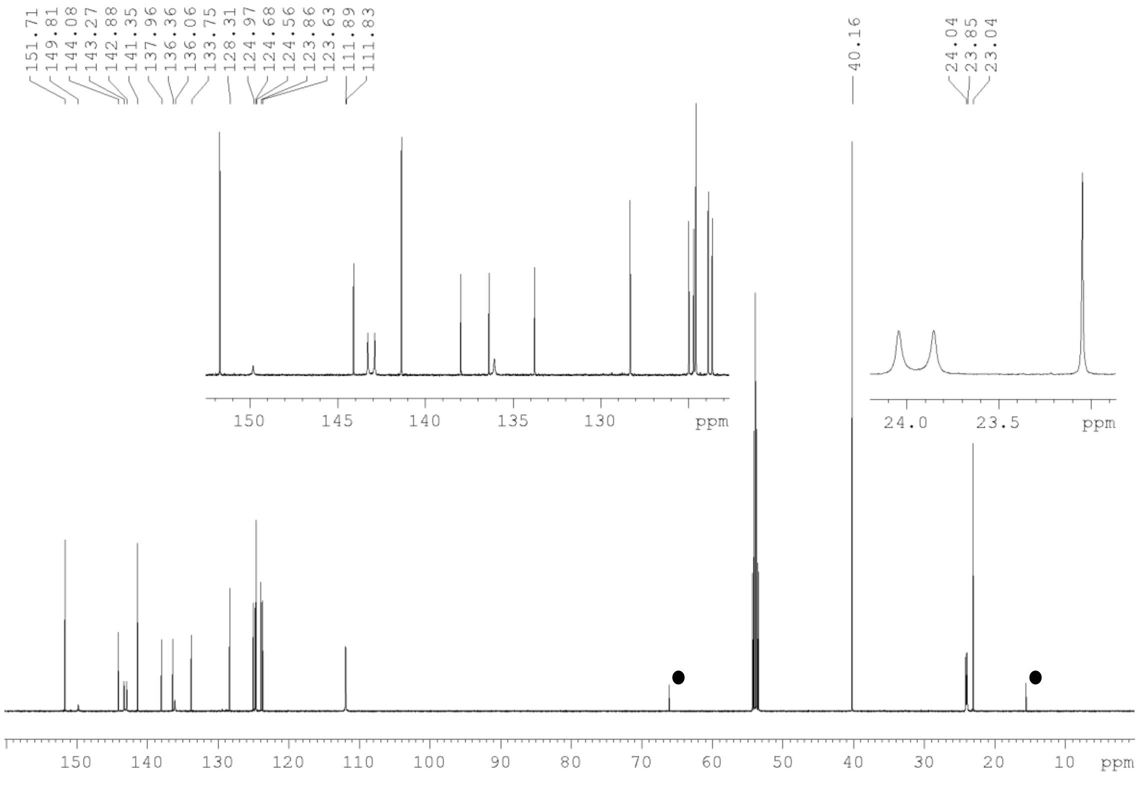

Figure S16: ^{13}C{^{1}H} NMR spectrum of **9a** recorded in CD_2Cl_2 at 125 MHz. Dots mark remaining diethyl ether.

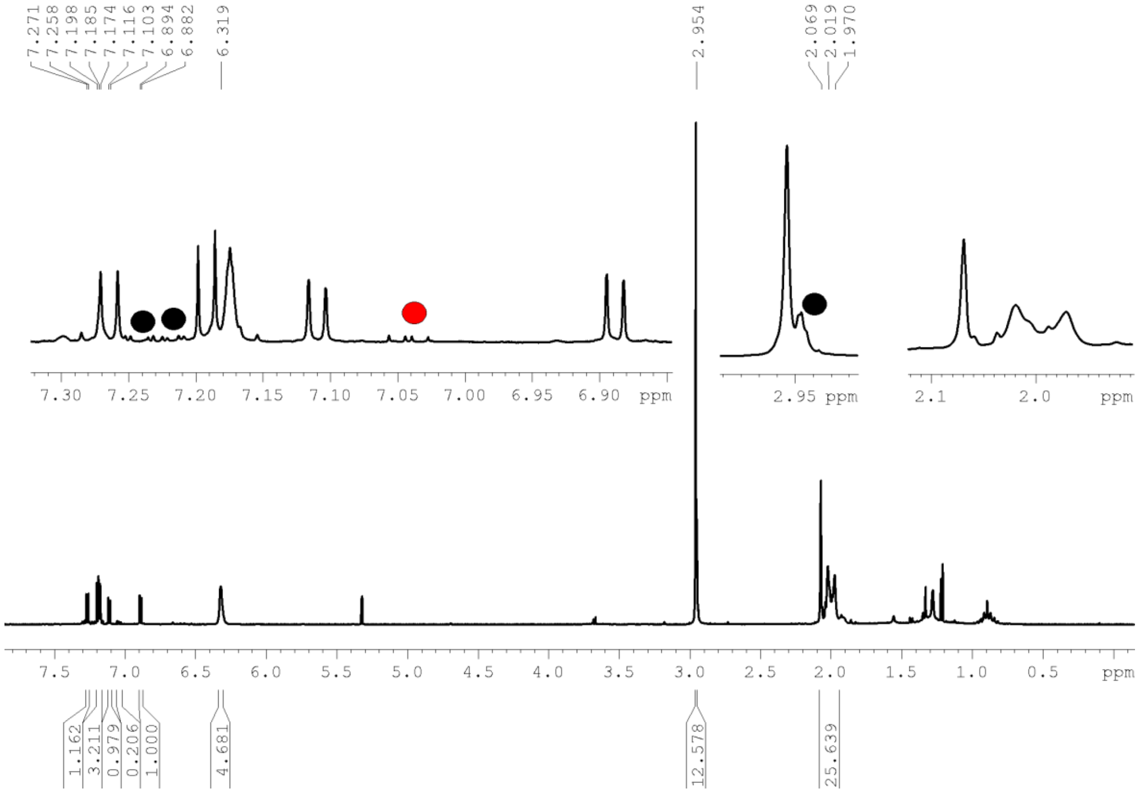

Figure S17: ^{1}H NMR spectrum of compound **9b** recorded in CD_2Cl_2 at 300 MHz. Dots mark remaining starting material. The red dot marks the signal which was used for estimation of the ratio of starting material to product.

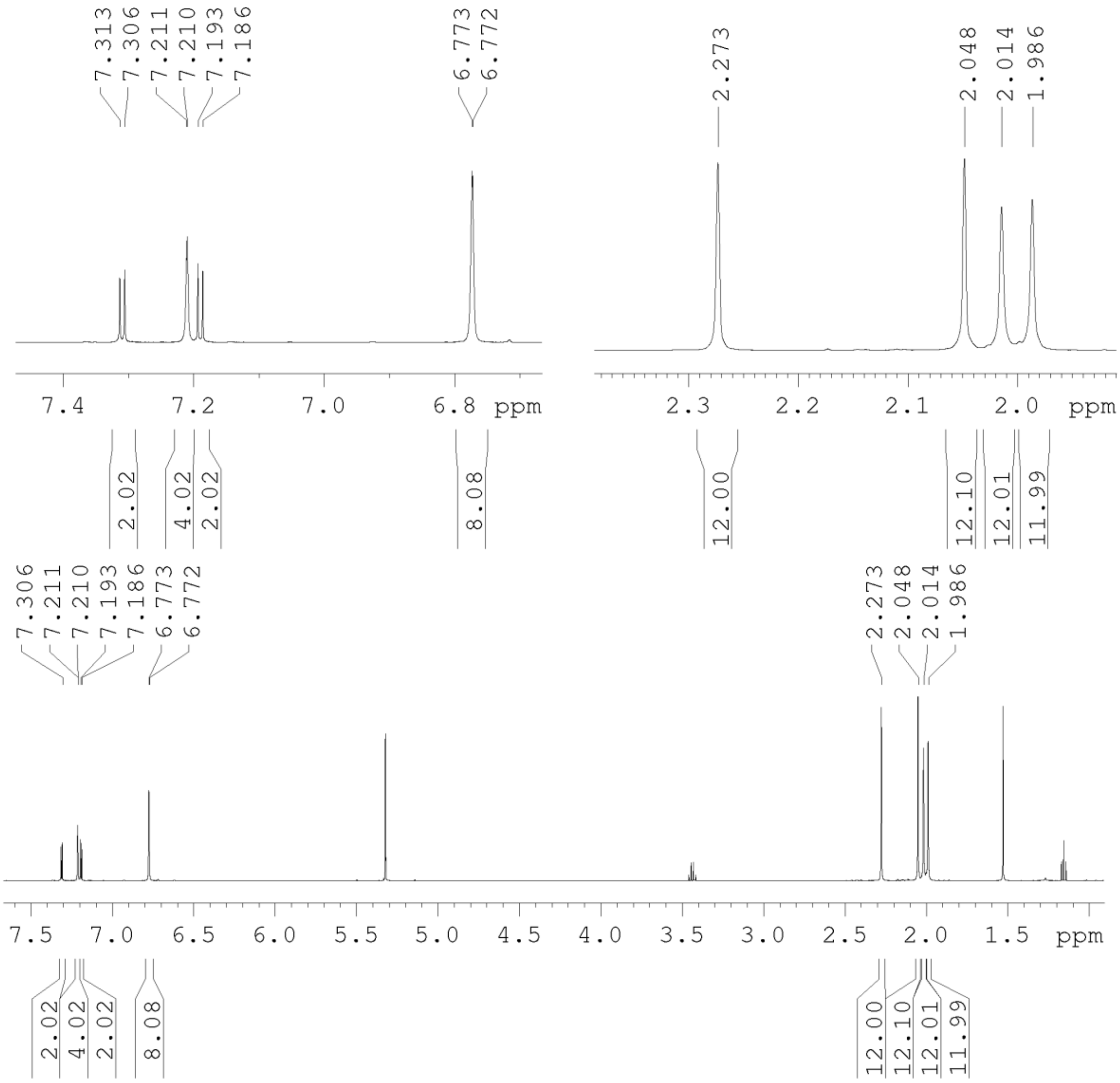

Figure S18: ^{1}H NMR spectrum of compound **Neut0** recorded in CD_2Cl_2 at 500 MHz.

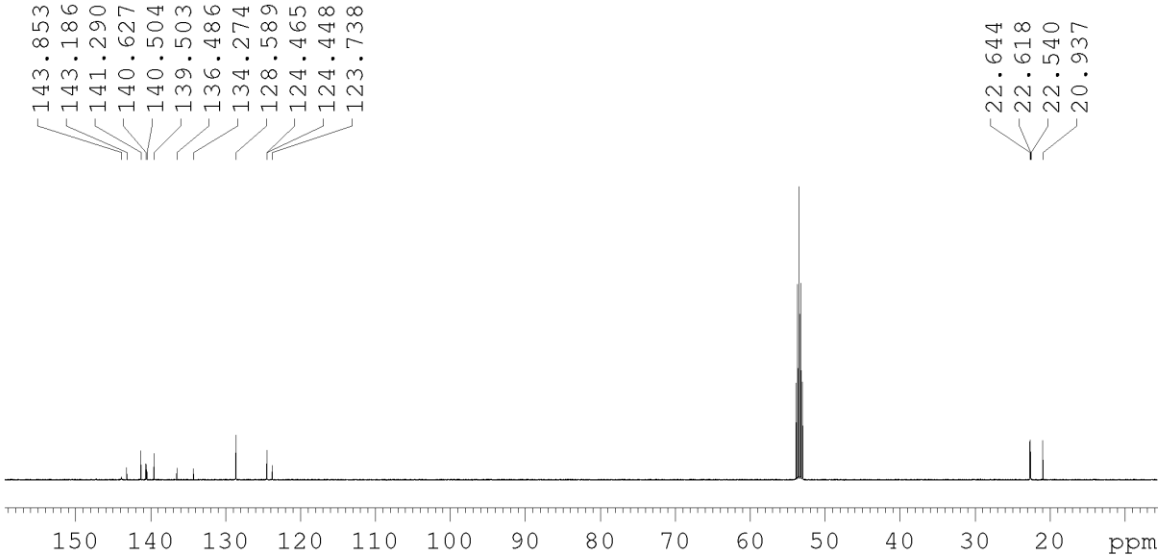

Figure S19: $^{13}C\{^{1}H\}$ NMR spectrum of compound **Neut0** recorded in CD_2Cl_2 at 125 MHz.

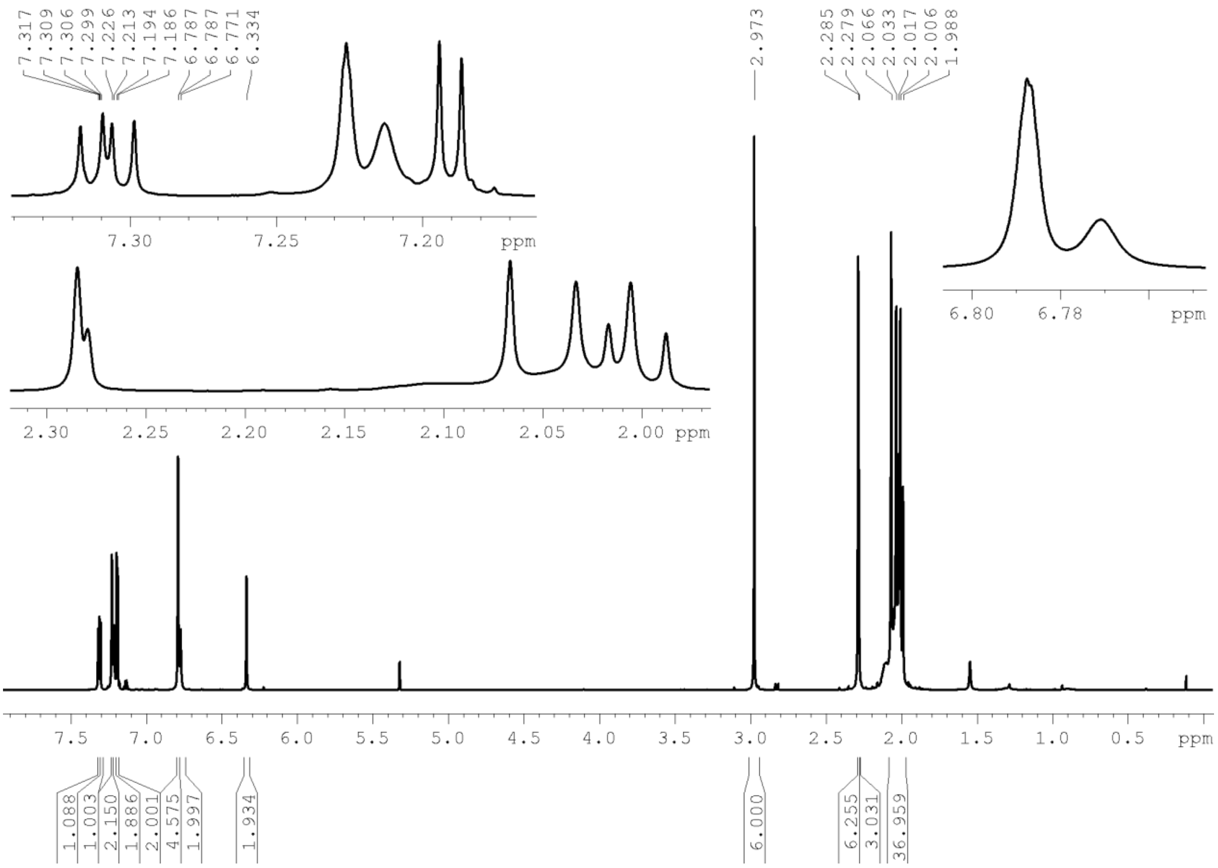

Figure S20: ^{1}H NMR spectrum of compound **Neut1** recorded in CD_2Cl_2 at 500 MHz.

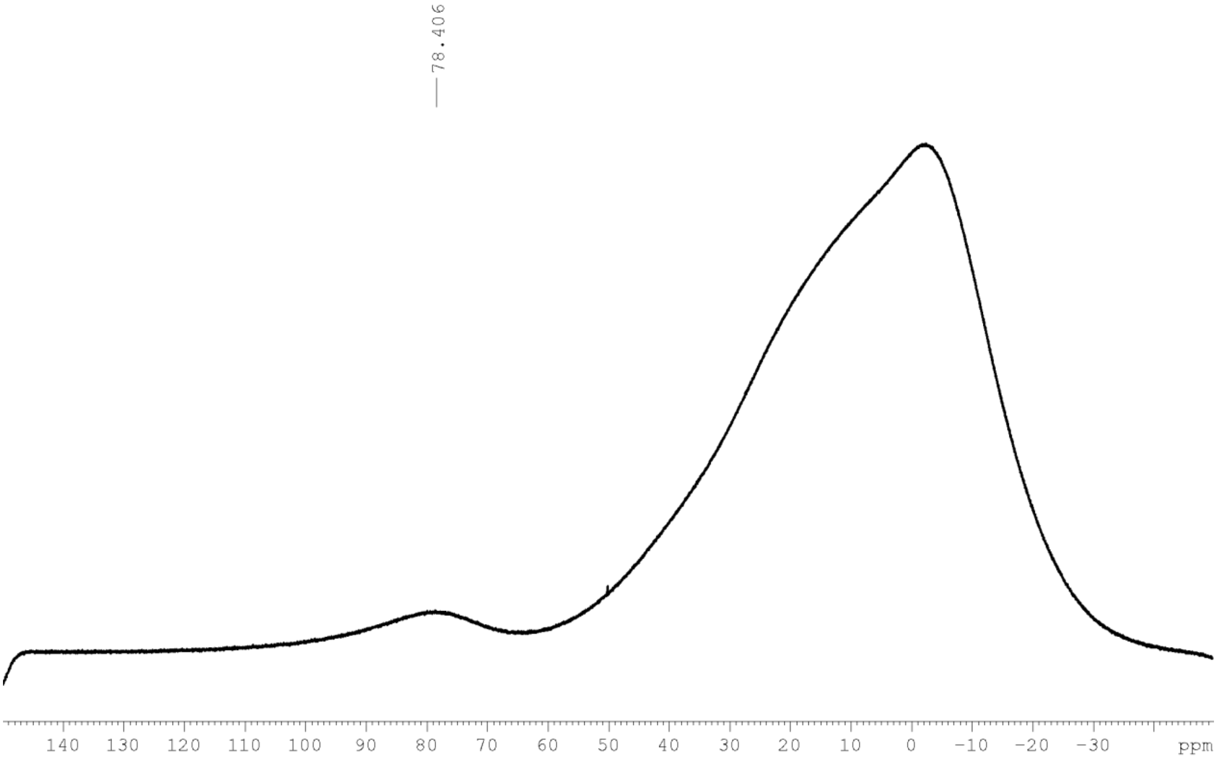

Figure S21: $^{11}B\{^{1}H\}$ NMR spectrum of compound **Neut1** recorded in CD_2Cl_2 at 160 MHz.

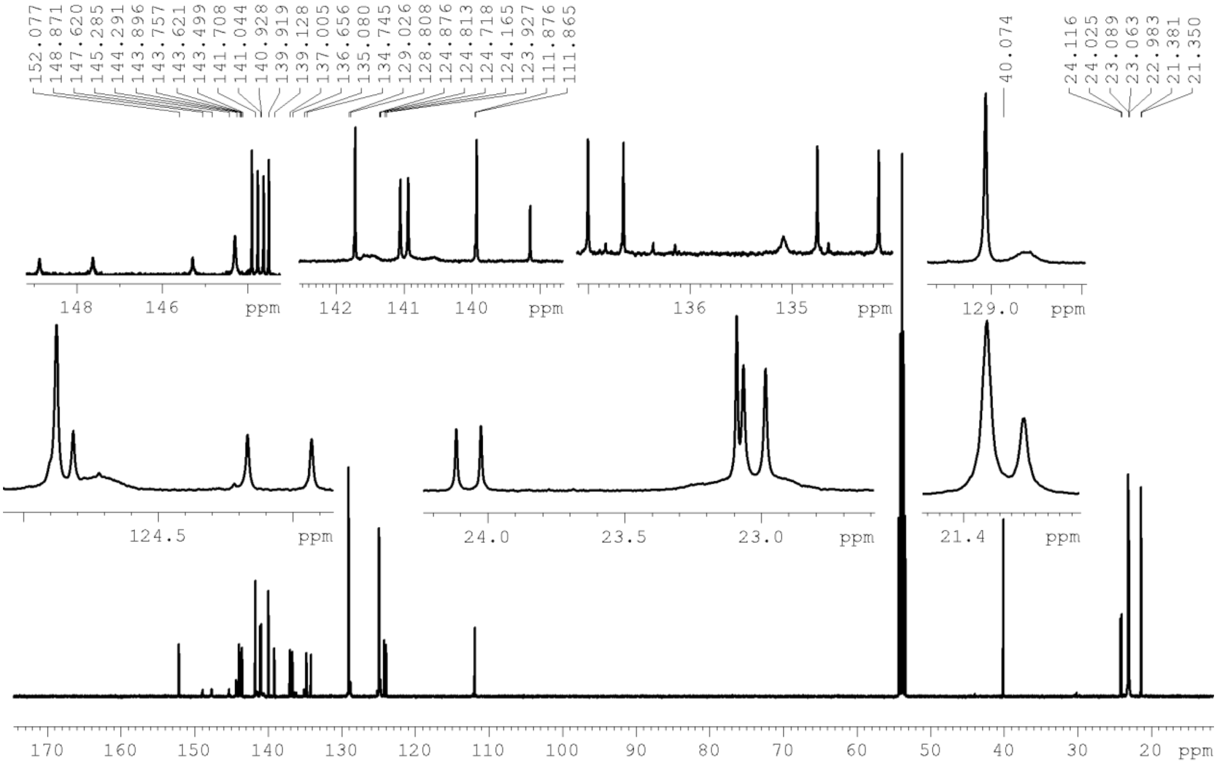

Figure S22: $^{13}C\{^{1}H\}$ NMR spectrum of compound **Neut1** recorded in CD_2Cl_2 at 125 MHz.

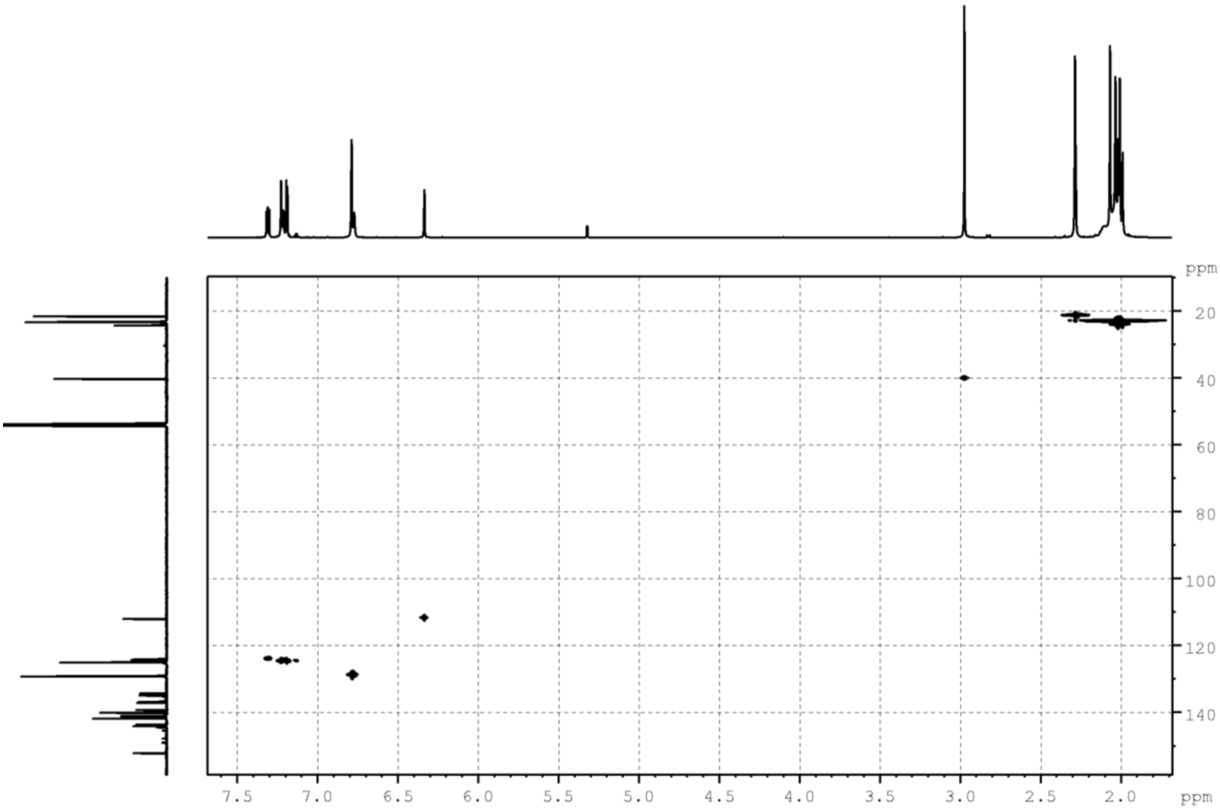

Figure S23: ^{1}H – ^{13}C HSQC spectrum of compound **Neut1** recorded in CD_2Cl_2 at 500 MHz.

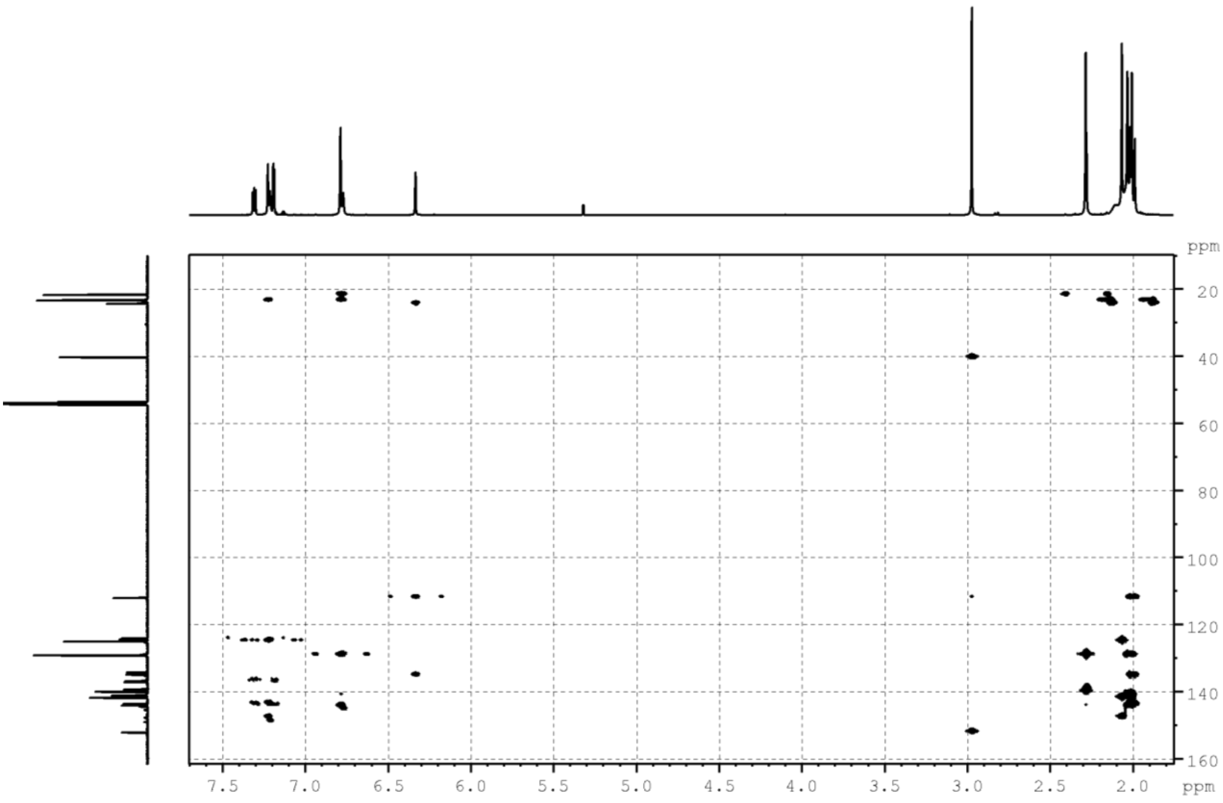

Figure S24: ^{1}H – ^{13}C HMBC spectrum of compound **Neut1** recorded in CD_2Cl_2 at 500 MHz.

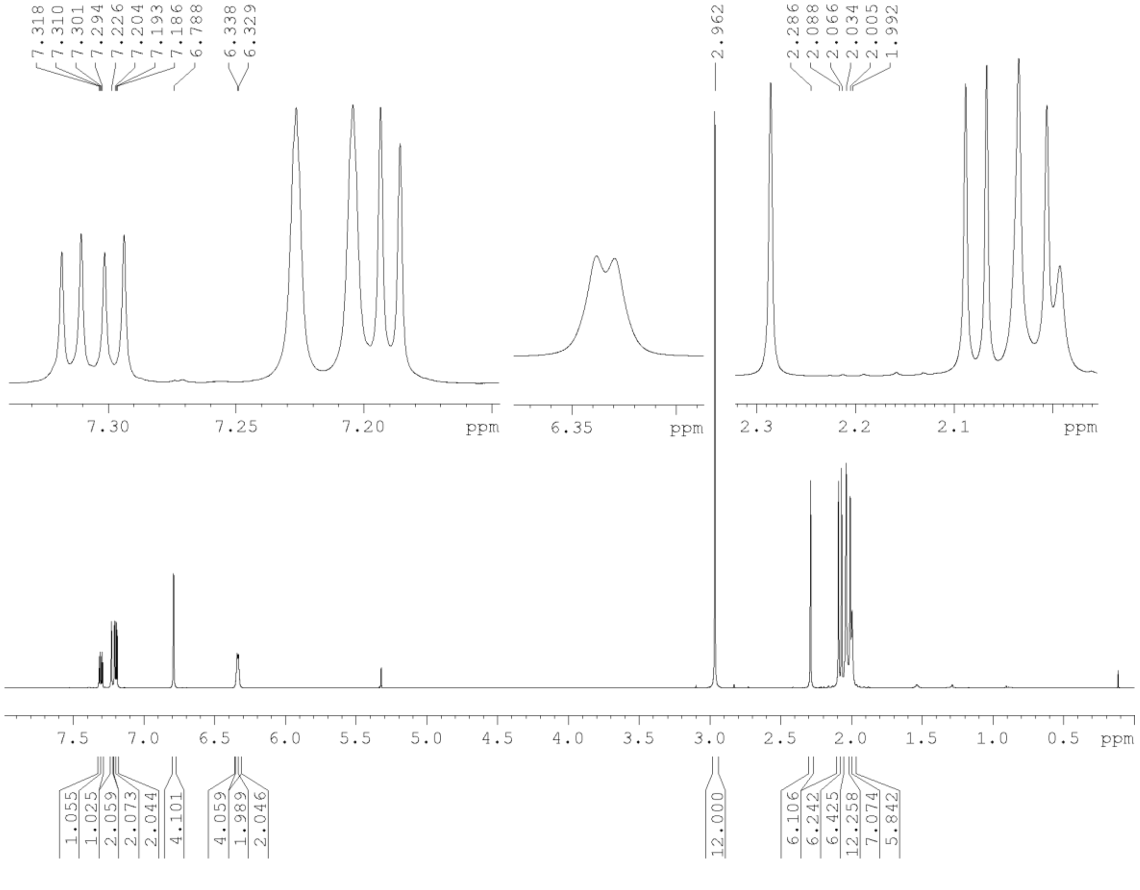

Figure S25: ^{1}H NMR spectrum of **Neut2** recorded in CD_2Cl_2 at 500 MHz.

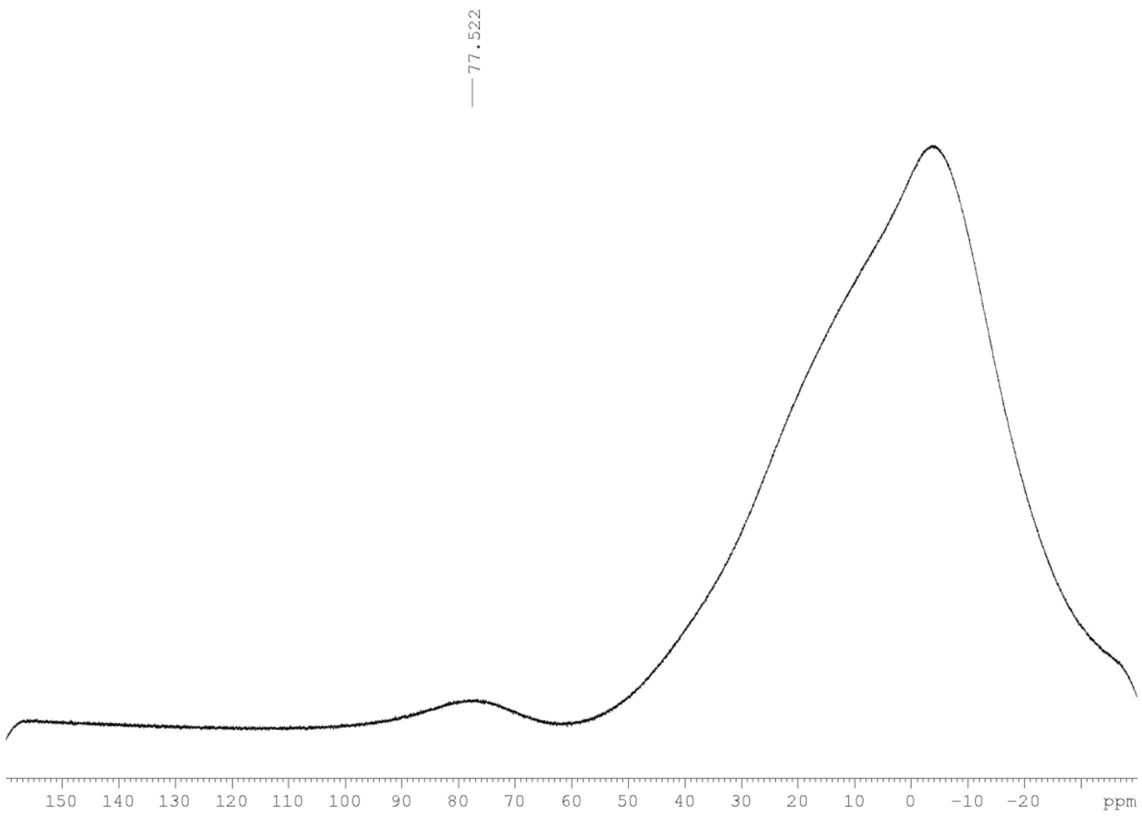

Figure S26: $^{11}B\{^{1}H\}$ NMR spectrum of **Neut2** recorded in CD_2Cl_2 at 160 MHz.

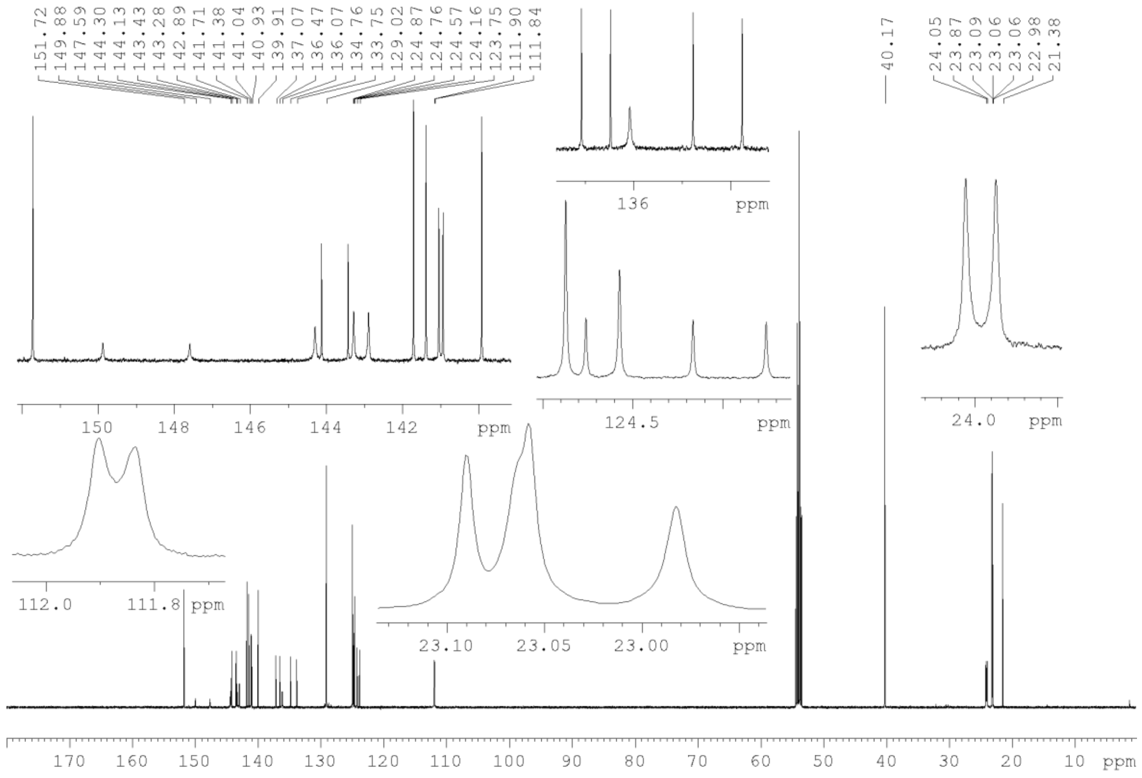

Figure S27: $^{13}C\{^{1}H\}$ NMR spectrum of **Neut2** recorded in CD_2Cl_2 at 125 MHz.

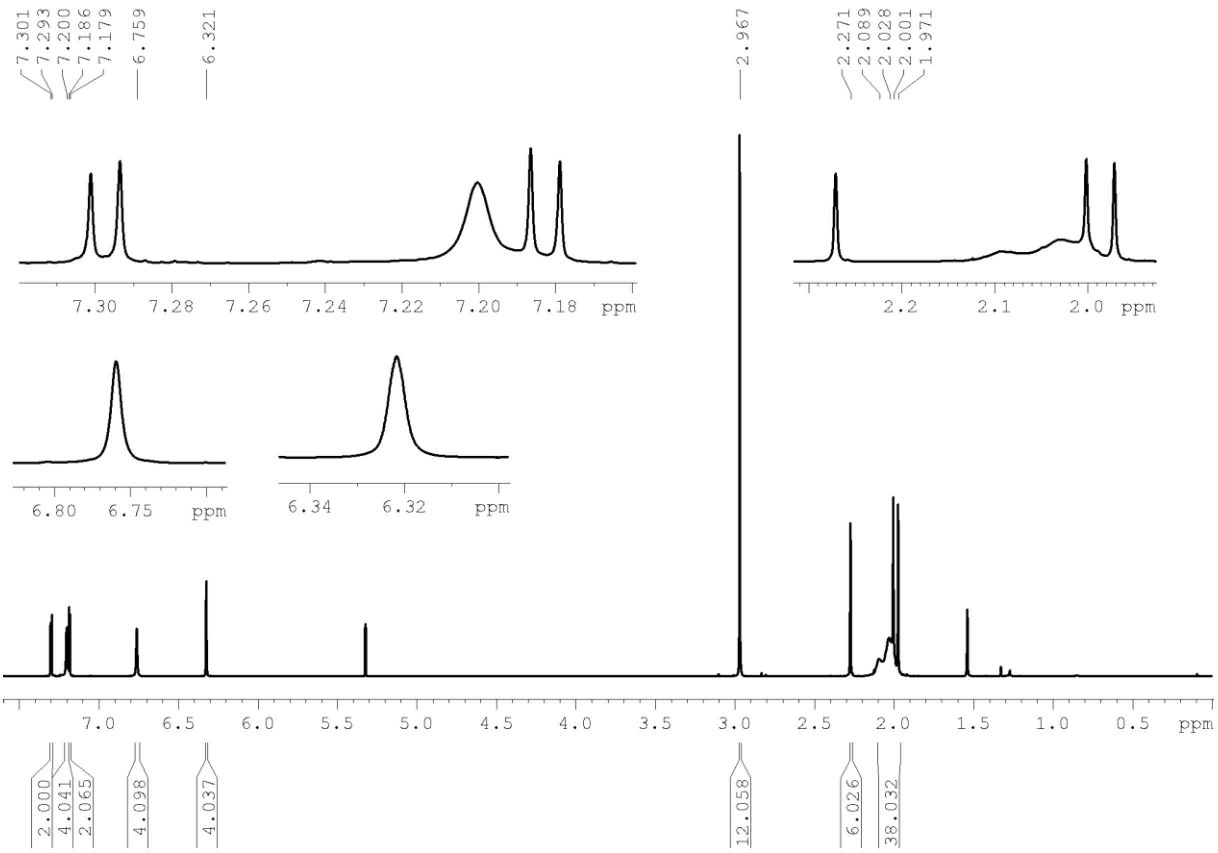

Figure S28: ^{1}H NMR spectrum of compound **Neut(i)2** recorded in CD_2Cl_2 at 500 MHz.

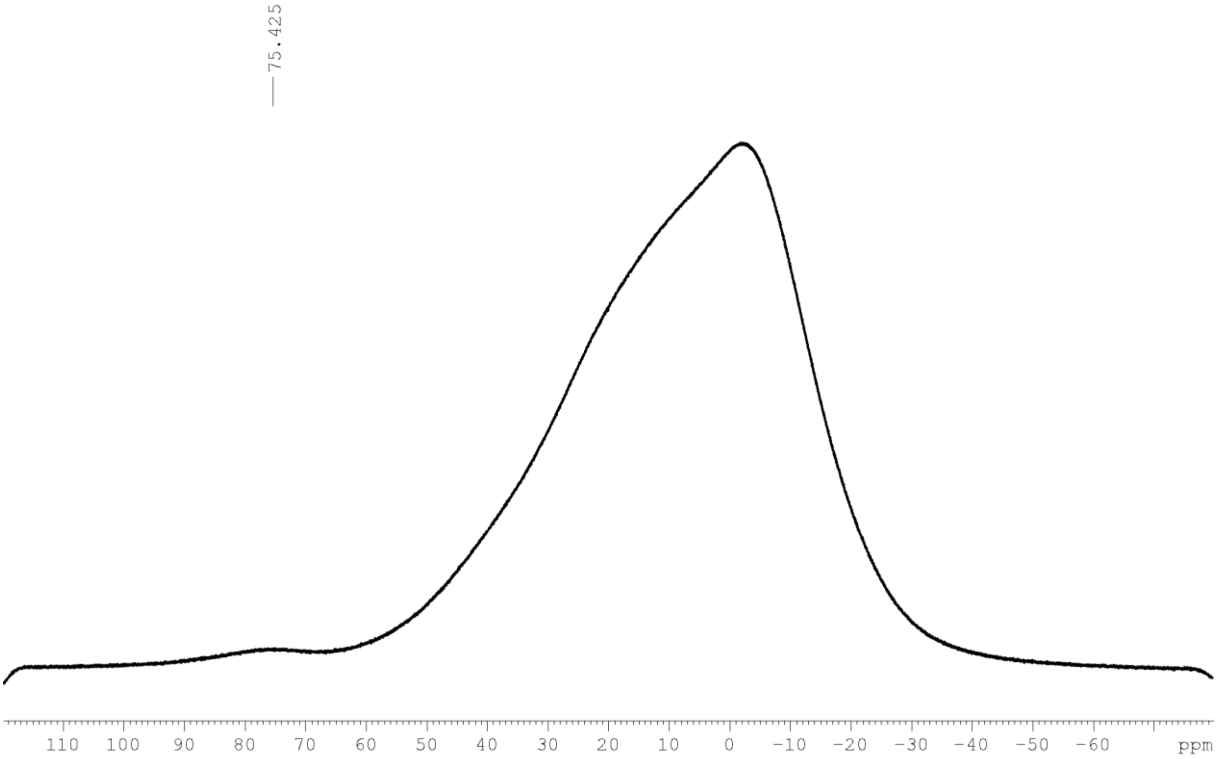

Figure S29: $^{11}B\{^{1}H\}$ NMR spectrum of compound **Neut(i)2** recorded in CD_2Cl_2 at 160 MHz.

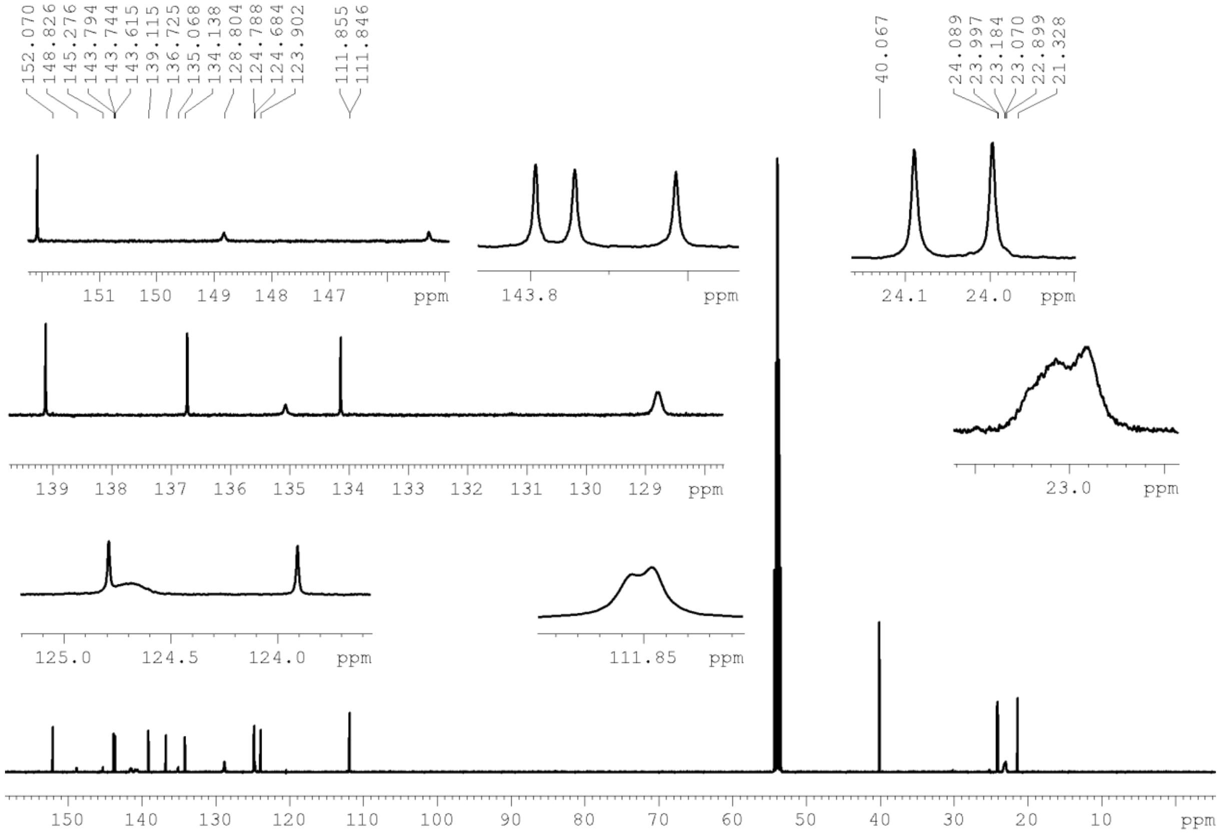

Figure S30: $^{13}C\{^{1}H\}$ NMR spectrum of compound **Neut(i)2** recorded in CD_2Cl_2 at 125 MHz.

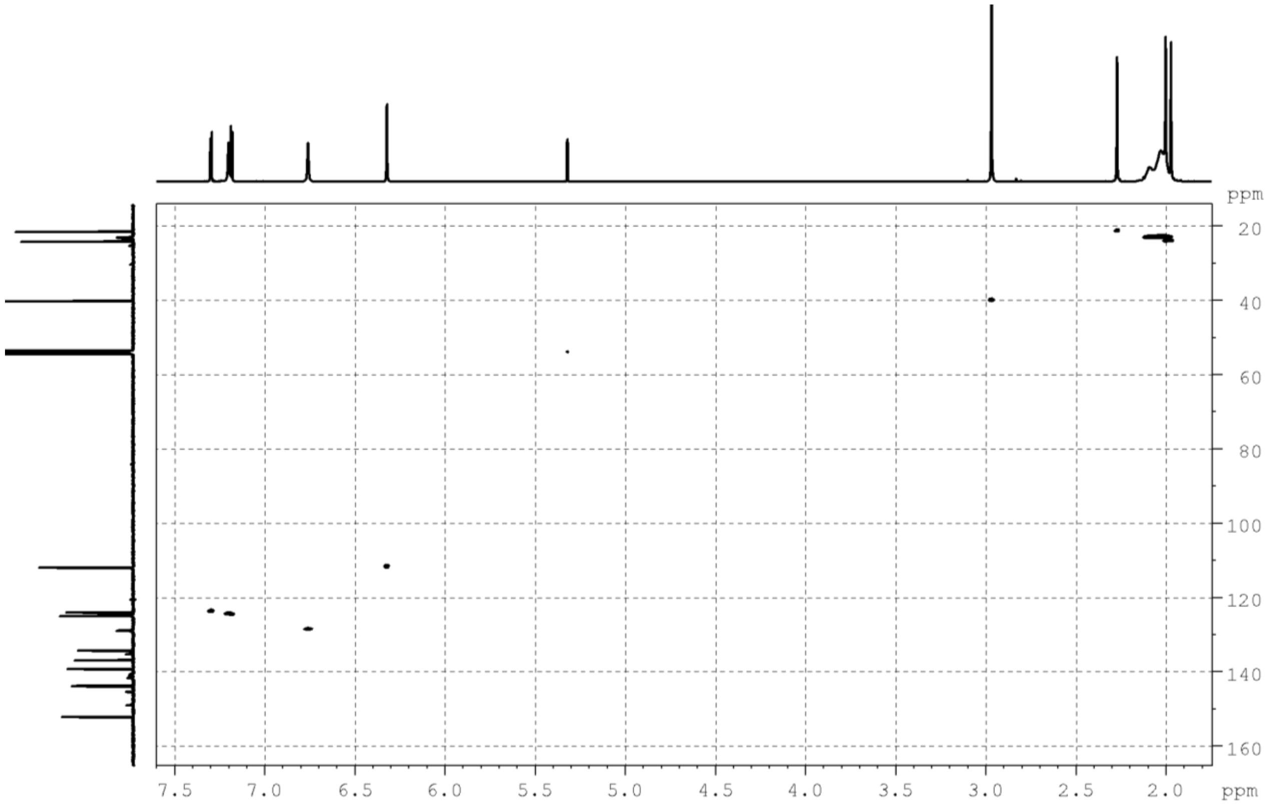

Figure S31: ^{1}H – ^{13}C HSQC spectrum of compound **Neut(i)2** recorded in CD_2Cl_2 at 500 MHz.

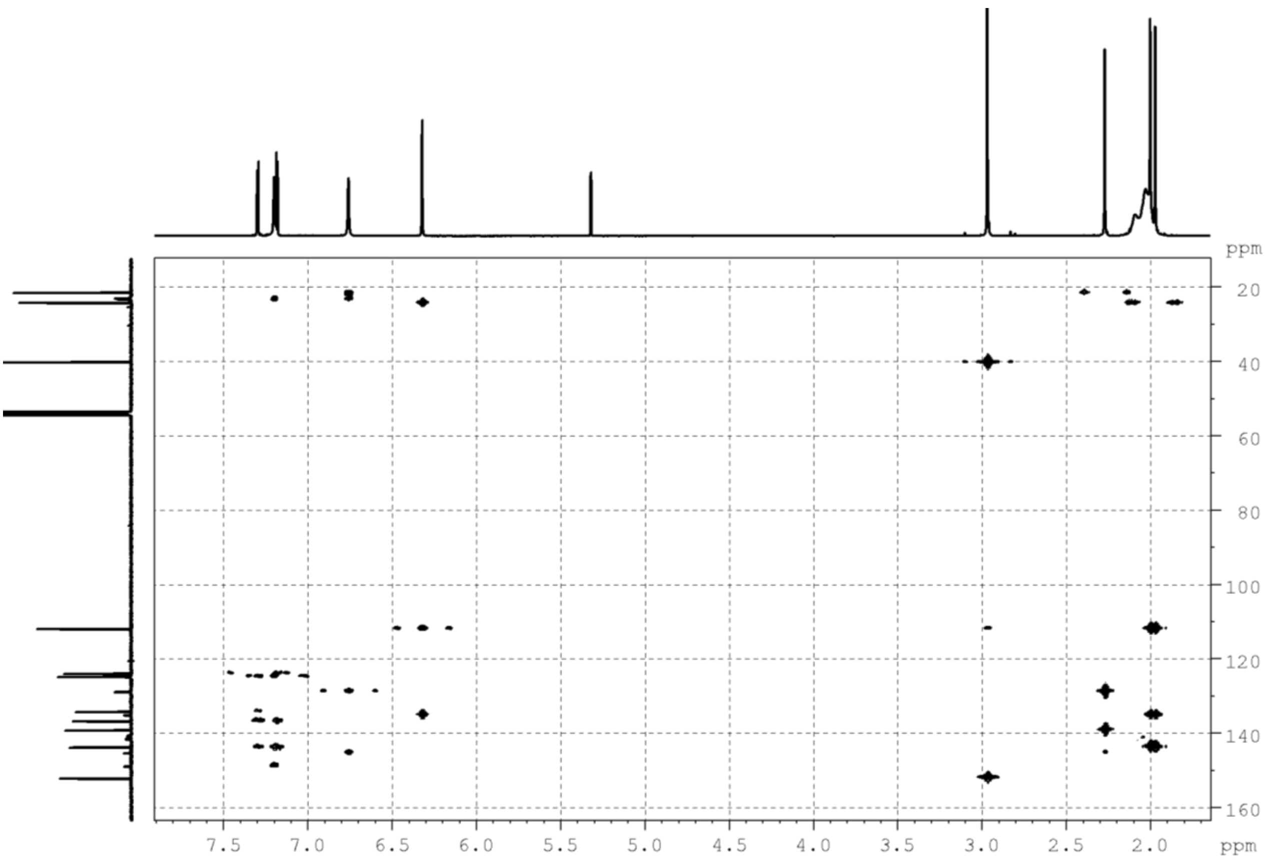

Figure S32: ^{1}H – ^{13}C HMBC spectrum of compound **Neut(i)2** recorded in CD_2Cl_2 at 500 MHz.

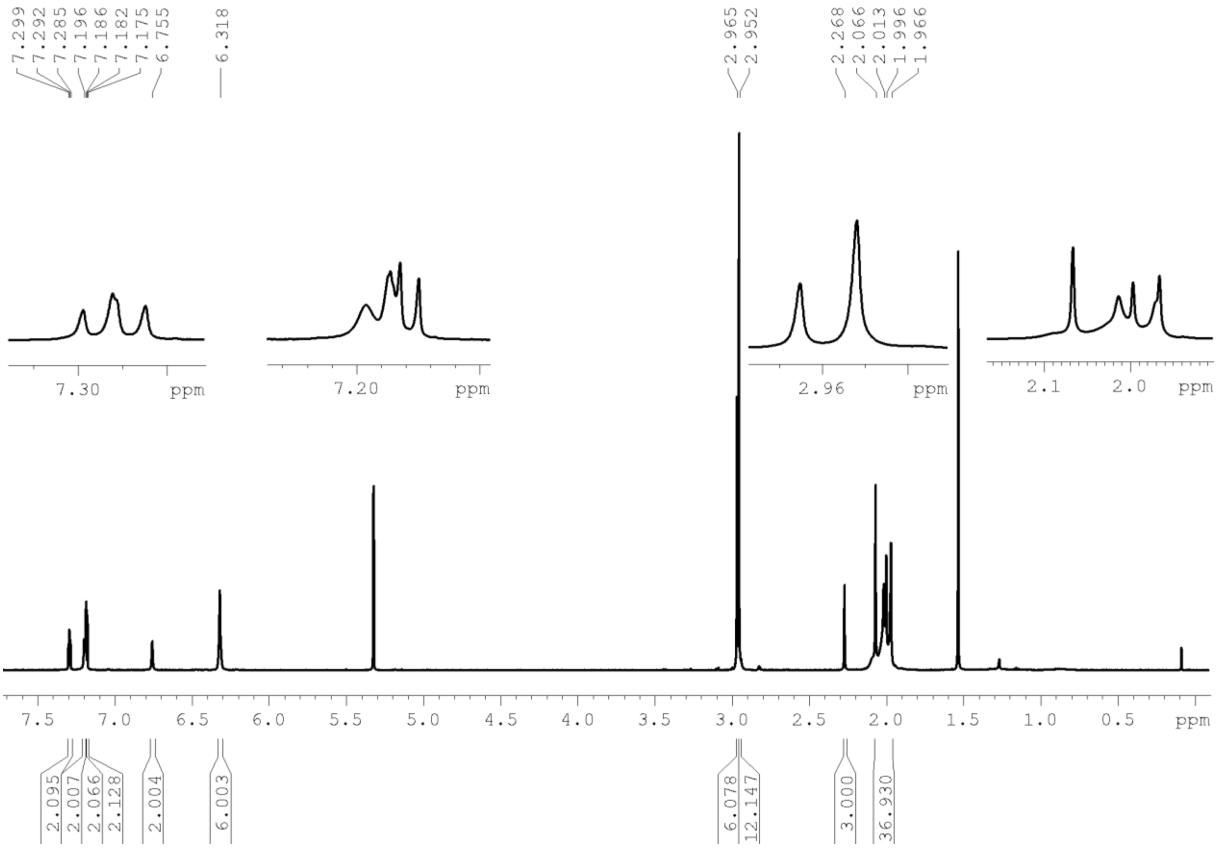

Figure S33: ^{1}H NMR spectrum of compound **Neut3** recorded in CD_2Cl_2 at 500 MHz.

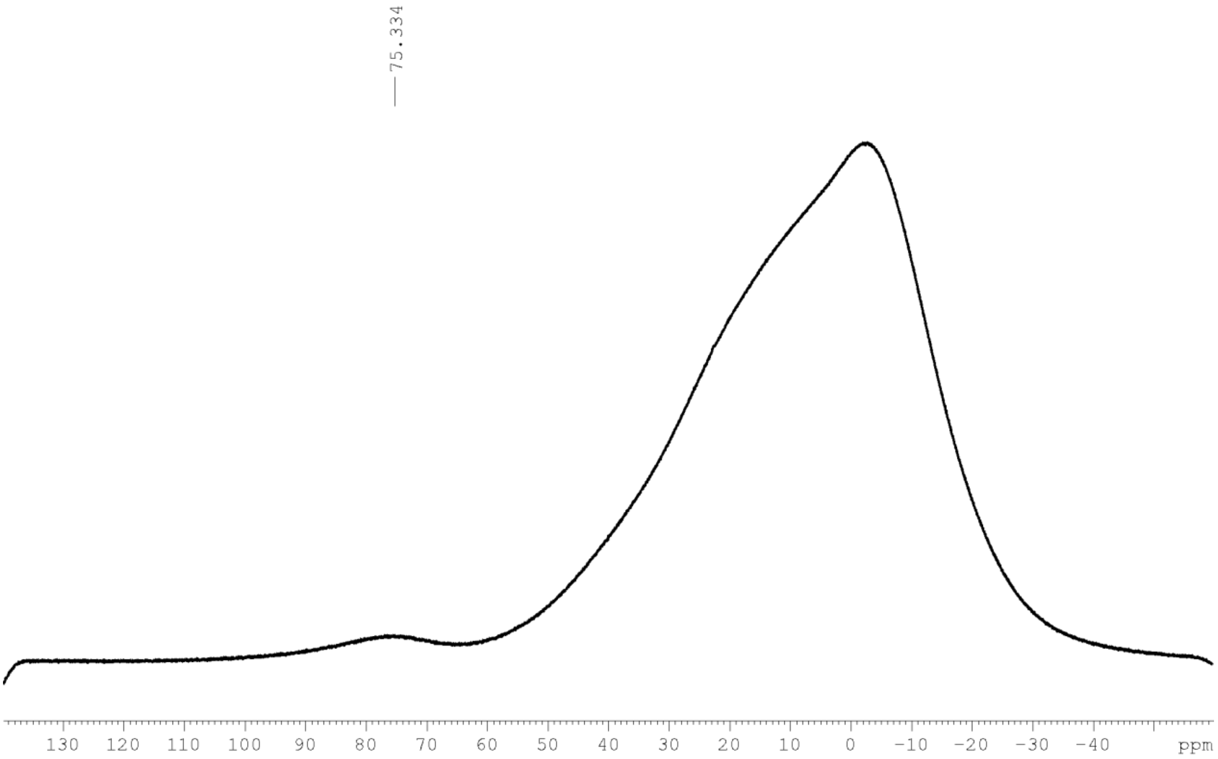

Figure S34: $^{11}B\{^{1}H\}$ NMR spectrum of compound **Neut3** recorded in CD_2Cl_2 at 160 MHz.

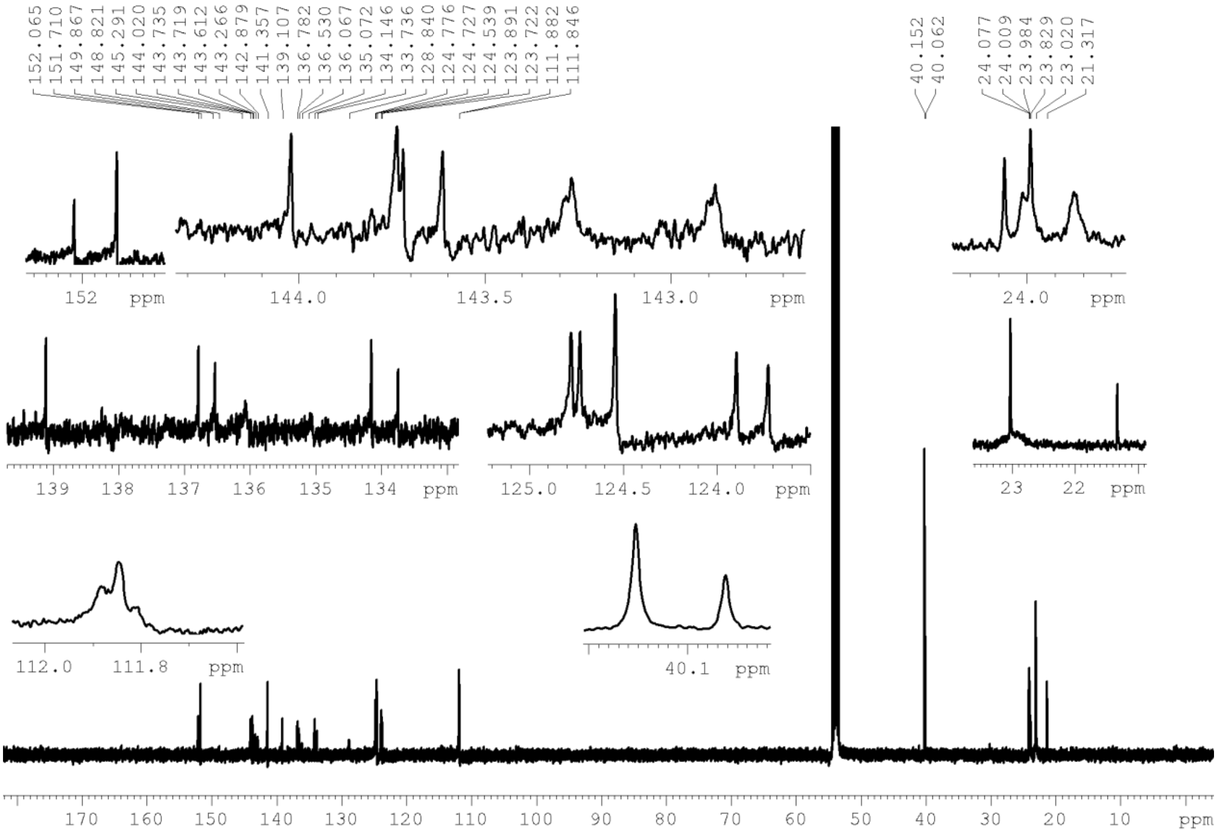

Figure S35: $^{13}C\{^{1}H\}$ NMR spectrum of compound **Neut3** recorded in CD_2Cl_2 at 125 MHz.

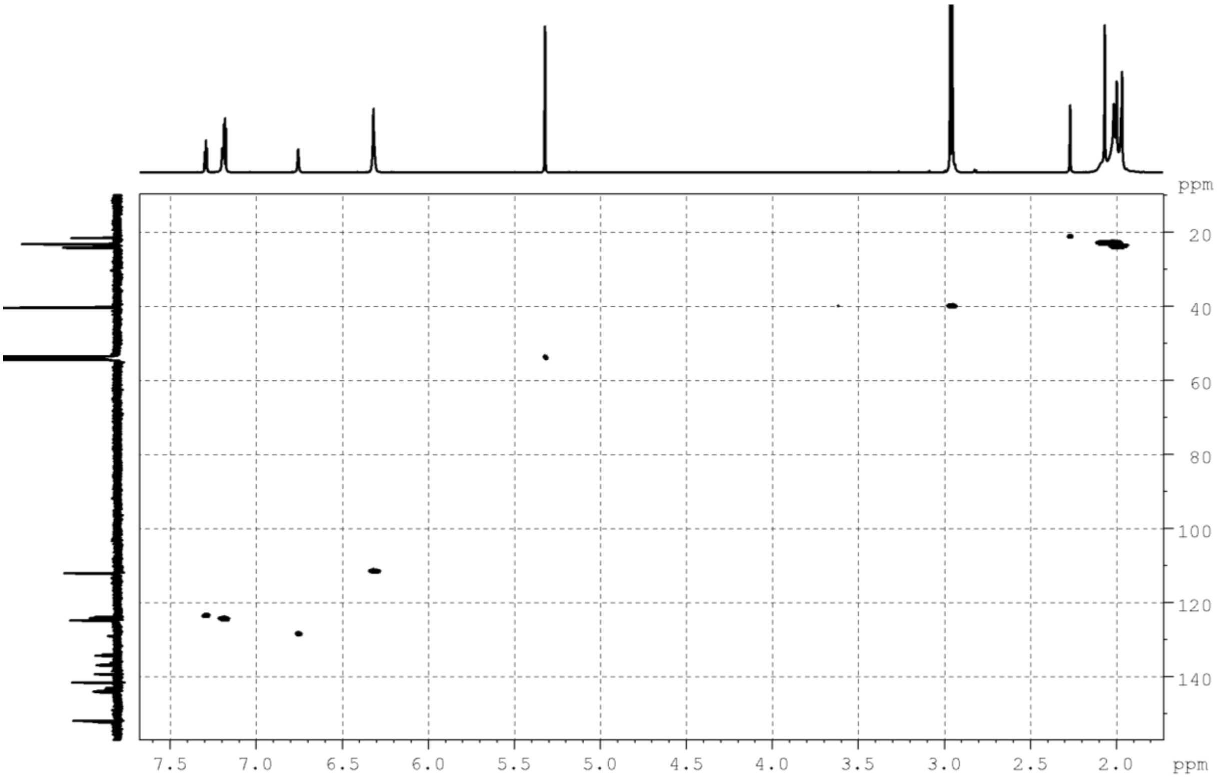

Figure S36: ^{1}H – ^{13}C HSQC spectrum of compound **Neut3** recorded in CD_2Cl_2 at 500 MHz.

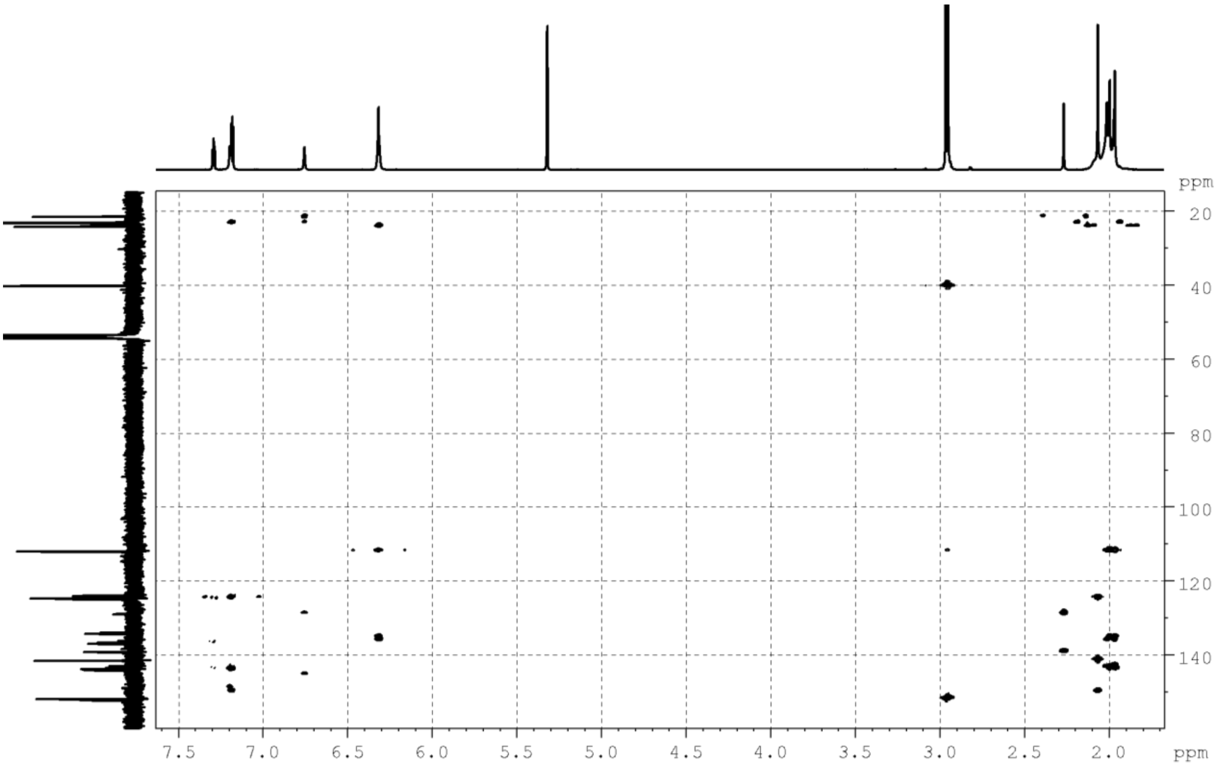

Figure S37: ^{1}H – ^{13}C HMBC spectrum of compound **Neut3** recorded in CD_2Cl_2 at 500 MHz.

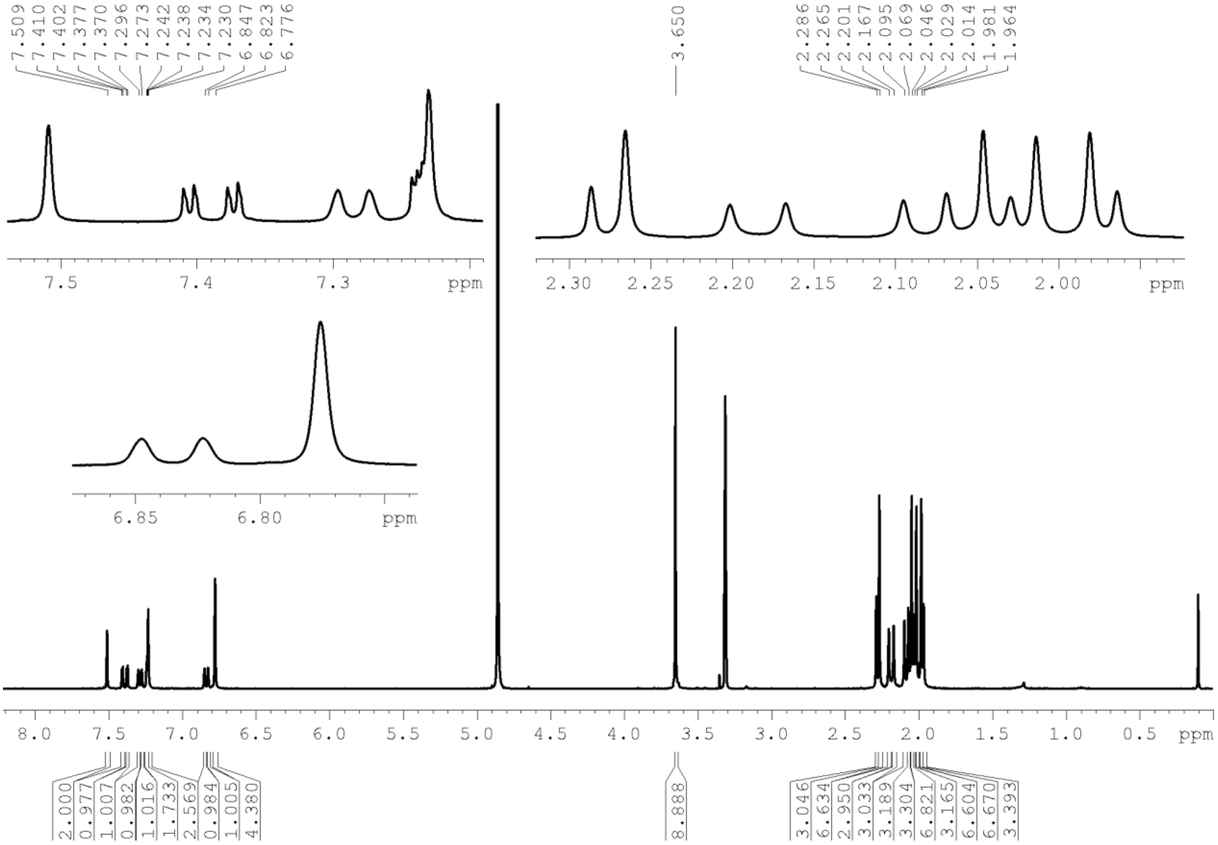

Figure S38: ^{1}H NMR spectrum of compound **Cat^{1+}** recorded in CD_3OD at 500 MHz.

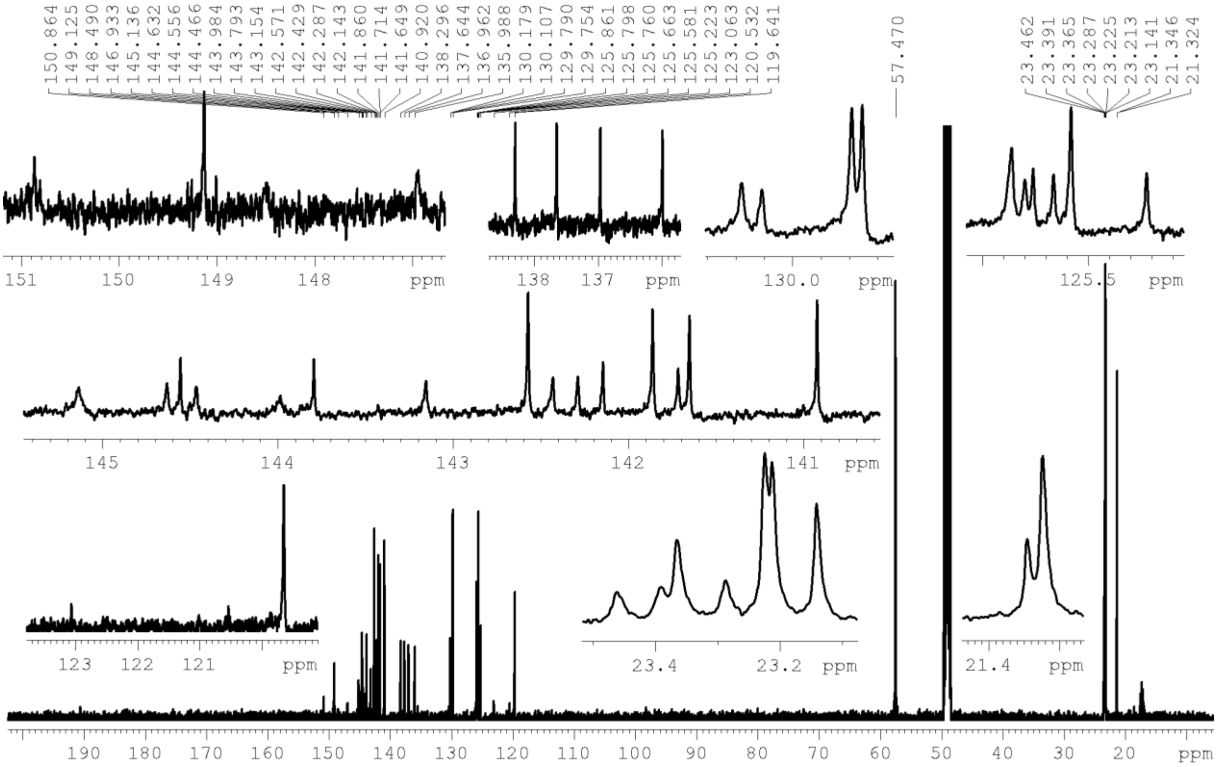

Figure S39: $^{13}C\{^{1}H\}$ NMR spectrum of compound **Cat^{1+}** recorded in CD_3OD at 125 MHz.

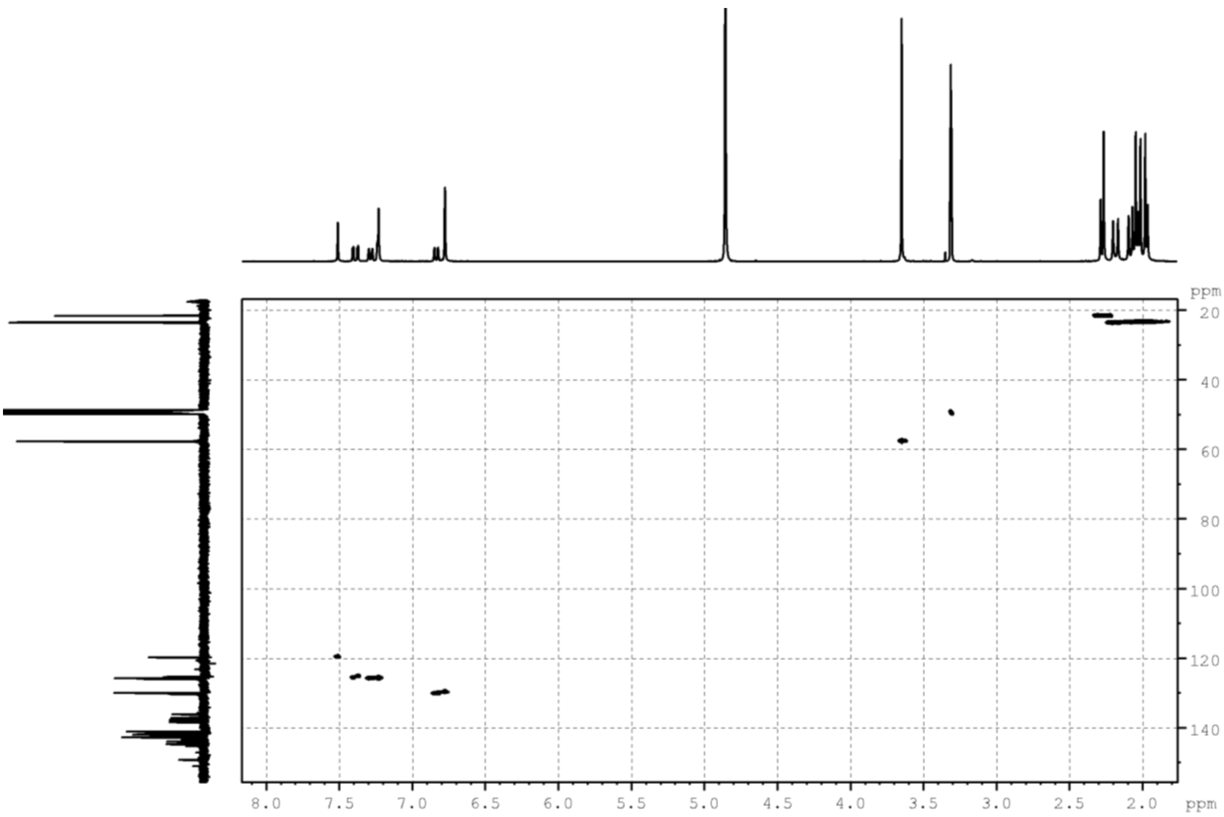

Figure S40: ^{1}H – ^{13}C HSQC spectrum of compound **Cat^{1+}** recorded in CD_3OD at 500 MHz.

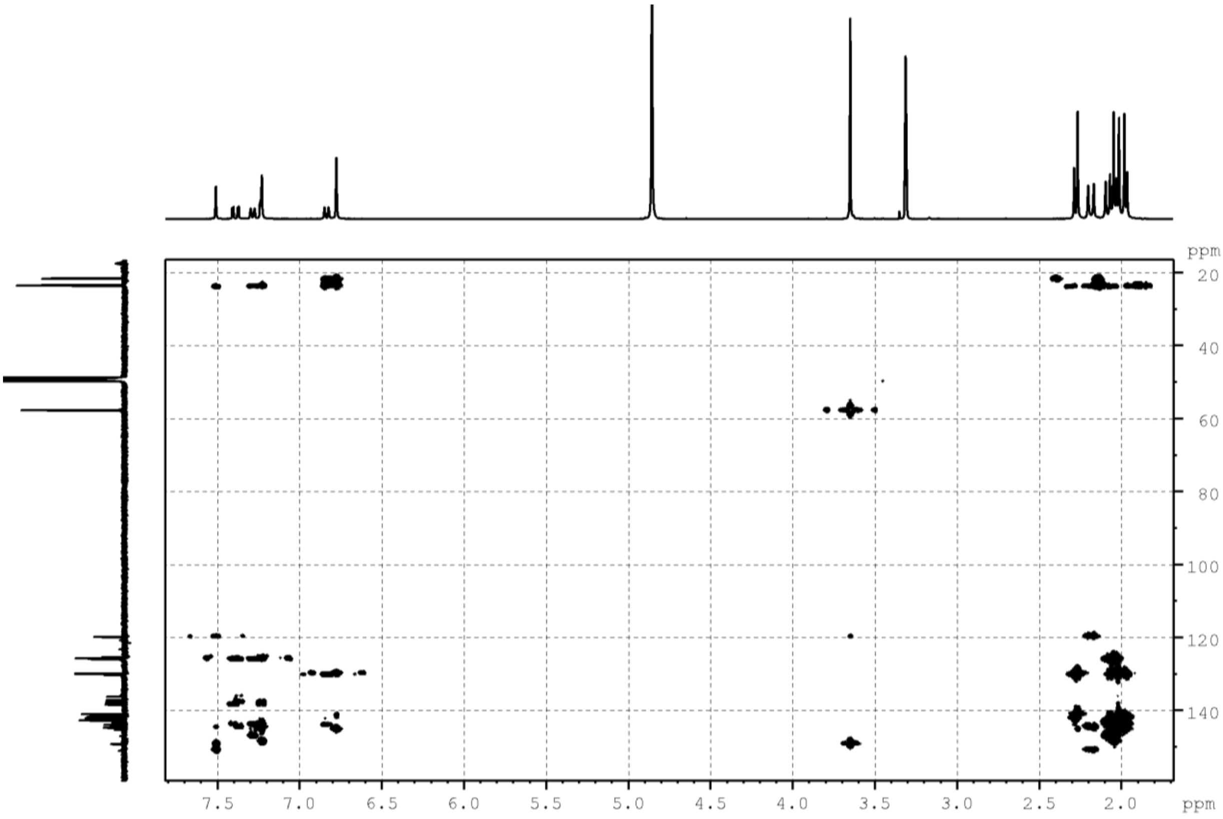

Figure S41: ^{1}H – ^{13}C HMBC spectrum of compound **Cat^{1+}** recorded in CD_3OD at 500 MHz.

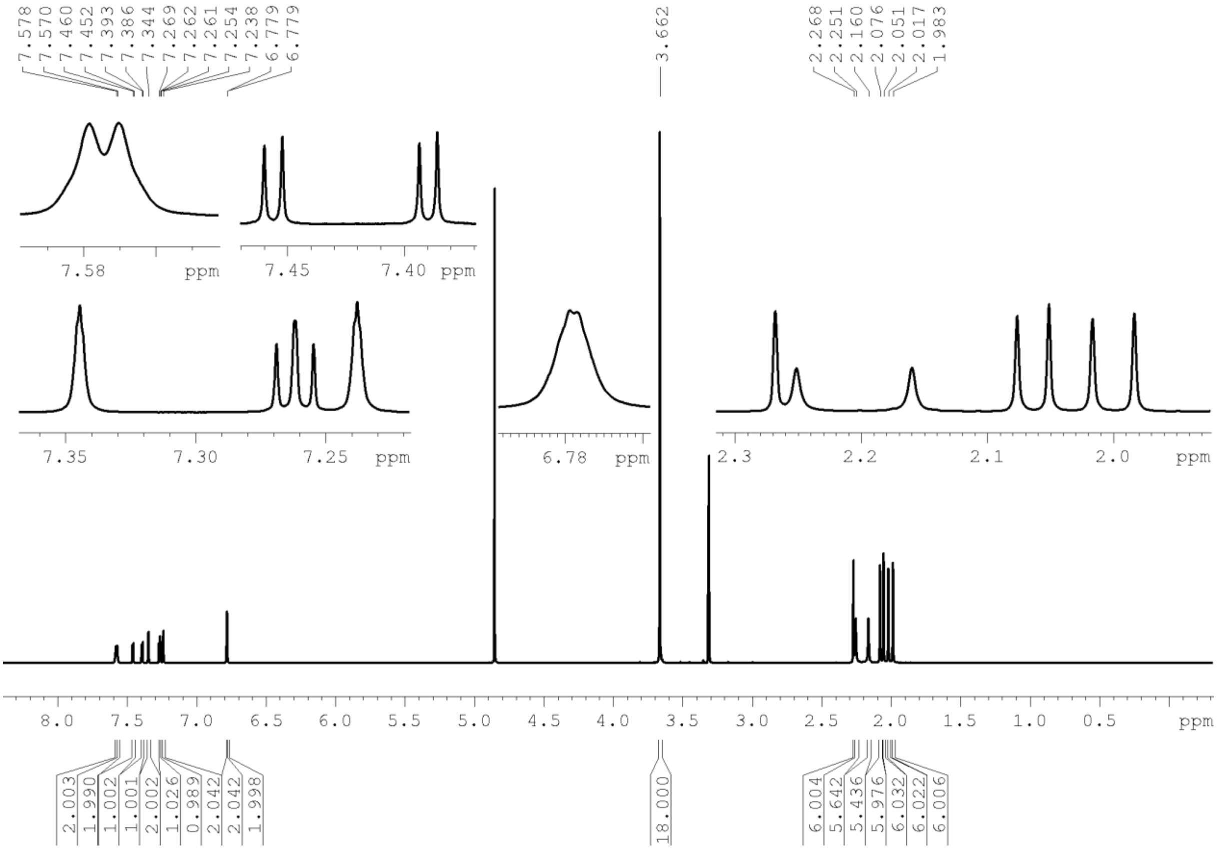

Figure S42: ^{1}H NMR spectrum of **Cat^{2+}** recorded in CD_3OD at 500 MHz.

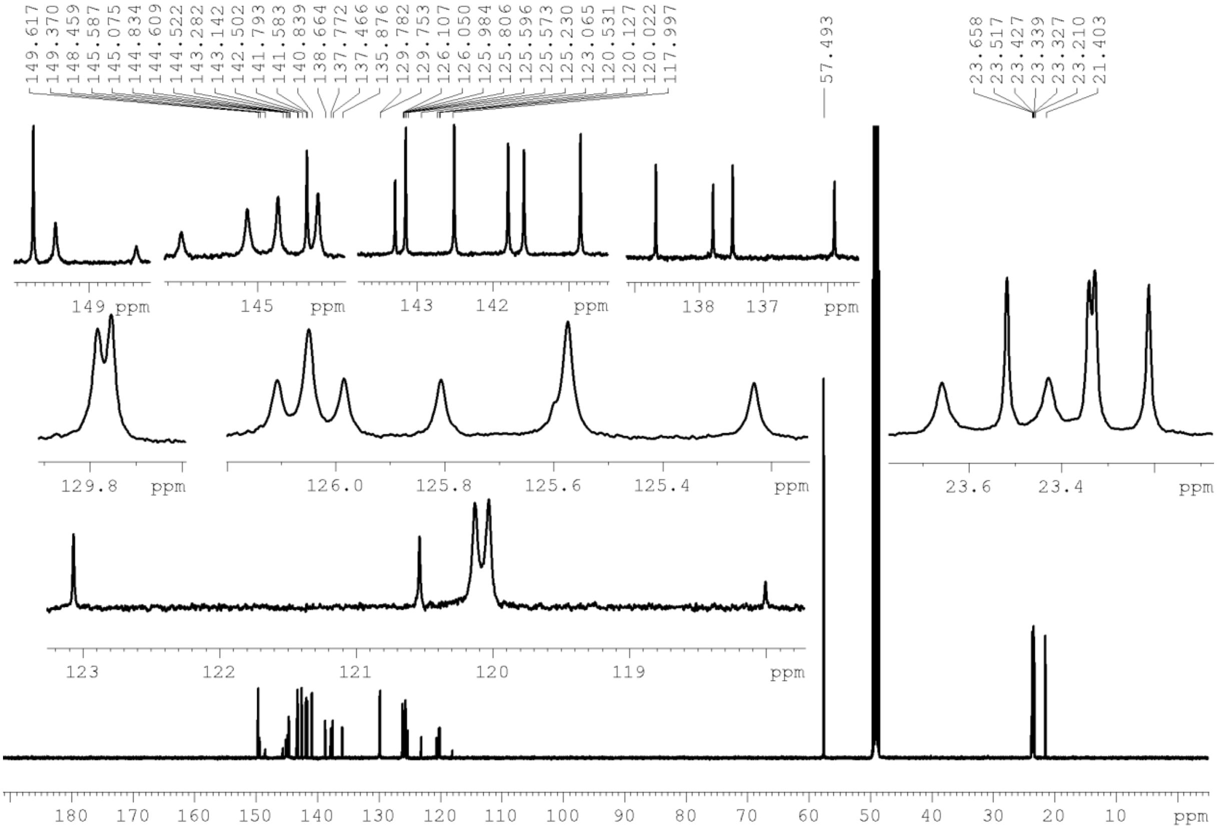

Figure S43: $^{13}C\{^{1}H\}$ NMR spectrum of **Cat^{2+}** recorded in CD_3OD at 125 MHz.

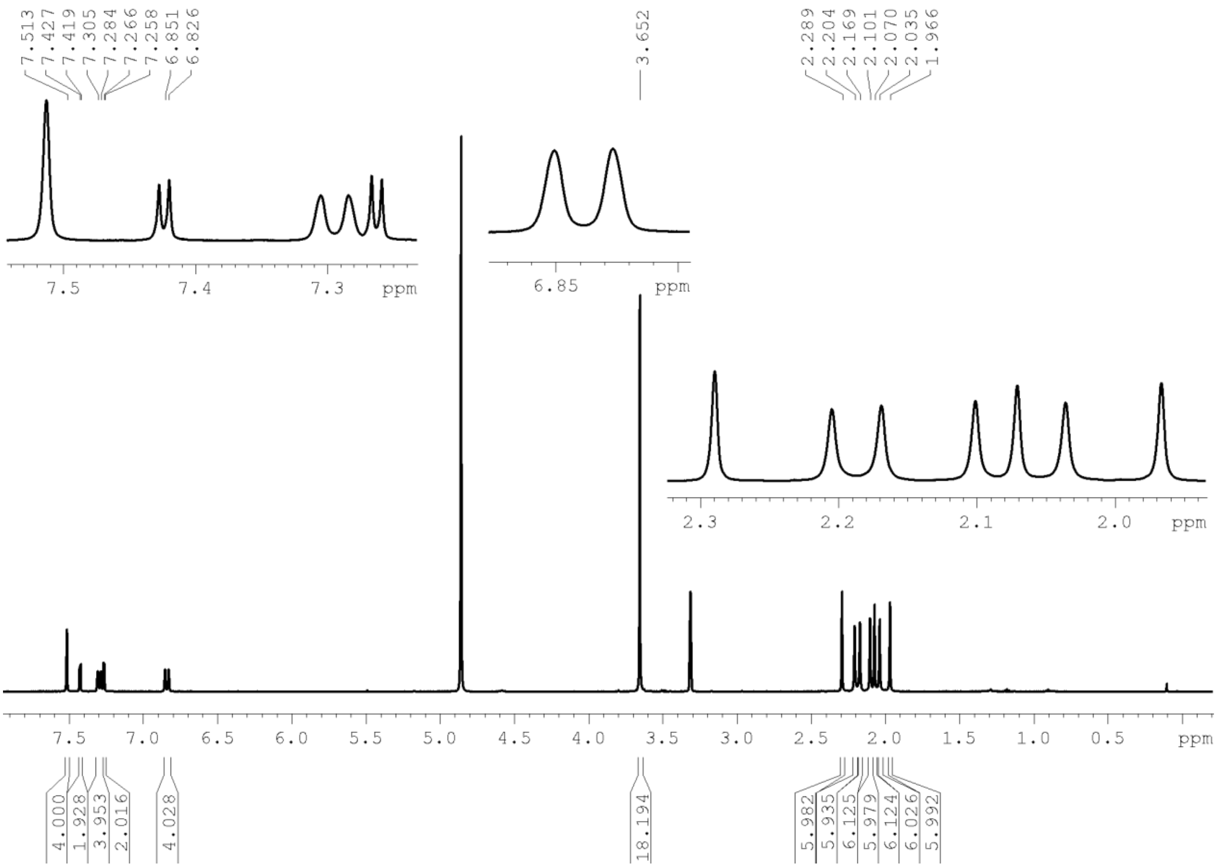

Figure S44: ^{1}H NMR spectrum of **Cat(i)$^{2+}$** recorded in CD_3OD at 500 MHz.

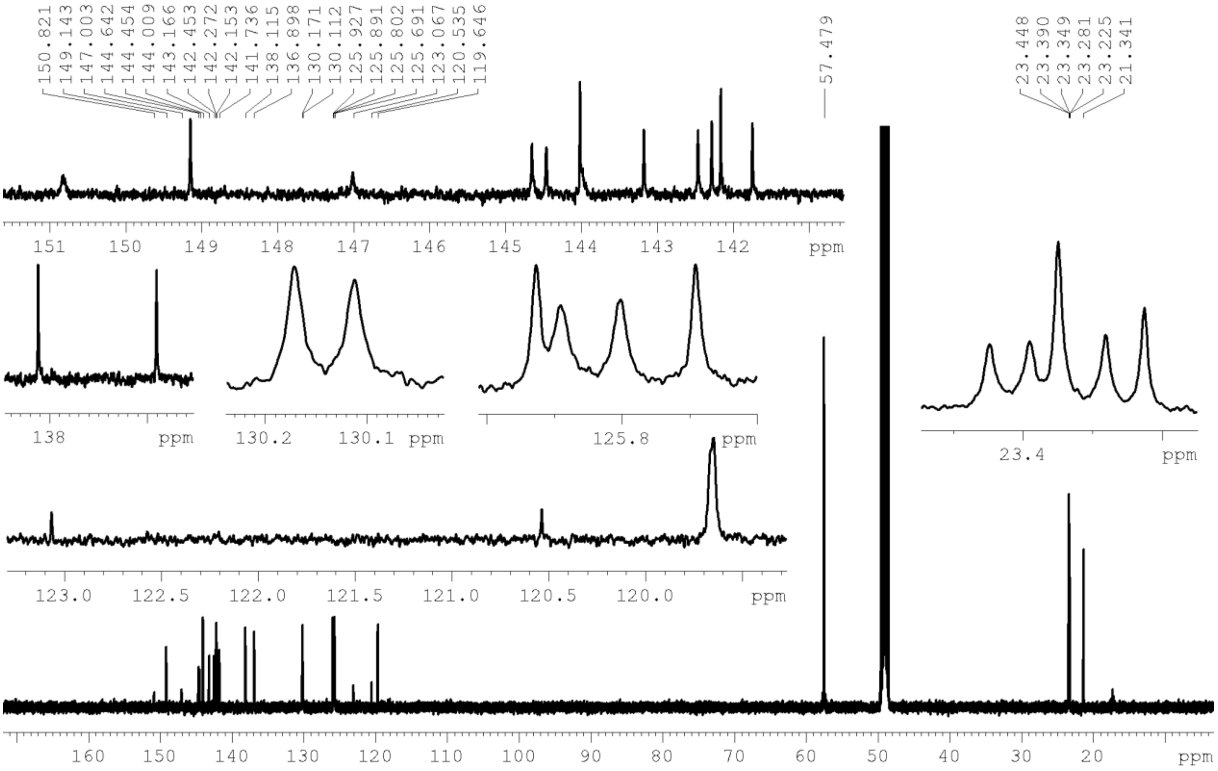

Figure S45: $^{13}C\{^{1}H\}$ NMR spectrum of **Cat(i)$^{2+}$** recorded in CD_3OD at 125 MHz.

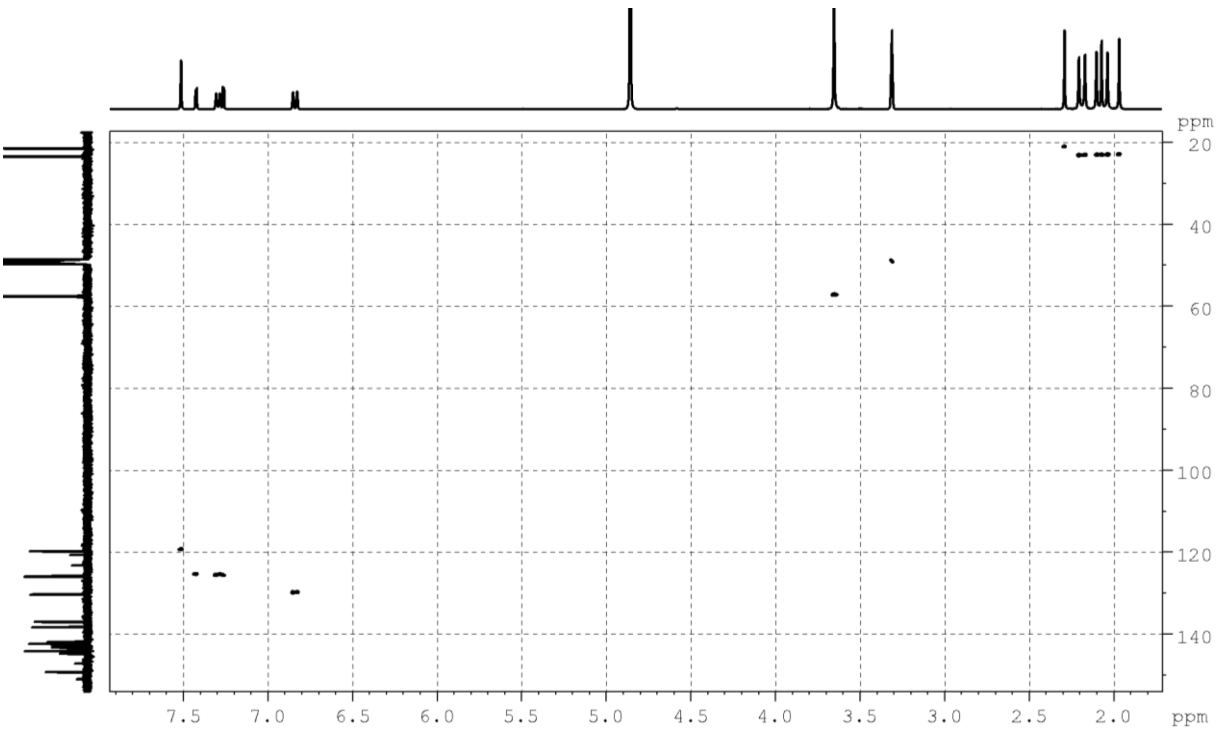

Figure S46: ^{1}H – ^{13}C HSQC spectrum of compound **Cat(i)$^{2+}$** recorded in CD_3OD at 500 MHz.

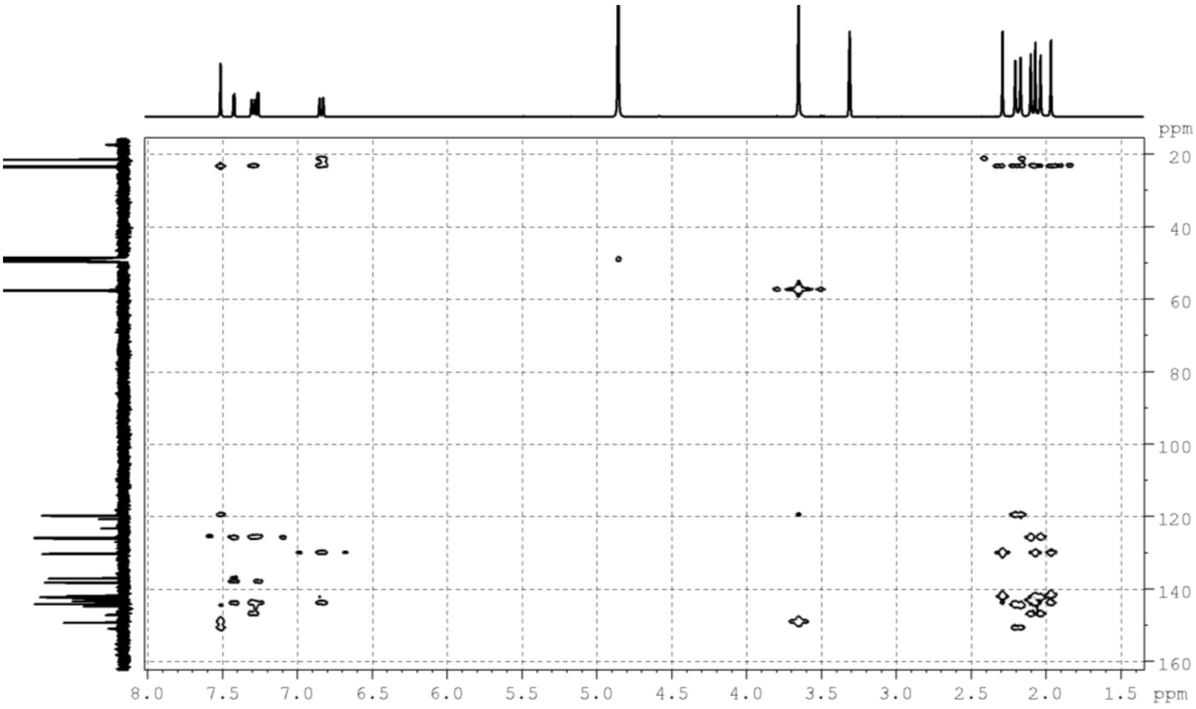

Figure S47: ^{1}H – ^{13}C HMBC spectrum of compound **Cat(i)$^{2+}$** recorded in CD_3OD at 500 MHz.

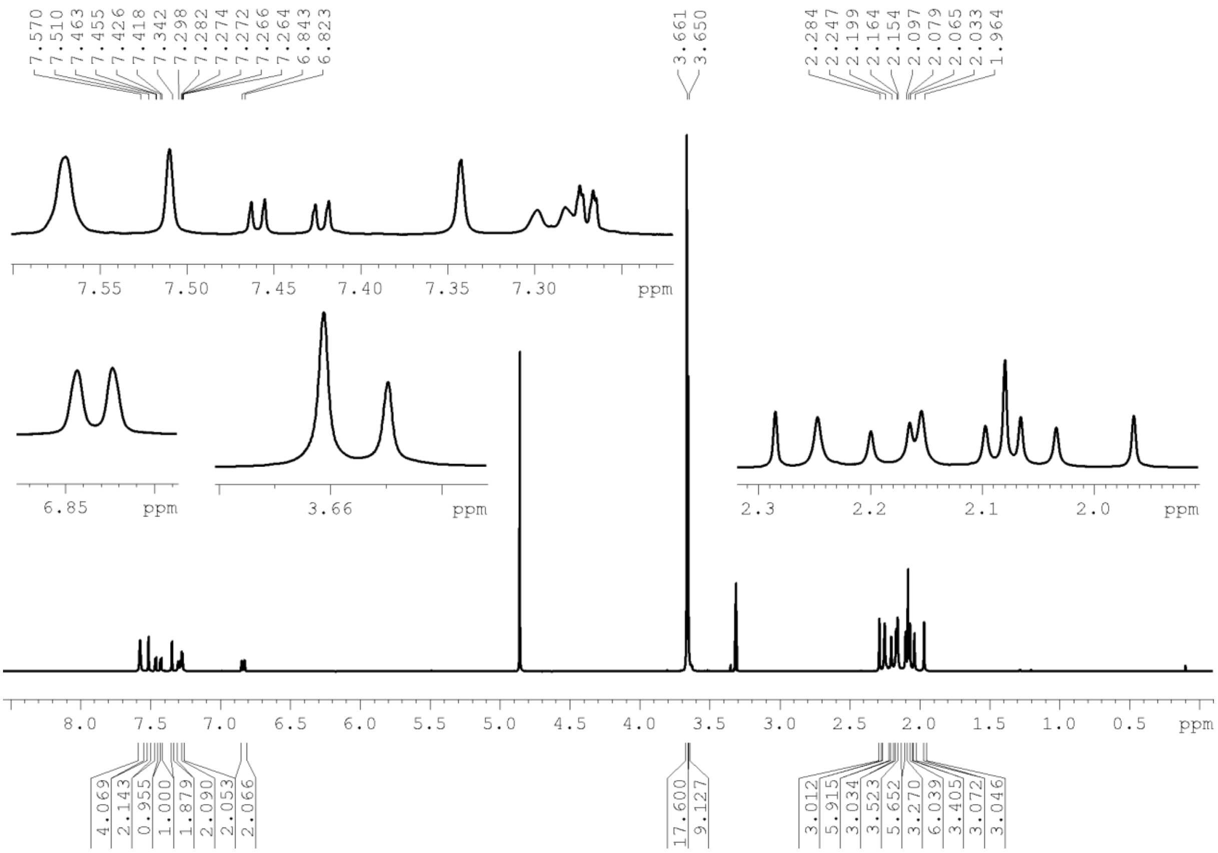

Figure S48: ^{1}H NMR spectrum of **Cat^{3+}** recorded in CD_3OD at 500 MHz.

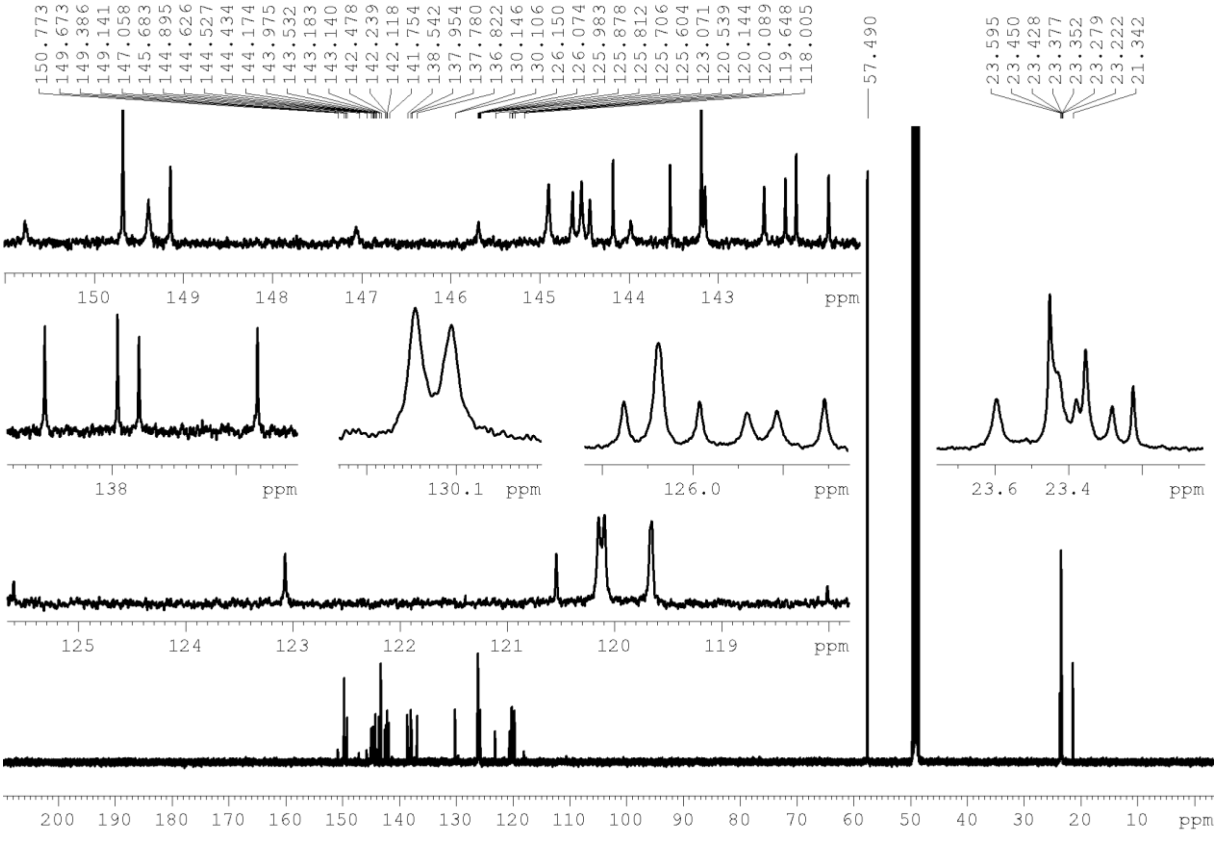

Figure S49: $^{13}C\{^{1}H\}$ NMR spectrum of **Cat^{3+}** recorded in CD_3OD at 125 MHz.

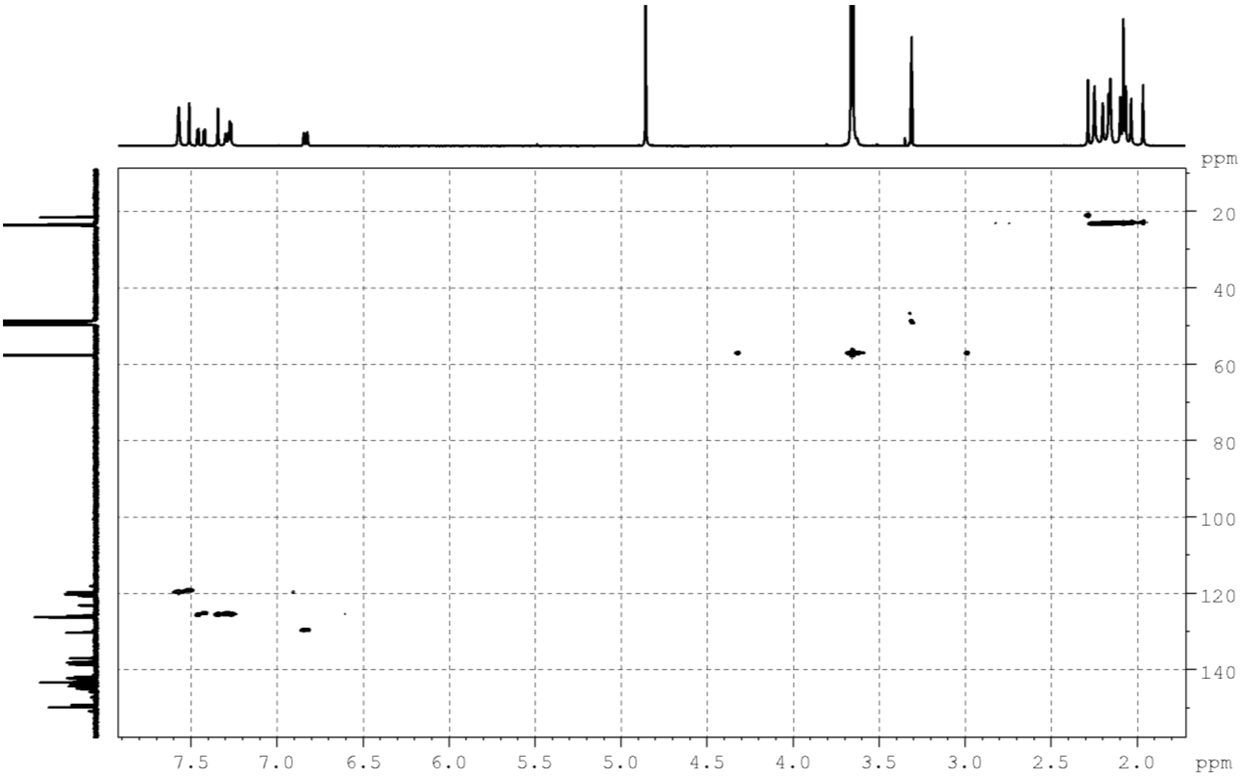

Figure S50: ^{1}H – ^{13}C HSQC spectrum of compound **Cat^{3+}** recorded in CD_3OD at 500 MHz.

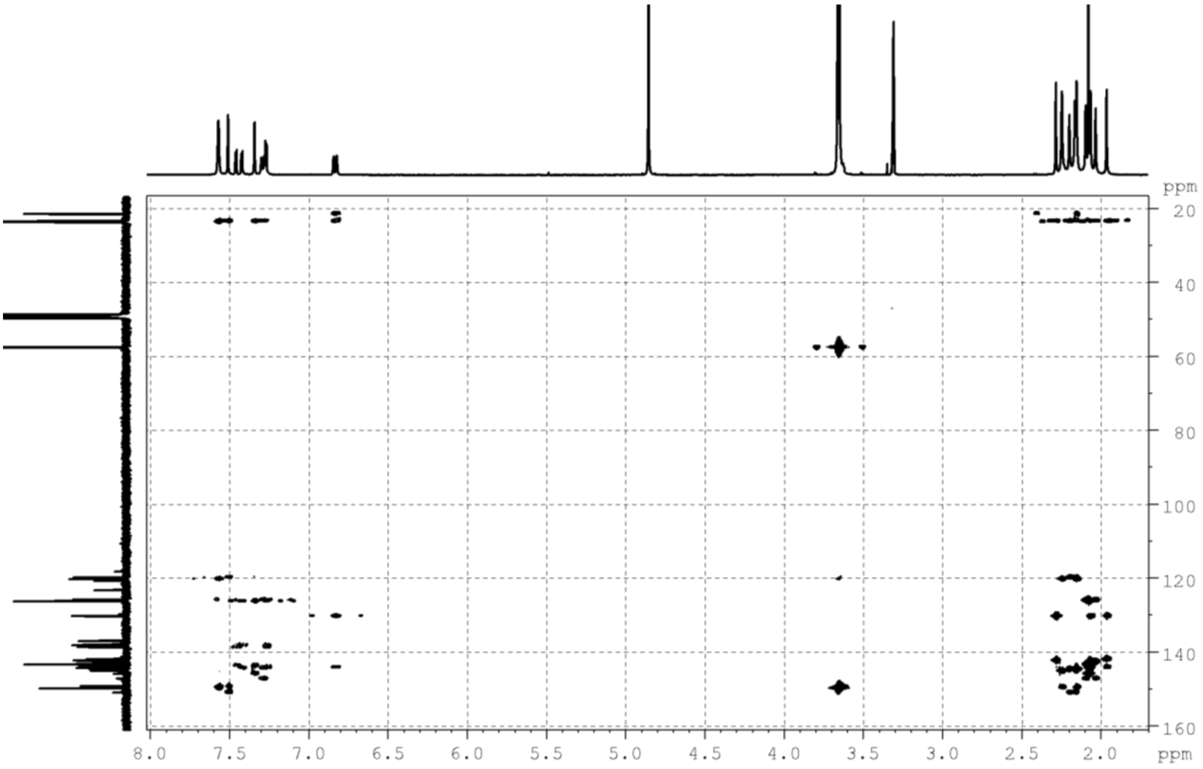

Figure S51: ^{1}H – ^{13}C HMBC spectrum of compound **Cat^{3+}** recorded in CD_3OD at 500 MHz.

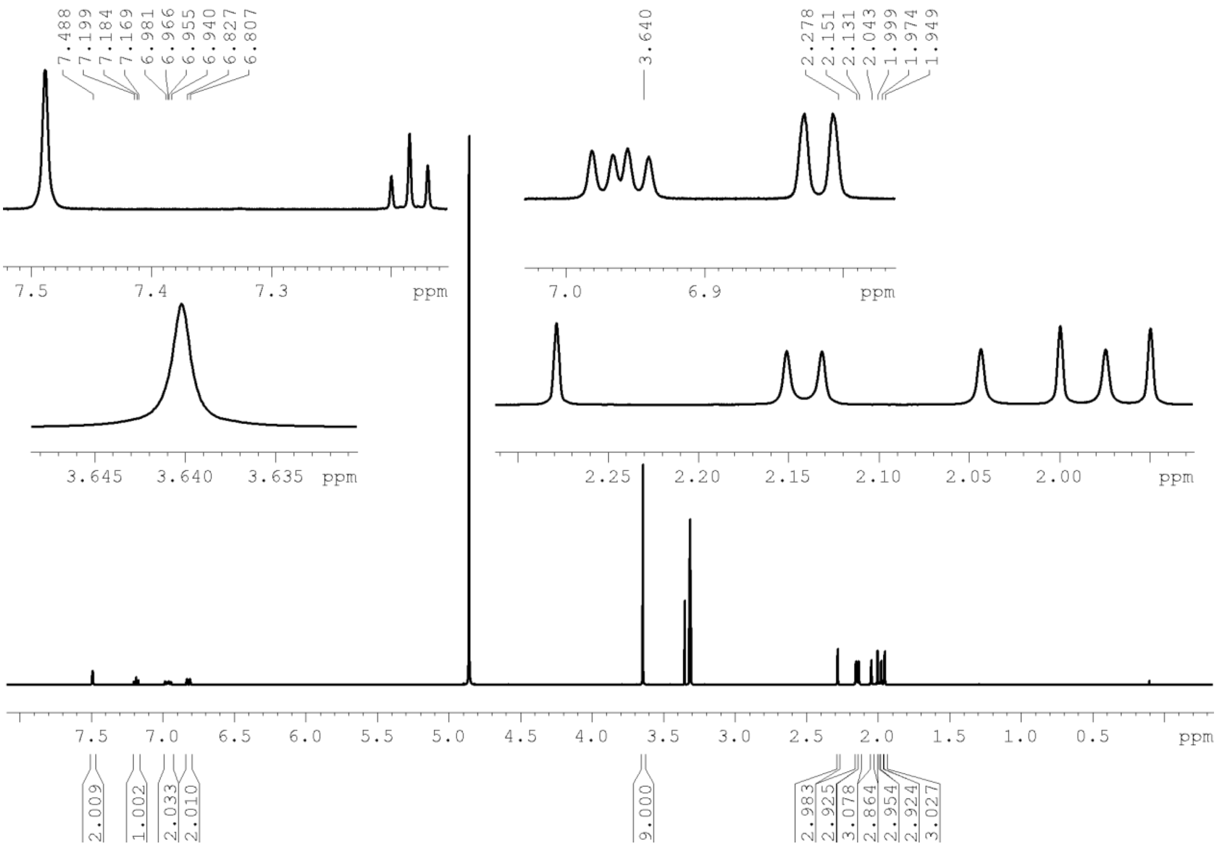

Figure S52: ^{1}H NMR spectrum of compound **5c** recorded in CD_3OD at 500 MHz.

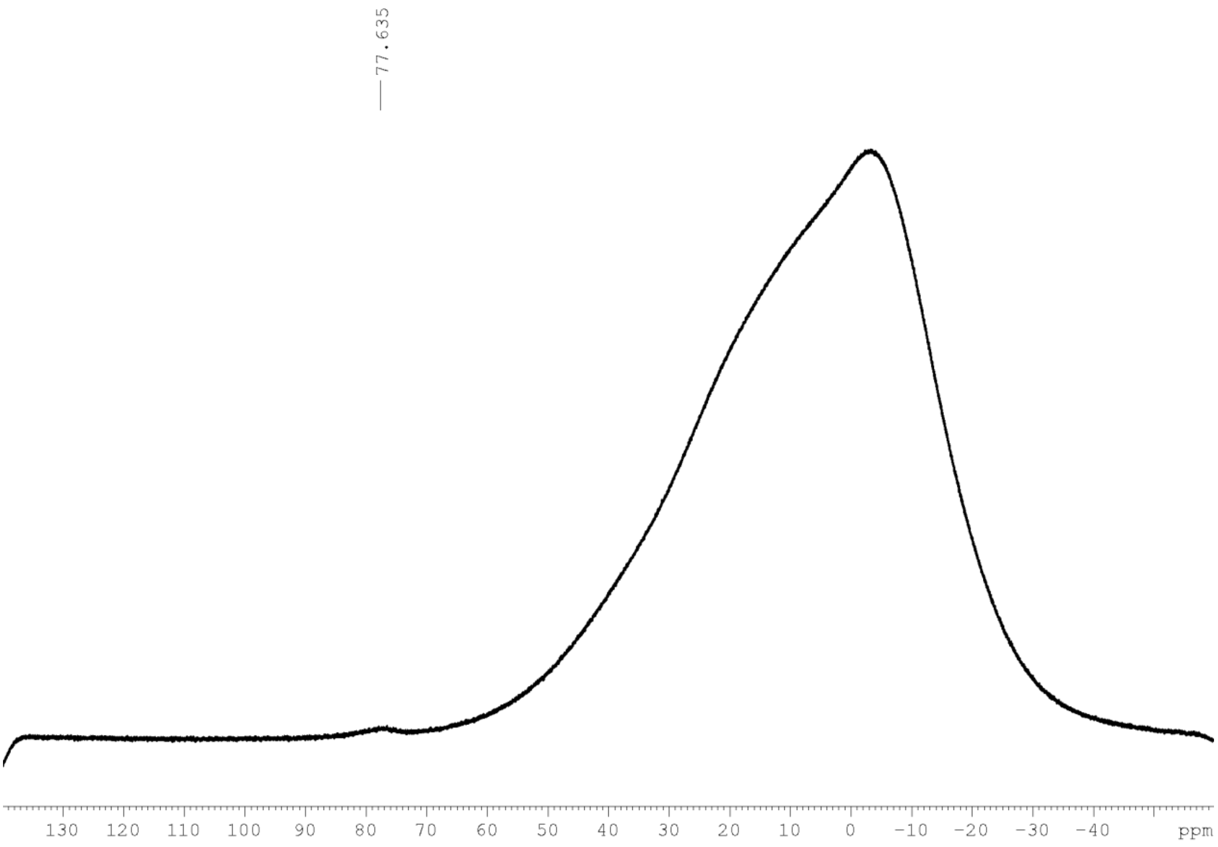

Figure S53: ^{11}B{^{1}H} NMR spectrum of compound **5c** recorded in CD_3OD at 160 MHz.

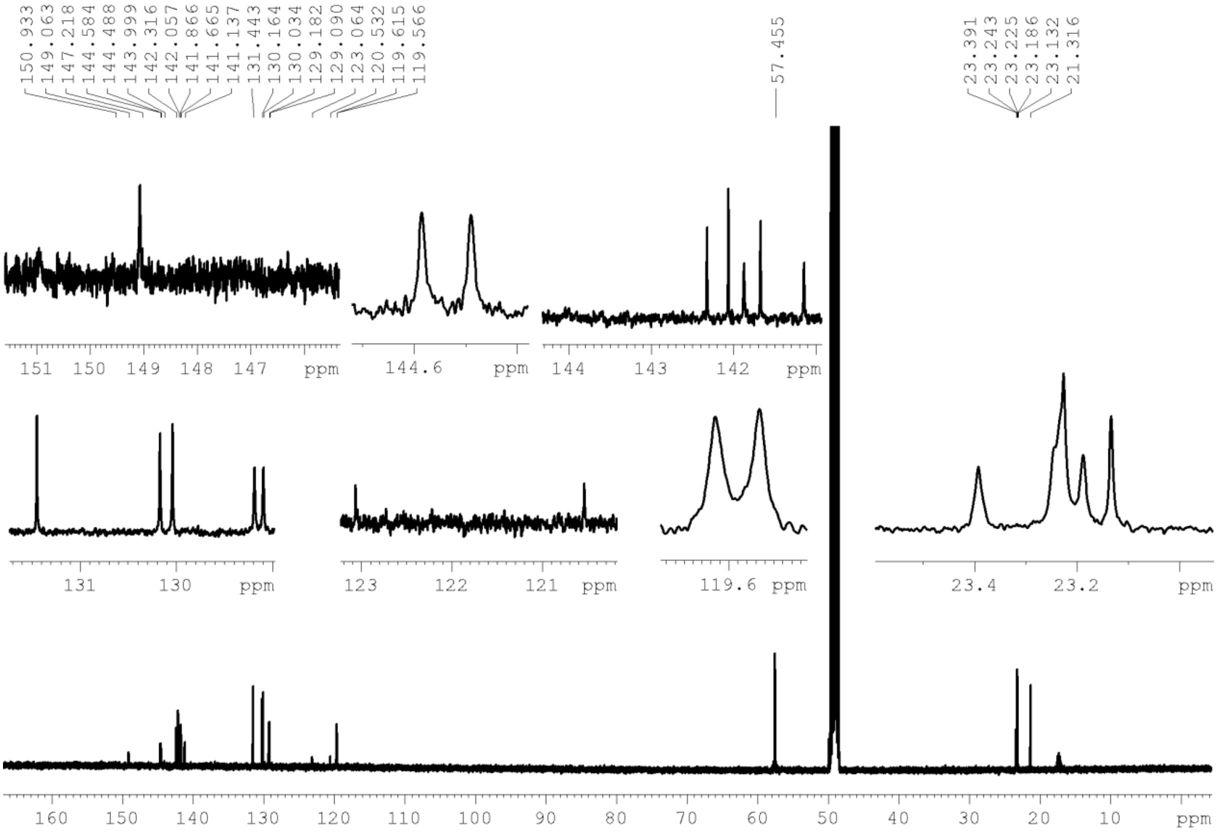

Figure S54: $^{13}C\{^{1}H\}$ NMR spectrum of compound **5c** recorded in CD_3OD at 125 MHz.

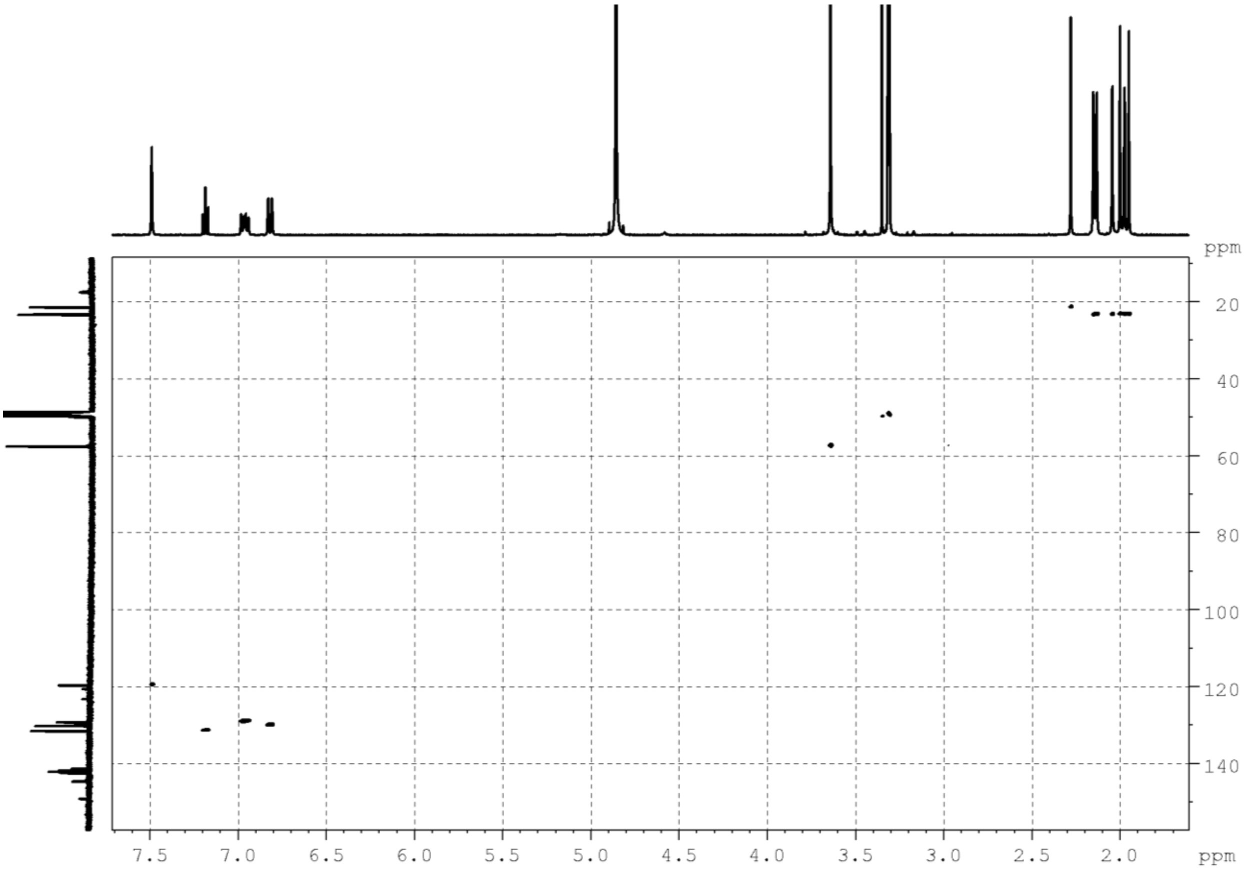

Figure S55: ^{1}H – ^{13}C HSQC spectrum of compound **5c** recorded in CD_3OD at 500 MHz.

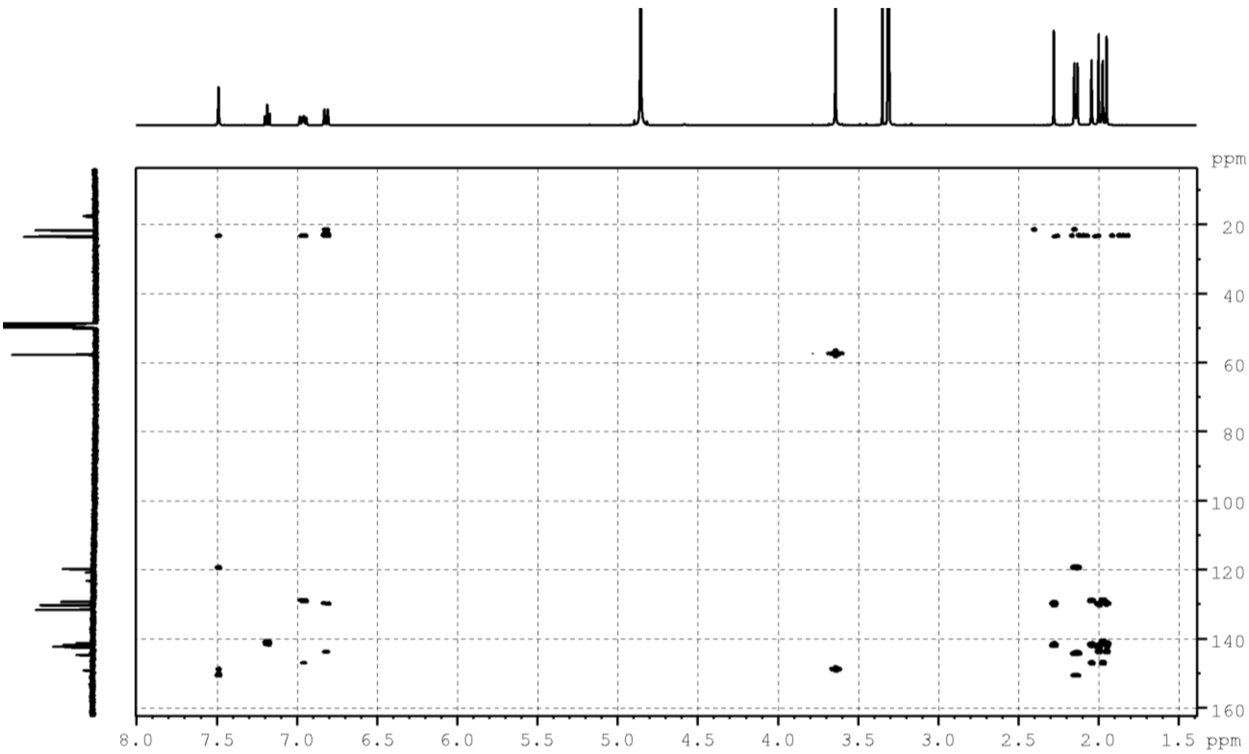

Figure S56: ^{1}H – ^{13}C HMBC spectrum of compound **5c** recorded in CD_3OD at 500 MHz.

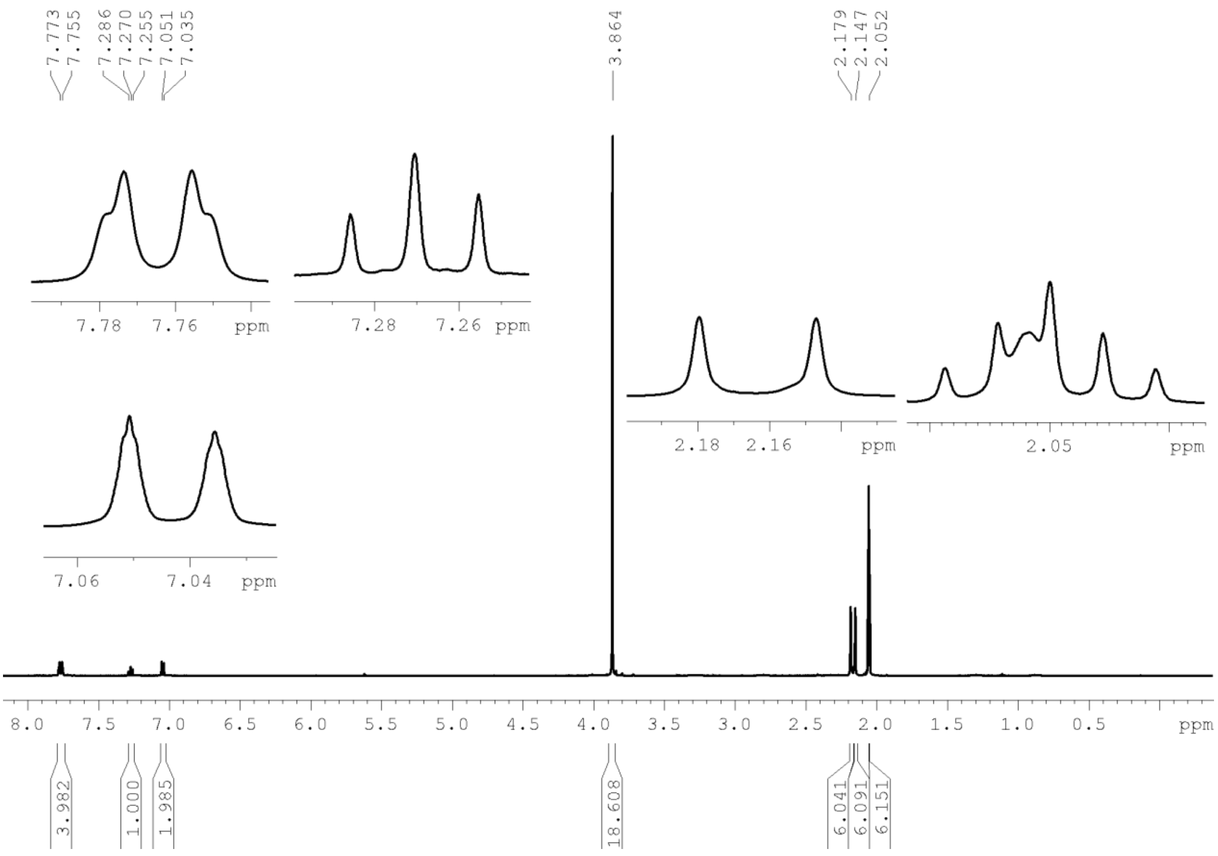

Figure S57: ^{1}H NMR spectrum of compound **6c** recorded in acetone-d_6 at 500 MHz.

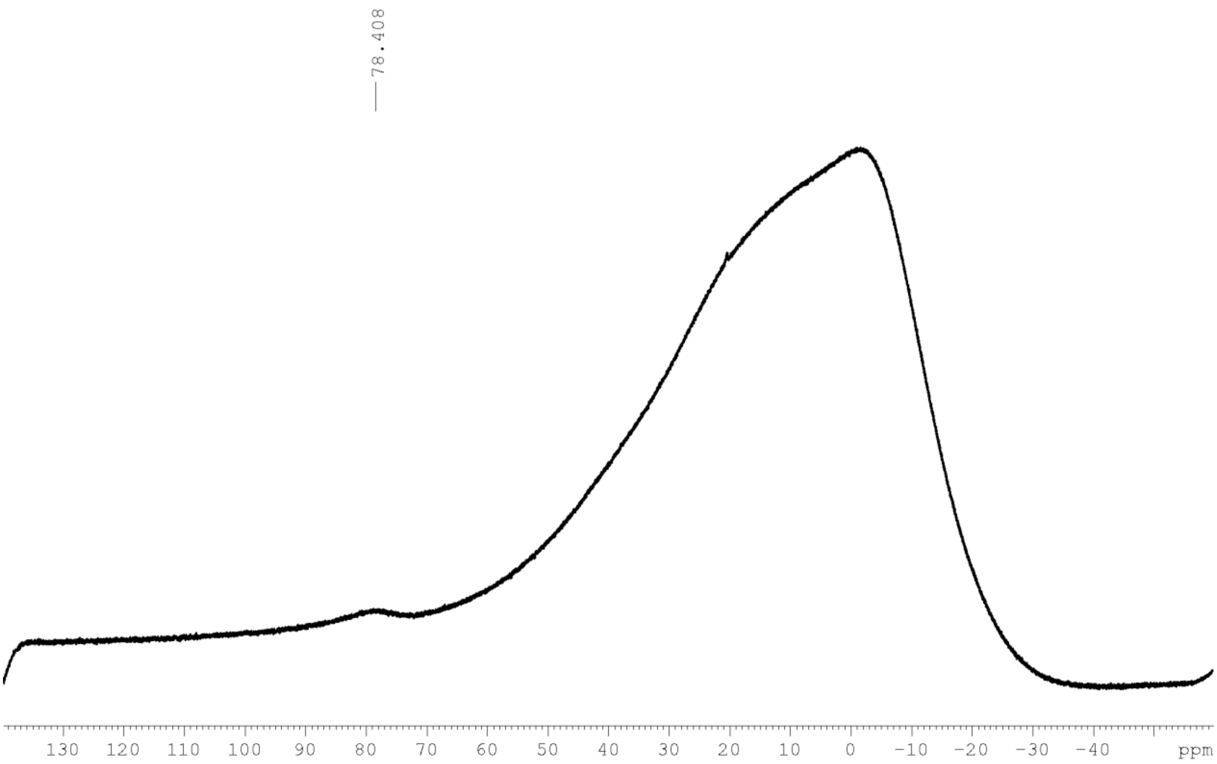

Figure S58: $^{11}B\{^1H\}$ NMR spectrum of compound **6c** recorded in acetone-d_6 at 160 MHz.

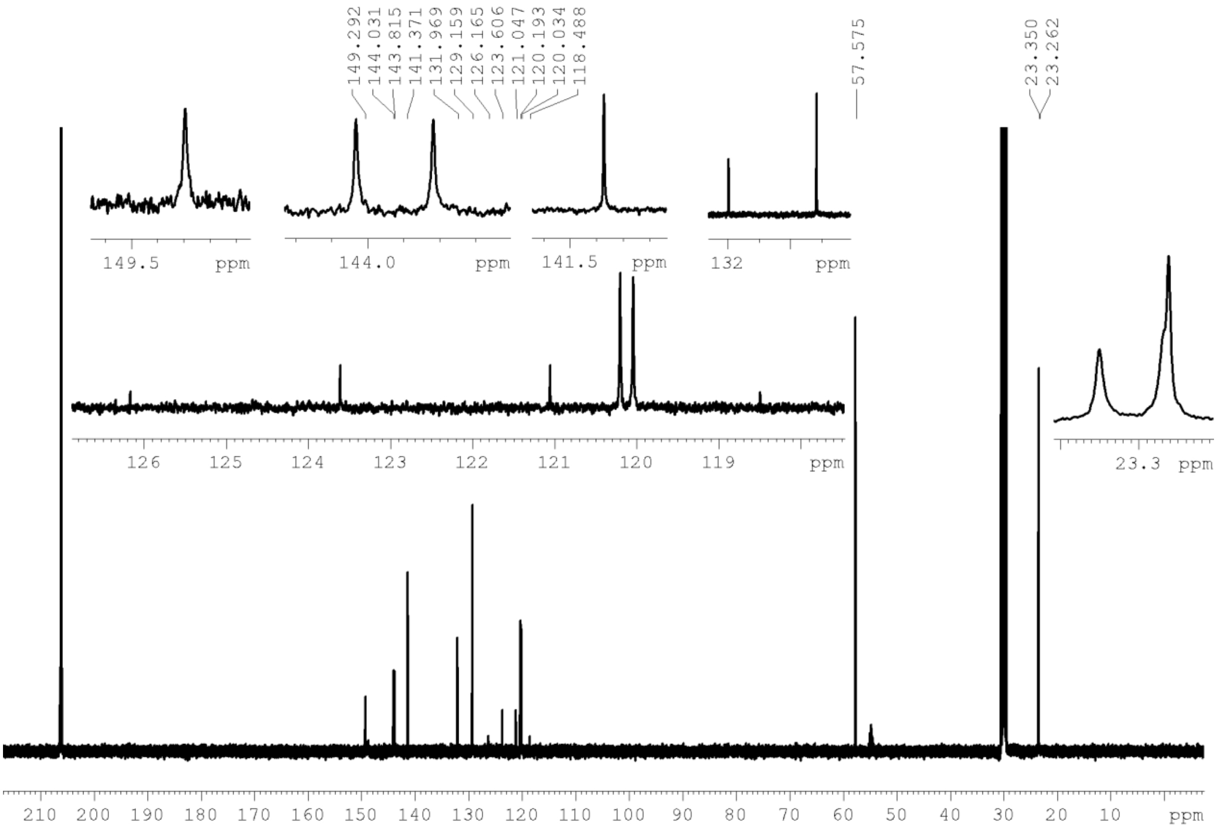

Figure S59: $^{13}C\{^1H\}$ NMR spectrum of compound **6c** recorded in acetone-d_6 at 125 MHz.

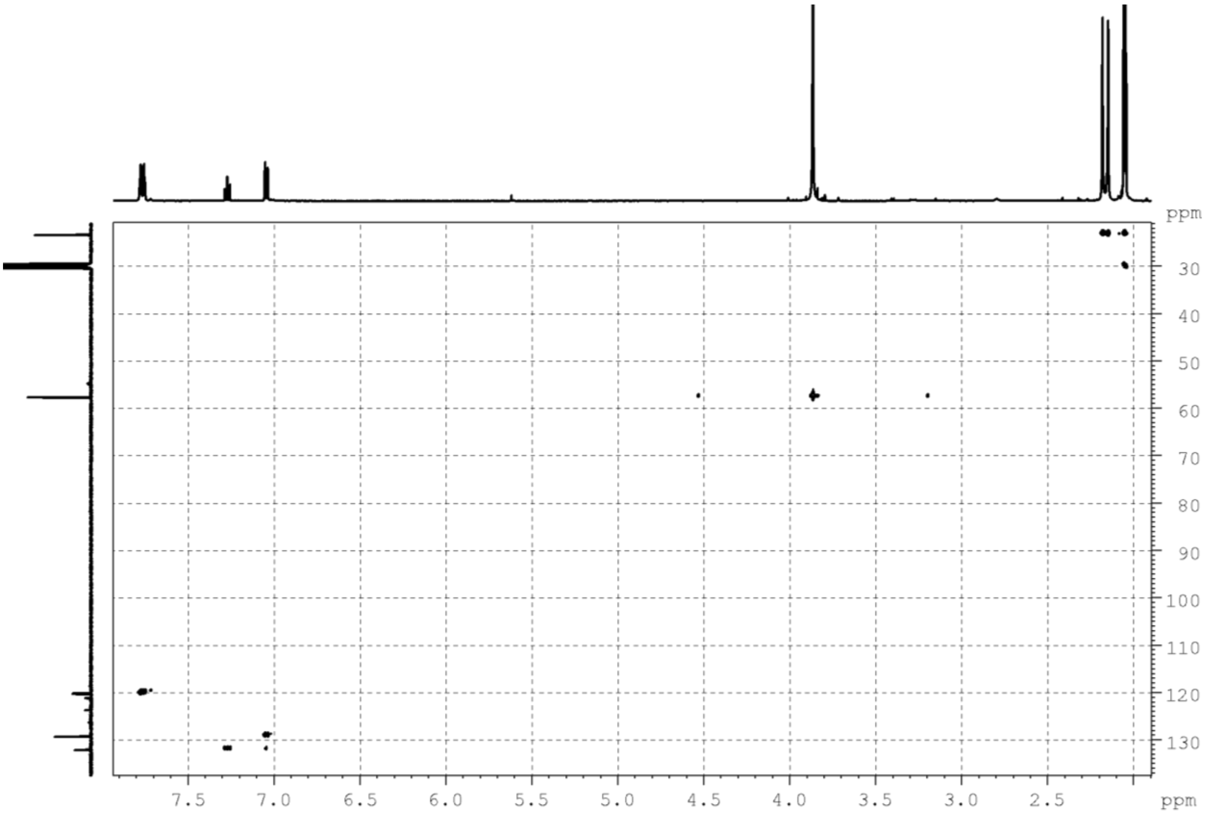

Figure S60: ^{1}H – ^{13}C HSQC spectrum of compound **6c** recorded in acetone-d_6 at 500 MHz.

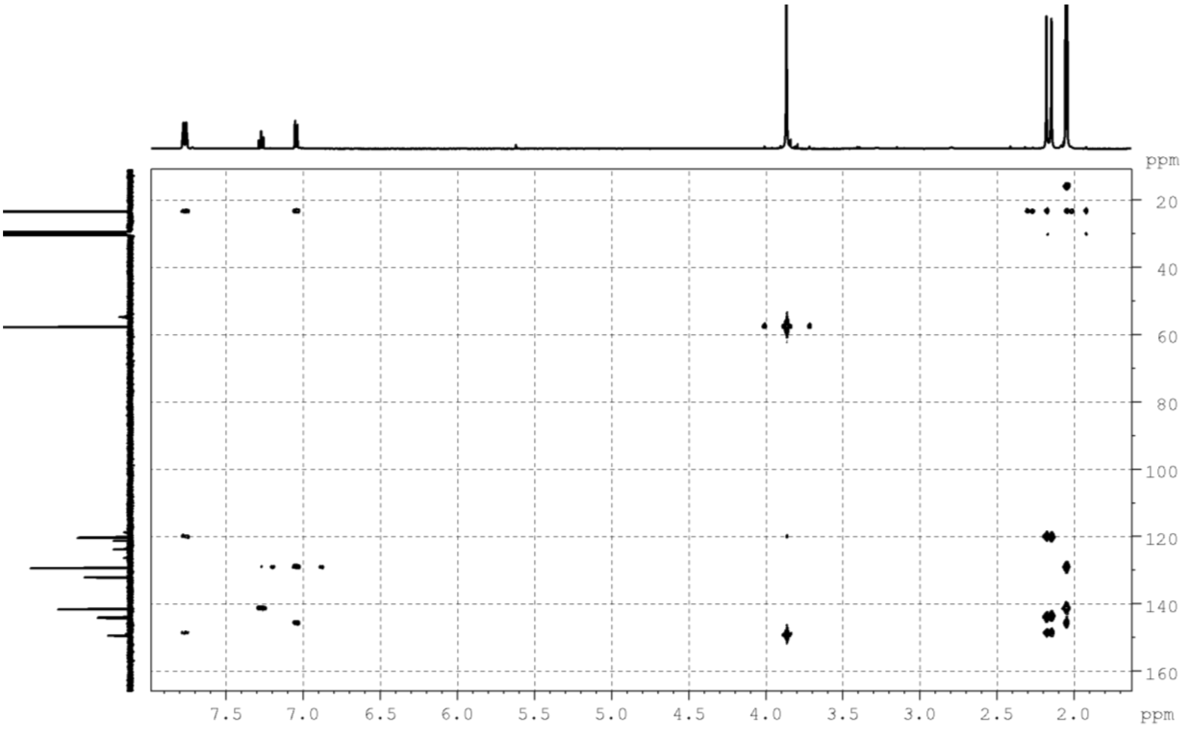

Figure S61: ^{1}H – ^{13}C HMBC spectrum of compound **6c** recorded in acetone-d_6 at 500 MHz.

Crystal Structures

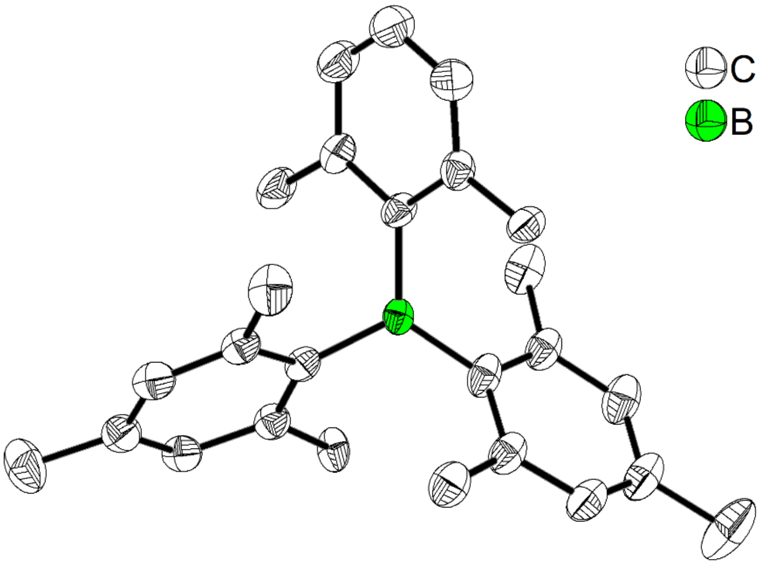

Figure S62: The solid-state molecular structure of **4a** determined by single-crystal X-ray diffraction at 100 K. All ellipsoids are drawn at the 50% probability level. H atoms are omitted for clarity. One of the mesityl groups together with the xylyl group are disordered and only the major parts (70%) are shown here.

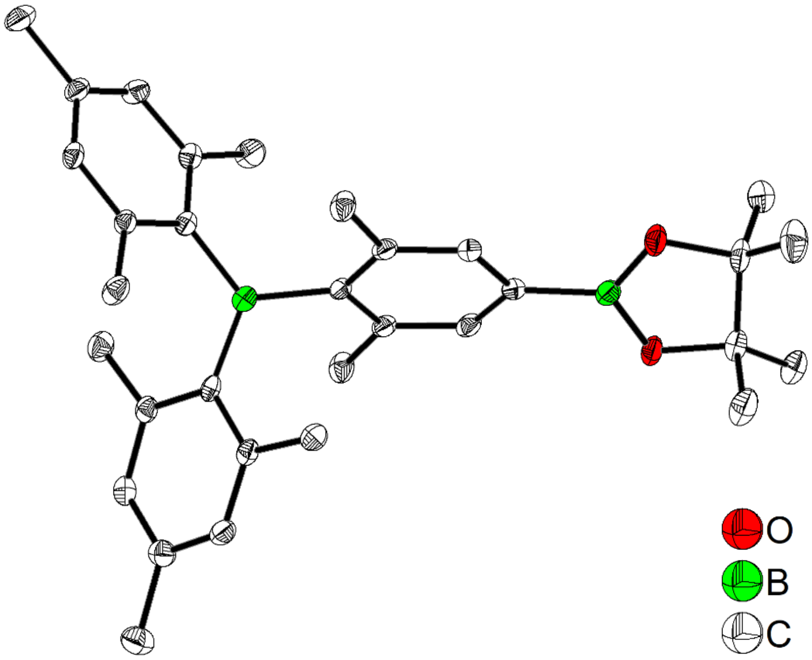

Figure S63: The solid-state molecular structure of **4b** determined by single-crystal X-ray diffraction at 100 K. All ellipsoids are drawn at the 50% probability level. H atoms are omitted for clarity.

Table S1: Single-crystal X-ray diffraction data and structure refinements of **4a** and **4b**.

Data	**4a**	**4b**
CCDC number	2072401	2072402
Empirical formula	$C_{26}H_{31}B$	$C_{32}H_{42}B_2O_2$
Formula weight / g·mol^{-1}	354.32	480.27
T / K	100(2)	100(2)
Radiation, λ / Å	Mo-K$_\alpha$ 0.71073	Mo-K$_\alpha$ 0.71073
Crystal size / mm^3	0.29×0.34×0.37	0.59×0.59×0.75
Crystal color, habit	colorless block	colorless block
Crystal system	monoclinic	monoclinic
Space group	*Cc*	$P2_1/n$
a / Å	11.796(6)	8.157(3)
b / Å	11.621(5)	38.889(18)
c / Å	16.070(7)	8.858(5)
α / °	90	90
β / °	107.577(17)	92.36(3)
γ / °	90	90
Volume / Å^3	2100.0(17)	2808(2)
Z	4	4
ρ_{calc} / g·cm^{-3}	1.121	1.136
μ / mm^{-1}	0.062	0.067
F(000)	768	1040
θ range / °	2.520 – 27.484	2.360 – 26.054
Reflections collected	17611	20889
Unique reflections	4806	5368
Parameters / restraints	408 / 752	337 / 0
GooF on F^2	1.049	1.117
R_1 [I>2σ(I)]	0.0461	0.0627
wR^2 (all data)	0.1346	0.1514
Max. / min. residual electron density / e·Å^{-3}	In s0.303 / –0.174	0.287 / –0.253

Solubility in Water

The solubility of **Cat^{1+}**-**Cat^{3+}** in pure water is not sufficient for the determination of an extinction coefficient. Figure S64 shows that upon shaking ca. 30 mmol of each compound in 10 mL of Millipore water for 3 d results in very weakly absorbing solutions. This indicates that small amounts of the compounds **Cat^{2+}**, **Cat(i)$^{2+}$**, and **Cat^{3+}** dissolved upon intensive shaking. However, for **Cat^{1+}**, no absorption was detected. Thus, this compound is not soluble in pure water. Upon increasing the concentration, the compounds do not dissolve as single molecules, leading to slightly colored solutions which become colorless upon filtration. Therefore, it is suggested that aqueous solutions of all compounds are prepared from concentrated solutions in MeCN which can be diluted with water to a percentage as low as ca. 0.7% of MeCN in water without precipitation of the compounds.

Table S2: Summary of conditions applied for determination of extinction coefficient of selectively charged, *bis*-triarylborane chromophores in pure water. ✗ = not detected.

Compound	**Cat^{1+}**	**Cat^{2+}**	**Cat(i)$^{2+}$**	**Cat^{3+}**
Amount [mg]	0.0358	0.0324	0.0372	0.0372
Amount [mol]	33.64 x10^{-9}	25.77 x10^{-9}	29.59 x10^{-9}	25.65 x10^{-9}
Expected concentration [mol/L]	3.36 x10^{-6}	2.58 x10^{-6}	2.96 x10^{-6}	2.56 x10^{-6}
Visible color of shaken solution	colorless	pale yellow	pale yellow	pale yellow
Color of solution after filtration	colorless	colorless	colorless	colorless
Absorption detected after filtration	✗	✗	✗	✗

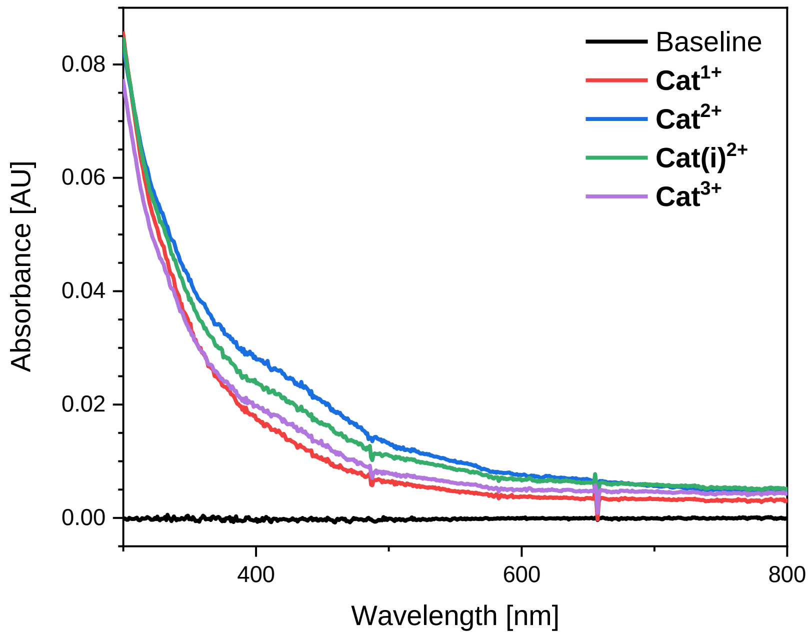

Figure S64: Absorption of filtered, aqueous solutions of selectively cationic, *bis*-triarylborane chromophores measured in 1 mL quartz cuvettes.

Photophysical Properties

The neutral triarylboranes **4a**, **5a**, and **6a** display no significant solvatochromism in their absorption spectra, while their emission spectra display positive solvatochromism. This bathochromic shift increases with increasing number of dimethylamino groups. However, the introduction of a second dimethylamino group in **6a** does not lead to a more pronounced redshift of the emission maximum. The molar extinction coefficient increases by ca. 7000 L mol^{-1} cm^{-1} per amino group. The fluorescence lifetime and fluorescence quantum yield of the amino-substituted triarylboranes **5a** and **6a** increase with solvent polarity. For the latter, this trend is interrupted in acetonitrile.

For the cationic triarylboranes **5c** and **6c**, solvatochromism was not observed in their absorption spectra but a small, positive solvatochromism was observed in their emission spectra. The fluorescence lifetime and fluorescence quantum yield increase with solvent polarity. The molar extinction coefficient decreases when changing the solvent from acetonitrile to 1% MeCN in water, but is almost the same for both compounds. Thus, upon methylation, positive solvatochromism of the emission is almost lost most likely due to the loss of the charge-transfer process which was supported by DFT and TD-DFT calculations. The latter show that the HOMOs of all triarylboranes are located at the most electron-rich aromatic system (4-(*N*,*N*-dimethylamino)-2,6-dimethylphenyl for **5a**, **6a**; mesityl for **4a**, **5c**; 2,6-dimethylphenyl for **6c**). If there are more than one, both arenes contribute equally. Additionally, the LUMOs are mainly localized at the boron center and the π* orbitals of the three arenes in all cases. For **5c** and **6c**, the LUMOs are extended to the trimethylammonium-substituted arene due to the contribution of the electron-withdrawing NMe_3^+ groups. Thus, the HOMO-LUMO transition is mainly of CT-character which is supported by the Λ values obtained from TD-DFT calculations which are smaller than 0.6 for all triarylboranes.

The neutral *bis*-triarylboranes, do not display clear solvatochromism in their absorption spectra, but a positive solvatochromism in their emission was observed. For **Neut0**, no change of the fluorescence lifetime or the fluorescence quantum yield was observed upon increasing the solvent polarity while for the amino substituted compounds, both values increase with increasing solvent polarity. However, when dissolved in the same solvent, the properties of **Neut1**-**Neut3** are very similar, except for the molar extinction coefficient in hexane, which was found to increase non-linearly with increasing number of amino groups. Thus, CT character of the main transition can be assumed for **Neut1**-**Neut3**, but not for **Neut0** as the latter bears no amino groups. DFT calculations show the HOMOs and LUMOs of the neutral *bis*-triarylboranes to be mainly localized at the bithiophene bridge. Thus, the transitions of lowest energy are of π-π* nature. This is further supported by TD-DFT calculations and Λ values between 0.66 and 0.72, thus reflecting delocalized orbitals and locally excited transitions.[30] In contrast, for the

cationic *bis*-triarylboranes, Λ values are between 0.30 and 0.53 reflecting the CT character of the transitions, and rather localized orbitals. The HOMOs of the cationic *bis*-triarylboranes are mainly localized at the bithiophene bridge, while the LUMOs are mainly localized at the most electron deficient boron center (**5c** for **Cat^{1+}**; **6c** for **Cat^{2+}**, **Cat^{3+}**). Therefore, the transition for most of the cationic *bis*-triarylboranes is not of π-π* nature but of π-p nature. In the case of **Cat(i)$^{2+}$** and **Cat^{4+}**,[4] the LUMO is expanded from one boron center to the other over the bithiophene bridge. As a result, the lowest energy transition is of π-π* nature.

The absorption spectra of the cationic *bis*-triarylboranes do not display solvatochromism, while the absorption maxima determined in the same solvent shift bathochromically with increasing number of trimethylammonium groups. A small positive solvatochromism is found in the emission spectra of each compound, while the emission maxima shift bathochromically with increasing dipole moment of the cationic *bis*-triarylboranes in the order **Cat^{1+}≈Cat(i)$^{2+}$<<Cat^{3+}<Cat^{2+}**. Note that the dipole moment of charged compounds is defined relative to its origin and thus, is not an observable quantity. However, the term dipole moment will be used herein to describe the distribution of the electron density over the molecules for convenience. The fluorescence lifetimes and fluorescence quantum yields determined in acetonitrile increase with increasing dipole moment of the cationic *bis*-triarylboranes, while the molar extinction coefficient does not change significantly with increasing number of trimethylammonium groups. In 1% MeCN in water, the molar extinction coefficient increases by ca. 12 000 L mol^{-1} cm^{-1} with the number of trimethylammonium groups. The effects of the number or distribution of trimethylammonium groups on absorption, emission, fluorescence lifetime, or fluorescence quantum yield is less pronounced than that in pure acetonitrile.

In summary, the photophysical properties of **4a**, **5c**, and **6c** are very similar to one another as these compounds are electronically similar due to the loss of the electron-donating amine upon methylation. Introduction of a strong electron-donating functionality, in this case dimethylamino group(s), leads to redshifted absorption and emission spectra and to an increase in the molar extinction coefficient. Similar results were found for the *bis*-triarylboranes. The absorption and emission maxima of all triarylboranes and *bis*-triarylboranes investigated are solvent dependent as expected for donor-acceptor (D–A) compounds in which the boron center is the electron-acceptor. The electron-donor for the neutral compounds is the amine moiety. In the case of **4a** and **5c**, the mesityl group acts as the electron-donor, whereas for **6c**, only the 2,6-dimethylphenyl moiety is left as supported by DFT and TD-DFT calculations. Upon methylation of the neutral *bis*-triarylboranes, the bithiophene becomes the electron-donating moiety. The absorption and emission maxima of the cationic *bis*-triarylboranes were found to be bathochromically shifted compared to their respective, single triarylboranes. Thus, it can be concluded that the photophysical properties result mainly from the interaction of the most

electron-poor boron center with the most electron-rich donating moiety for all compounds investigated.

Neutral Triarylboranes

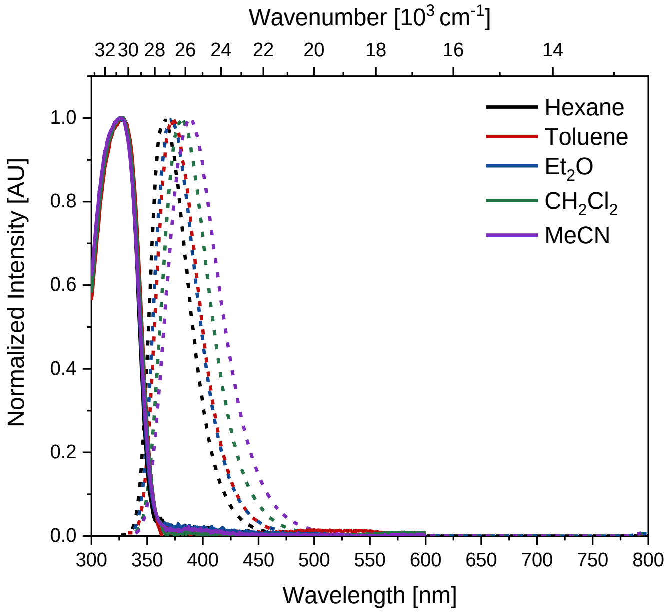

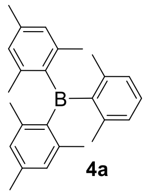

4a

Figure S65: Absorption (solid lines) and emission (dotted lines; excitation at λ_{max}^{abs}) spectra of **4a** in solvents of different polarity.

Table S3: Photophysical properties of compound **4a**.

Solvent	λ_{max}^{abs} [nm]	λ_{max}^{fl} [nm]	Apparent Stokes shift [cm^{-1}]	τ [ns]	Φ_f	τ_0 [ns]	k_{nr} [10^9 s^{-1}]	k_r [10^9 s^{-1}]	ε [L mol^{-1} cm^{-1}]
Hexane	326	367	3400	1.48	0.10	14.8	0.61	0.07	14 700
Toluene	326	374	3900	1.40	0.06	23.3	0.67	0.04	15 000
Et_2O	325	371	3800	1.50	0.08	18.7	0.61	0.05	
CH_2Cl_2	326	381	4400	1.40	0.07	20.0	0.66	0.05	
MeCN	326	388	4900	1.56	0.07	22.3	0.60	0.04	

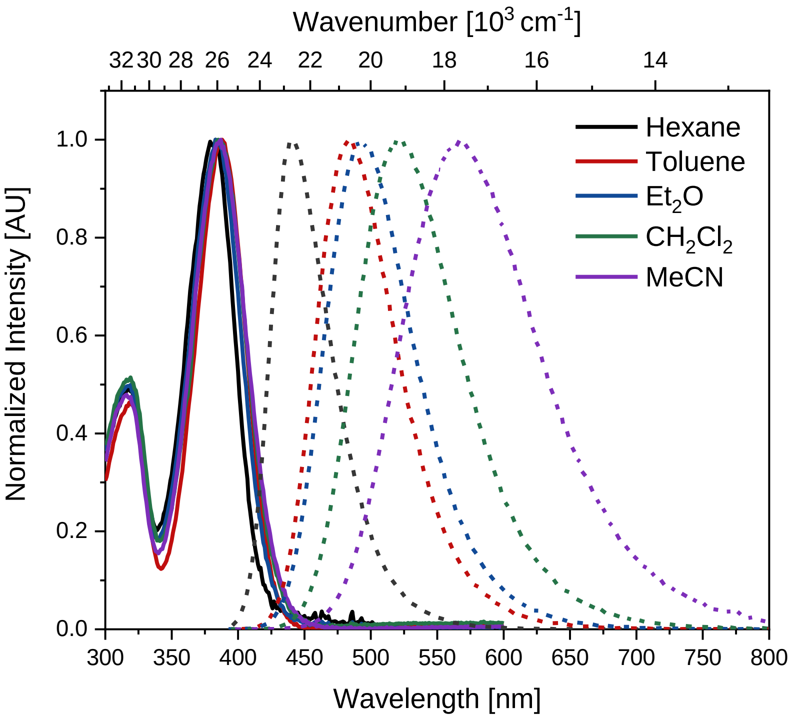

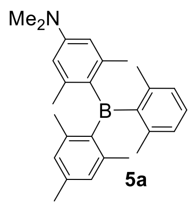

5a

Figure S66: Absorption (solid lines) and emission (dotted lines; excitation at λ_{max}^{abs}) spectra of **5a** in solvents of different polarity.

Table S4: Photophysical properties of compound **5a**.

Solvent	λ_{max}^{abs} [nm]	λ_{max}^{fl} [nm]	Apparent Stokes shift [cm^{-1}]	τ [ns]	Φ_f	τ_0 [ns]	k_{nr} [10^9 s^{-1}]	k_r [10^9 s^{-1}]	ε [L mol^{-1} cm^{-1}]
Hexane	383	440	3400	5.17	0.34	15.2	0.13	0.07	22 000
Toluene	388	486	5200	8.53	0.48	17.8	0.06	0.06	
Et_2O	383	492	5800	10.22	0.48	21.3	0.05	0.05	
CH_2Cl_2	385	520	6700	15.40	0.58	26.5	0.03	0.04	
MeCN	387	567	8200	11.48	0.25	45.9	0.07	0.02	

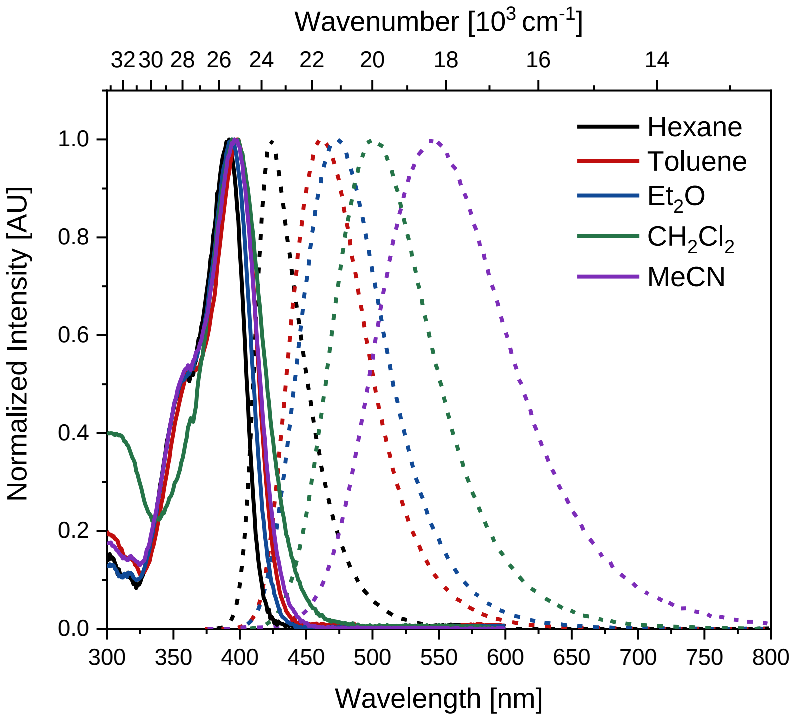

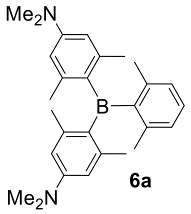

Figure S67: Absorption (solid lines) and emission (dotted lines; excitation at λ_{max}^{abs}) spectra of **6a** in solvents of different polarity.

Table S5: Photophysical properties of compound **6a**.

Solvent	λ_{max}^{abs} [nm]	λ_{max}^{fl} [nm]	Apparent Stokes shift [cm^{-1}]	τ [ns]	Φ_f	τ_0 [ns]	k_{nr} [10^9 s^{-1}]	k_r [10^9 s^{-1}]	ε [L mol^{-1} cm^{-1}]
Hexane	390	424	2100	1.55	0.13	11.9	0.56	0.08	30 000
Toluene	398	462	3500	0.16<br>3.32	0.25	13.3	0.23	0.08	27 000
Et_2O	394	474	4300	5.24	0.24	21.8	0.14	0.05	
CH_2Cl_2	397	501	5200	12.01	0.41	29.3	0.05	0.03	
MeCN	395	546	7000	9.50	0.28	33.9	0.08	0.03	

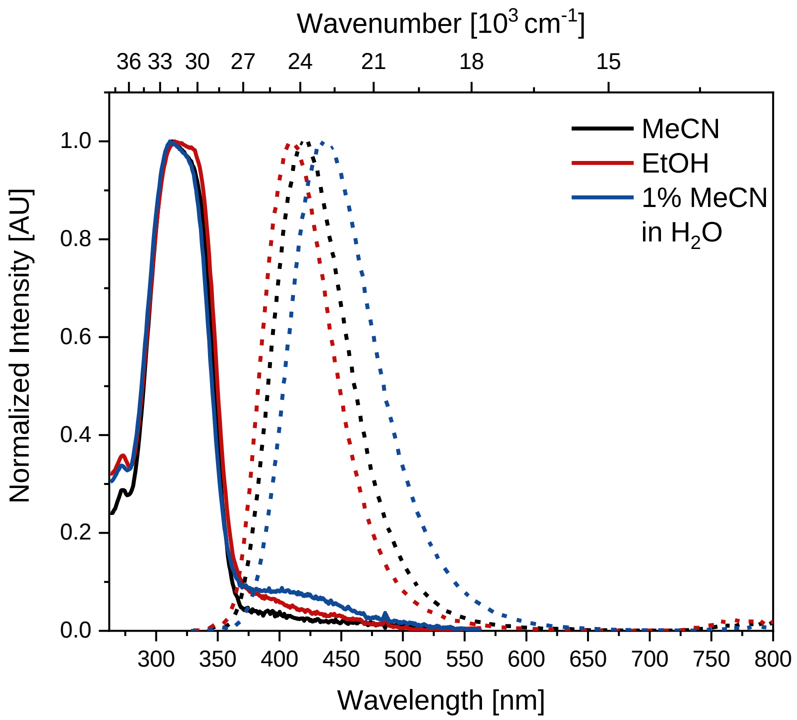

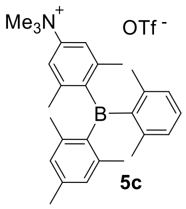

5c

Figure S68: Absorption (solid lines) and emission (dotted lines; excitation at λ_{max}^{abs}) spectra of **5c** in solvents of different polarity.

Table S6: Photophysical properties of compound **5c**.

Solvent	λ_{max}^{abs} [nm]	λ_{max}^{fl} [nm]	Apparent Stokes shift [cm^{-1}]	τ [ns]	Φ_f	τ_0 [ns]	k_{nr} [10^9 s^{-1}]	k_r [10^9 s^{-1}]	ε [L mol^{-1} cm^{-1}]
MeCN	314	420	8 000	3.37	0.11	30.6	0.26	0.03	10 000
EtOH	315	410	7 400	2.39	0.08	29.9	0.38	0.03	
1% MeCN in H_2O	313	437	9 100	7.49	0.19	39.4	0.11	0.02	7 000

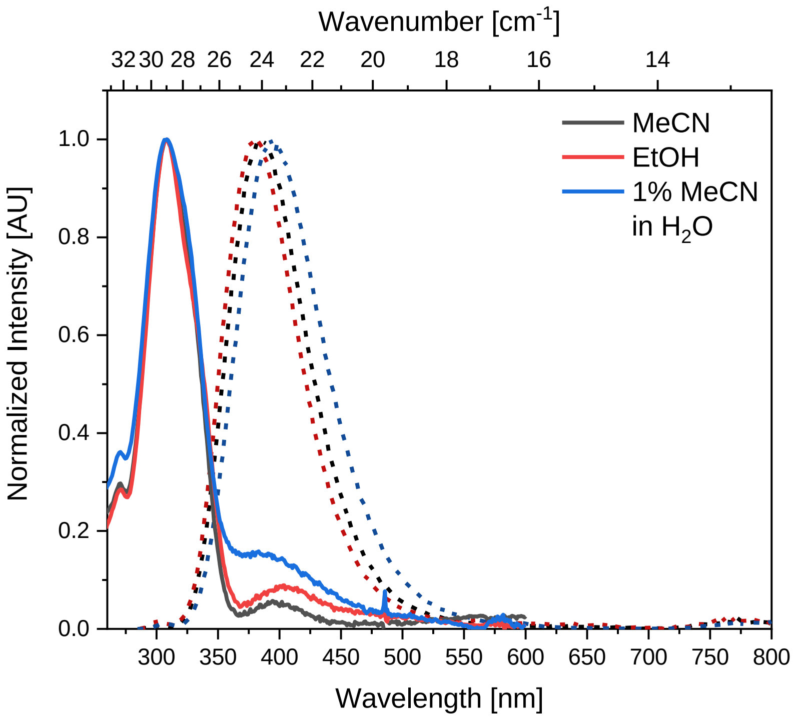

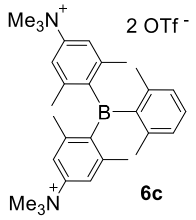

6c

Figure S69: Absorption (solid lines) and emission (dotted lines; excitation at λ_{max}^{abs}) spectra of **6c** in solvents of different polarity.

Table S7: Photophysical properties of compound **6c**.

Solvent	λ_{max}^{abs} [nm]	λ_{max}^{fl} [nm]	Apparent Stokes shift [cm^{-1}]	τ [ns]	Φ_f	τ_0 [ns]	k_{nr} [10^9 s^{-1}]	k_r [10^9 s^{-1}]	ε [L mol^{-1} cm^{-1}]
MeCN	309	416	8 300	4.40	0.10	44.0	0.20	0.02	11 400
EtOH	308	412	8 200	3.76	0.09	41.8	0.24	0.02	
1% MeCN in H_2O	308	425	8 900	6.65	0.11	60.4	0.13	0.02	10 300

Neutral *bis*-Triarylboranes

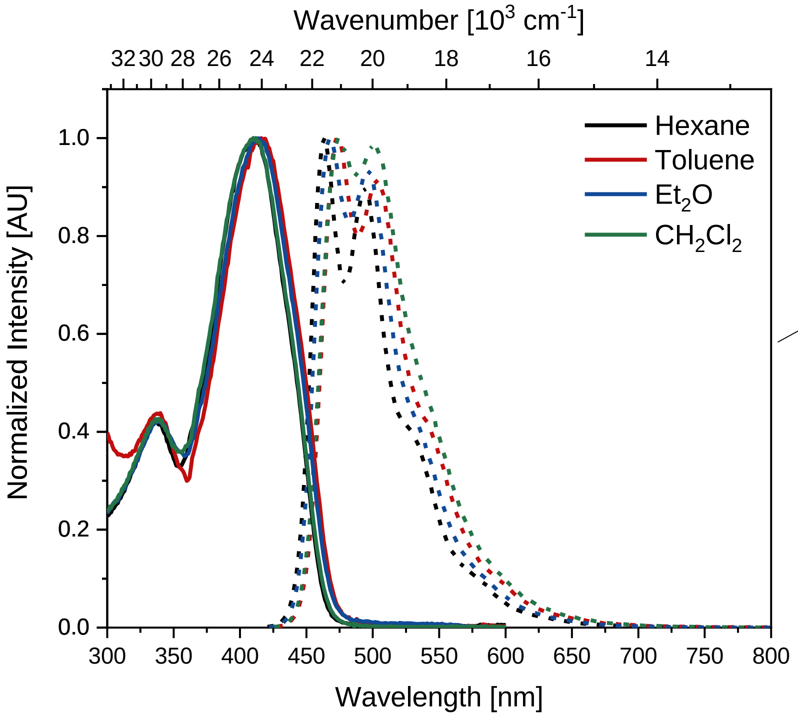

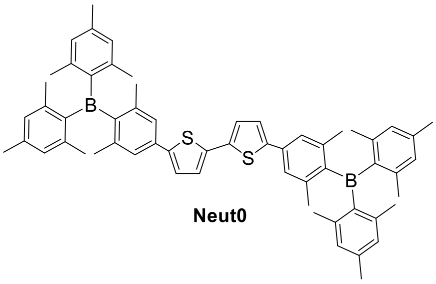

Neut0

Figure S70: Absorption (solid lines) and emission (dotted lines; excitation at λ_{max}^{abs}) spectra of **Neut0** in solvents of different polarity.

Table S8: Photophysical properties of compound **Neut0**.

Solvent	λ_{max}^{abs} [nm]	λ_{max}^{fl} [nm]	Apparent Stokes shift [cm^{-1}]	τ [ns]	Φ_f	τ_0 [ns]	k_{nr} [10^9 s^{-1}]	k_r [10^9 s^{-1}]	ε [L mol^{-1} cm^{-1}]
Hexane	410	464	2 800	0.52	0.31	1.68	1.33	0.60	65 700
Toluene	416	474	2 900	0.56	0.35	1.60	1.16	0.62	52 000
Et_2O	410	470	3 100	0.58	0.33	1.76	1.16	0.57	
CH_2Cl_2	414	477	3 200	0.55	0.31	1.77	1.25	0.56	

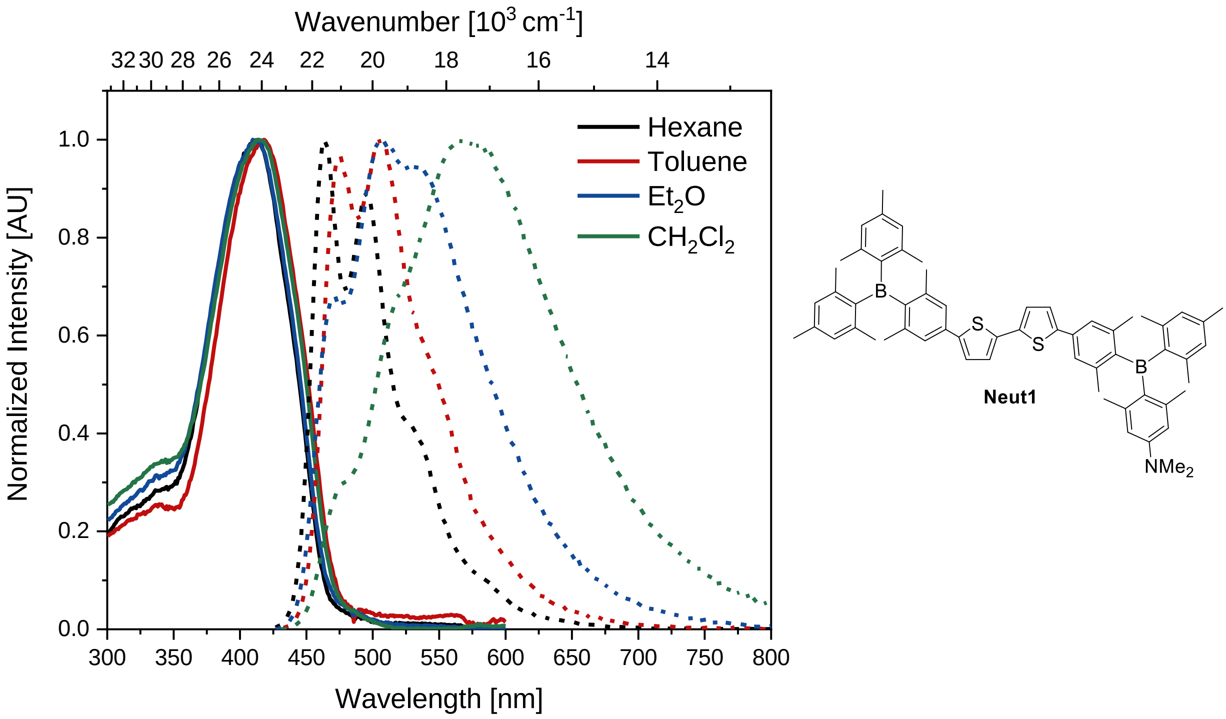

Figure S71: Absorption (solid lines) and emission (dotted lines; excitation at λ_{max}^{abs}) spectra of **Neut1** in solvents of different polarity.

Table S9: Photophysical properties of compound **Neut1**.

Solvent	λ_{max}^{abs} [nm]	λ_{max}^{fl} [nm]	Apparent Stokes shift [cm^{-1}]	τ [ns]	Φ_f	τ_0 [ns]	k_{nr} [10^9 s^{-1}]	k_r [10^9 s^{-1}]	ε [L mol^{-1} cm^{-1}]
Hexane	412	464	2 700	0.54	0.38	1.42	1.15	0.70	75 000
Toluene	418	507	4 200	0.33 (13%) 1.61 (87%)	0.36	-	-	-	
Et_2O	412	507	4 500	0.61 (19%) 4.35 (81%)	0.33	-	-	-	
CH_2Cl_2	416	568	6 400	0.69 (21%) 4.67 (79%)	0.21	-	-	-	

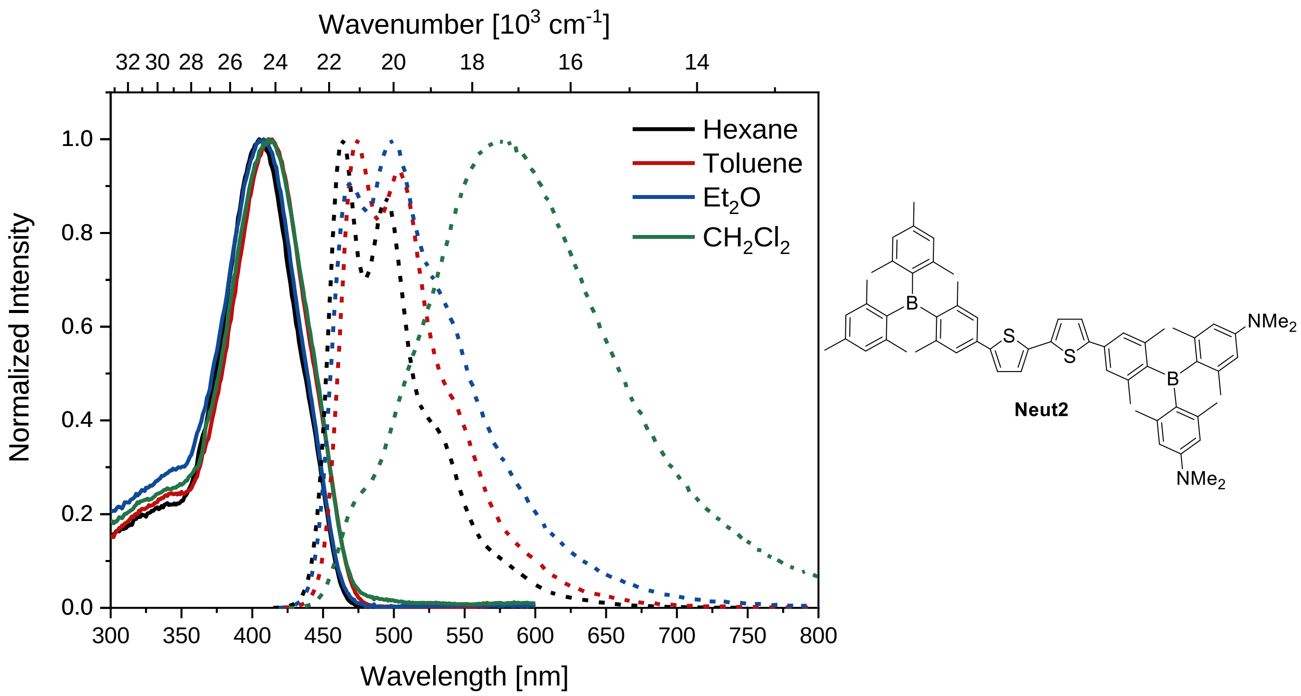

Figure S72: Absorption (solid lines) and emission (dashed lines; excitation at λ_{max}^{abs}) spectra of **Neut2** in solvents of different polarity.

Table S10: Photophysical properties of **Neut2** in solvents of different polarity.

Solvent	λ_{max}^{abs} [nm]	λ_{max}^{fl} [nm]	Apparent Stokes shift [cm^{-1}]	τ [ns]	τ_0 [ns]	k_{nr} [10^9 s^{-1}]	k_r [10^9 s^{-1}]	Φ_f	ε [L mol^{-1} cm^{-1}]
Hexane	405	464	3 100	0.54	1.69	1.26	0.59	0.32	76 000
Toluene	412	474	3 200	0.89	2.70	0.75	0.37	0.33	-
Et_2O	408	499	4 400	2.57	7.79	0.26	0.19	0.33	-
CH_2Cl_2	411	576	7 000	3.92	30.15	0.22	0.03	0.13	-

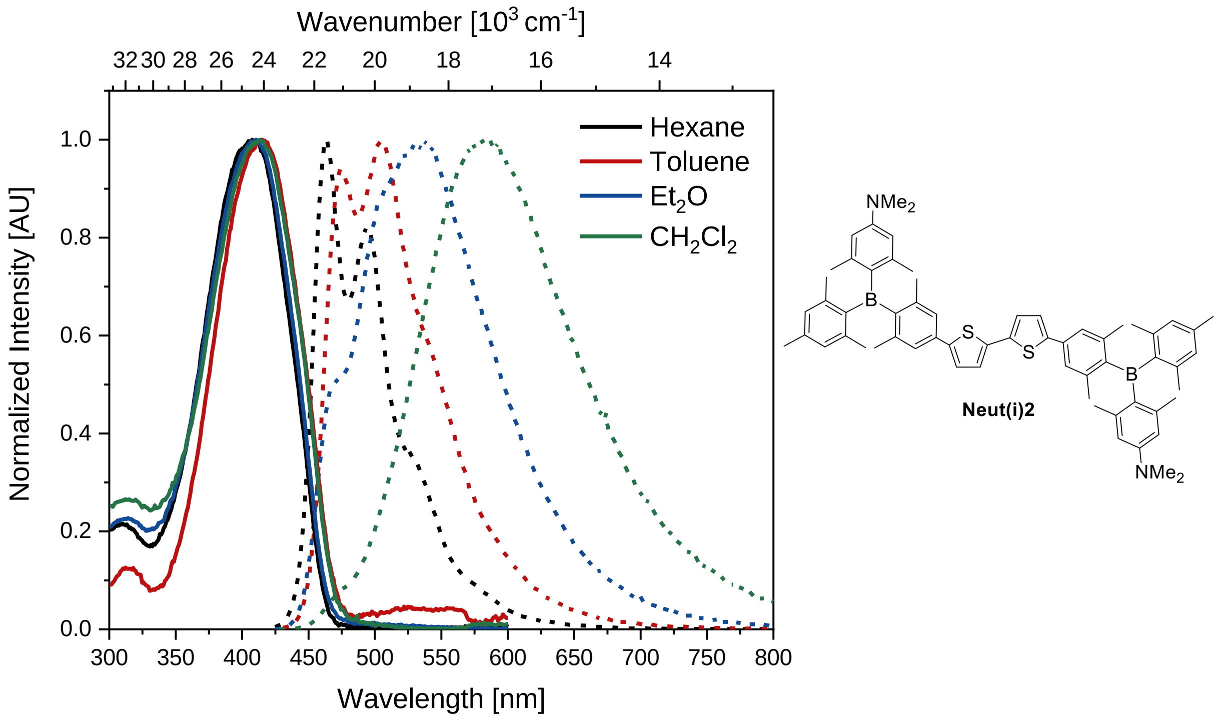

Figure S73: Absorption (solid lines) and emission (dotted lines; excitation at λ_{max}^{abs}) spectra of **Neut(i)2** in solvents of different polarity.

Table S11: Photophysical properties of compound **Neut(i)2**.

Solvent	λ_{max}^{abs} [nm]	λ_{max}^{fl} [nm]	Apparent Stokes shift [cm^{-1}]	τ [ns]	Φ_f	τ_0 [ns]	k_{nr} [10^9 s^{-1}]	k_r [10^9 s^{-1}]	ε [L mol^{-1} cm^{-1}]
Hexane	408	464	2 900	0.54	0.39	1.38	1.13	0.72	103 000
Toluene	416	505	4 200	0.47 (8%) 2.11 (92%)	0.38	-	-	-	
Et$_2$O	410	534	5 700	5.15	0.37	13.9	0.12	0.07	
CH$_2$Cl$_2$	413	583	7 100	4.96	0.20	24.8	0.16	0.04	

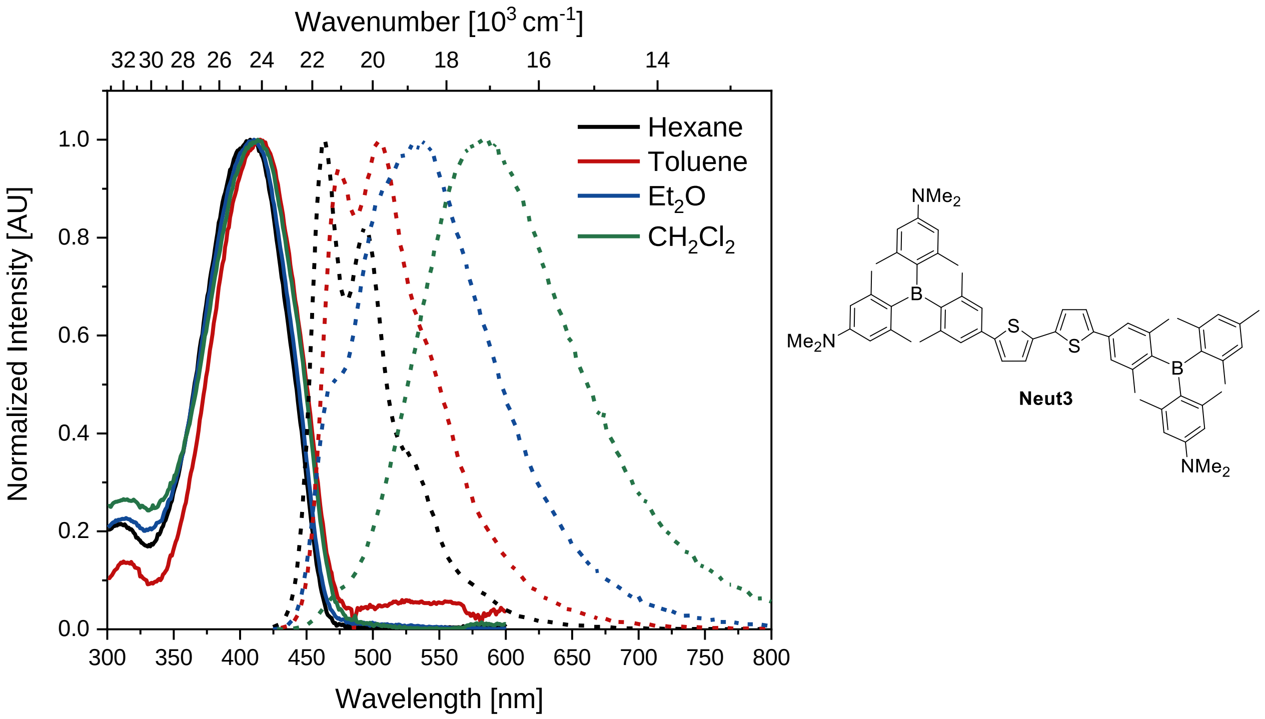

Figure S74: Absorption (solid lines) and emission (dotted lines; excitation at λ_{max}^{abs}) spectra of **Neut3** in solvents of different polarity.

Table S12: Photophysical properties of compound **Neut3**.

Solvent	λ_{max}^{abs} [nm]	λ_{max}^{fl} [nm]	Apparent Stokes shift [cm^{-1}]	τ [ns]	Φ_f	τ_0 [ns]	k_{nr} [10^9 s^{-1}]	k_r [10^9 s^{-1}]	ε [L mol^{-1} cm^{-1}]
Hexane	404	463	3 100	0.52	0.39	1.33	1.17	0.75	129 000
Toluene	411	504	4 500	1.60	0.38	4.21	0.39	0.24	
Et_2O	406	530	5 800	4.58	0.35	13.1	0.14	0.08	
CH_2Cl_2	413	583	7 100	4.77	0.20	21.7	0.16	0.05	

Cationic *bis*-Triarylboranes

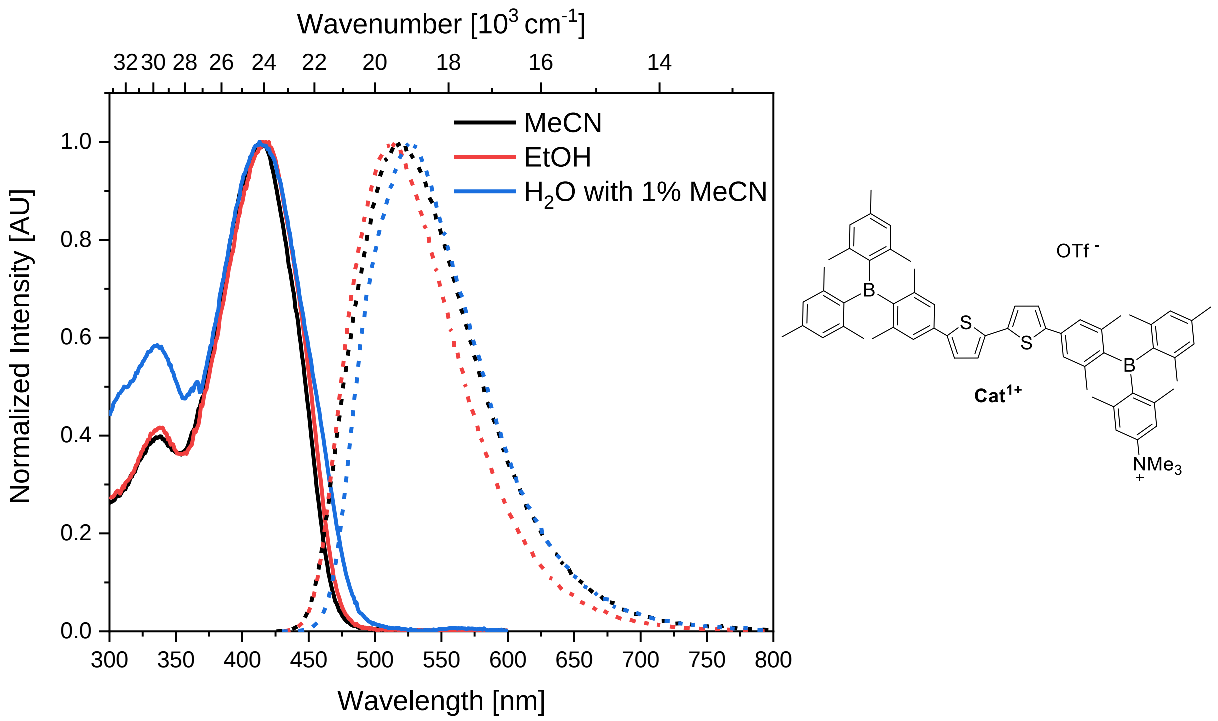

Figure S75: Absorption (solid lines) and emission (dotted lines; excitation at λ_{max}^{abs}) spectra of **Cat^{1+}** in solvents of different polarity.

Table S13: Photophysical properties of **Cat^{1+}** in solvents of different polarity.

Solvent	λ_{max}^{abs} [nm]	λ_{max}^{fl} [nm]	Apparent Stokes shift [cm^{-1}]	τ [ns]	Φ_f	τ_0 [ns]	k_{nr} [10^9 s^{-1}]	k_r [10^9 s^{-1}]	ε [L mol^{-1} cm^{-1}]
EtOH	417	513	4 500	0.70	0.28	2.50	1.03	0.40	-
MeCN	414	519	4 900	0.82	0.34	2.41	0.80	0.41	52 000
1% MeCN in water	415	527	5 100	0.54 (89%) 1.93 (11%)	0.07	-	-	-	29 500

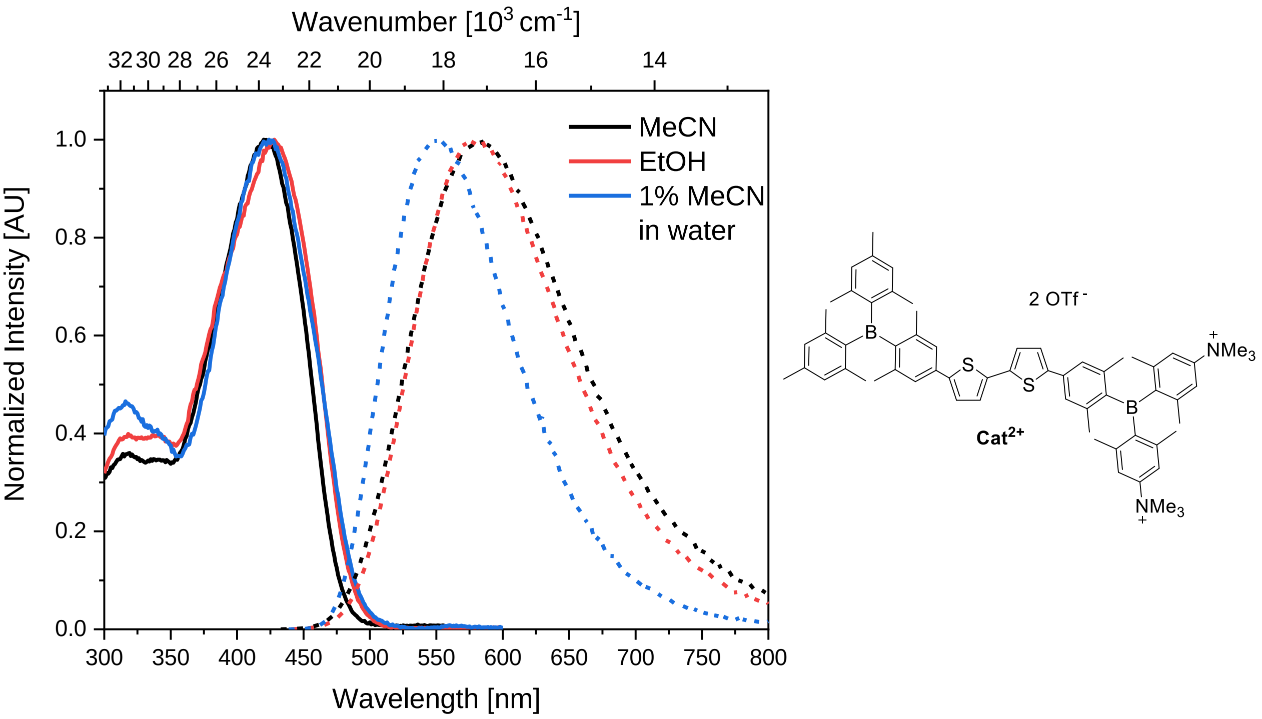

Figure S76: Absorption (solid lines) and emission (dotted lines; excitation at λ_{max}^{abs}) spectra of **Cat**$^{2+}$ in solvents of different polarity.

Table S14: Photophysical properties of **Cat**$^{2+}$.

Solvent	λ_{max}^{abs} [nm]	λ_{max}^{fl} [nm]	Apparent Stokes shift [cm^{-1}]	τ [ns]	Φ_f	τ_0 [ns]	k_{nr} [10^9 s^{-1}]	k_r [10^9 s^{-1}]	ε [L mol^{-1} cm^{-1}]
EtOH	428	582	6 200	3.09	0.49	6.33	0.16	0.16	-
MeCN	420	584	6 700	2.98	0.48	6.21	0.17	0.16	51 000
1% MeCN in water	424	550	5 400	0.76 (52%) 2.74 (48%)	0.15	-	-	-	31 100

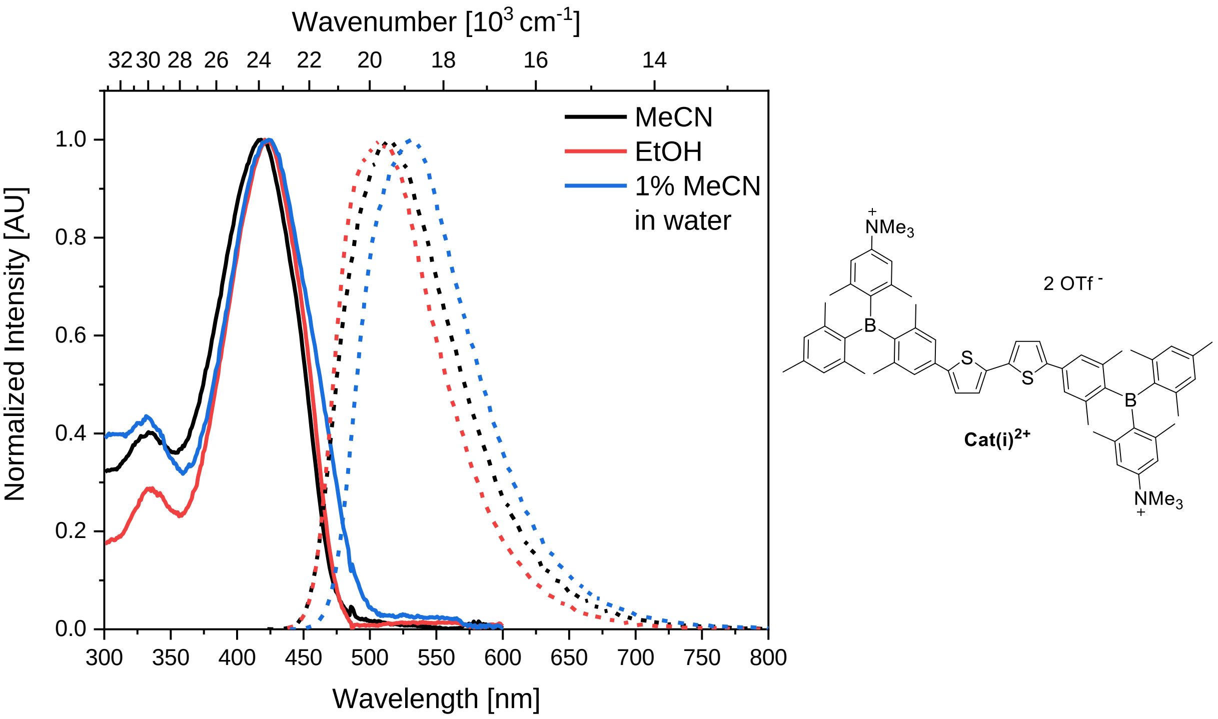

Figure S77: Absorption (solid lines) and emission (dotted lines; excitation at λ_{max}^{abs}) spectra of **Cat(i)$^{2+}$** in solvents of different polarity.

Table S15: Photophysical properties of compound **Cat(i)$^{2+}$**.

Solvent	λ_{max}^{abs} [nm]	λ_{max}^{fl} [nm]	Apparent Stokes shift [cm^{-1}]	τ [ns]	Φ_f	τ_0 [ns]	k_{nr} [10^9 s^{-1}]	k_r [10^9 s^{-1}]	ε [L mol^{-1} cm^{-1}]
EtOH	423	509	4 000	0.70	0.35	2.00	0.93	0.50	-
MeCN	418	515	4 500	0.75	0.35	2.14	0.87	0.47	51 000
1% MeCN in water	424	530	4 700	0.63 (89%) 1.79 (11%)	0.15	-	-	-	3 400

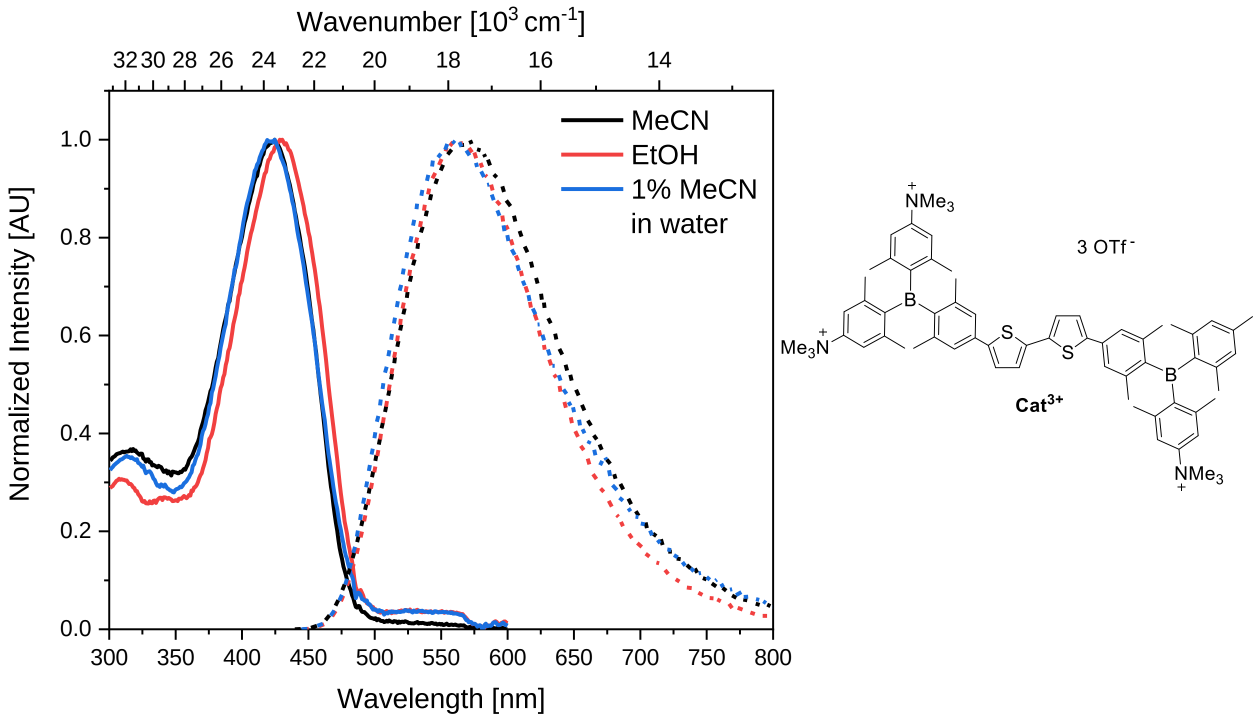

Figure S78: Absorption (solid lines) and emission (dotted lines; excitation at λ_{max}^{abs}) spectra of **Cat^{3+}** in solvents of different polarity.

Table S16: Photophysical properties of compound **Cat^{3+}**.

Solvent	λ_{max}^{abs} [nm]	λ_{max}^{fl} [nm]	Apparent Stokes shift [cm^{-1}]	τ [ns]	Φ_f	τ_0 [ns]	k_{nr} [10^9 s^{-1}]	k_r [10^9 s^{-1}]	ε [L mol^{-1} cm^{-1}]
EtOH	431	564	5 500	0.92 (16%) 2.44 (84%)	0.46	-	-	-	-
MeCN	424	568	6 000	2.41	0.46	5.24	0.22	0.19	51 000
1% MeCN in water	423	558	5 700	0.24 (80%) 1.98 (20%)	0.08	-	-	-	36 000

Singlet Oxygen Sensitizing

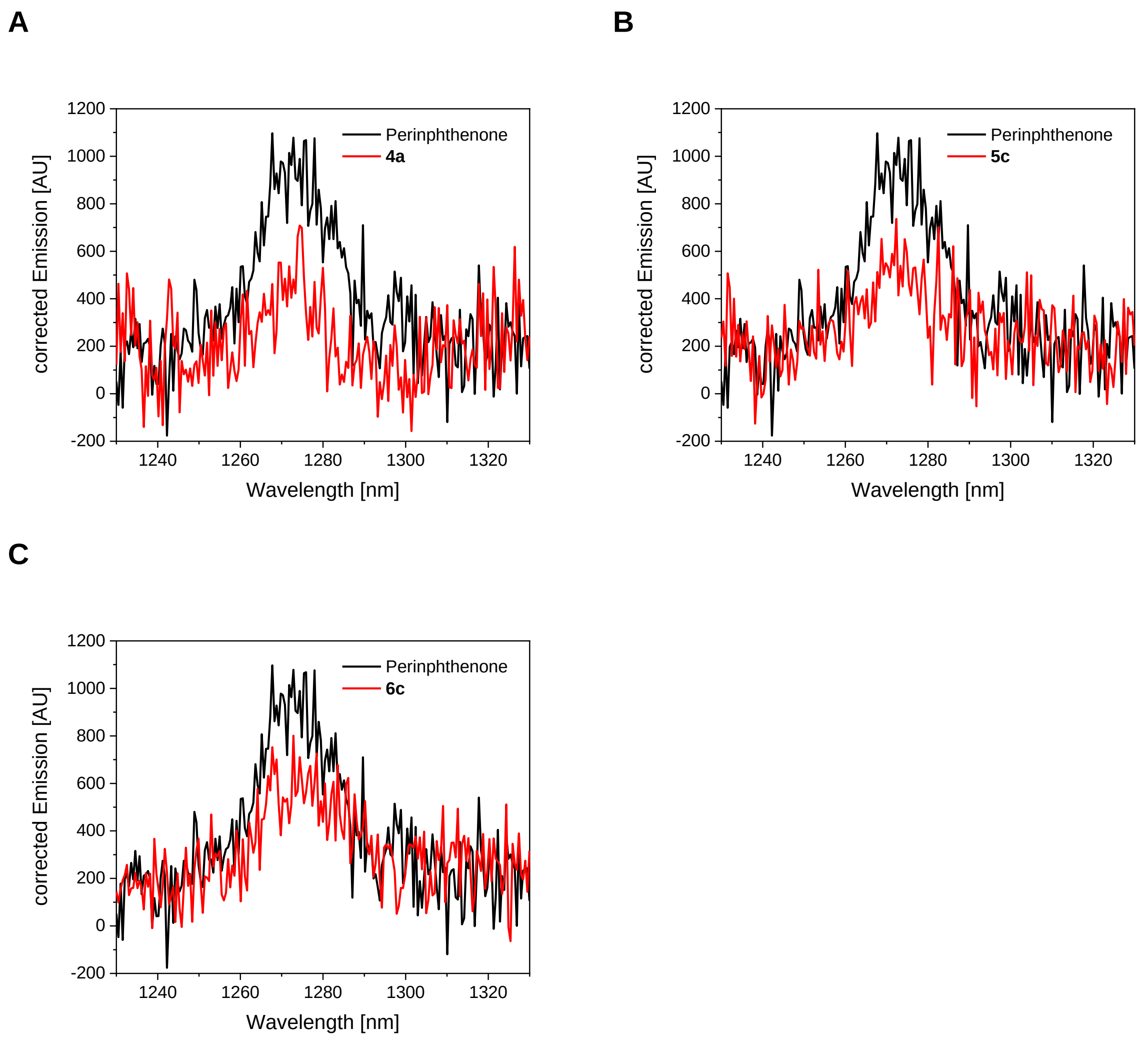

Figure S79: Emission spectra of **A) 4a**, **B) 5c**, and **C) 6c** relative to perinaphthenone determined in acetonitrile.

Table S17: Comparison of singlet oxygen sensitizing efficiency and fluorescence quantum yield of triarylboranes **4a**, **5c**, and **6c**.

Compound	Singlet oxygen sensitizing efficiency Φ_Δ	Fluorescence quantum yield Φ_f
**4a**	0.3	0.34
**5c**	0.6	0.10
**6c**	0.5	0.11

Note that absorption and emission spectra of all compounds recorded after the singlet oxygen measurement remained unchanged (Figure S80, Figure S81).

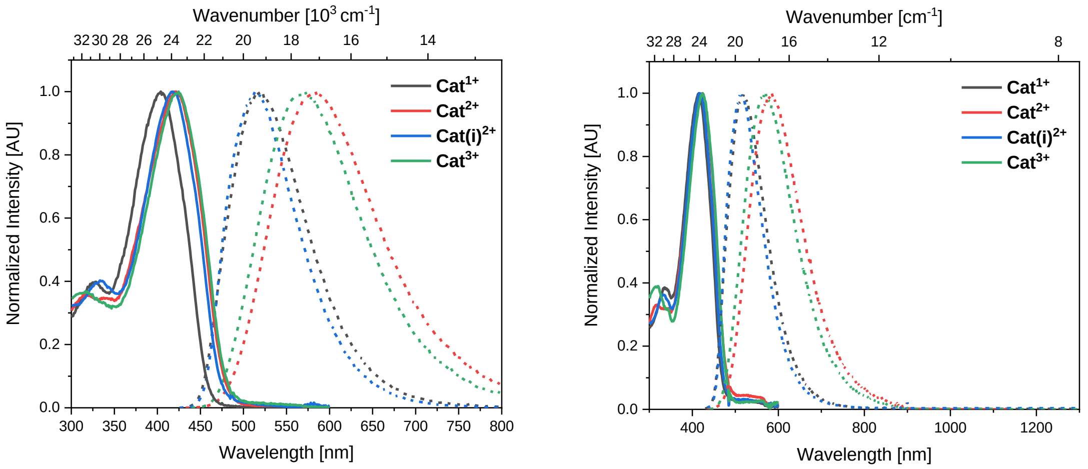

Figure S80: Comparison of absorption and emission spectra of **Cat^{1+}-Cat^{3+}** recorded in acetonitrile before (left) and after (right) singlet oxygen measurement. [Note: emission measured from 430-900 nm with the red-sensitive photomultiplier (PMT-R928P) detector, and from 900.5 to 1300 nm with the near-IR PMT detector described in the general experimental details.]

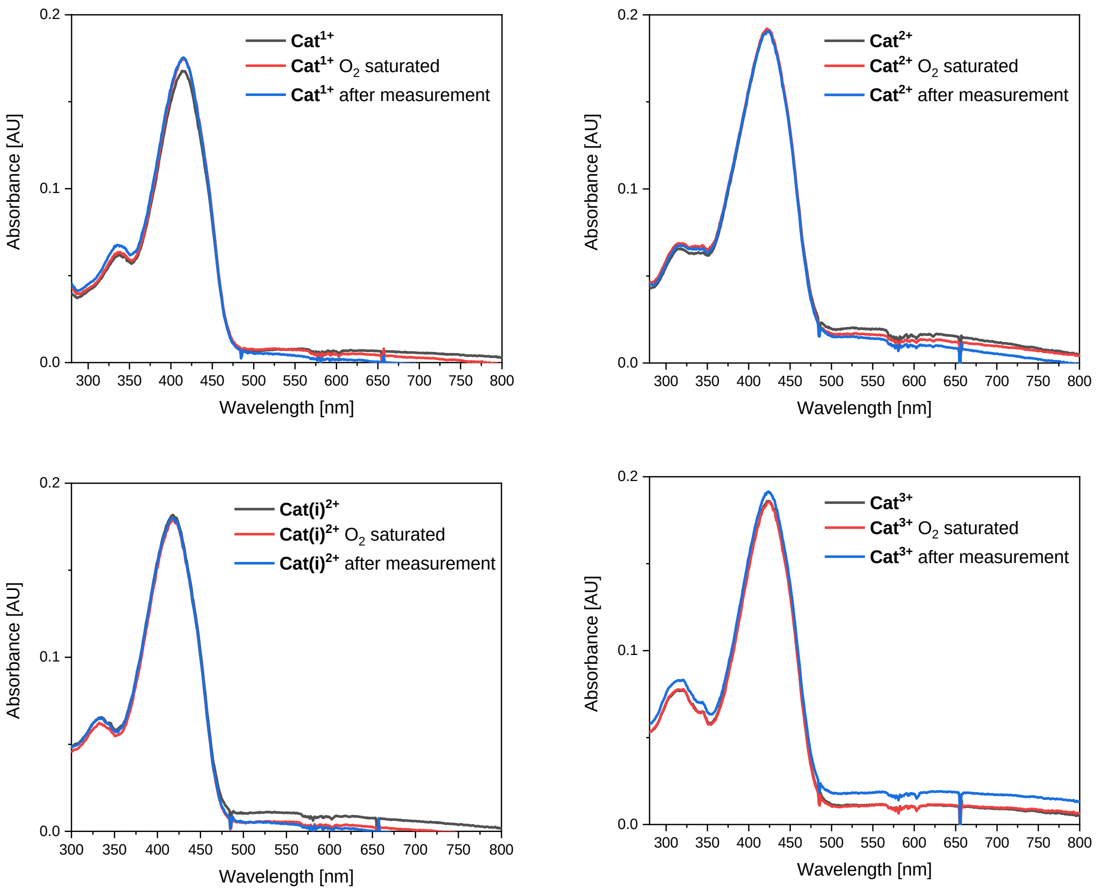

Figure S81: Absorption spectra of **Cat^{1+}-Cat^{3+}** in solution, in O_2 saturated solution and after measurement of singlet oxygen sensing.

Cyclic Voltammetry

For all triarylboranes, one partially reversible $1e^-$ reduction potential for the boron center and one $1e^-$ oxidation potential was found per amino group, the second oxidation potential of **6a** being irreversible. The oxidation potentials do not change within the series of triarylboranes. The reduction potentials decrease with increasing electron density at the boron center which increases in the order **6c**<**5c**<**4a**<**5a**<**6a** due to the more pronounced electron-donating effect of the amino-group NMe_2 compared to the methyl group, and to the electron-withdrawing effect of NMe_3^+ groups. As a result, the LUMO energies decrease upon methylation of the amine (see Table 2). The same is true for the HOMO energies.

For all neutral *bis*-triarylboranes in THF, one partially reversible reduction at ca. -2.35 V was observed which consists of two, simultaneous $1e^-$ reductions. Furthermore, an irreversible $1e^-$ reduction at ca. -3.05 V was observed. For all dimethylamino-substituted *bis*-triarylboranes, an irreversible oxidation occurred at ca. 0.35 V; only **Neut3** has an additional irreversible oxidation at 0.49 V.

For all cationic *bis*-triarylboranes, two fully reversible $1e^-$ oxidation processes were observed at ca. 0.65 V and 1.0 V which result from the bithiophene bridge. However, for **Cat**$^{1+}$, **Cat**$^{2+}$, and **Cat**$^{3+}$, two partially reversible $1e^-$ reductions were observed for each compound. As observed for the neutral *bis*-triarylboranes, **Cat(i)**$^{2+}$ and **Cat**$^{3+}$ have an additional, irreversible reduction. Compounds **Cat(i)**$^{2+}$ and **Cat**$^{4+}$ display only one reduction potential, which results from two simultaneous $1e^-$ reductions, as the boron centers in these *bis*-triarylboranes are the same on both sides of the molecule. Therefore, it can be concluded that their reduction potentials of -2.07 V and -1.92 V might be the same in all *bis*-triarylboranes having the structural motifs **5c** and **6c**, respectively. Indeed, almost identical reduction potentials are found for **Cat**$^{2+}$, and especially for **Cat**$^{3+}$. Thus, the second reduction potential of -2.18 V found for **Cat**$^{2+}$ likely results from the neutral, dimesityl-substituted boron moiety **4a**. Therefore, **Cat**$^{1+}$ is expected to display two reduction potentials at ca. -2.07 V and -2.18 V. However, only one reduction potential at -2.13 V is detected, which results from two simultaneous $1e^-$ reductions. As the expected values differ only by 0.11 V and the width of the waves from these measurements is ca. 0.1 V for the other compounds, it seems plausible that the expected reduction potentials cannot be resolved by the measurement. Thus, an average reduction potential is obtained instead of two individual ones. However, a minor shoulder at the left side of the reduction wave can be observed whose resolution was not improved by square wave experiments (Figure S94D).

Comparing the reduction potential of the cationic triarylboranes **5c** (-2.24 V) and **6c** (-2.02 V) with the potential of the respective moiety in the *bis*-triarylboranes shows less negative

reduction potentials by ca. 0.17 eV and 0.08 eV, respectively. Thus, a lower electron density at the boron centers of the *bis*-triarylboranes can be assumed.

Comparing the results of the neutral *bis*-triarylboranes with the corresponding cationic *bis*-triarylboranes, less negative reduction potentials are observed in the latter due to decreased electron density at the boron centers. The neutral *bis*-triarylboranes show only one reversible reduction potential consisting of two simultaneous 1e$^-$ processes, but the cationic *bis*-triarylboranes show two reversible 1e$^-$ reductions. Thus, it can be concluded that the π-conjugation, and therefore the communication between the boron centers, is improved upon methylation of the amino groups. This finding is in accordance with the results of Griesbeck *et al*.[31] who reported a slight decrease of the rotational barrier around the B–C bond by 0.7 kcal/mol upon methylation of **Neut4** to **Cat^{4+}**.

From the oxidation and reduction potentials, the corresponding HOMO and LUMO energies in the gas phase can be calculated according to literature procedures[32-35] using the following equations:

E(HOMO) = solvent correction − $E_{1/2}^{ox}$

E(LUMO) = solvent correction − $E_{1/2}^{red}$

solvent corrections: CH_2Cl_2: −5.16 eV; MeCN: −5.10 eV; THF: −5.26 eV; these values are obtained from the fact that the oxidation potential of the Fc/Fc^+ couple is different in different solvents when measured against SCE (standard calomel electrode; E(Fc/Fc^+, CH_2Cl_2, $[NBu_4][PF_6]$) = 0.46 eV; E(Fc/Fc^+, MeCN, $[NBu_4][PF_6]$) = 0.40 eV; E(Fc/Fc^+, THF, $[NBu_4][PF_6]$) = 0.56 eV).[34] To refer the values obtained against SHE (standard hydrogen electrode), a value of 4.46 eV has to be taken into account.[33] Finally, the absolute value of the SHE is 0.244 eV.[35] Summarizing these different values leads to the values for the solvent correction shown above resulting in the possibility to calculate the HOMO and LUMO energies in the gas phase from experimental results.[32]

Whenever no oxidation potential was detectable, the onset potential was determined from the absorption spectra, and the HOMO was calculated according to the following equation: E(HOMO) = E(LUMO) − E(onset absorption). Thus, for **4a**, **5c**, **6c**, and **Neut0**, the same values for ΔE_{opt} and ΔE_{CV} are obtained.

The HOMO and LUMO energies of the cationic *bis*-triarylboranes and the neutral *bis*-triarylboranes do not show any dependence on the number of dimethylamino or trimethylammonium groups attached to the bithiophene core. However, upon methylation, the energy of the orbitals tends to decrease slightly. When comparing the HOMO and LUMO

energies of the *bis*-triarylboranes with the values determined for the single triarylboranes, an increase of the HOMO and a slight decrease of the LUMO energies can be observed. Thus, the HOMO-LUMO gap is slightly decreased, most likely due to the introduction of the bithiophene bridge.

Hence, the introduction of the conjugated bridge influences the reduction and oxidation processes more than the introduction of different numbers of dimethylamino or trimethylammonium groups.

Neutral Triarylboranes

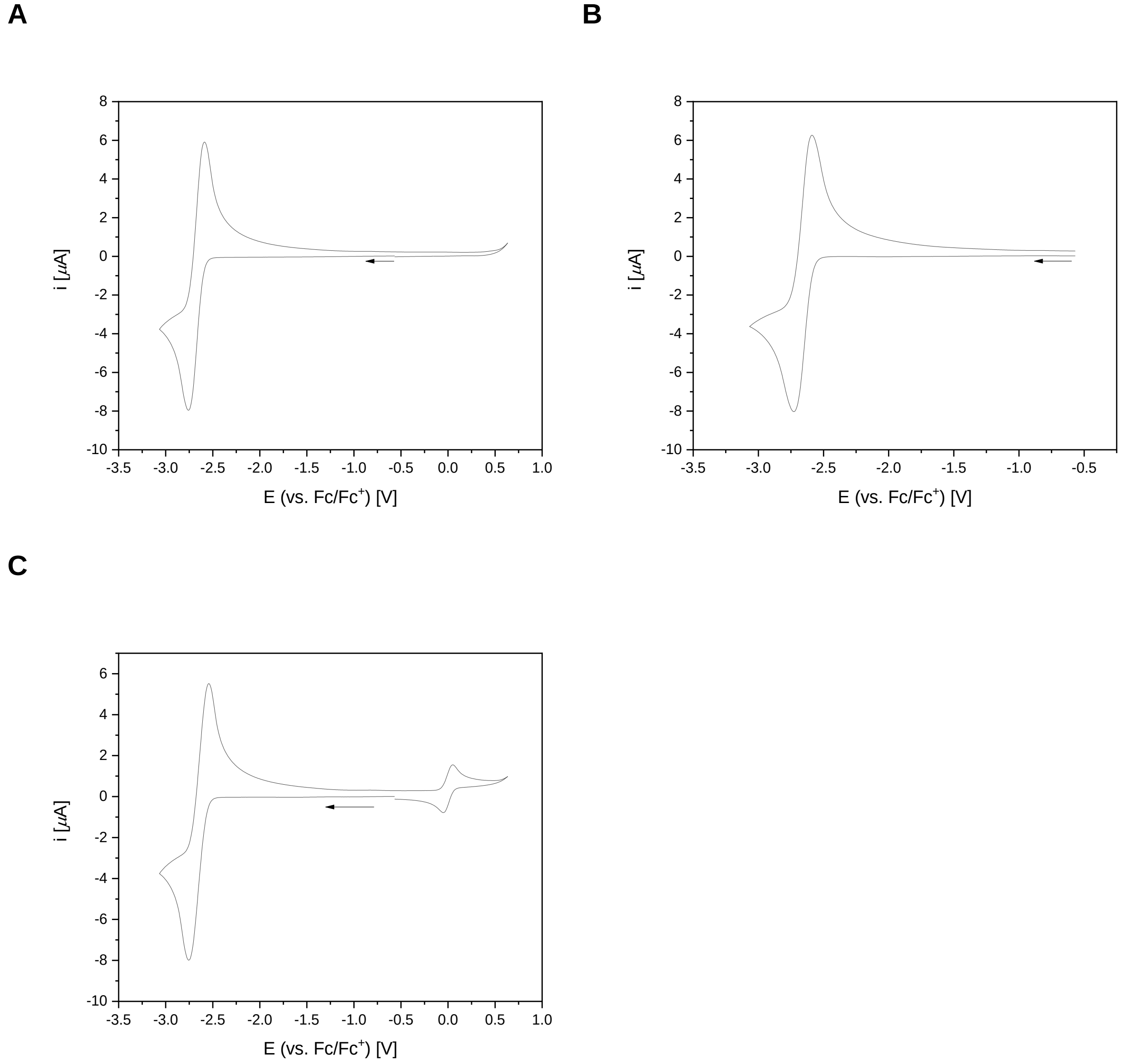

Figure S82: **A)** Cyclic voltammogram of **4a** in THF. **B)** Reversible Reduction of **4a** in THF. **C)** Cyclic voltammogram of **4a** in THF in the presence of Fc/Fc^{+}.

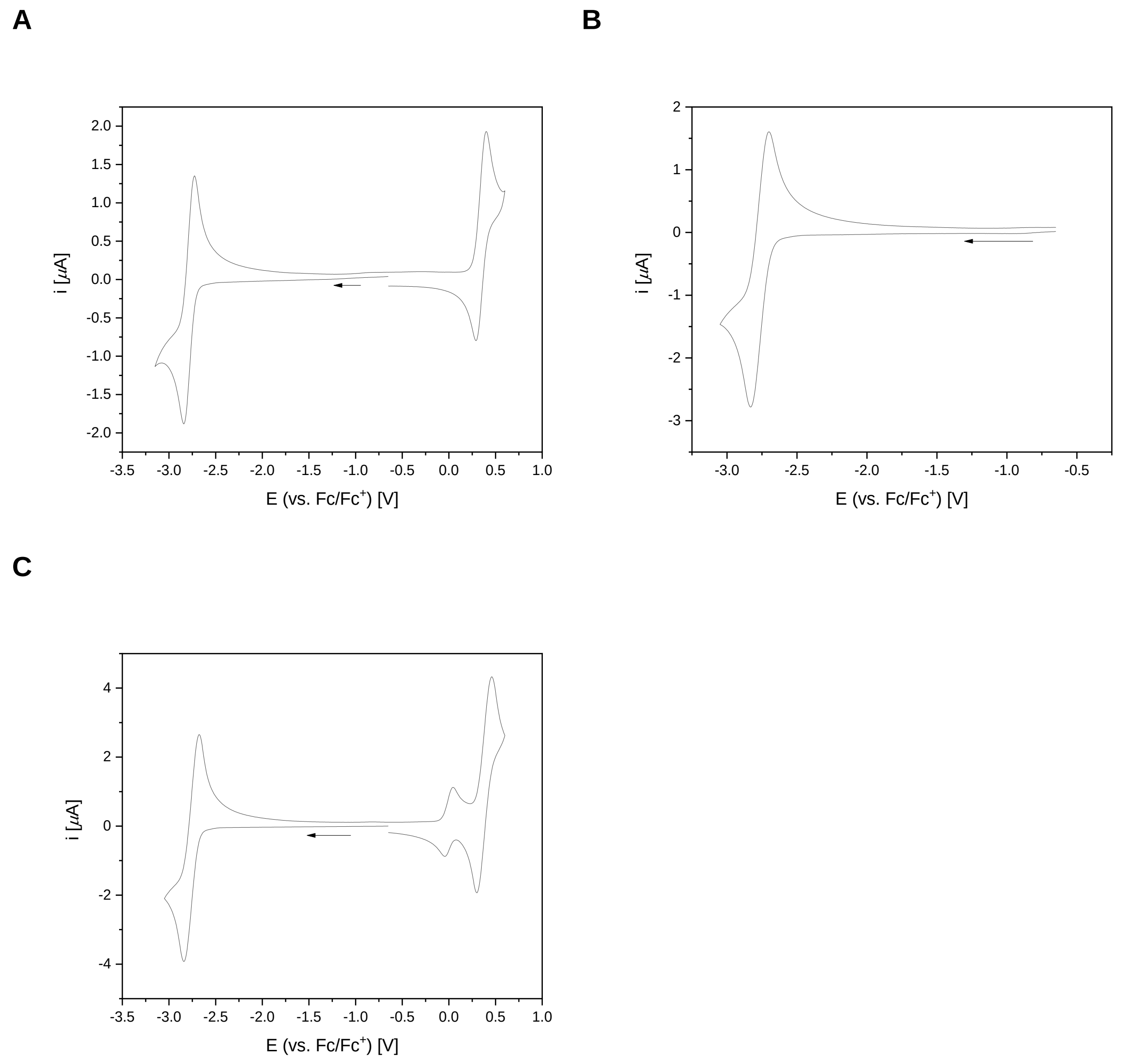

Figure S83: **A)** Cyclic voltammogram of **5a** in THF. **B)** Partially reversible reduction of **5a** in THF. **C)** Cyclic voltammogram of **5a** in THF in the presence of Fc/Fc^+.

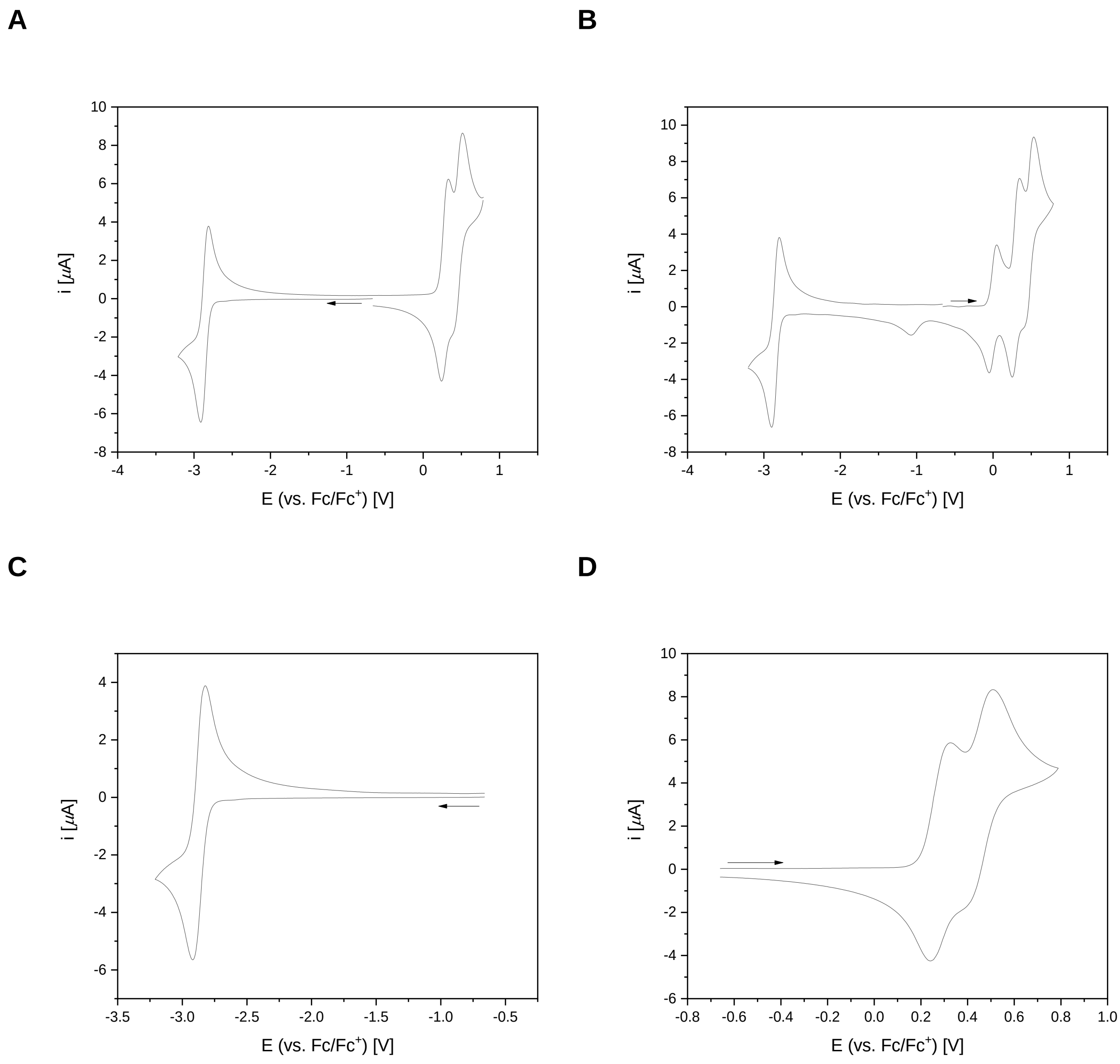

Figure S84: **A)** Cyclic voltammogram of **6a** in THF. **B)** Cyclic voltammogram of **6a** in THF in the presence of Fc/Fc^{+}. **C)** Partially reversible reduction of **6a** in THF. **D)** Reversible oxidations of **6a** in THF.

A

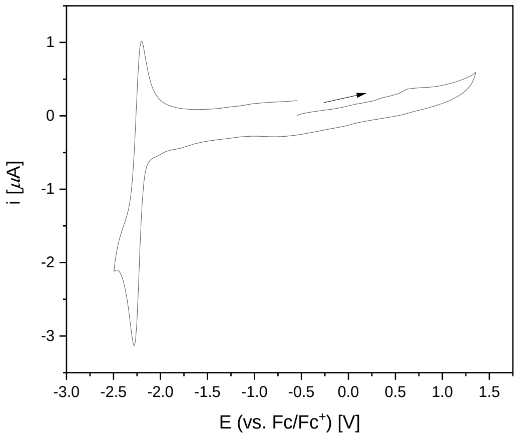

B

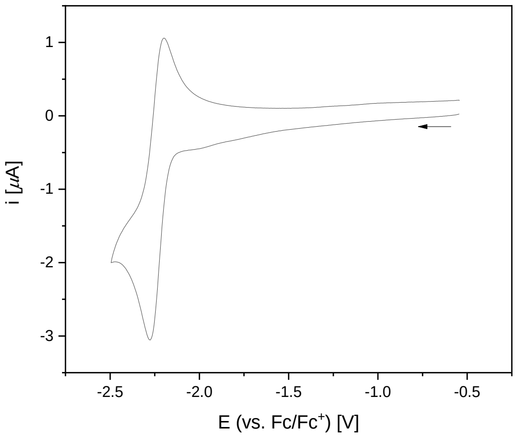

C

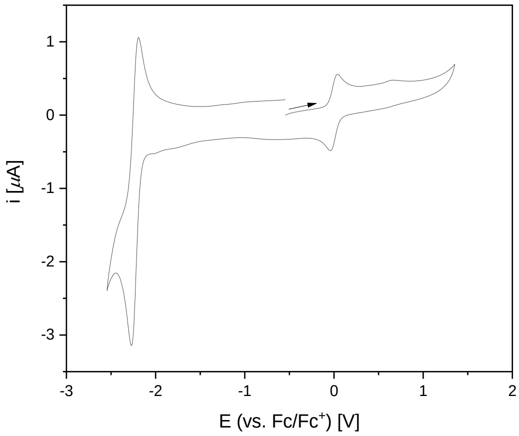

Figure S85: **A)** Cyclic voltammogram of **5c** in MeCN. **B)** Reversible reduction of **5c** in MeCN. **C)** Cyclic voltammogram of **5c** in MeCN in the presence of Fc/Fc^{+}.

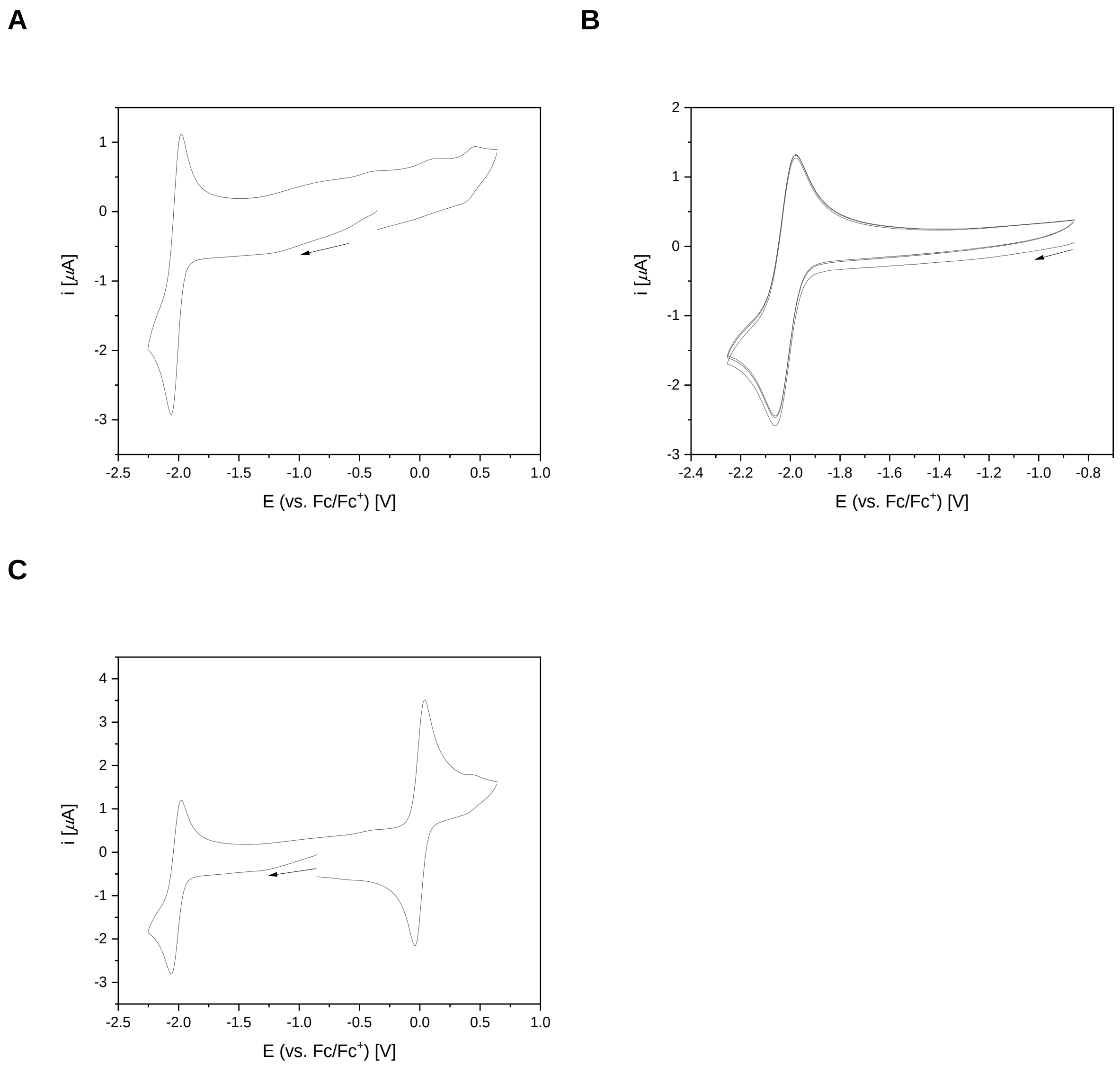

Figure S86: **A)** Cyclic voltammogram of **6c** in MeCN. **B)** Reversible reduction of **6c** in MeCN. **C)** Cyclic voltammogram of **6c** in MeCN in the presence of Fc/Fc^{+}.

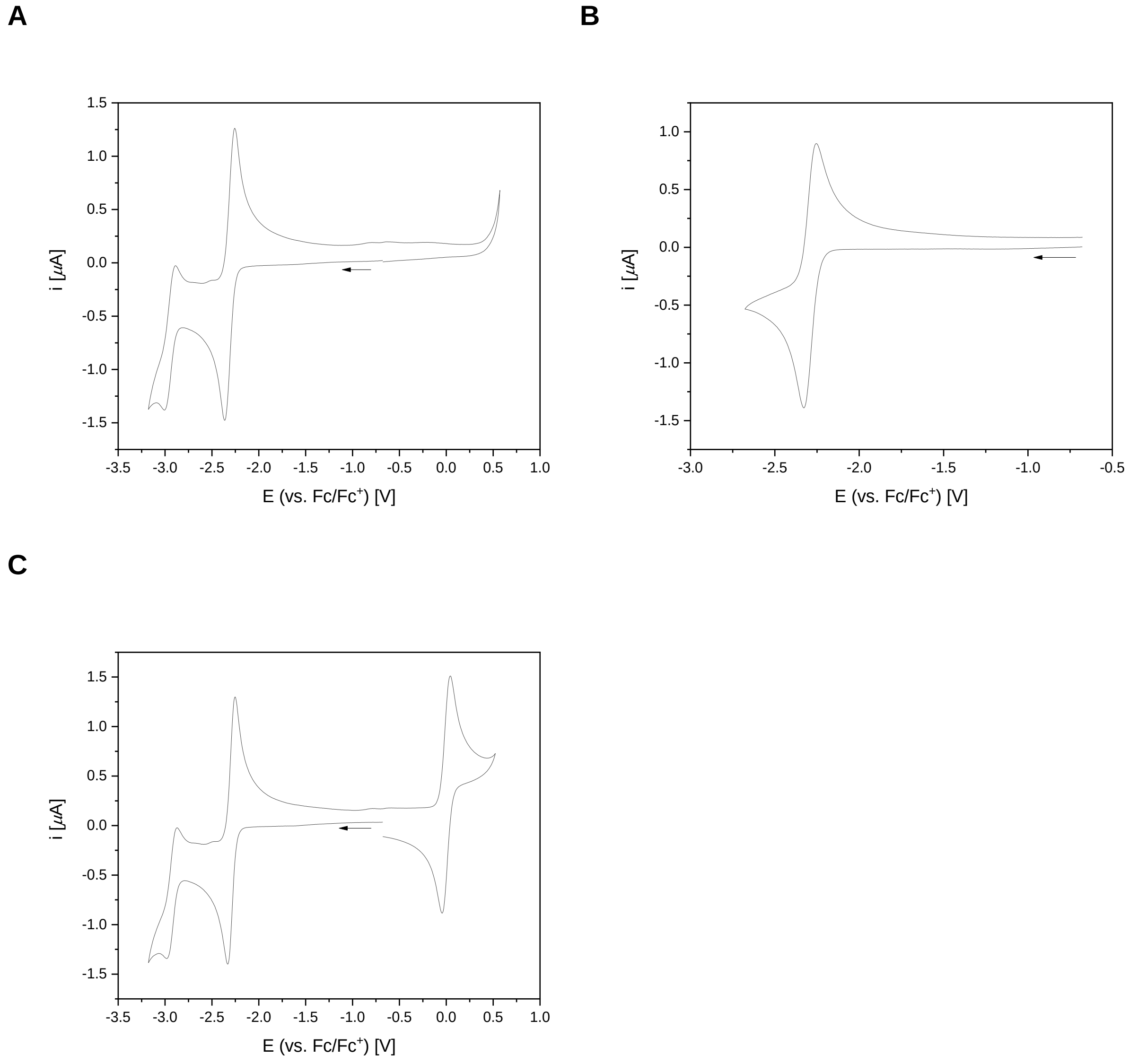

Figure S87: **A)** Cyclic voltammogram of **Neut0** in THF. **B)** Reversible Reduction of **Neut0** in THF. **C)** Cyclic voltammogram of **Neut0** in THF in the presence of Fc/Fc^{+}.

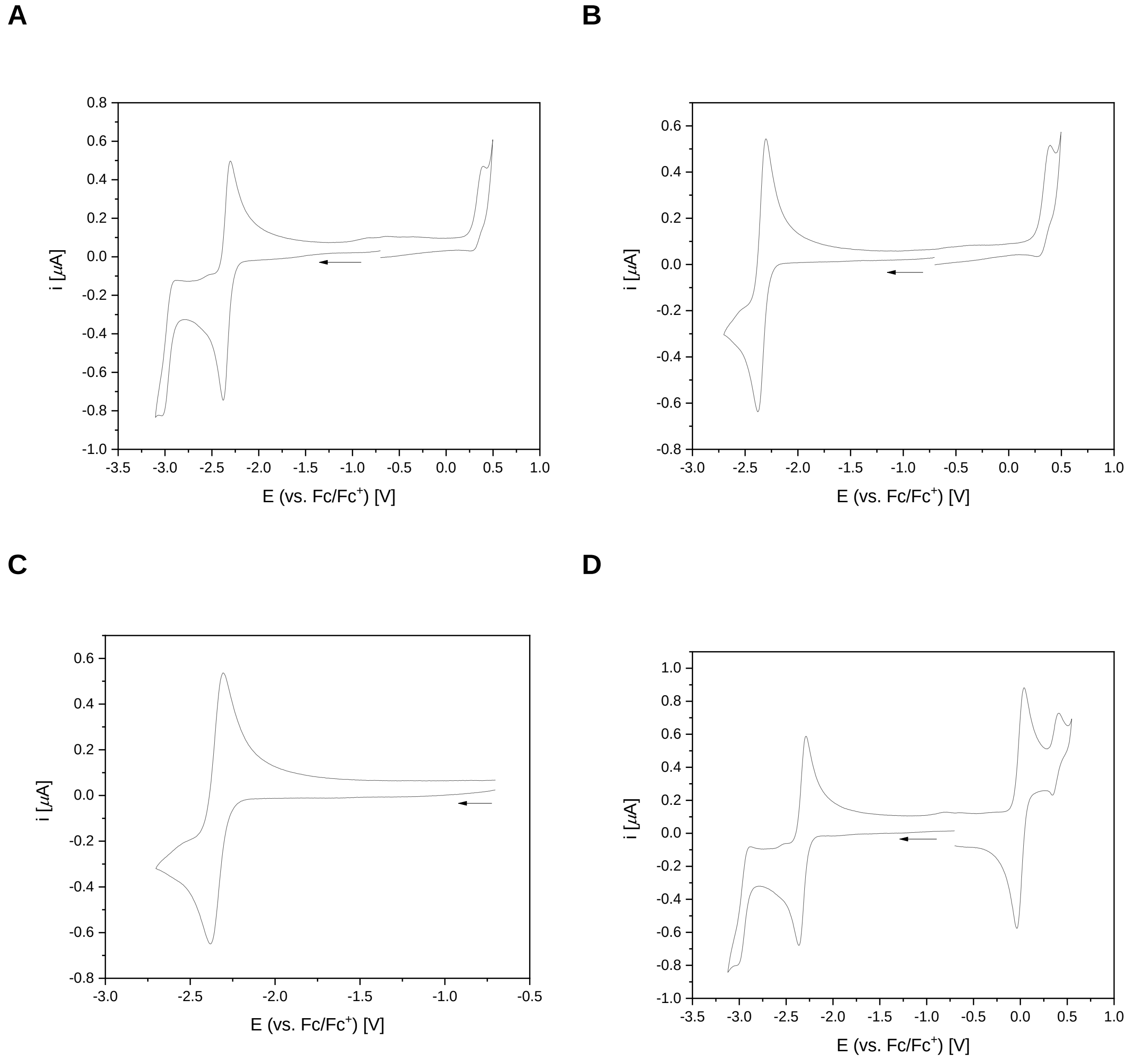

Figure S88: **A)** Cyclic voltammogram of **Neut1** in THF. **B)** First reduction and first oxidation of **Neut1** in THF. **C)** Reversible Reduction of **Neut1** in THF. **D)** Cyclic voltammogram of **Neut1** in THF in the presence of Fc/Fc$^+$.

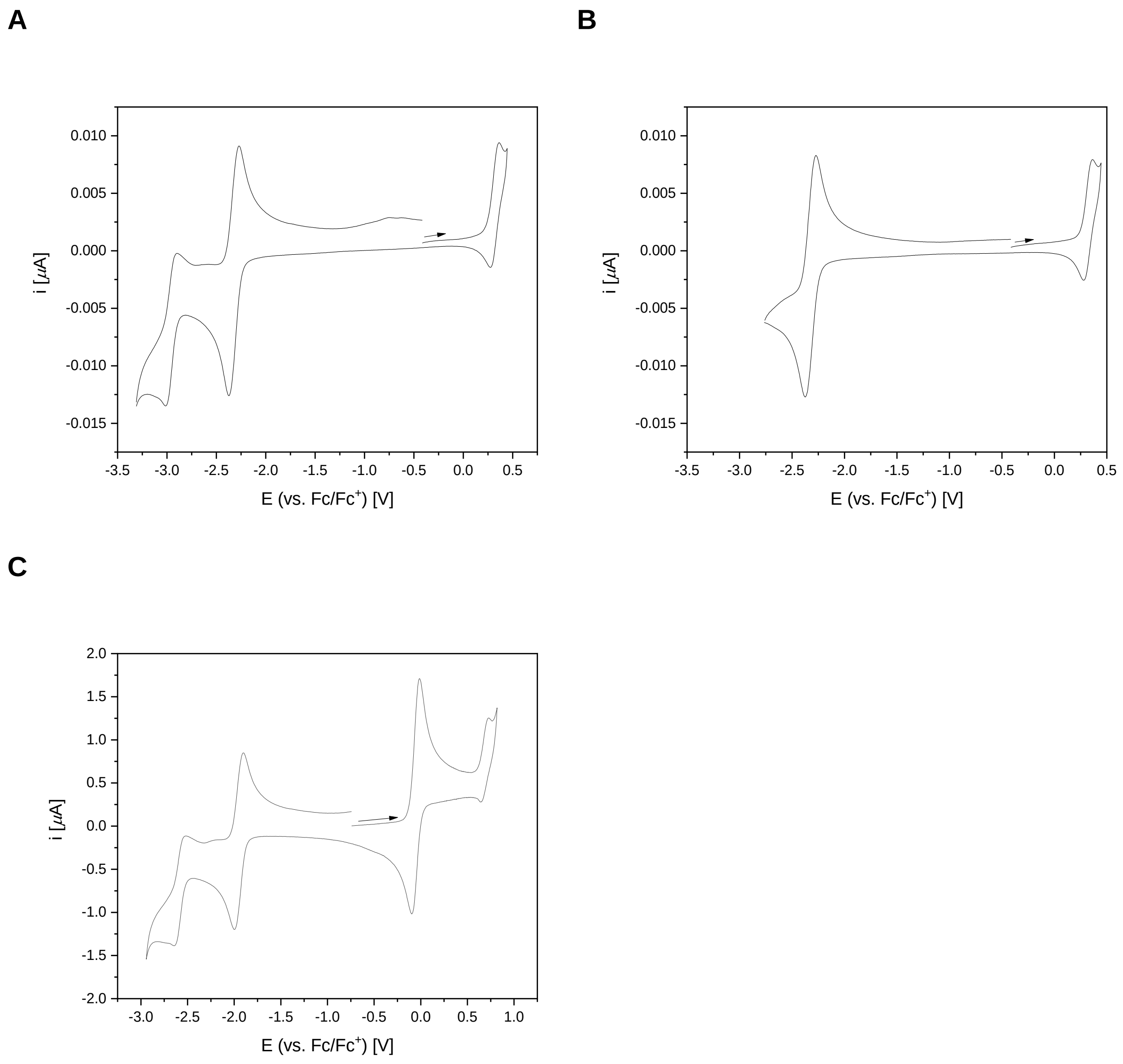

Figure S89: **A)** Cyclic voltammogram of **Neut2** in THF. **B)** First reduction and first oxidation of **Neut2** in THF. **C)** Cyclic voltammogram of **Neut2** in THF in the presence of Fc/Fc^{+}.

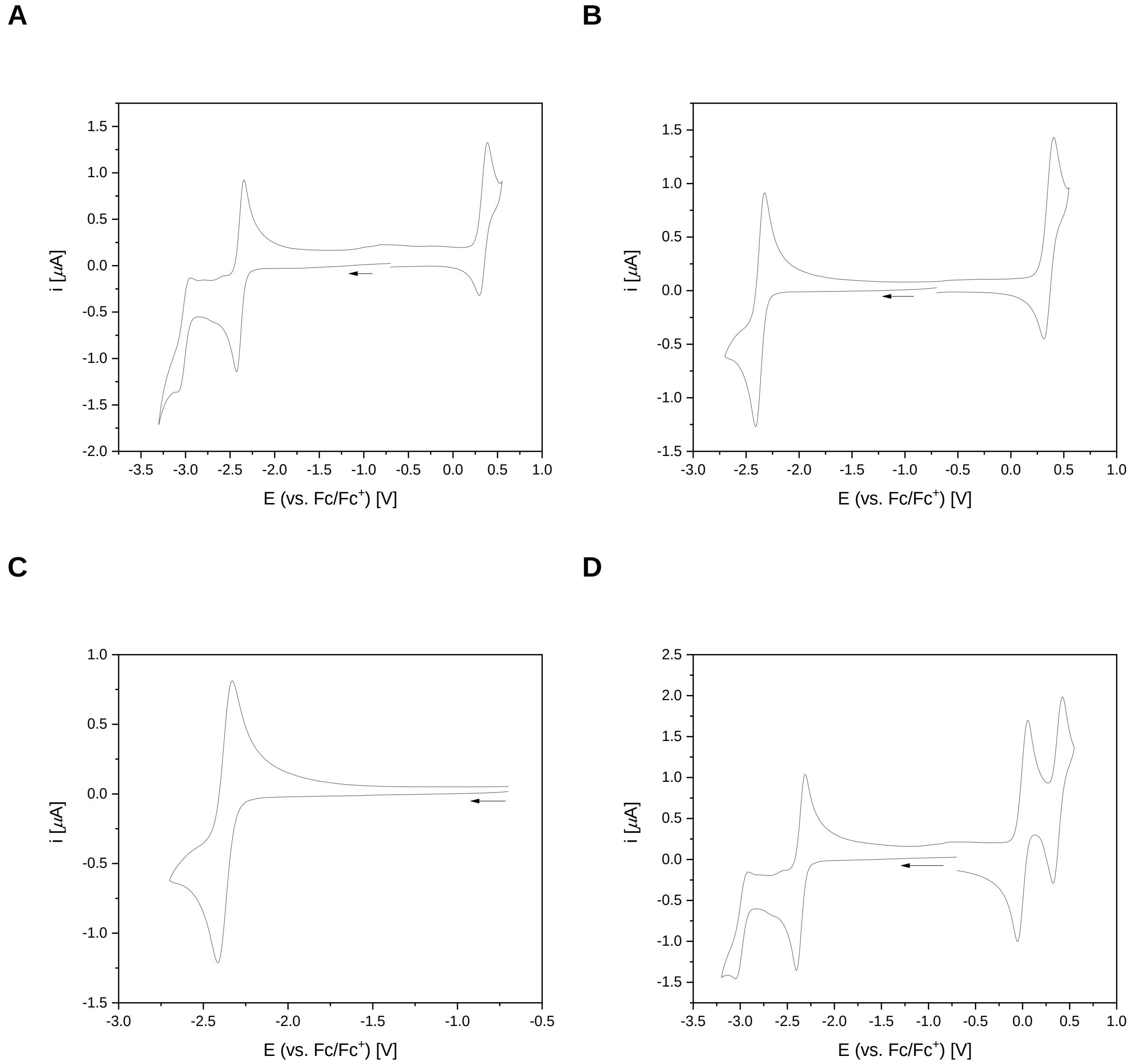

Figure S90: **A)** Cyclic voltammogram of **Neut(i)2** in THF. **B)** First reduction and first oxidation of **Neut(i)2** in THF. **C)** Reversible Reduction of **Neut(i)2** in THF. **D)** Cyclic voltammogram of **Neut(i)2** in THF in the presence of Fc/Fc^+.

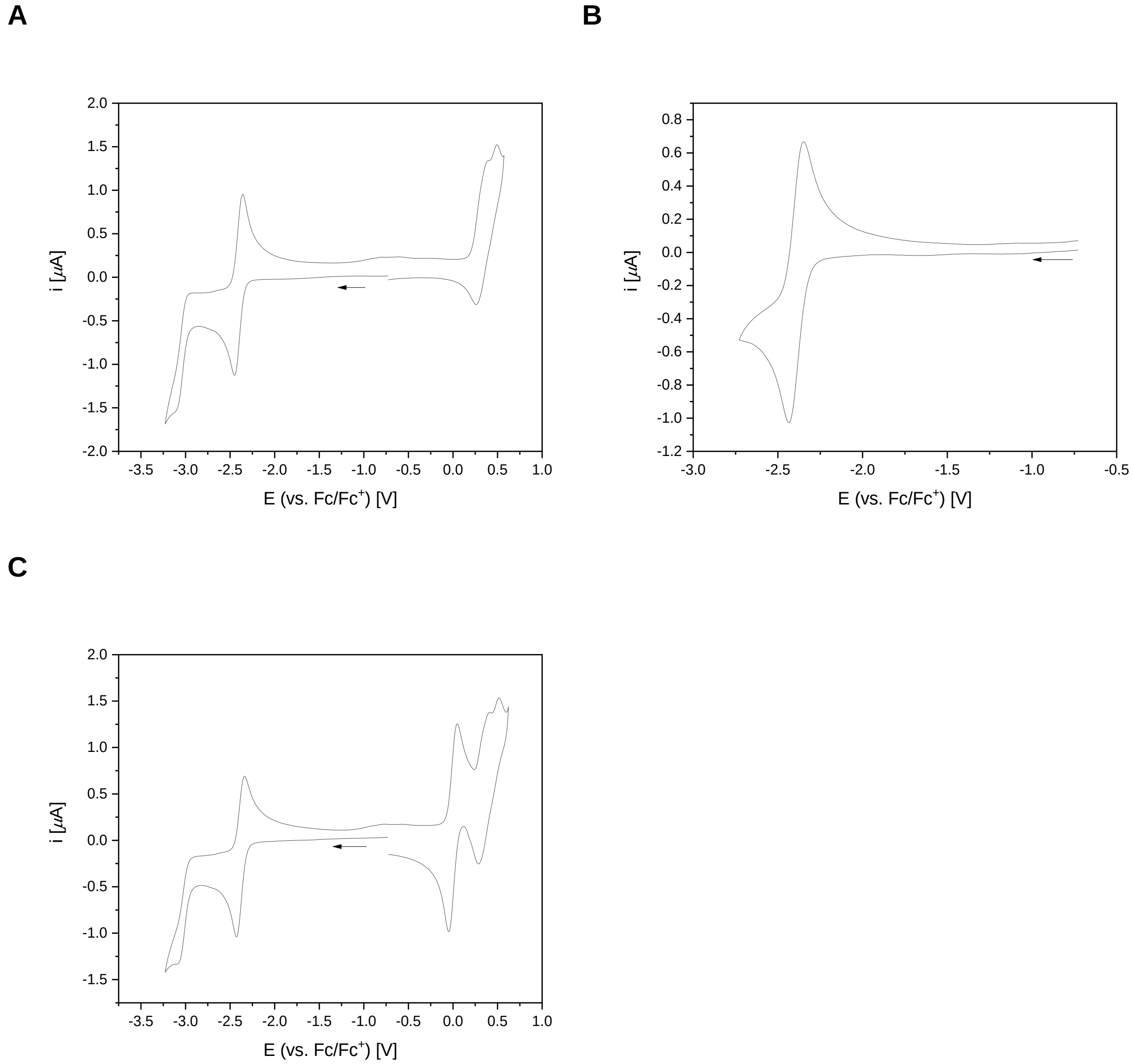

Figure S91: **A)** Cyclic voltammogram of **Neut3** in THF. **B)** Reversible reduction of **Neut3** in THF. **C)** Cyclic voltammogram of **Neut3** in THF in the presence of Fc/Fc$^+$.

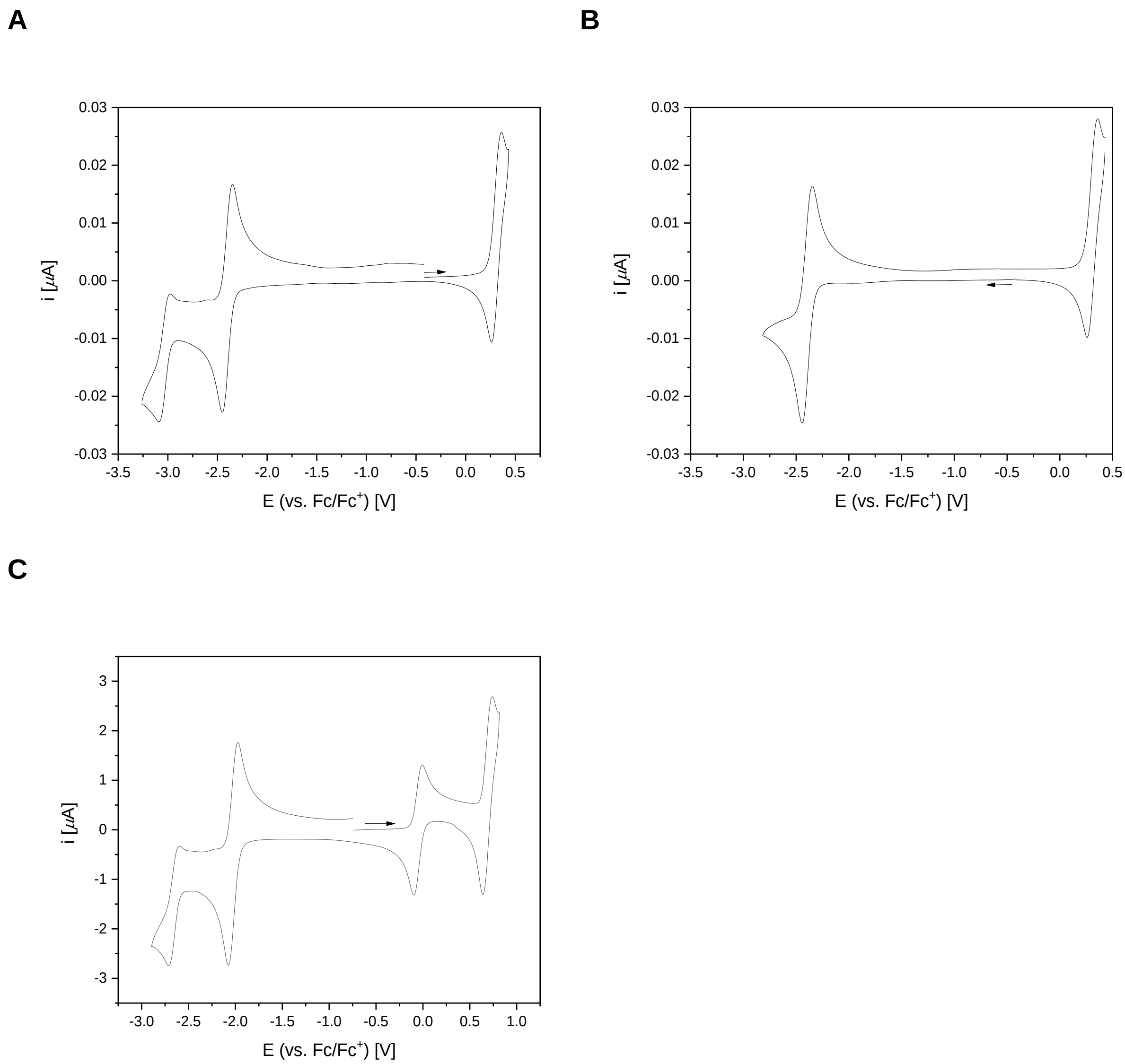

Figure S92: **A)** Cyclic voltammogram of **Neut4** in THF. **B)** First reduction and first oxidation of **Neut4** in THF. **C)** Cyclic voltammogram of **Neut4** in THF in the presence of Fc/Fc^{+}.

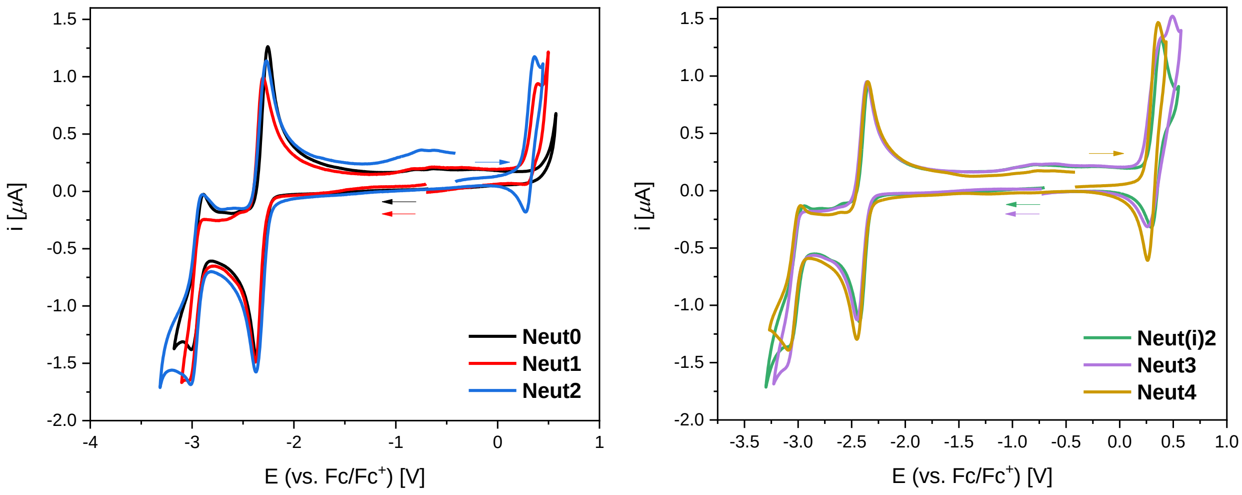

Figure S93: Cyclic voltammograms of **Neut0**-**Neut4** in THF.

Table S18: Summary of electrochemical potentials of neutral compounds determined in THF.

THF	$E_{1/2}$ (red1) [V]	$E_{1/2}$ (red2) [V]	$E_{1/2}$ (ox1) [V]	$E_{1/2}$ (ox2) [V]
**Neut0**	-2.31	-2.95 (irrev.)		
**Neut1**	-2.34	-3.03 (irrev.)	0.39 (irrev.)	
**Neut2**	-2.33	-2.96 (irrev.)	0.32 (irrev.)	
**Neut(i)2**	-2.39	-3.09 (irrev.)	0.34 (irrev.)	
**Neut3**	-2.40	-3.11 (irrev.)	0.40 (irrev.)	0.49 (irrev.)
**Neut4**	-2.40	-3.03 (irrev.)	0.32 (irrev.)	
**4a**	-2.67			
**5a**	-2.78		0.34	
**6a**	-2.86		0.28	0.51 (irrev.)

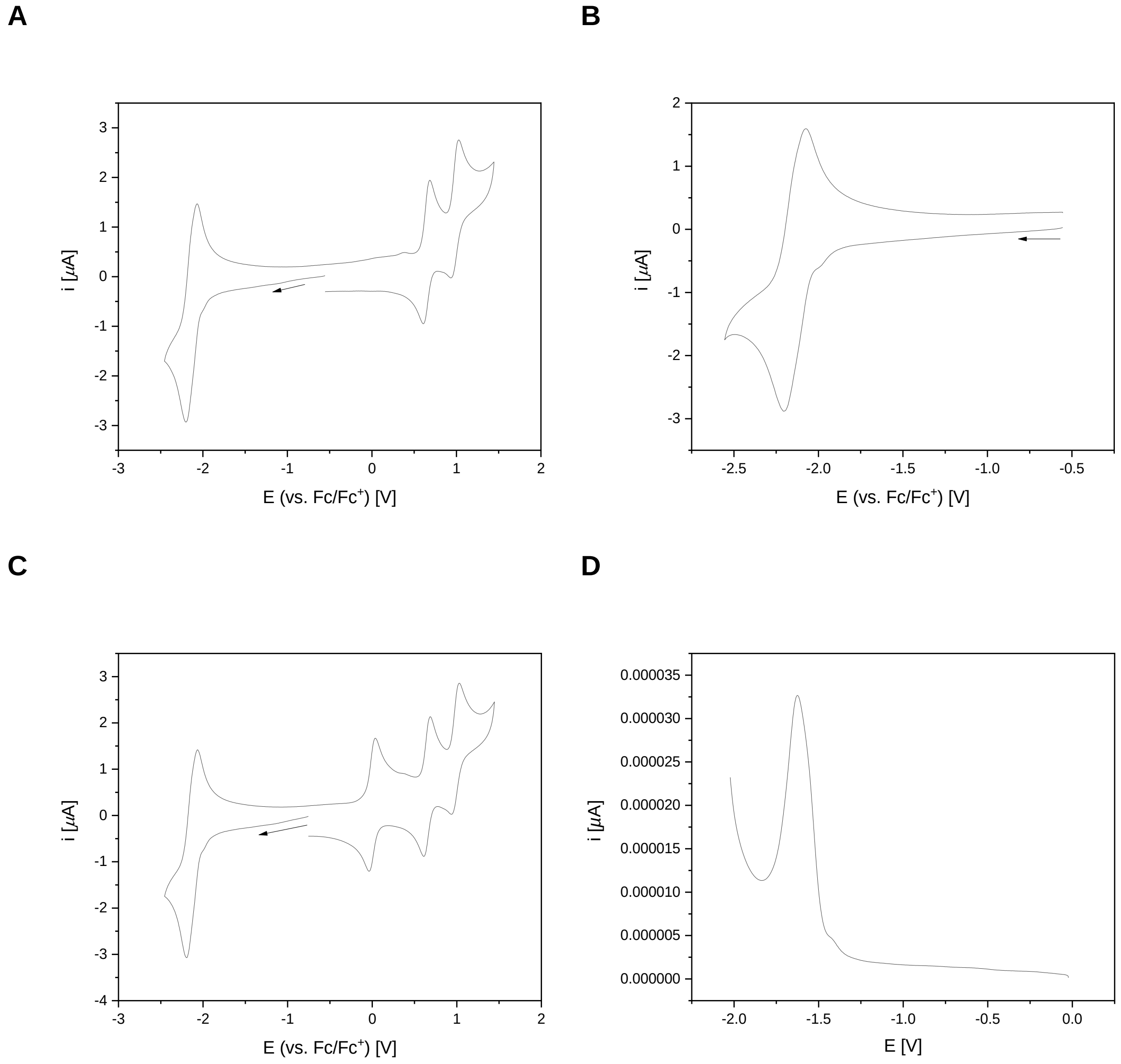

Figure S94: **A)** Cyclic voltammogram of **Cat^{1+}** in MeCN. **B)** Reversible reduction of **Cat^{1+}** in MeCN. **C)** Cyclic voltammogram of **Cat^{1+}** in MeCN in the presence of Fc/Fc^{+}. **D)** Square wave experiment for **Cat^{1+}** in THF.

A

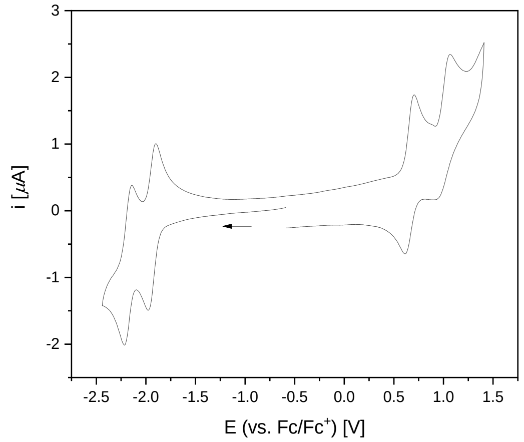

B

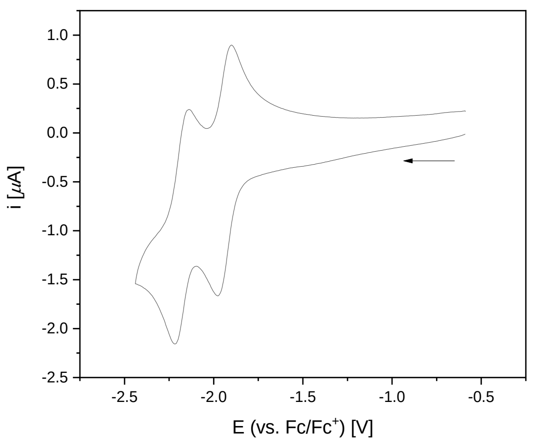

C

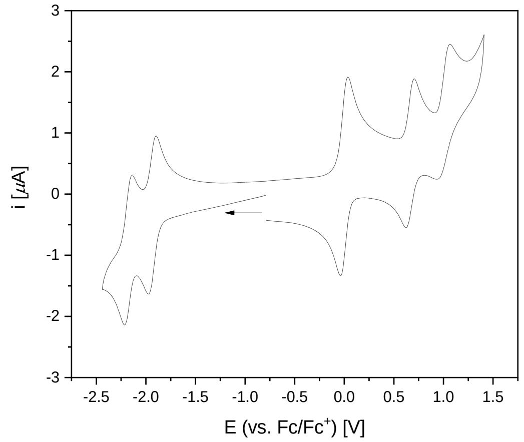

Figure S95: **A)** Cyclic voltammogram of **Cat^{2+}** in MeCN. **B)** Reversible reduction of **Cat^{2+}** in MeCN. **C)** Cyclic voltammogram of **Cat^{2+}** in MeCN in the presence of Fc/Fc^{+}.

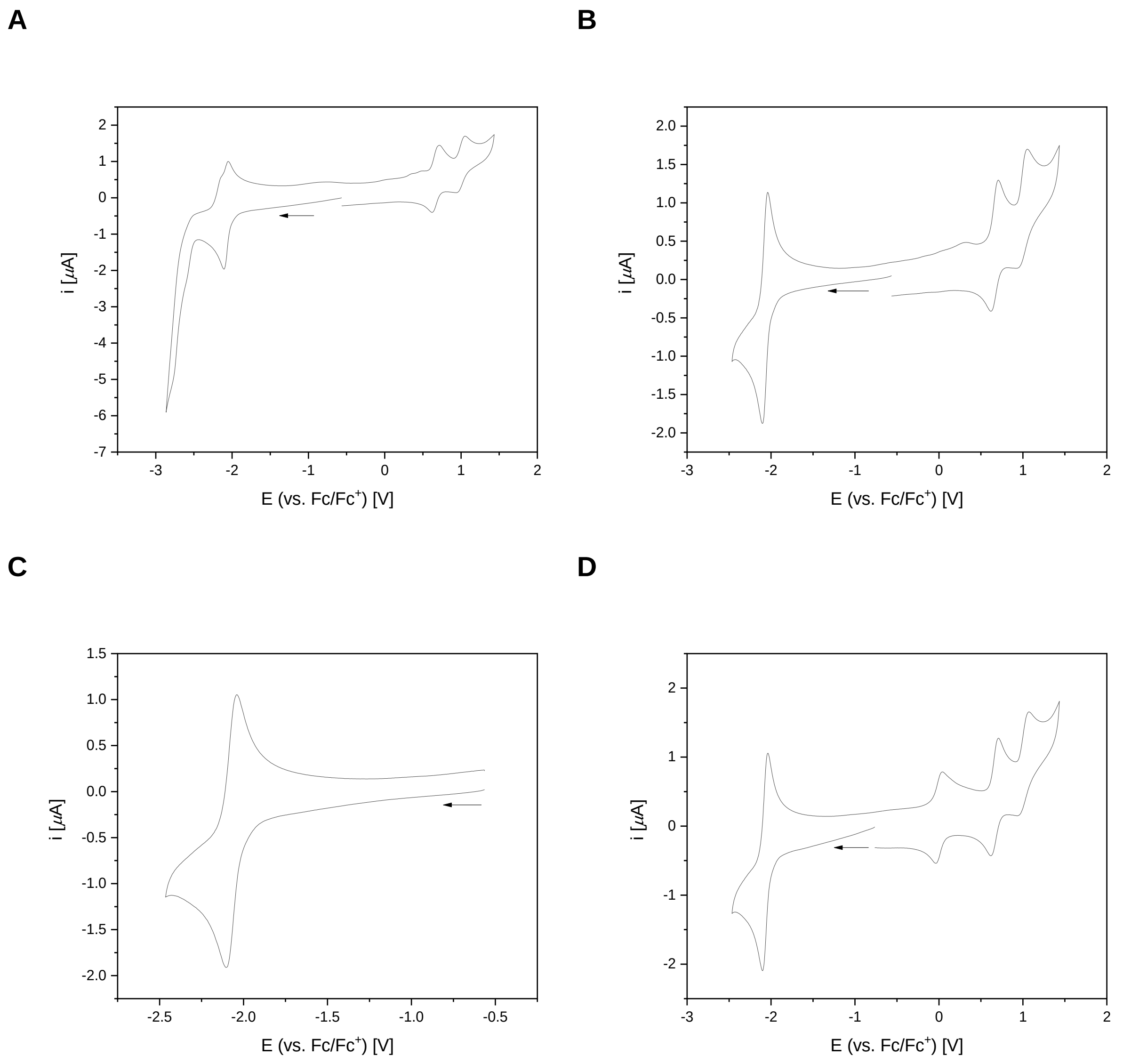

Figure S96: **A)** Cyclic voltammogram of **Cat(i)$^{2+}$** in MeCN. **B)** Cyclic voltammogram of **Cat(i)$^{2+}$** in MeCN without irreversible reduction. **C)** Reversible reduction of **Cat(i)$^{2+}$** in MeCN. **D)** Cyclic voltammogram of **Cat(i)$^{2+}$** in MeCN in the presence of Fc/Fc^{+}.

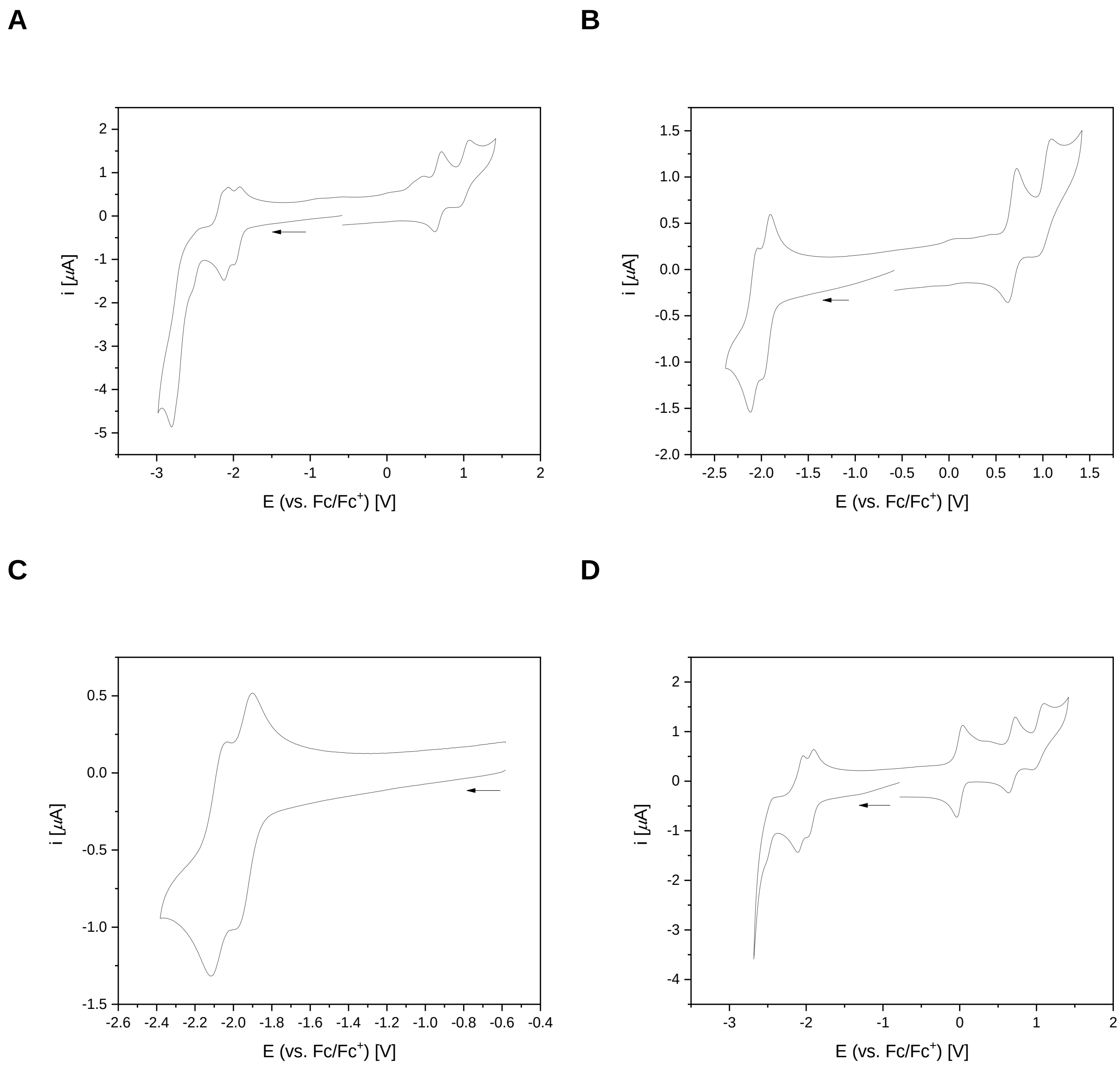

Figure S97: **A)** Cyclic voltammogram of **Cat^{3+}** in MeCN. **B)** Cyclic voltammogram of **Cat^{3+}** in MeCN without irreversible reduction. **C)** Reversible reduction of **Cat^{3+}** in MeCN. **D)** Cyclic voltammogram of **Cat^{3+}** in MeCN in the presence of Fc/Fc$^+$.

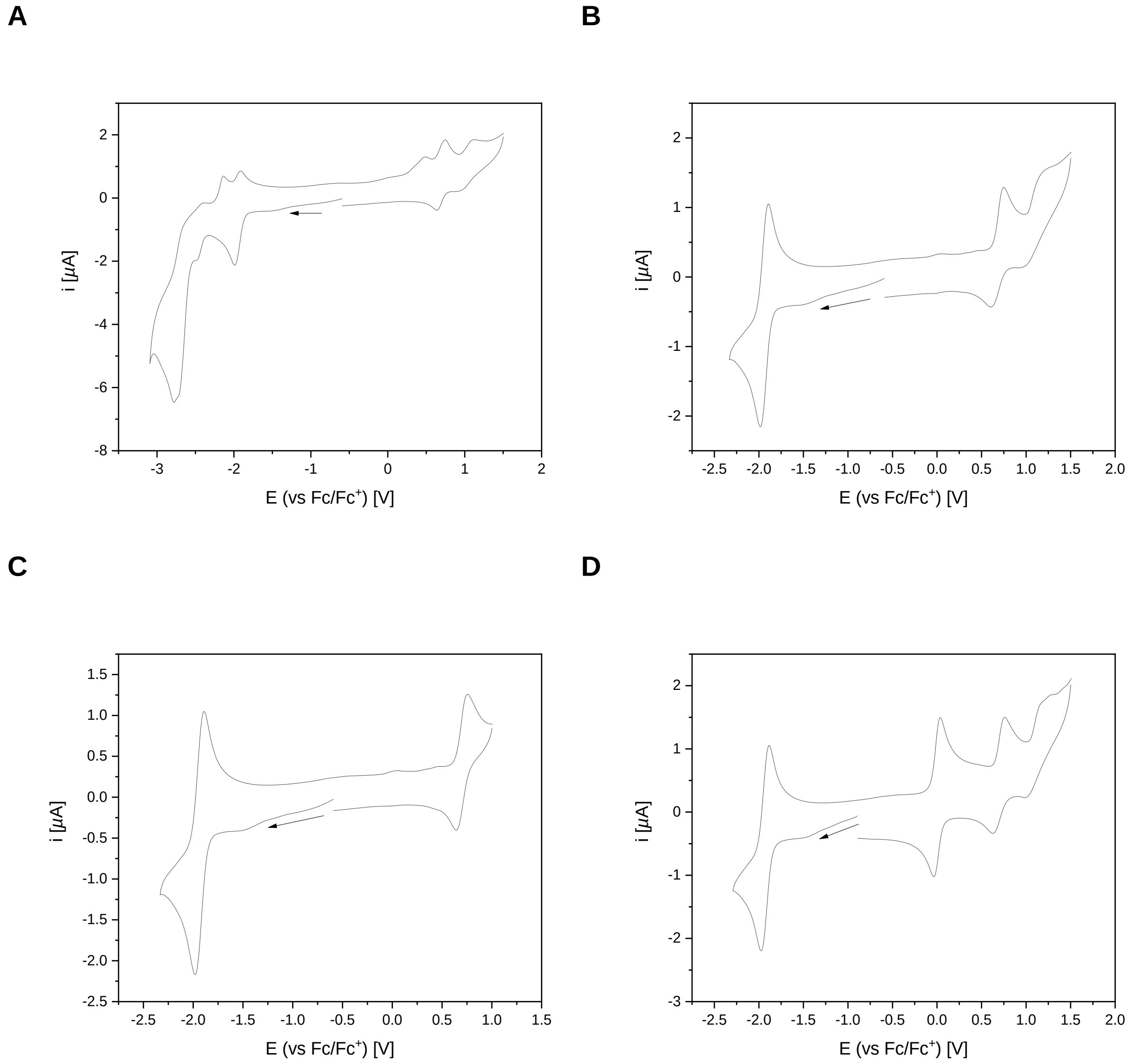

Figure S98: **A)** Cyclic voltammogram of **Cat**$^{4+}$ in MeCN. **B)** Cyclic voltammogram of **Cat**$^{4+}$ in MeCN without irreversible reduction. **C)** Reversible reduction and oxidation of **Cat**$^{4+}$ in MeCN. **D)** Cyclic voltammogram of **Cat**$^{4+}$ in MeCN in the presence of Fc/Fc^{+}.

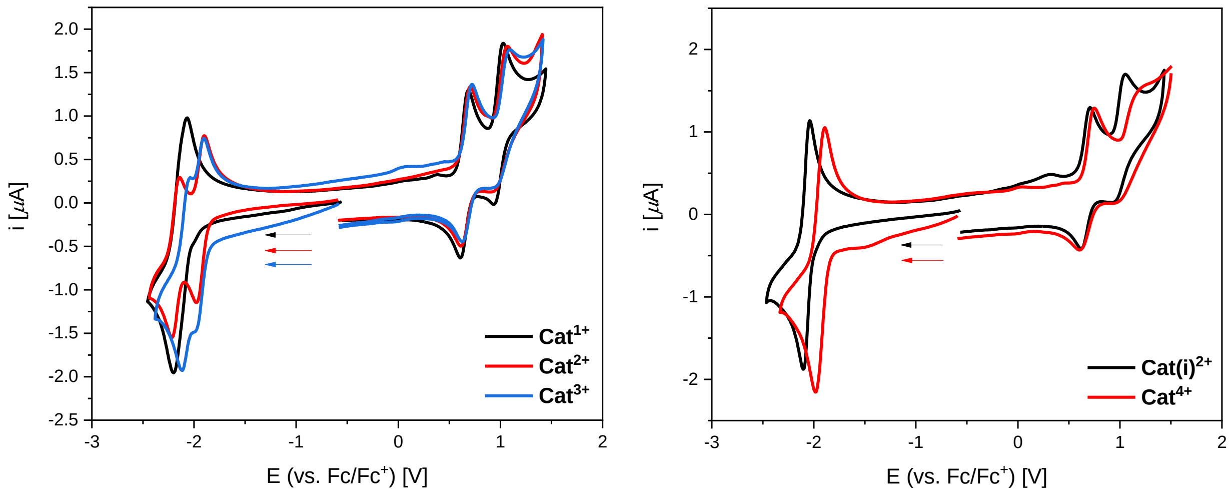

Figure S99: Cyclic voltammograms of **Cat^{1+}**-**Cat^{4+}** in MeCN.

Table S19: Summary of electrochemical potentials of cationic compounds determined in MeCN.

MeCN	$E_{1/2}$ (red1) [V]	$E_{1/2}$ (red2) [V]	$E_{1/2}$ (red3) [V]	$E_{1/2}$ (ox1) [V]	$E_{1/2}$ (ox2) [V]
**Cat^{1+}**	-2.13			0.64	0.98
**Cat^{2+}**	-1.94	-2.18		0.66	0.99
**Cat(i)$^{2+}$**	-2.07	-2.76 (irrev.)		0.66	0.98
**Cat^{3+}**	-1.94	-2.07	-2.80 (irrev.)	0.67	1.01
**Cat^{4+}**	-1.93	-2.78 (irrev.)		0.68	1.10
**5c**	-2.24				
**6c**	-2.02				

The redox couple Fc/Fc$^+$ is known to be a fully reversible 1e$^-$ redox couple. Thus, the peak-to-peak splitting (Table S20) is almost the same for all measurements. As a result, when comparing the peak-to-peak splitting values of this couple with the values obtained for the other triarylboranes and *bis*-triarylboranes, very similar values are found for all measurements. Thus, it can be assumed that each of these processes are 1e$^-$ processes.

Table S20: Summary of peak-to-peak splitting for all compounds.

Compound	Peak-to-peak splitting compound [V]	Peak-to-peak splitting Fc/Fc^+ [V]
**4a**	0.215	0.105
**5a**	Red: 0.165<br>Ox: 0.160	0.090
**6a**	Red: 0.100<br>Ox1: 0.095<br>Ox2: 0.115	0.100
**5c**	0.080	0.080
**6c**	0.080	0.080
**Neut0**	Rev red: 0.080<br>Irrev red: 0.125	0.085
**Neut1**	Rev red: 0.075<br>Irrev red: 0.135<br>Ox: 0.070	0.075
**Neut2**	Rev red: 0.100<br>Irrev red: 0.125<br>Ox: 0.090	0.109
**Neut(i)2**	Rev red: 0.095<br>Irrev red: 0.140<br>Ox: 0.100	0.110
**Neut3**	Rev red: 0.090<br>Irrev red: 0.205<br>Ox1: 0.125<br>(Ox2: 0.230)	0.095
**Neut4**	Rev red: 0.100<br>Irrev red: 0.115<br>Ox: 0.100	0.109
**Cat^{1+}**	Red: 0.130<br>Ox1: 0.075<br>Ox2: 0.095	0.075
**Cat^{2+}**	Red1: 0.075<br>Red2: 0.085<br>Ox1: 0.085<br>Ox2: 0.130	0.070
**$Cat(i)^{2+}$**	Red: 0.065<br>Ox1: 0.090<br>Ox2: 0.150	0.080
**Cat^{3+}**	Red1: 0.075<br>Red2: 0.070<br>Ox1: 0.090<br>Ox2: 0.165	0.080
**Cat^{4+}**	Red: 0.085<br>Ox1: 0.130<br>Ox2: 0.210	0.075

Studies in Buffered Solutions

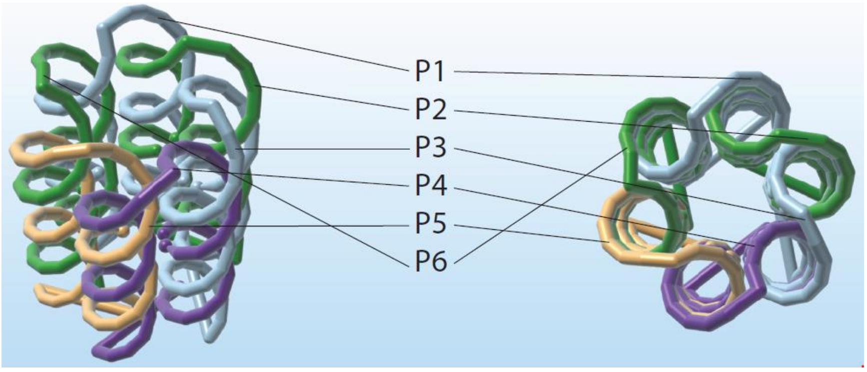

Figure S100: Schematic representation of DNApore.

Table S21: Composition of oligonucleotides P1-P6 used for synthesis of DNApore.

P1	AGCGAACGTGGATTTTGTCCGACATCGGCAAGCTCCCTTTTTCGACTATT
P2	CCGATGTCGGACTTTTACACGATCTTCGCCTGCTGGGTTTTGGGAGCTTG
P3	CGAAGATCGTGTTTTTCCACAGTTGATTGCCCTTCACTTTTCCCAGCAGG
P4	AATCAACTGTGGTTTTTCTCACTGGTGATTAGAATGCTTTTGTGAAGGGC
P5	TCACCAGTGAGATTTTTGTCGTACCAGGTGCATGGATTTTTGCATTCTAA
P6	CCTGGTACGACATTTTTCCACGTTCGCTAATAGTCGATTTTATCCATGCA

Physico-hemical Properties of **Cat^{1+}**-**Cat^{3+}** in Buffered Solution

Solubility in Sodium Cacodylate at pH 7

DMSO stock solutions of **Cat^{1+}**, **Cat^{2+}**, **Cat(i)$^{2+}$** and **Cat^{3+}** were diluted with sodium cacodylate solutions (pH 7, I = 0.05 M) to concentrations of ca. 2 x 10^{-5} – 8 x 10^{-6} M. The corresponding absorption maxima and molar extinction coefficients were found to be very similar for buffered solutions containing small amounts of DMSO and water solutions containing max. 1% acetonitrile (Table S22). The same is true for the respective emission spectra. However, upon heating, the emission of **Cat^{1+}**, **Cat^{2+}**, and **Cat^{3+}** is quenched by ca. 40-50%. In contrast, the emission of **Cat(i)$^{2+}$** increases by ca. 15% upon heating. After cooling to room temperature, the emission of **Cat^{2+}** and **Cat^{3+}** was restored, while the spectra of **Cat^{1+}** and **Cat(i)$^{2+}$** were not.

Stability of UV/Vis Spectra

The UV/Vis spectra of examined compounds were recorded in sodium cacodylate buffer (c = 0.05 M) at pH 7 (Figure S101-Figure S104). Absorbancies of aqueous solutions of the

compounds were proportional to their concentrations up to concentrations c = 2 × 10^{-5} M. The absorption maxima and the corresponding molar extinction coefficients are given in Table S22.

Table S22: Absorption maxima and molar extinction coefficient of **Cat^{1+}-Cat^{3+}** determined in 1% MeCN in water and DMSO in sodium cacodylate.

	DMSO in sodium cacodylate		1% MeCN in water	
	λ_{max}^{abs} [nm]	ε [L mol^{-1} cm^{-1}]	λ_{max}^{abs} [nm]	ε [L mol^{-1} cm^{-1}]
**Cat^{1+}**	414	33 300 ±600	415	29 500 ±1 200
**Cat^{2+}**	422	37 700 ±900	424	31 100 ±300
**Cat(i)$^{2+}$**	417	30 800 ±200	424	34 000 ±1 500
**Cat^{3+}**	421	30 900 ±700	423	36 000 ±900

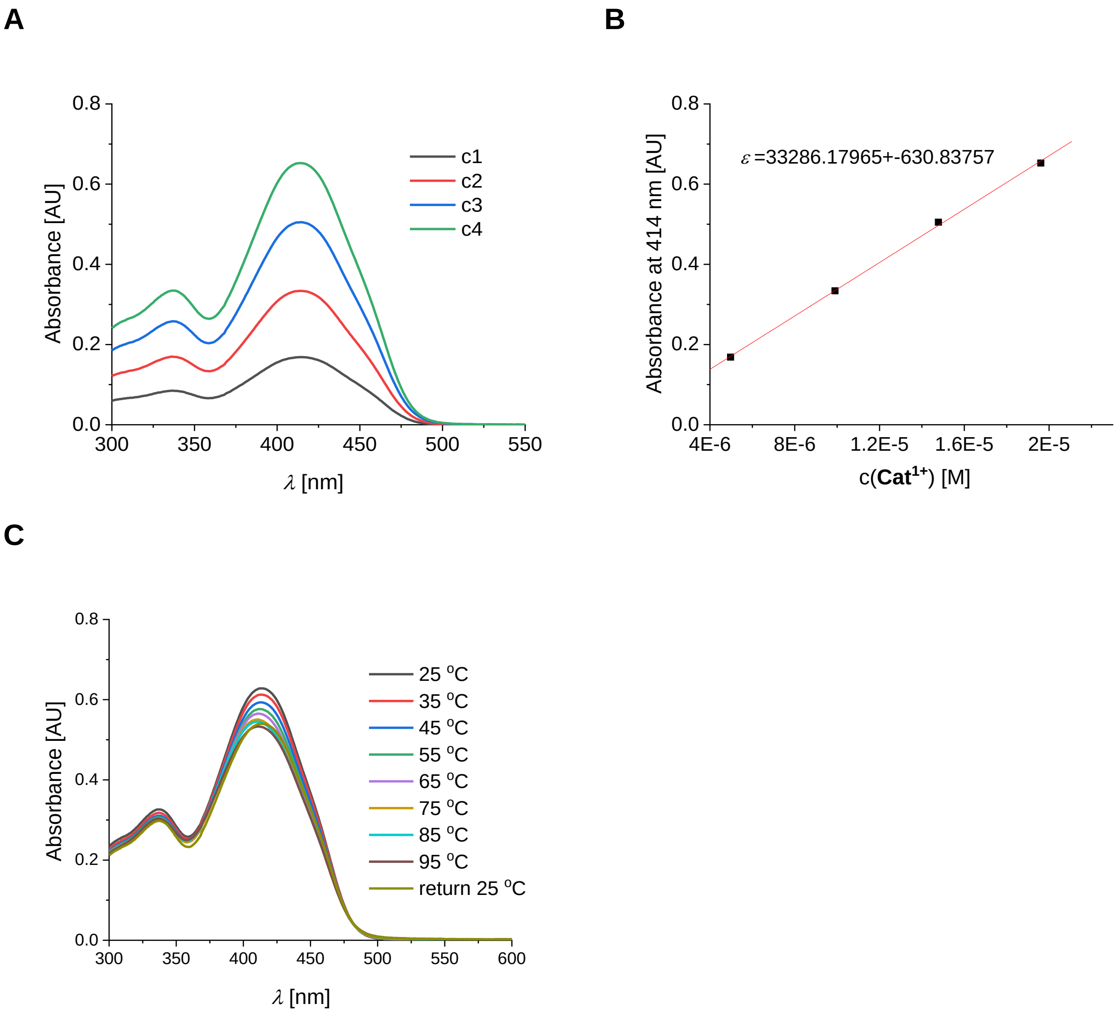

Figure S101: **A)** UV/Vis spectra of **Cat^{1+}** (c = 4.9 × 10^{-6} – 2 × 10^{-5} M). **B)** Linear dependence (—) of the absorbance at 414 nm (■) on the concentration of **Cat^{1+}**. **C)** Temperature dependence (T = 25 °C – 95 °C) of UV/Vis spectra (c = 2 × 10^{-5} M; sodium cacodylate buffer, pH = 7, *I* = 0.05 M).

A

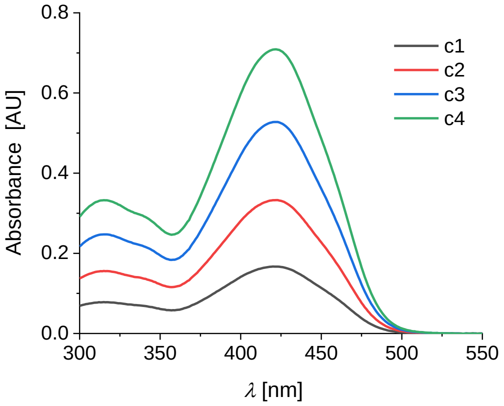

B

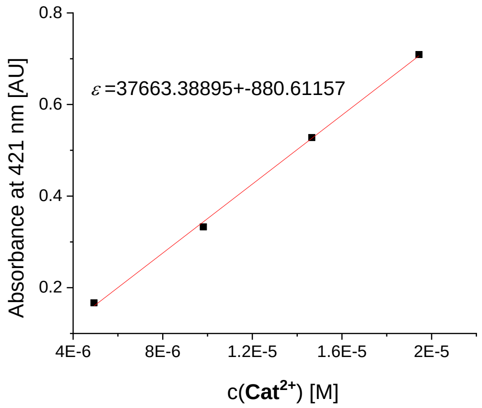

C

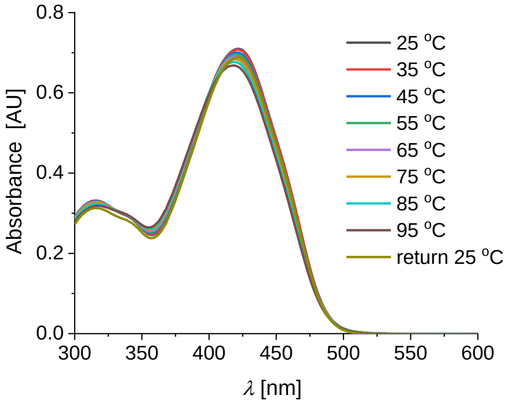

Figure S102: **A)** UV/Vis spectra of **Cat^{2+}** (c = 4.9 × 10^{-6} – 2 × 10^{-5} M). **B)** Linear dependence (—) of the absorbance at 422 nm (■) on the concentration of **Cat^{2+}**. **C)** Temperature dependence (T = 25 °C – 95 °C) of the UV/Vis spectra (c = 2 × 10^{-5} M; sodium cacodylate buffer, pH = 7, *I* = 0.05 M).

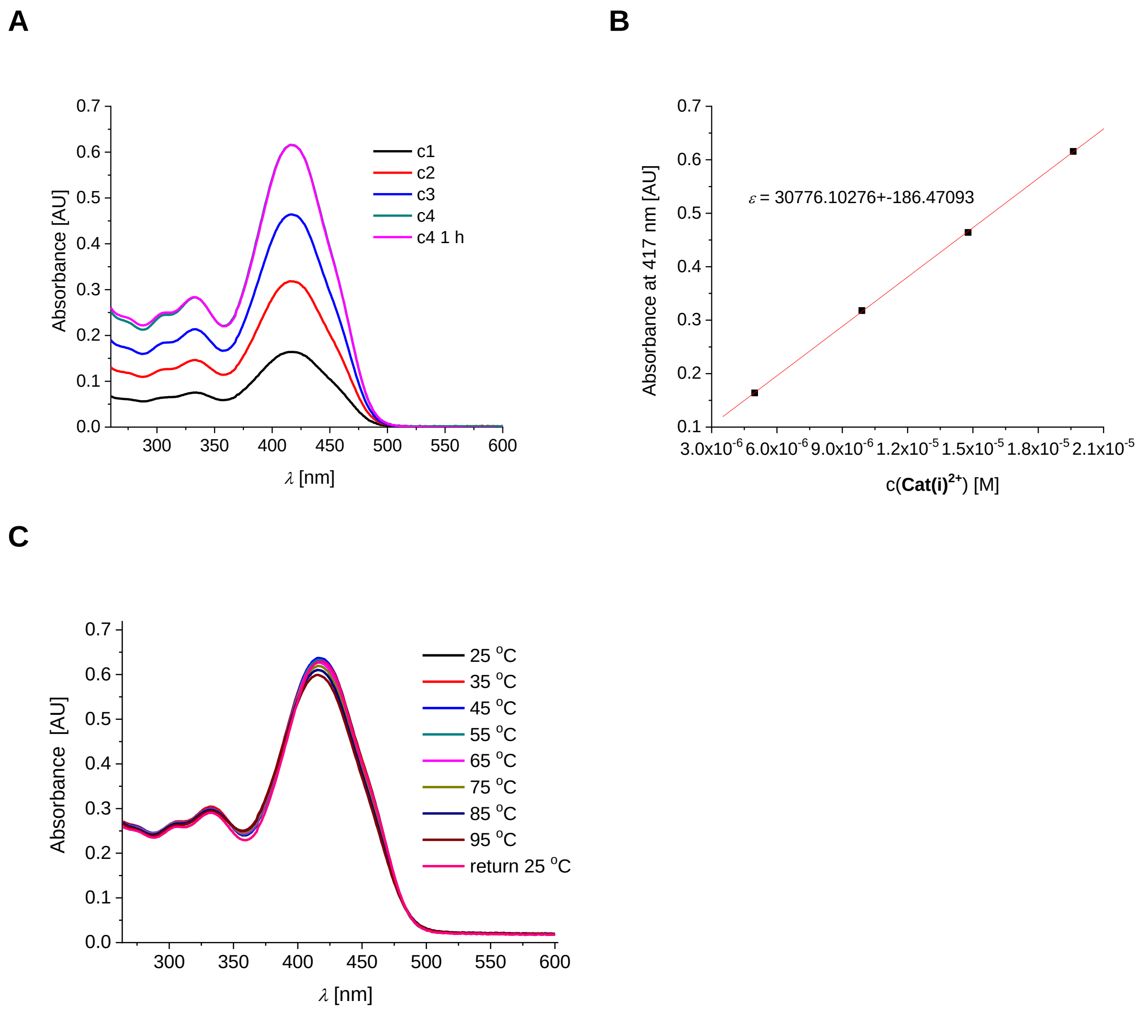

Figure S103: **A)** UV/Vis spectra of **Cat(i)**$^{2+}$ (c = 5 × 10^{-6} – 2 × 10^{-5} M). **B)** Linear dependence (—) of the absorbance at 417 nm (■) on the concentration of **Cat(i)**$^{2+}$. **C)** Temperature dependence (T = 25 °C – 95 °C) of UV/Vis spectra (c = 2 × 10^{-5} M; sodium cacodylate buffer, pH = 7, *I* = 0.05 M).

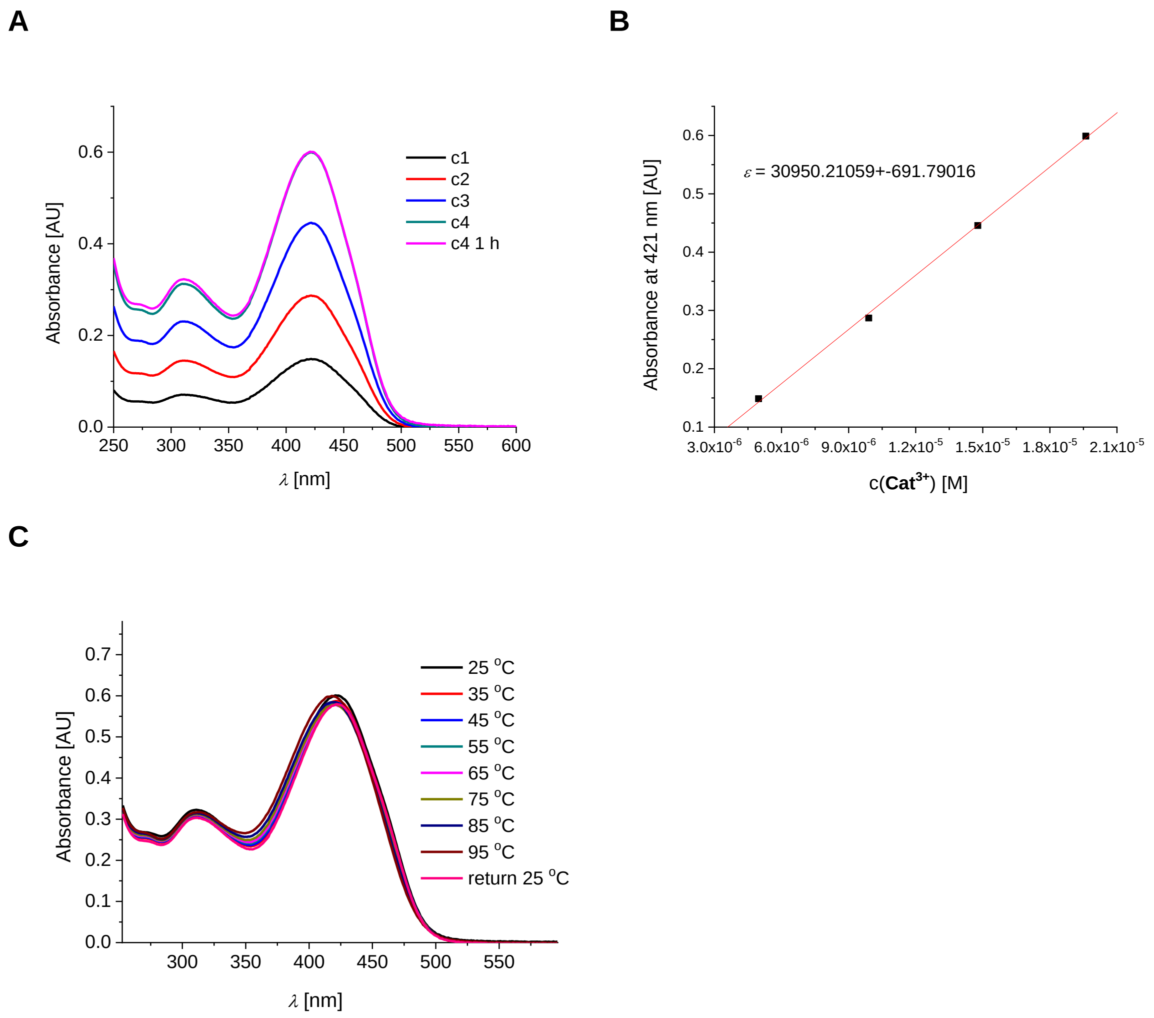

Figure S104: **A)** UV/Vis spectra of **Cat^{3+}** (c = 5 × 10^{-6} – 2 × 10^{-5} M). **B)** Linear dependence (—) of the absorbance at 421 nm (■) on the concentration of **Cat^{3+}. C)** Temperature dependence (T = 25 °C – 95 °C) of UV/Vis spectra (c = 2 × 10^{-5} M; sodium cacodylate buffer, pH = 7, *I* = 0.05 M).

Fluorimetric Spectra

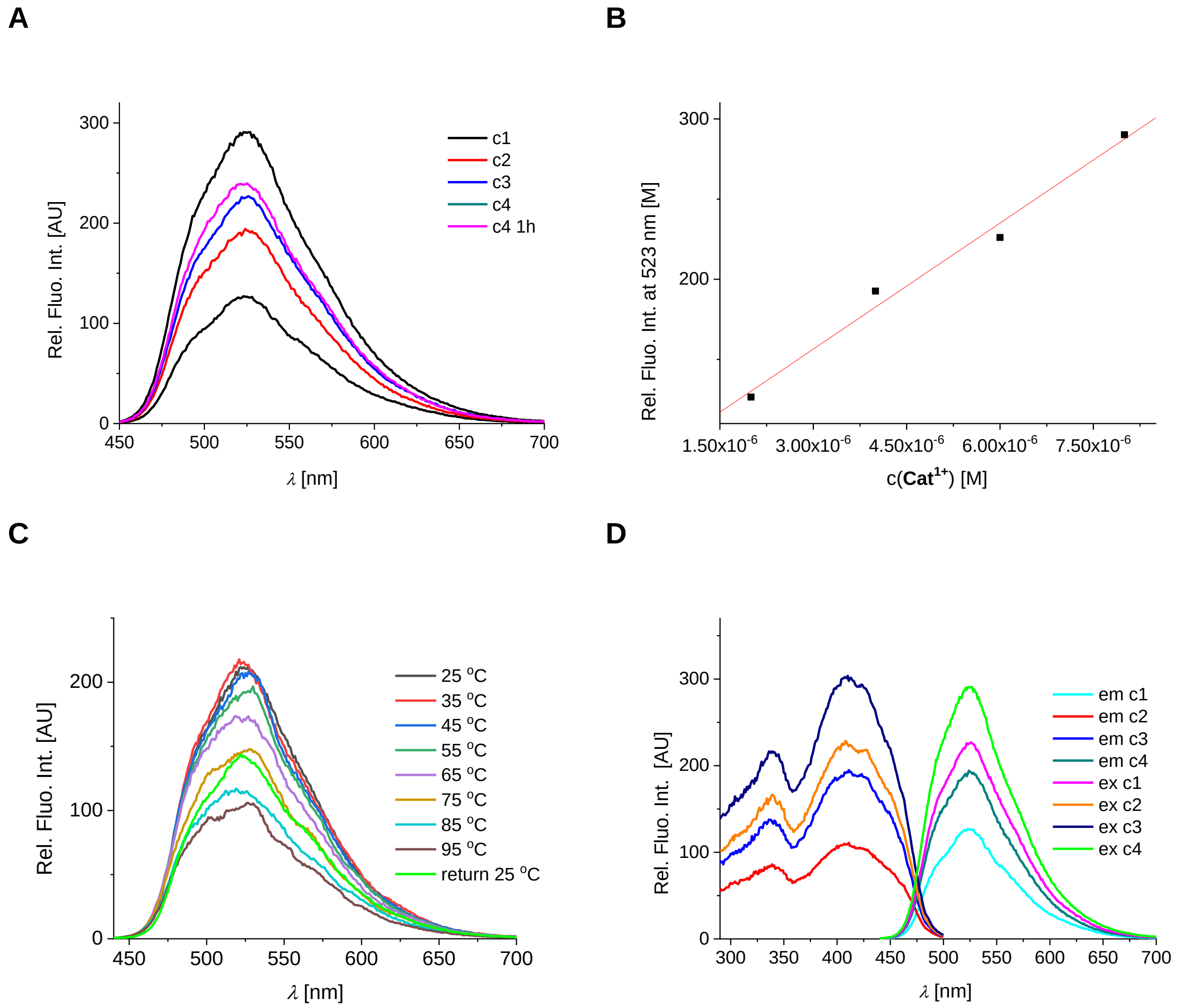

Figure S105: **A)** Emission spectra of **Cat^{1+}** (λ_{exc} = 414 nm; c = 2 × 10^{-6} – 8 × 10^{-6} M). **B)** Linear dependence (—) of the fluorescence intensity (λ_{exc} = 414 nm, λ_{em} = 523 nm) (■) on the concentration of **Cat^{1+}**. **C)** Influence of temperature increase (T = 25 °C – 95 °C) on fluorescence spectra of **Cat^{1+}** (c = 8 × 10^{-6} M; sodium cacodylate buffer, pH = 7, *I* = 0.05 M). **D)** Comparison of emission and excitation spectra (λ_{em} = 523 nm).

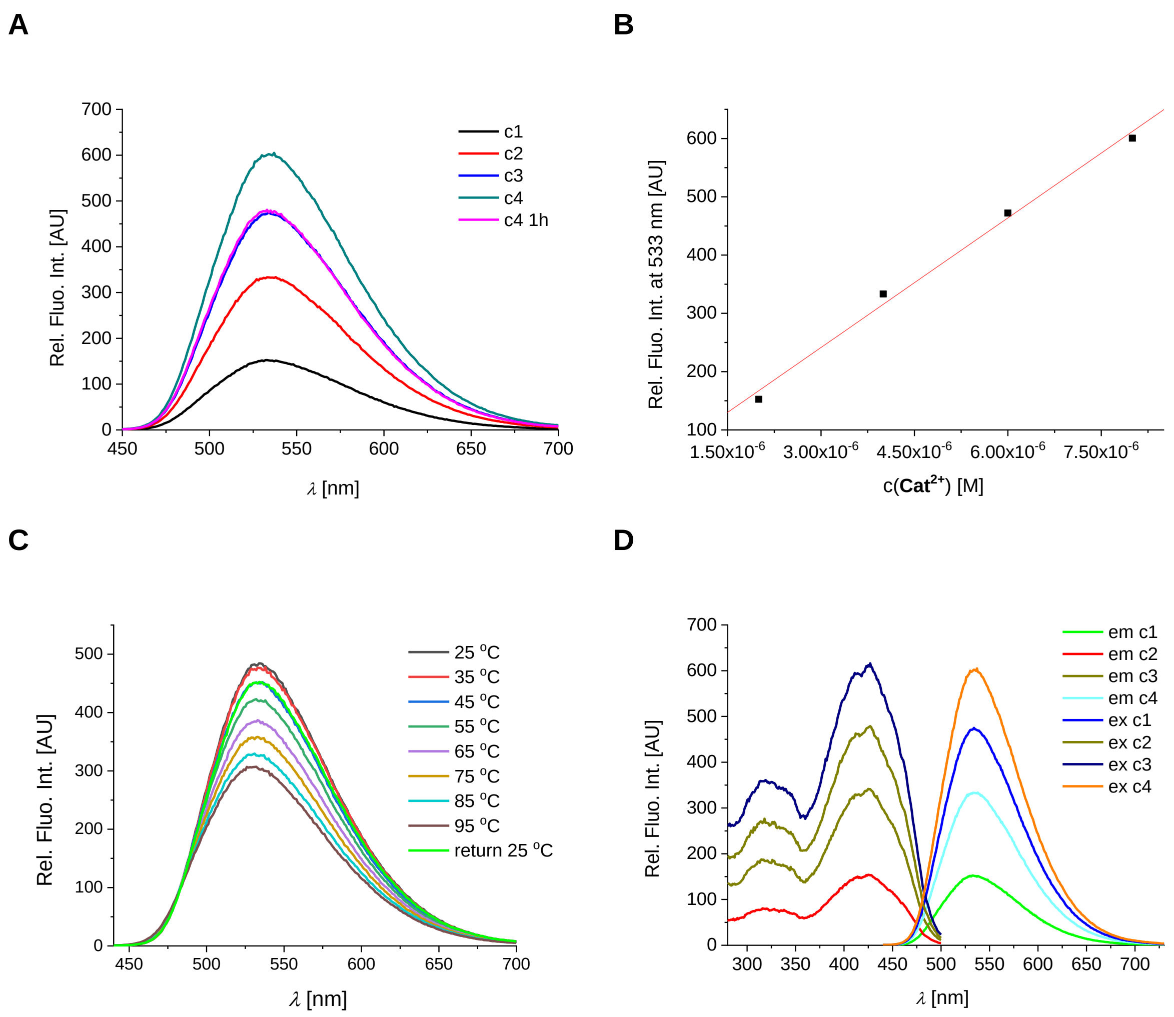

Figure S106: **A)** Emission spectra of **Cat^{2+}** (λ_{exc} = 422 nm; c = 2 × 10^{-6} – 8 × 10^{-6} M). **B)** Linear dependence (—) of the fluorescence intensity (λ_{exc} = 422 nm, λ_{em} = 533 nm) (■) on the concentration of **Cat^{2+}**. **C)** Influence of temperature increase (T = 25 °C – 95 °C) on fluorescence spectra of **Cat^{2+}** (c = 8 × 10^{-6} M; sodium cacodylate buffer, pH = 7, *I* = 0.05 M). **D)** Comparison of emission and excitation spectra (λ_{em} = 533 nm).

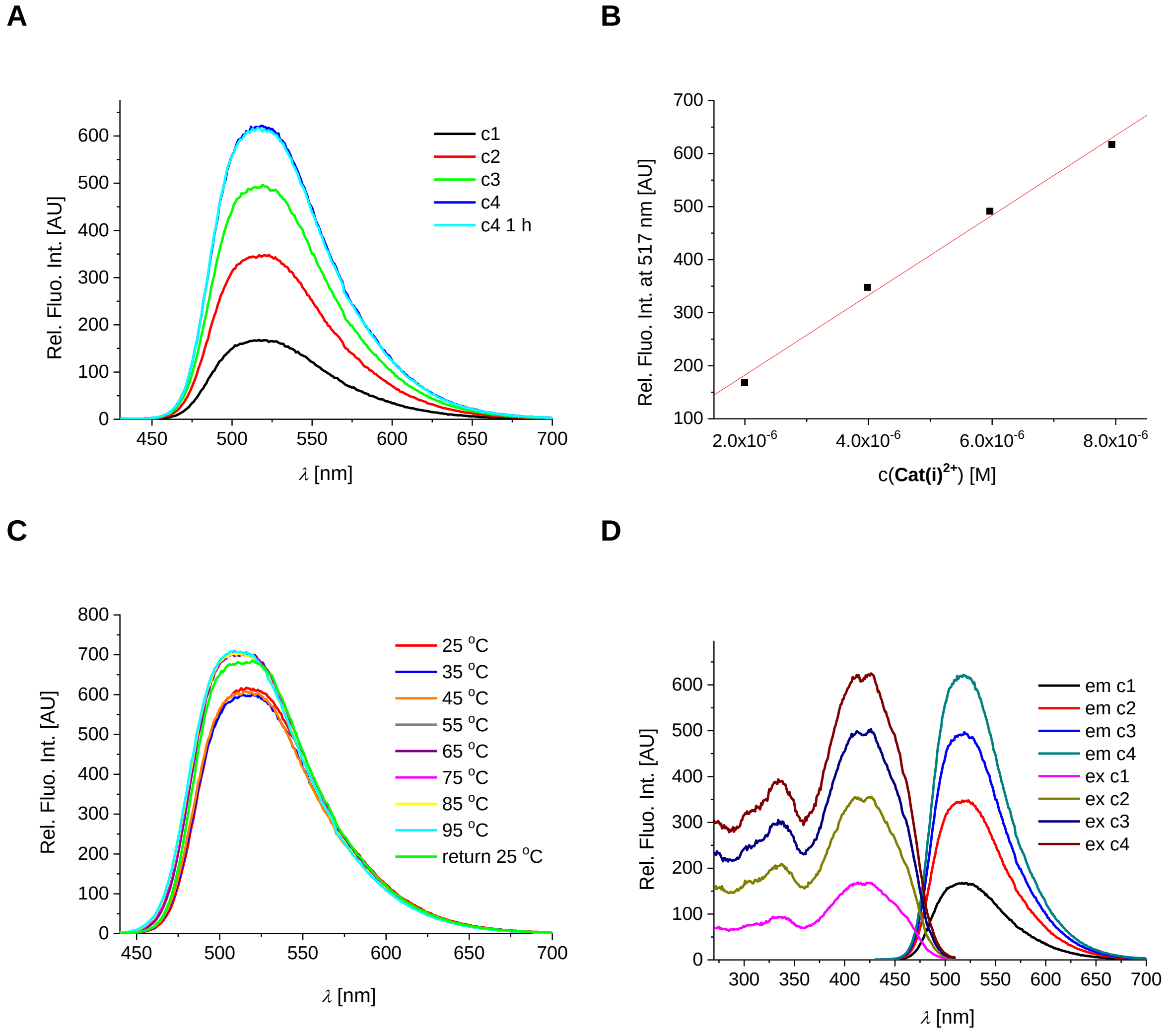

Figure S107: **A)** Emission spectra of **Cat(i)$^{2+}$** (λ_{exc} = 417 nm; c = 2 × 10^{-6} – 8 × 10^{-6} M). **B)** Linear dependence (—) of the fluorescence intensity (λ_{exc} = 417 nm, λ_{em} = 517 nm) (■) on the concentration of **Cat(i)$^{2+}$**. **C)** Influence of temperature increase (T = 25 °C – 95 °C) on fluorescence spectra of **Cat(i)$^{2+}$** (c = 8 × 10^{-6} M; sodium cacodylate buffer, pH = 7, *I* = 0.05 M). **D)** Comparison of emission and excitation spectra (λ_{em} = 517 nm).

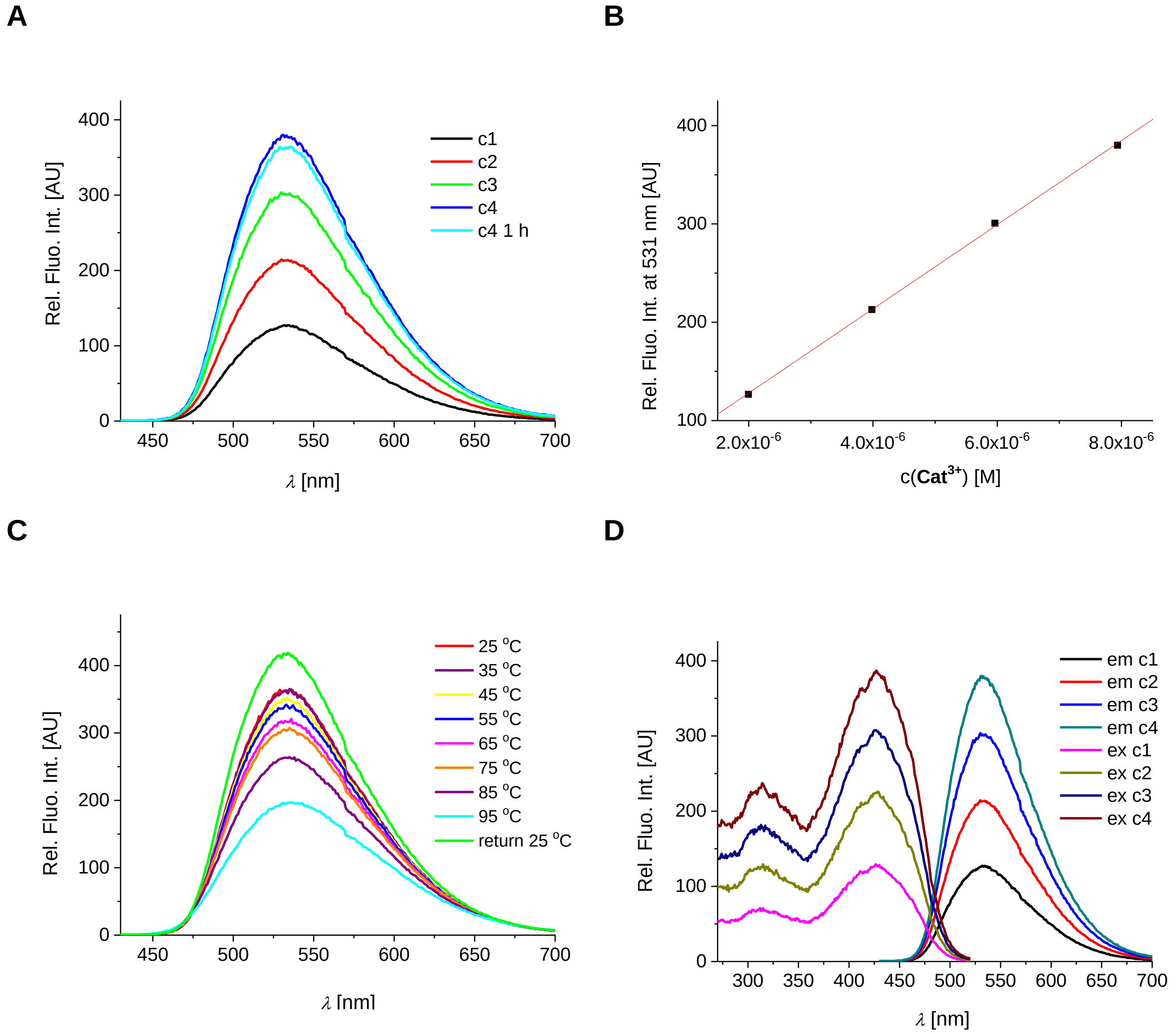

Figure S108: **A)** Emission spectra of **Cat^{3+}** (λ_{exc} = 421 nm; c = 2 × 10^{-6} – 8 × 10^{-6} M). **B)** Linear dependence (—) of the fluorescence intensity (λ_{exc} = 421 nm, λ_{em} = 531 nm) (■) on the concentration of **Cat^{3+}**. **C)** Influence of temperature increase (T = 25 °C – 95 °C) on fluorescence spectra of **Cat^{3+}** (c = 8 × 10^{-6} M; sodium cacodylate buffer, pH = 7, *I* = 0.05 M). **D)** Comparison of emission and excitation spectra (λ_{em} = 531 nm).

Interaction of **Cat1+-Cat3+** with ctDNA, and pApU at pH 7

Thermal Melting Experiments

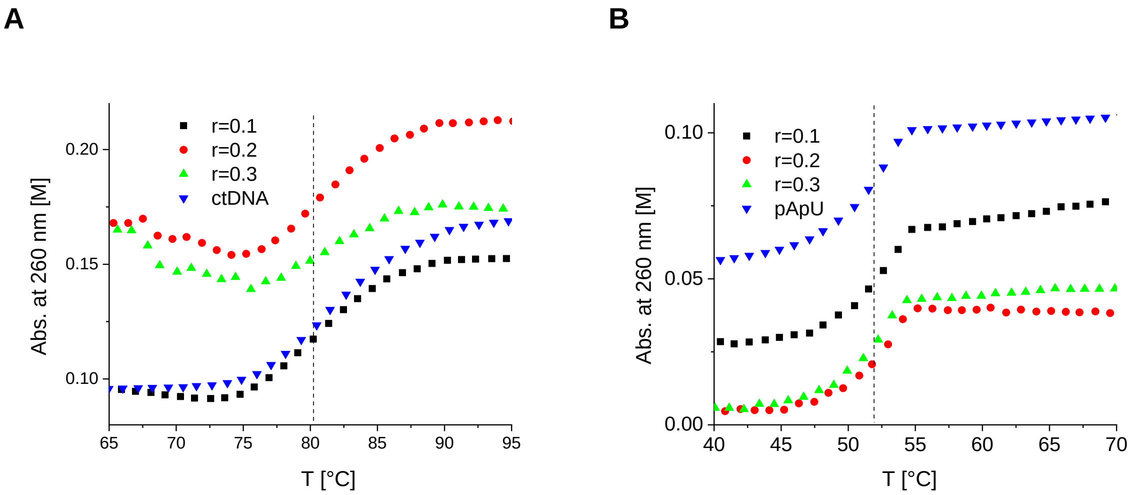

Figure S109: **A)** Thermal denaturation curves of ctDNA (c(ctDNA) = 2.26×10^{-5} M, $r_{[\mathbf{Cat^{1+}}]/[\mathbf{ctDNA}]}$ = 0.1 to 0.3) at pH 7 (sodium cacodylate buffer, I = 0.05 M) upon addition of **Cat^{1+}**. Error in ΔT_m values: ±0.5 °C. **B)** Thermal denaturation curves of pApU (c(pApU) = 2.49×10^{-5} M, $r_{[\mathbf{Cat^{1+}}]/[\mathbf{pApU}]}$ = 0.1 to 0.3) at pH 7 (sodium cacodylate buffer, I = 0.05 M) upon addition of **Cat^{1+}**. Error in ΔT_m values: ±0.5 °C.

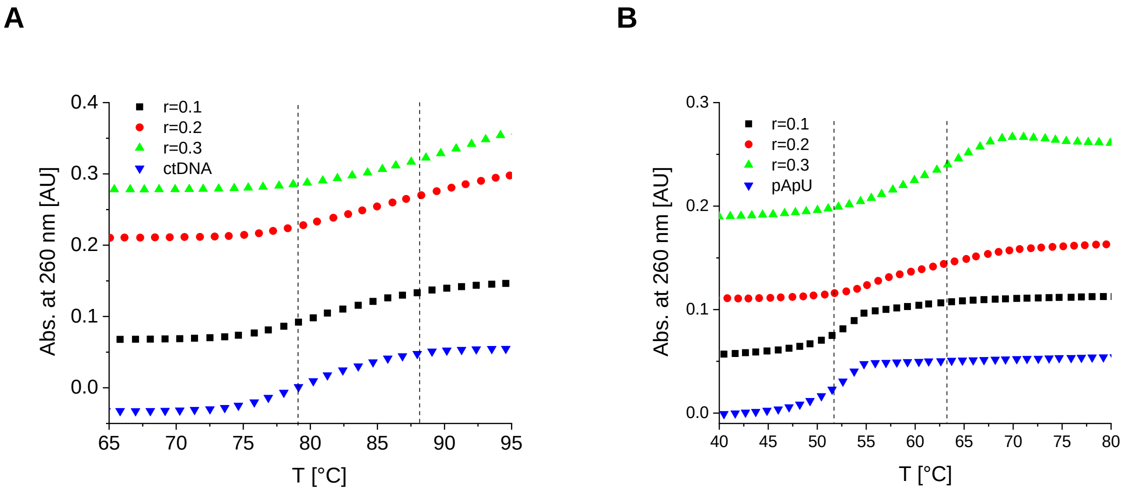

Figure S110: **A)** Thermal denaturation curves of ctDNA (c(ctDNA) = 2.26×10^{-5} M, $r_{[\mathbf{Cat^{2+}}]/[\mathbf{ctDNA}]}$ = 0.1 to 0.3) at pH 7 (sodium cacodylate buffer, I = 0.05 M) upon addition of **Cat^{2+}**. Error in ΔT_m values: ±0.5 °C. **B)** Thermal denaturation curves of pApU (c(pApU) = 2.49×10^{-5} M, $r_{[\mathbf{Cat^{2+}}]/[\mathbf{pApU}]}$ = 0.1 to 0.3) at pH 7 (sodium cacodylate buffer, I = 0.05 M) upon addition of **Cat^{2+}**. Error in ΔT_m values: ±0.5 °C.

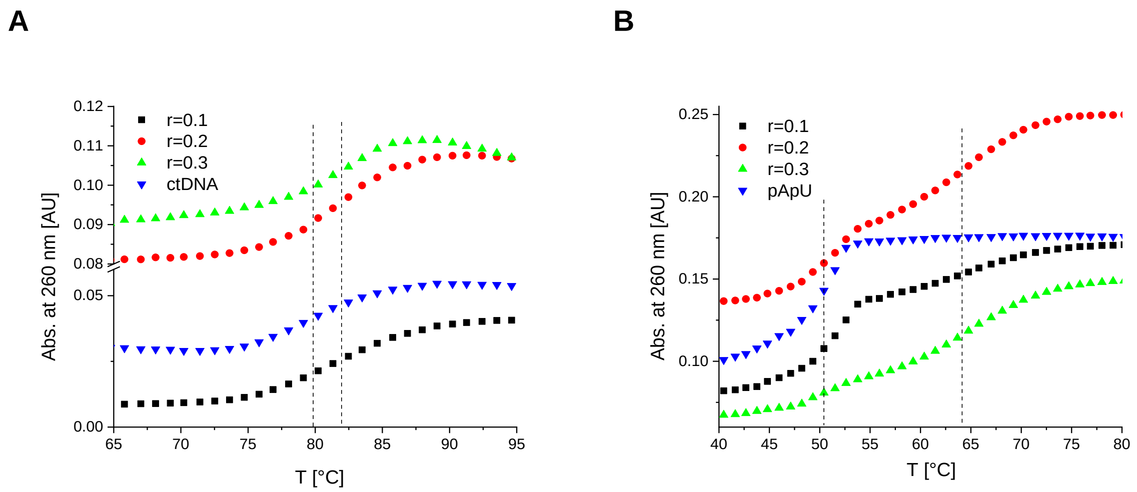

Figure S111: **A)** Thermal denaturation curves of ctDNA (c(ctDNA) = 2.26×10^{-5} M, $r_{[\mathbf{Cat(i)}^{2+}]/[\mathbf{ctDNA}]}$ = 0.1 to 0.3) at pH 7 (sodium cacodylate buffer, I = 0.05 M) upon addition of **Cat(i)$^{2+}$**. Error in ΔT_m values: ±0.5 °C. **B)** Thermal denaturation curves of pApU (c(pApU) = 2.49×10^{-5} M, $r_{[\mathbf{Cat(i)}^{2+}]/[\mathbf{pApU}]}$ = 0.1 to 0.3) at pH 7 (sodium cacodylate buffer, I = 0.05 M) upon addition of **Cat(i)$^{2+}$**. Error in ΔT_m values: ±0.5 °C.

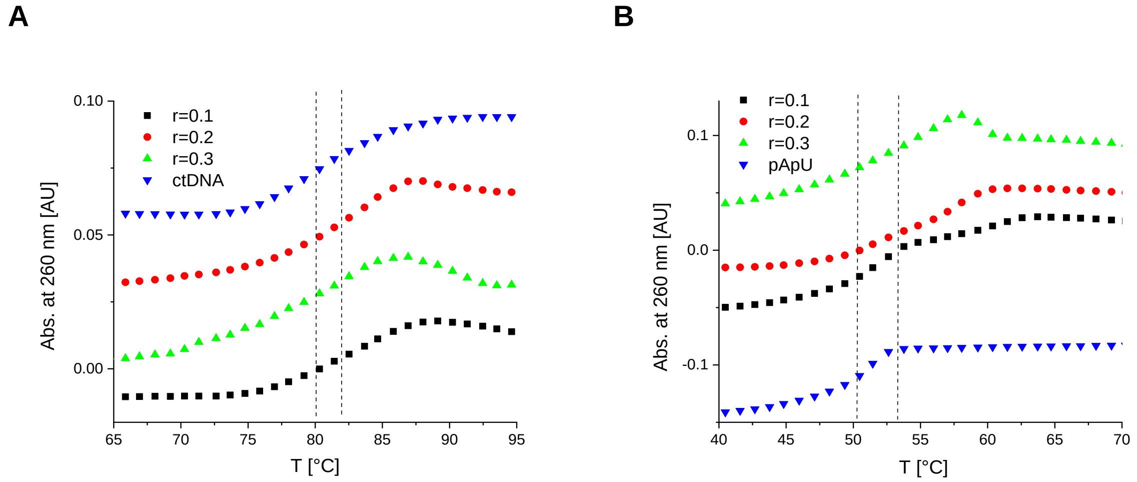

Figure S112: **A)** Thermal denaturation curves of ctDNA (c(ctDNA) = 2.26×10^{-5} M, $r_{[\mathbf{Cat}^{3+}]/[\mathbf{ctDNA}]}$ = 0.1 to 0.3) at pH 7 (sodium cacodylate buffer, *I* = 0.05 M) upon addition of **Cat^{3+}**. Error in ΔT_m values: ±0.5 °C. **B)** Thermal denaturation curves of pApU (c(pApU) = 2.49×10^{-5} M, $r_{[\mathbf{Cat}^{3+}]/[\mathbf{pApU}]}$ = 0.1 to 0.3) at pH 7 (sodium cacodylate buffer, *I* = 0.05 M) upon addition of **Cat^{3+}**. Error in ΔT_m values: ±0.5 °C.

Fluorimetric Titrations

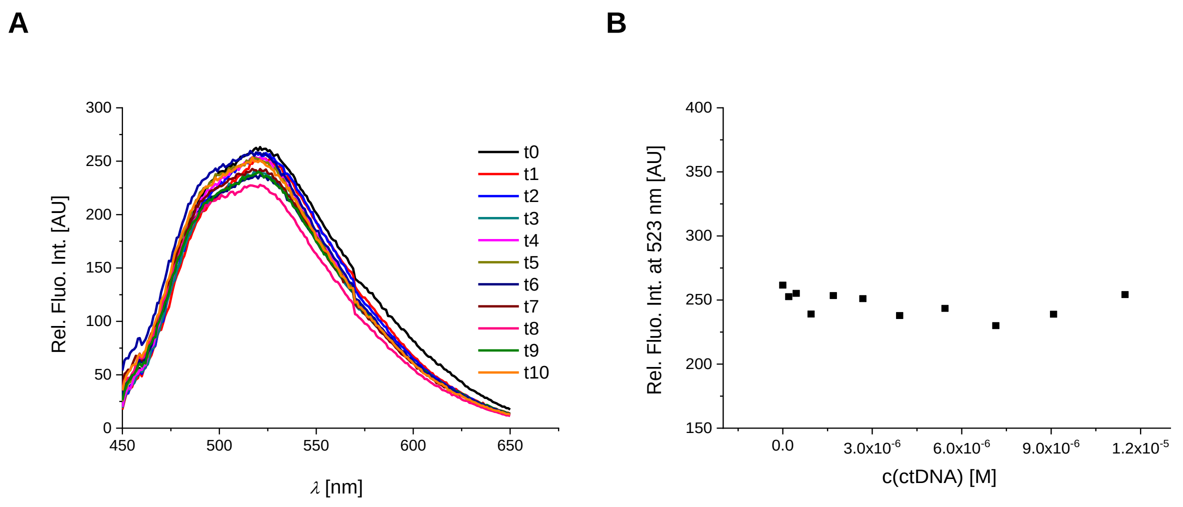

Figure S113: **A)** Fluorimetric titration of **Cat^{1+}** (c = 1 × 10^{-7} M; λ_{exc} = 414 nm; pH 7, sodium cacodylate buffer, *I* = 0.05 M) with ctDNA. **B)** Dependence of fluorescence of **Cat^{1+}** at λ_{max} = 523 nm on c(ctDNA).

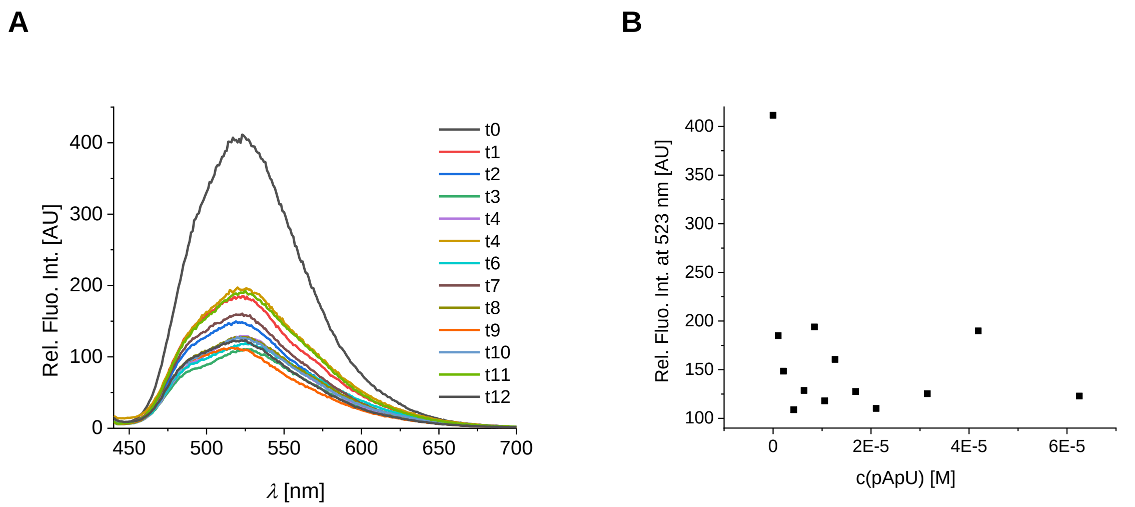

Figure S114: **A)** Fluorimetric titration of **Cat^{1+}** (c = 5 × 10^{-7} M; λ_{exc} = 414 nm; pH 7, sodium cacodylate buffer, *I* = 0.05 M) with pApU. **B)** Dependence of fluorescence of **Cat^{1+}** at λ_{max} = 523 nm on c(pApU). Note that during the measurement, formation of colloids was observed.

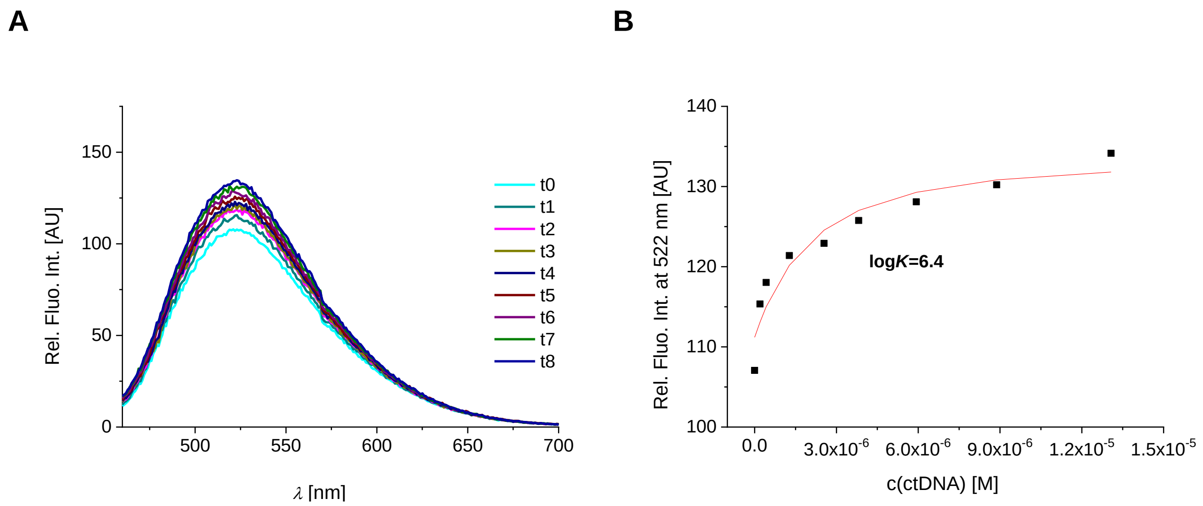

Figure S115: **A)** Fluorimetric titration of **Cat^{2+}** (c = 1 × 10^{-7} M; λ_{exc} = 422 nm; pH 7, sodium cacodylate buffer, *I* = 0.05 M) with ctDNA. **B)** Dependence of fluorescence of **Cat^{2+}** at λ_{max} = 522 nm on c(ctDNA).

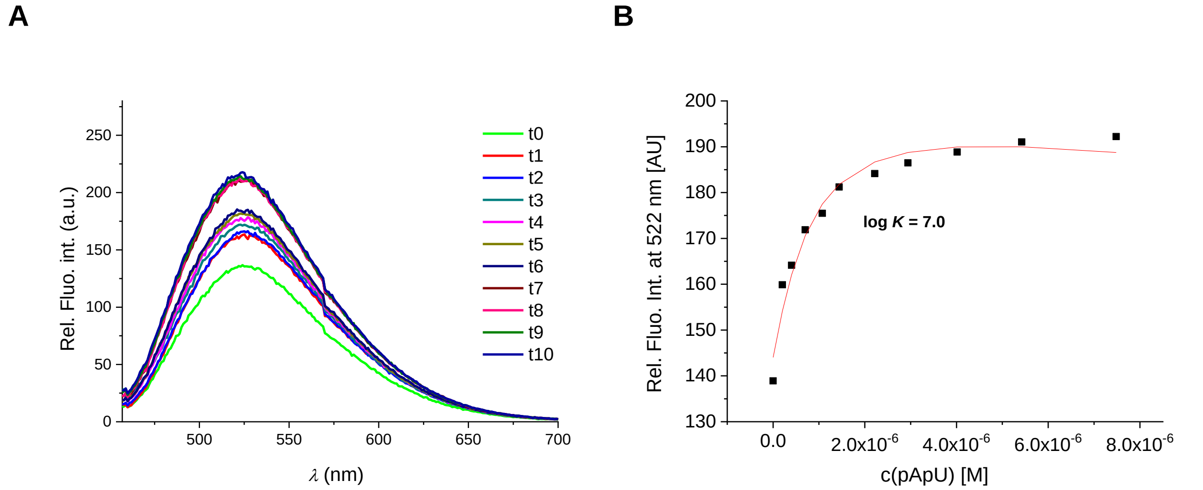

Figure S116: **A)** Fluorimetric titration of **Cat^{2+}** (c = 1 × 10^{-7} M; λ_{exc}= 422 nm; pH 7, sodium cacodylate buffer, *I* = 0.05 M) with pApU. **B)** Dependence of fluorescence of **Cat^{2+}** at λ_{max} = 522 nm on c(pApU).

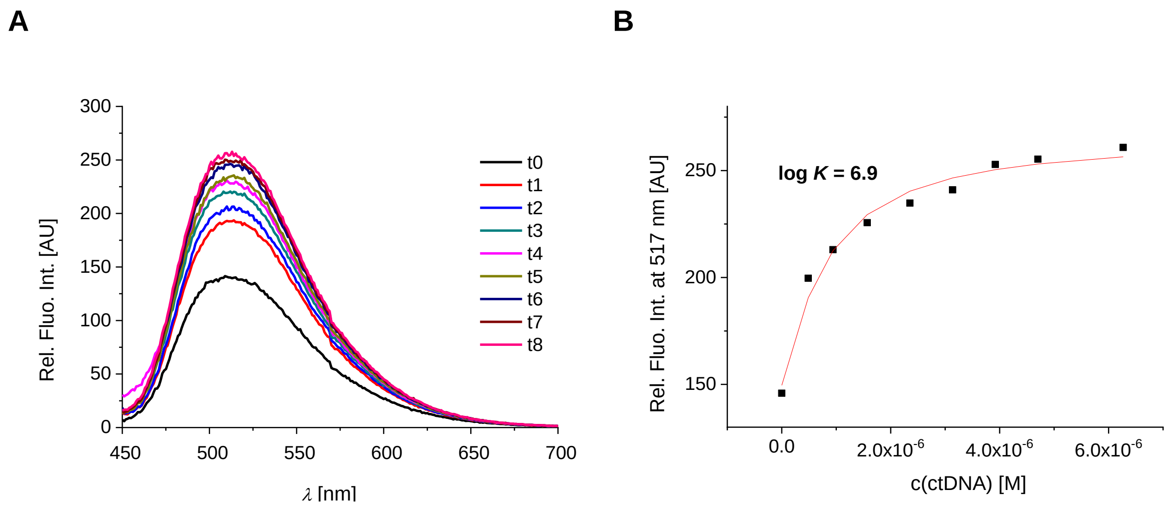

Figure S117: **A)** Fluorimetric titration of **Cat(i)$^{2+}$** (c = 1 × 10^{-7} M; λ_{exc} = 417 nm; pH 7, sodium cacodylate buffer, *I* = 0.05 M) with ctDNA. **B)** Dependence of fluorescence of **Cat(i)$^{2+}$** at λ_{max} = 517 nm on c(ctDNA).

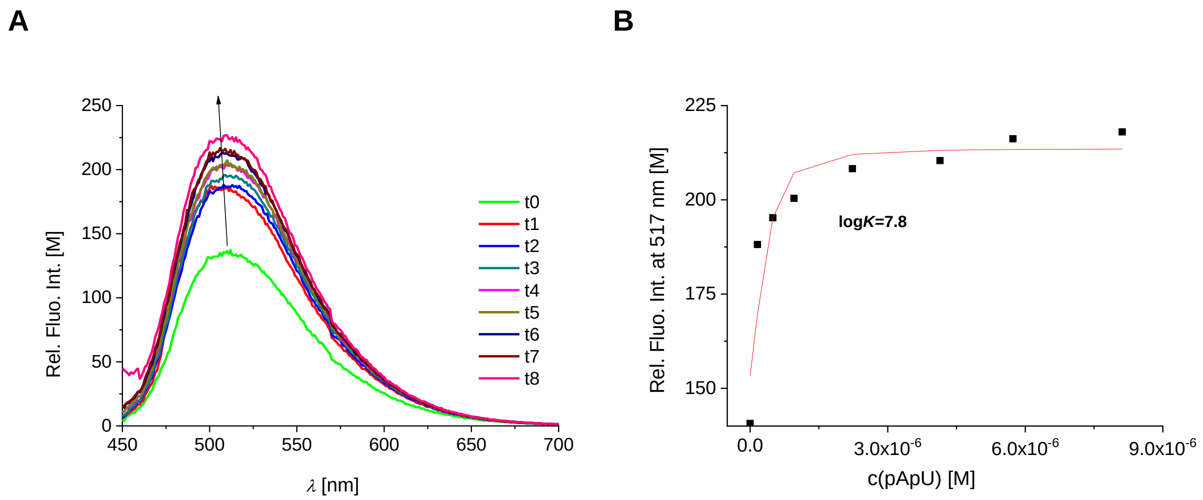

Figure S118: **A)** Fluorimetric titration of **Cat(i)$^{2+}$** (c = 1 × 10^{-7} M; λ_{exc} = 417 nm; pH 7, sodium cacodylate buffer, *I* = 0.05 M) with pApU. **B)** Dependence of fluorescence of **Cat(i)$^{2+}$** at λ_{max} = 517 nm on c(pApU).

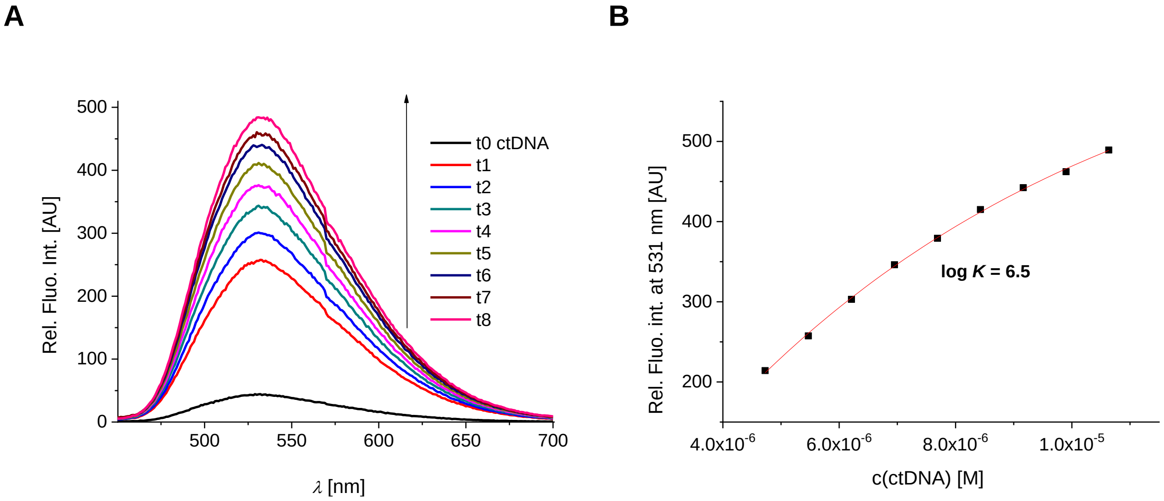

Figure S119: **A)** Fluorimetric titration of ctDNA (c = 1 × 10^{-7} M ; λ_{exc} = 421 nm; pH 7, sodium cacodylate buffer, *I* = 0.05 M) with **Cat^{3+}**. **B)** Dependence of fluorescence of **Cat^{3+}** at λ_{max} = 531 nm on c(ctDNA).

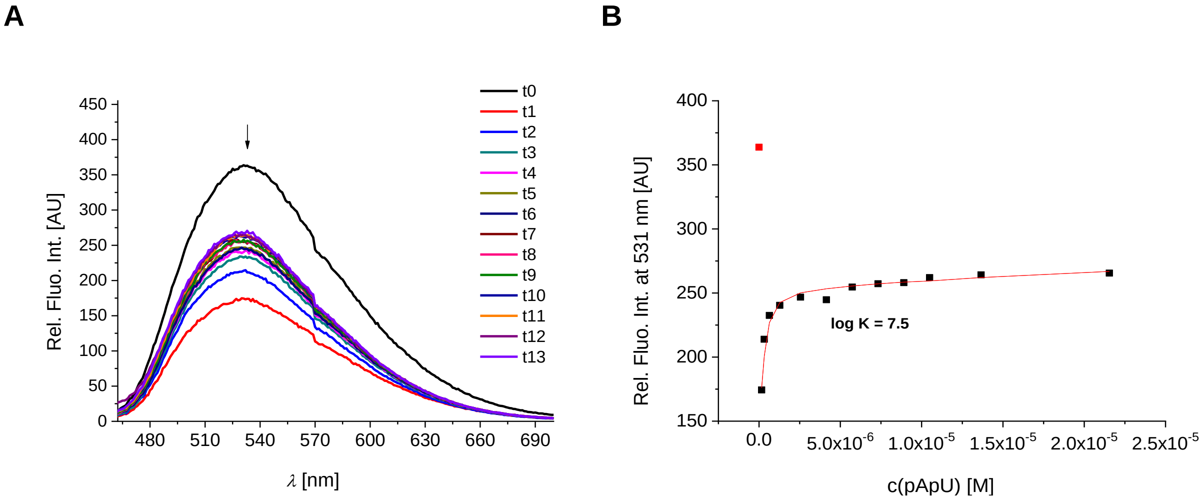

Figure S120: **A)** Fluorimetric titration of **Cat^{3+}** (c = 1 × 10^{-7} M; λ_{exc} = 421 nm; pH 7, sodium cacodylate buffer, *I* = 0.05 M) with pApU. **B)** Dependence of fluorescence of **Cat^{3+}** at λ_{max} = 531 nm on c(pApU). The data point marked in red was not included in calculations.

Circular Dichroism Experiments

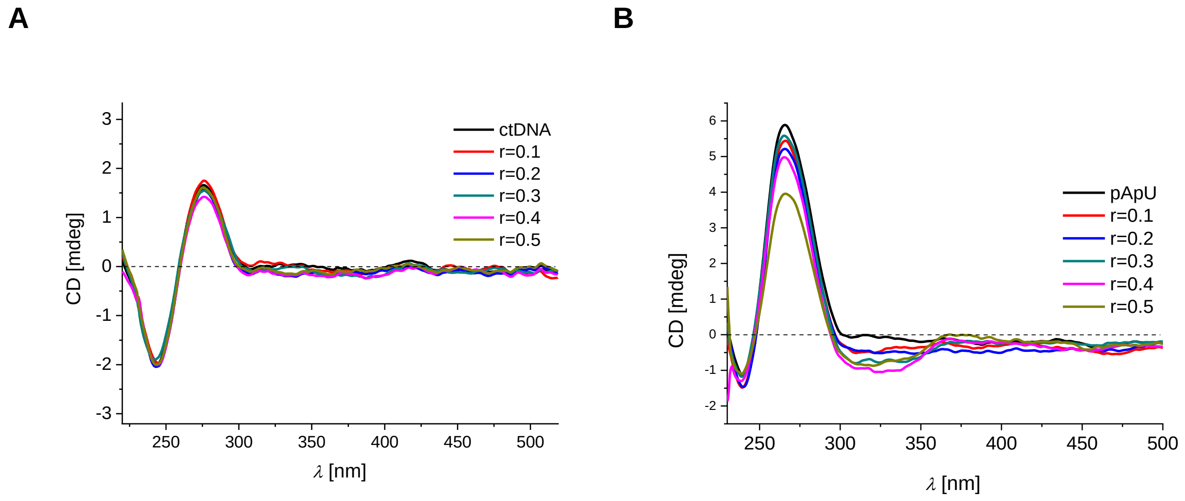

Figure S121: **A)** CD titration of ctDNA (c(ctDNA) = 2 × 10^{-5} M; pH 7, sodium cacodylate buffer, *I* = 0.05 M) with **Cat^{1+}** at molar ratios $r_{[compound]/[polynucleotide]}$ = 0.1 – 0.5. **B)** CD titration of pApU (c(pApU) = 2.5 × 10^{-5} M; pH 7, sodium cacodylate buffer, *I* = 0.05 M) with **Cat^{1+}** at molar ratios $r_{[compound]/[polynucleotide]}$ = 0.1 – 0.5.

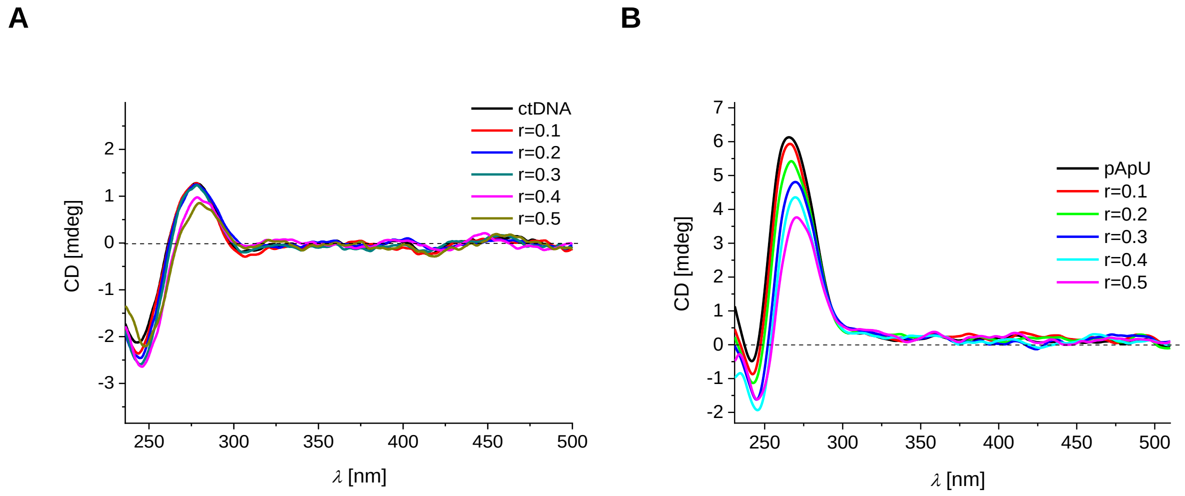

Figure S122: **A)** CD titration of ctDNA (c(ctDNA) = 2 × 10^{-5} M; pH 7, sodium cacodylate buffer, *I* = 0.05 M) with **Cat^{2+}** at molar ratios $r_{[compound]/[polynucleotide]}$ = 0.1 – 0.5. **B)** CD titration of pApU (c(pApU) = 2.5 × 10^{-5} M; pH 7, sodium cacodylate buffer, *I* = 0.05 M) with **Cat^{2+}** at molar ratios $r_{[compound]/[polynucleotide]}$ = 0.1 – 0.5.

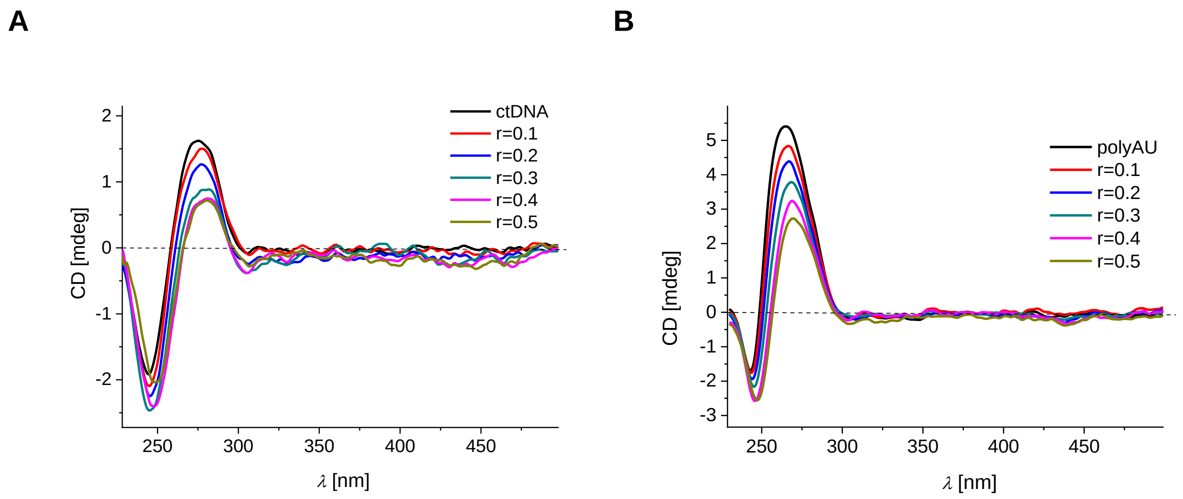

Figure S123: **A)** CD titration of ctDNA (c(ctDNA) = 2 × 10^{-5} M; pH 7, sodium cacodylate buffer, I = 0.05 M) with **Cat(i)$^{2+}$** at molar ratios $r_{[compound]/[polynucleotide]}$ = 0.1 – 0.5. **B)** CD titration of pApU (c(pApU) = 2.5 × 10^{-5} M; pH 7, sodium cacodylate buffer, I = 0.05 M) with **Cat(i)$^{2+}$** at molar ratios $r_{[compound]/[polynucleotide]}$ = 0.1 – 0.5.

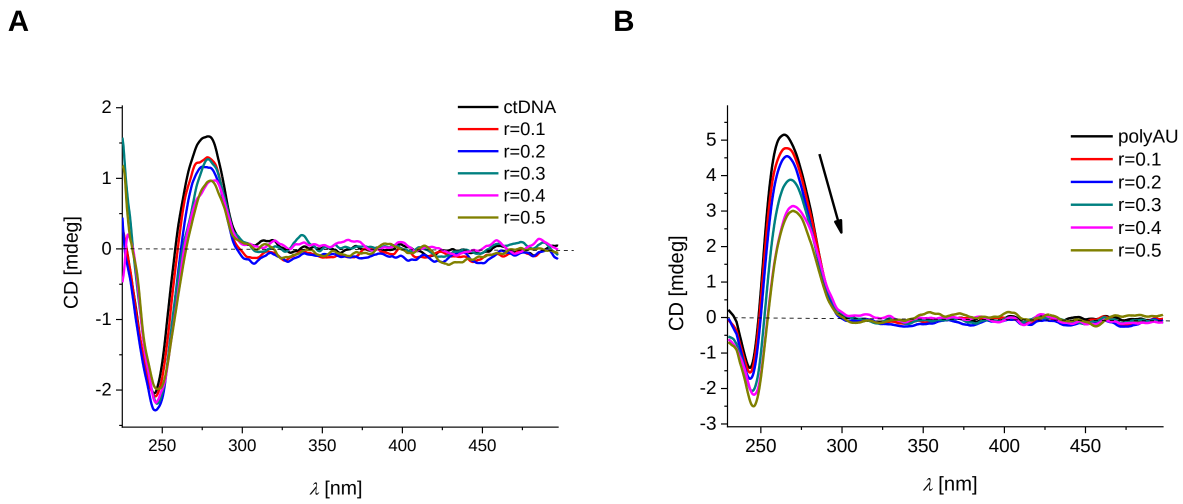

Figure S124: **A)** CD titration of ctDNA (c(ctDNA) = 2 × 10^{-5} M; pH 7, sodium cacodylate buffer, I = 0.05 M) with **Cat^{3+}** at molar ratios $r_{[compound]/[polynucleotide]}$ = 0.1 – 0.5. **B)** CD titration of pApU (c(pApU) = 2.5 × 10^{-5} M; pH 7, sodium cacodylate buffer, I = 0.05 M) with **Cat^{3+}** at molar ratios $r_{[compound]/[polynucleotide]}$ = 0.1 – 0.5.

Interaction of **Cat1+-Cat4+** with ctDNA, and DNApore at pH 8

Thermal Melting Experiments

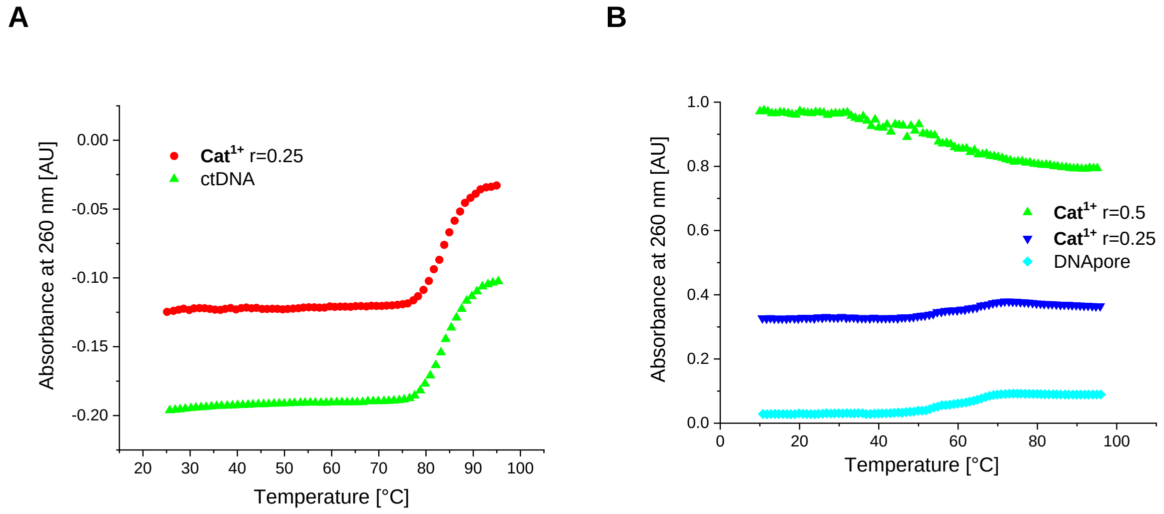

Figure S125: **A)** Thermal denaturation curves of ctDNA (c(ctDNA) = 2.26×10^{-5} M, $r_{[\mathbf{Cat^{1+}}]/[\mathbf{ctDNA}]}$ = 0.25) at pH 8 (15 mM Tris-HCl, 300 mM KCl) upon addition of **Cat1+**. Error in ΔT_m values: ±0.5 °C. **B)** Thermal denaturation curves of DNApore (c(DNApore) = 2×10^{-5} M, $r_{[\mathbf{Cat^{1+}}]/[\mathbf{DNApore}]}$ = 0.25 to 0.5) at pH 8 (15 mM Tris-HCl, 300 mM KCl) upon addition of **Cat1+**. Error in ΔT_m values: ±0.5 °C.

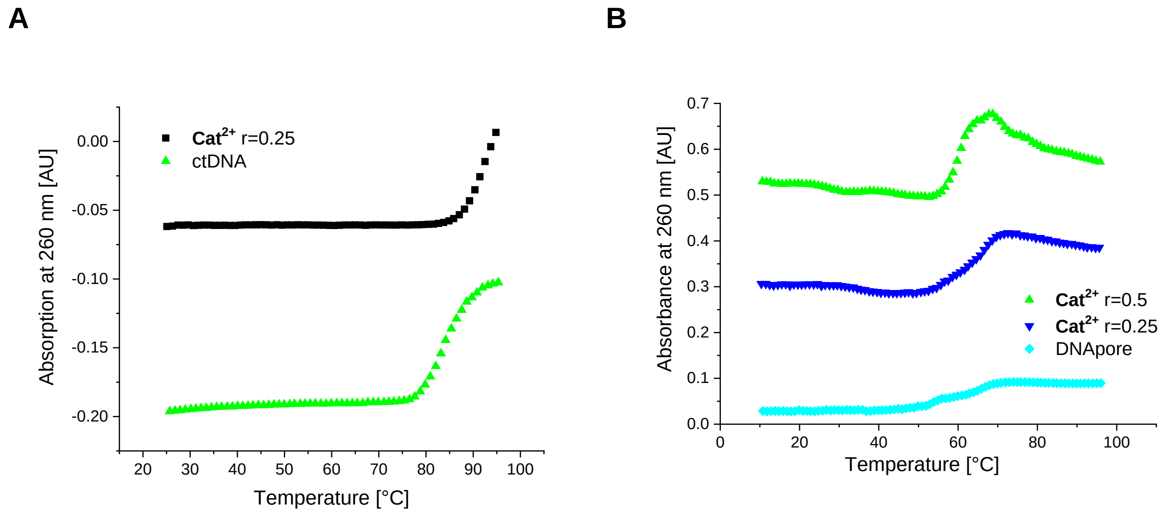

Figure S126: **A)** Thermal denaturation curves of ctDNA (c(ctDNA) = 2.26×10^{-5} M, $r_{[\mathbf{Cat^{2+}}]/[\mathbf{ctDNA}]}$ = 0.25) at pH 8 (15 mM Tris-HCl, 300 mM KCl) upon addition of **Cat2+**. Error in ΔT_m values: ±0.5 °C. **B)** Thermal denaturation curves of DNApore (c(DNApore) = 2.49×10^{-5} M, $r_{[\mathbf{Cat^{2+}}]/[\mathbf{DNApore}]}$ = 0.25 to 0.5) at pH 8 (15 mM Tris-HCl, 300 mM KCl) upon addition of **Cat2+**. Error in ΔT_m values: ±0.5 °C.

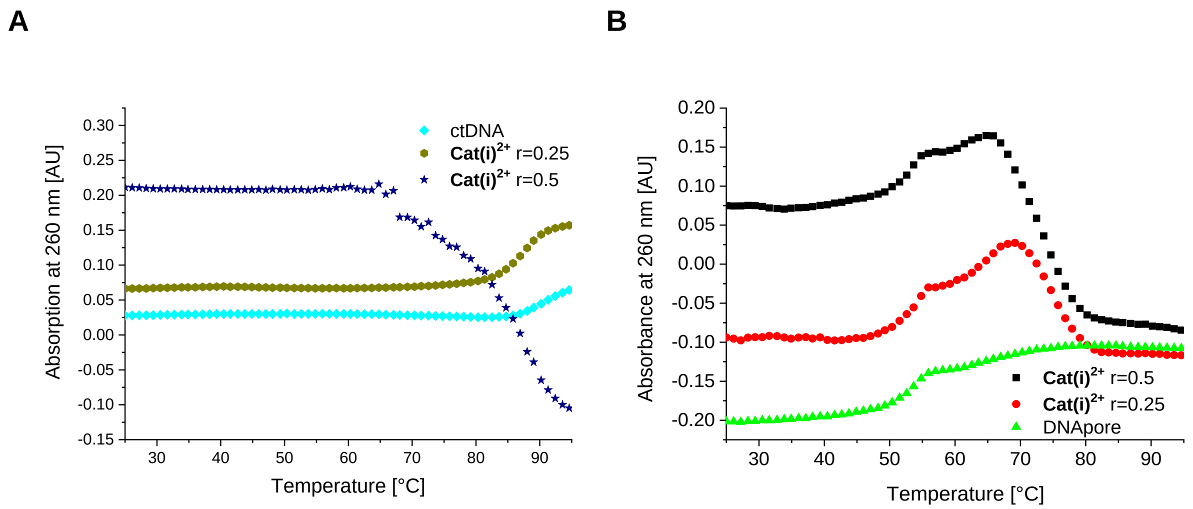

Figure S127: **A)** Thermal denaturation curves of ctDNA ($c(\text{ctDNA}) = 2.26 \times 10^{-5}$ M, $r_{[\mathbf{Cat(i)}^{2+}]/[\mathbf{ctDNA}]} = 0.25$ to 0.5) at pH 8 (15 mM Tris-HCl, 300 mM KCl) upon addition of **Cat(i)**$^{2+}$. Error in ΔT_m values: ±0.5 °C. **B)** Thermal denaturation curves of DNApore ($c(\text{DNApore}) = 1.05 \times 10^{-6}$ M, $r_{[\mathbf{Cat(i)}^{2+}]/[\mathbf{DNApore}]} = 0.25$ to 0.5) at pH 8 (15 mM Tris-HCl, 300 mM KCl) upon addition of **Cat(i)**$^{2+}$. Error in ΔT_m values: ±0.5 °C.

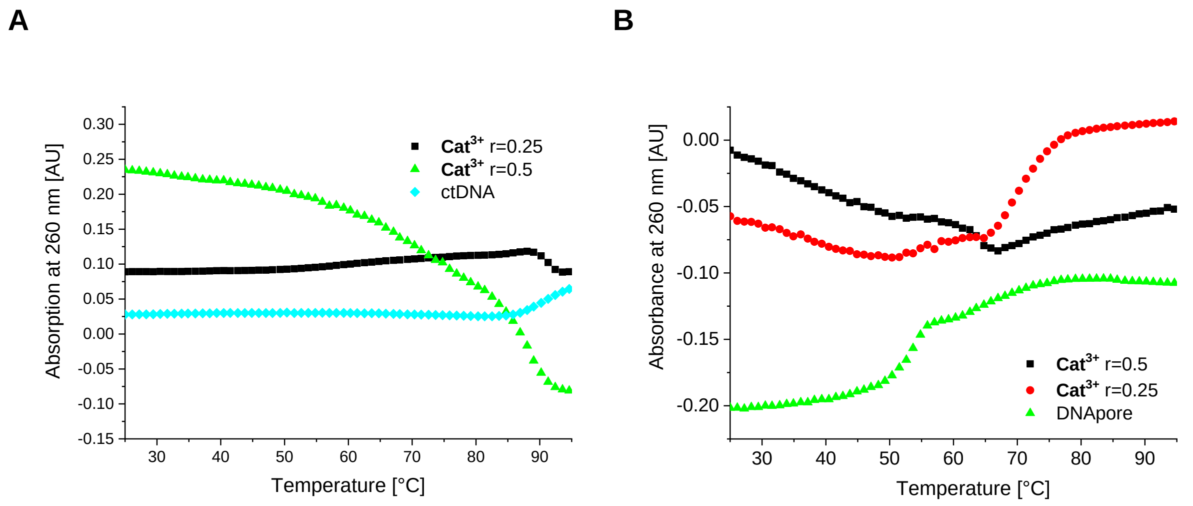

Figure S128: **A)** Thermal denaturation curves of ctDNA ($c(\text{ctDNA}) = 2.26 \times 10^{-5}$ M, $r_{[\mathbf{Cat}^{3+}]/[\mathbf{ctDNA}]} = 0.25$ to 0.5) at pH 8 (15 mM Tris-HCl, 300 mM KCl) upon addition of **Cat**$^{3+}$. Error in ΔT_m values: ±0.5 °C. **B)** Thermal denaturation curves of DNApore ($c(\text{DNApore}) = 1.05 \times 10^{-6}$ M, $r_{[\mathbf{Cat}^{3+}]/[\mathbf{DNApore}]} = 0.25$ to 0.5) at pH 8 (15 mM Tris-HCl, 300 mM KCl) upon addition of **Cat**$^{3+}$. Error in ΔT_m values: ±0.5 °C.

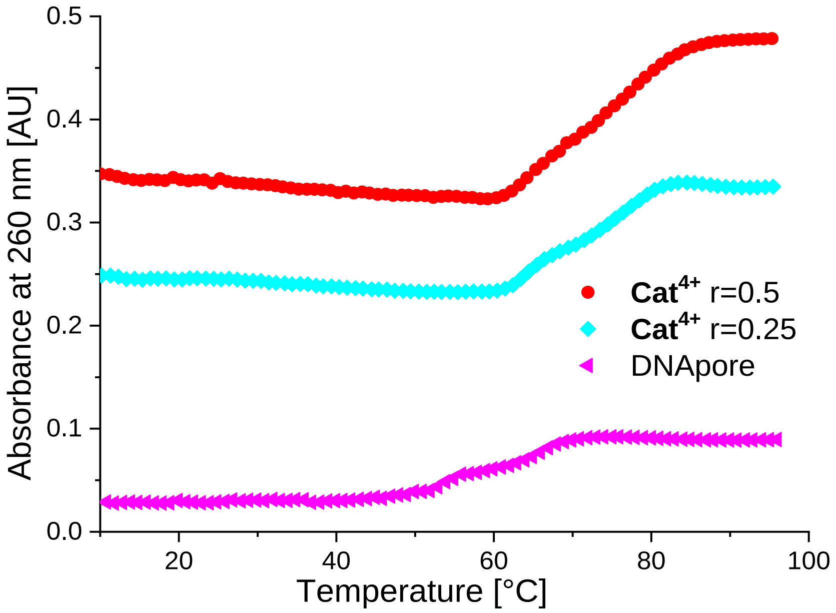

Figure S129: Thermal denaturation curves of DNApore (c(DNApore) = 2×10^{-5} M, $r_{[\mathbf{Cat^{4+}}]/[\mathbf{DNApore}]}$ = 0.25 to 0.5) at pH 8 (15 mM Tris-HCl, 300 mM KCl) upon addition of **Cat^{4+}**. Error in ΔT_m values: ±0.5 °C.

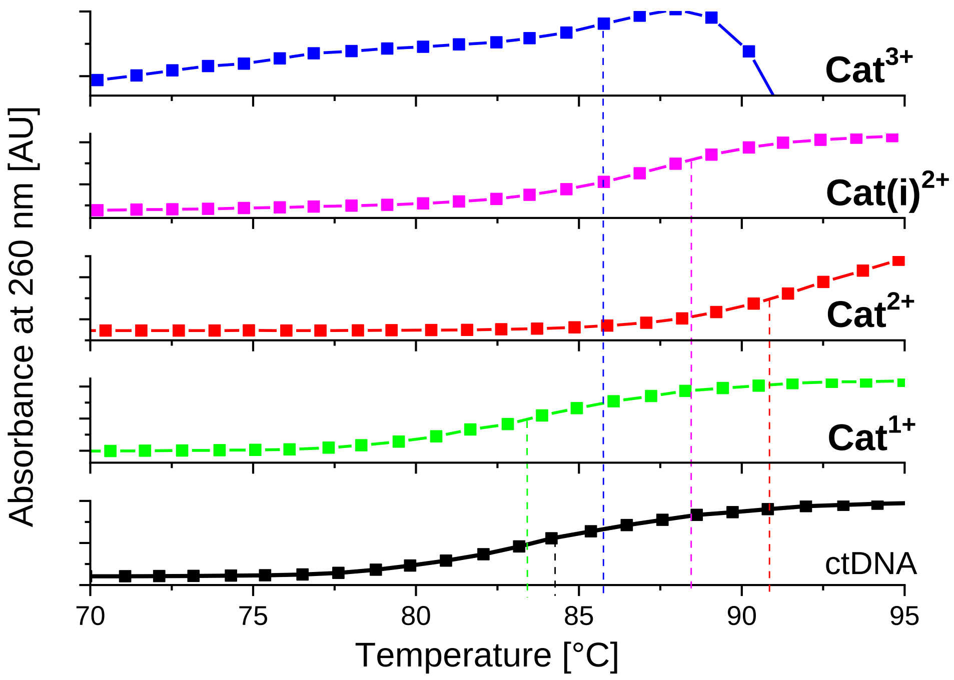

Figure S130: Thermal denaturation curves of ctDNA (c(ctDNA) = 2.26×10^{-5} M, $r_{[\textit{bis}\text{-triarylboranes}]/[\textbf{ctDNA}]}$ = 0.2) at pH 8 (15 mM Tris-HCl, 300 mM KCl) upon addition of *bis*-triarylboranes. Error in ΔT_m values: ±0.5 °C.

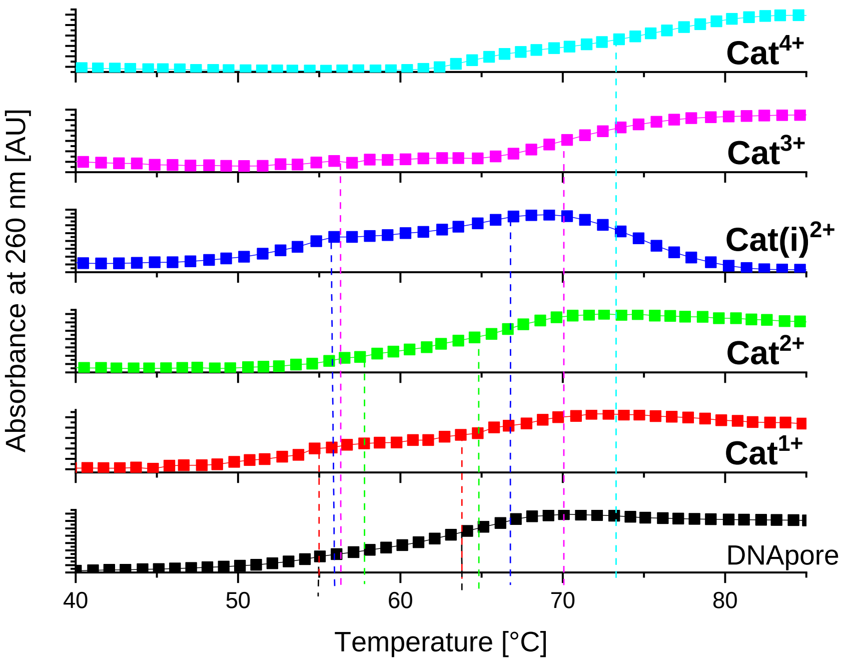

Figure S131: Melting curves of DNApore (c(DNApore) = 2 x 10^{-5} M; ratio $r_{[\text{compound}]/[\text{DNApore}]}$ = 0.25) upon addition of *bis*-triarylboranes at pH 8 (15 mM Tris-HCl, 300 mM KCl).

Fluorimetric Titrations

Fluorimetric titration of **Cat^{1+}** with ctDNA at pH 8 (15 mM Tris-HCl, 300 mM KCl) was not performed as the titration at pH 7 showed now binding between **Cat^{1+}** and ctDNA.

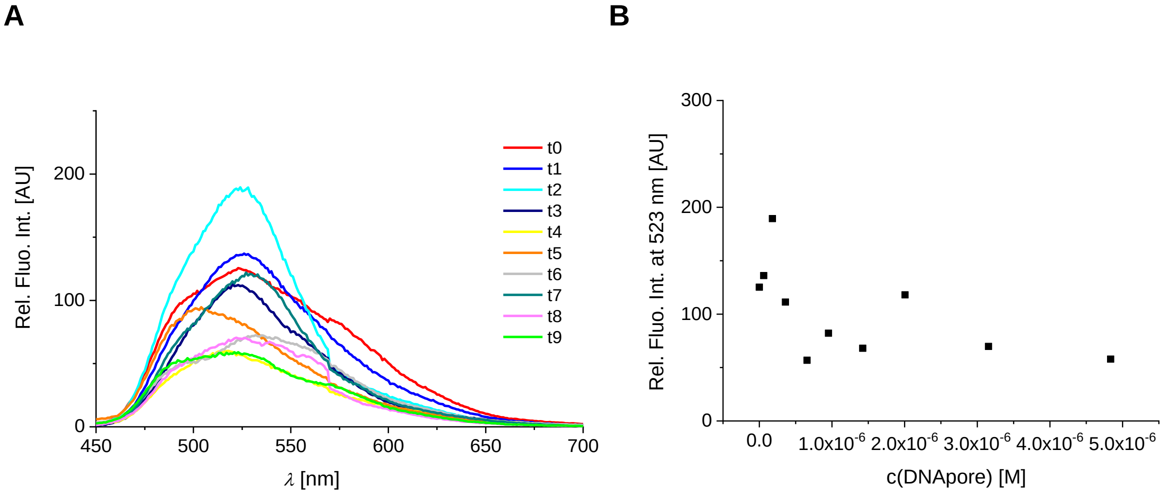

Figure S132: **A)** Fluorimetric titration of **Cat^{1+}** (c = 5 × 10^{-7} M; λ_{exc} = 414 nm; pH 8, 15 mM Tris-HCl, 300 mM KCl) with DNApore. **B)** Dependence of fluorescence of **Cat^{1+}** at λ_{max} = 524 nm on c(DNApore). Note that during the measurement, formation of colloids was observed.

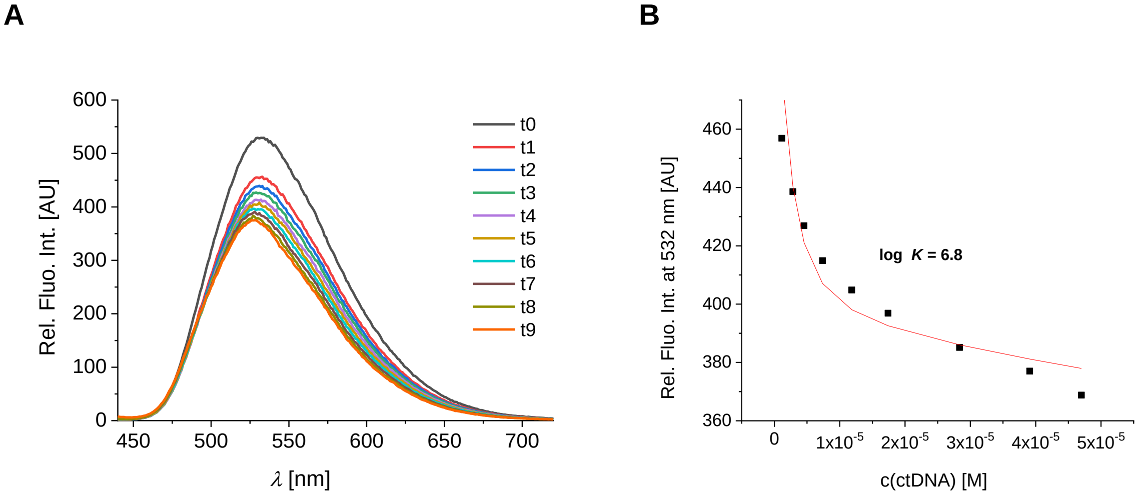

Figure S133: **A)** Fluorimetric titration of **Cat^{2+}** (c = 4 × 10^{-7} M; λ_{exc} = 422 nm; pH 8, 15 mM Tris-HCl, 300 mM KCl) with ctDNA. **B)** Dependence of fluorescence at λ_{max} = 532 nm on c(ctDNA).

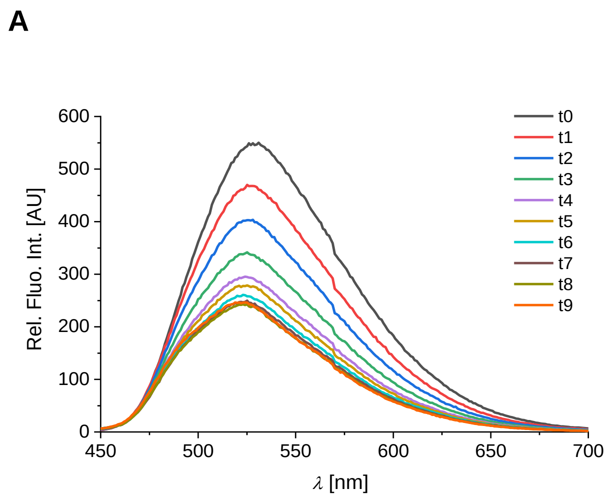

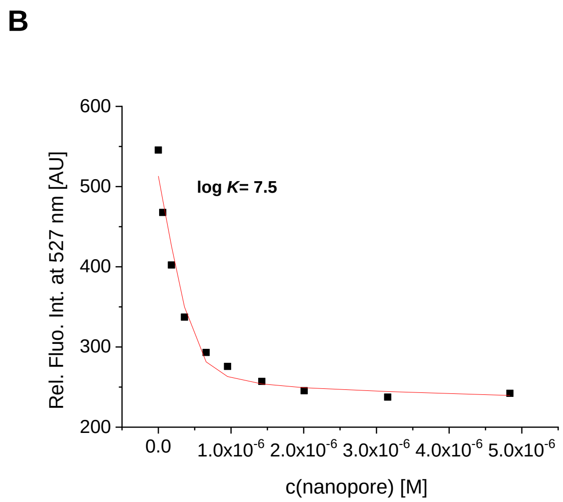

Figure S134: **A)** Fluorimetric titration of **Cat^{2+}** (c = 5 × 10^{-7} M; λ_{exc} = 422 nm; pH 8, 15 mM Tris-HCl, 300 mM KCl) with DNApore. **B)** Dependence of fluorescence of **Cat^{2+}** at λ_{max} = 527 nm on c(DNApore).

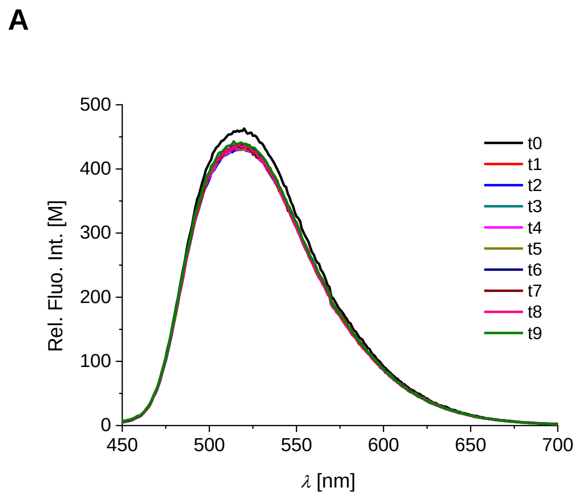

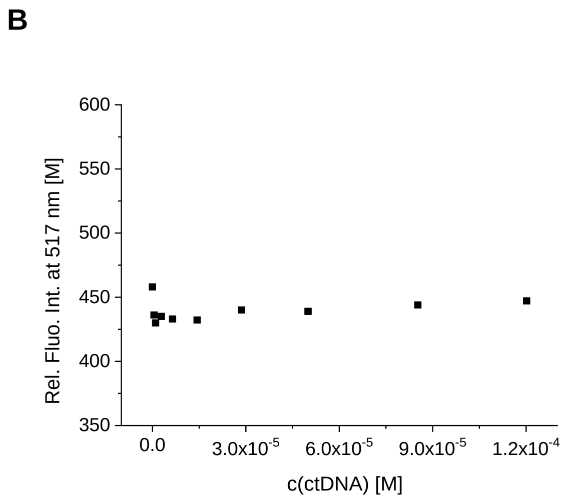

Figure S135: **A)** Fluorimetric titration of **Cat(i)$^{2+}$** (c = 5 × 10^{-7} M; λ_{exc} = 417 nm; pH 8, 15 mM Tris-HCl, 300 mM KCl) with ctDNA. **B)** Dependence of fluorescence of **Cat(i)$^{2+}$** at λ_{max} = 517 nm on c(ctDNA).

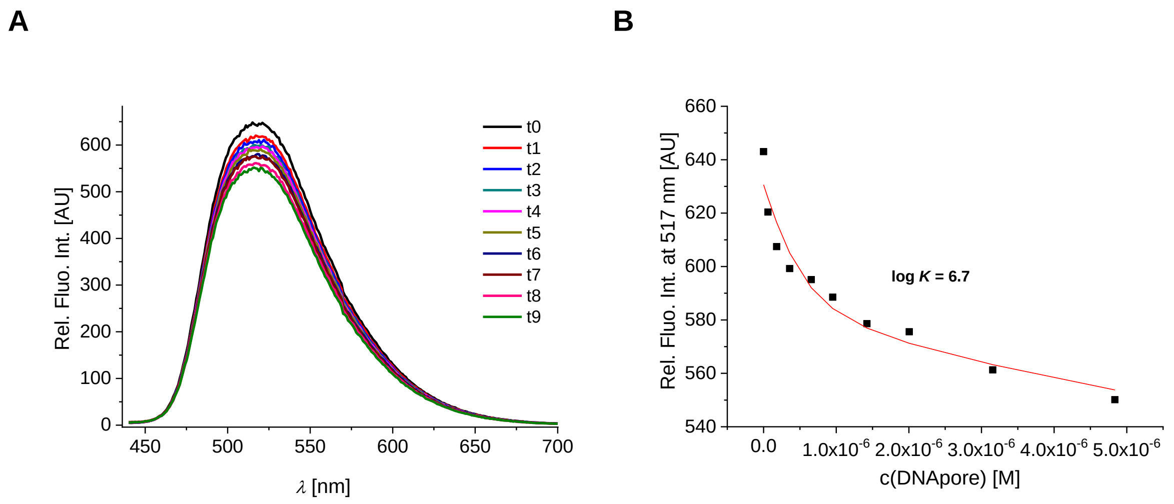

Figure S136: Fluorimetric titration of **Cat(i)$^{2+}$** (c = 5 × 10^{-7} M; λ_{exc} = 417 nm; pH 8, 15 mM Tris-HCl, 300 mM KCl) with DNApore. **B)** Dependence of fluorescence of **Cat(i)$^{2+}$** at λ_{max} = 517 nm on c(DNApore).

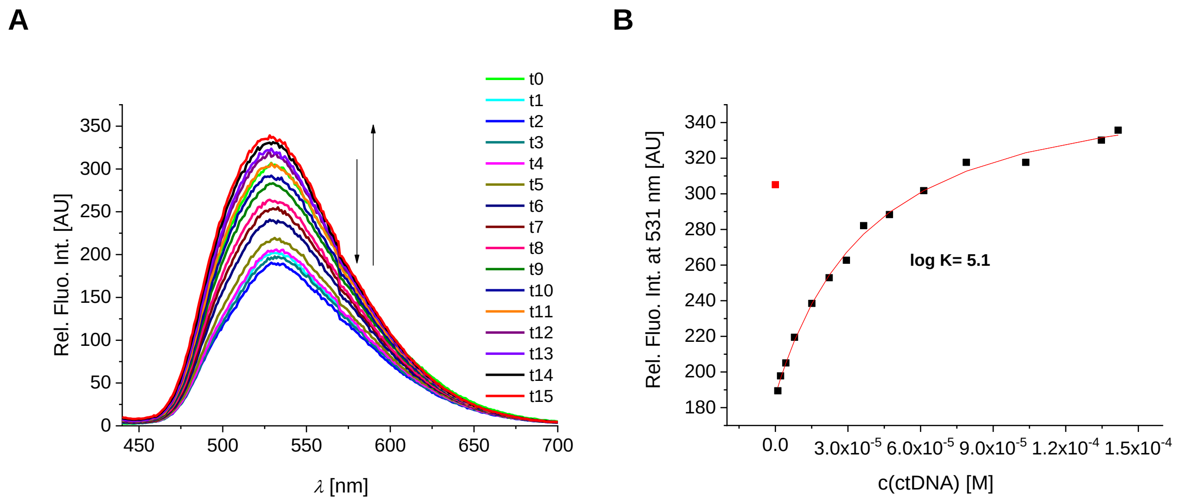

Figure S137: **A)** Fluorimetric titration of **Cat^{3+}** (c = 5×10^{-7} M; λ_{exc} = 421 nm; pH 8, 15 mM Tris-HCl, 300 mM KCl) with ctDNA. **B)** Dependence of fluorescence of **Cat^{3+}** at λ_{max} = 531 nm on c(ctDNA).

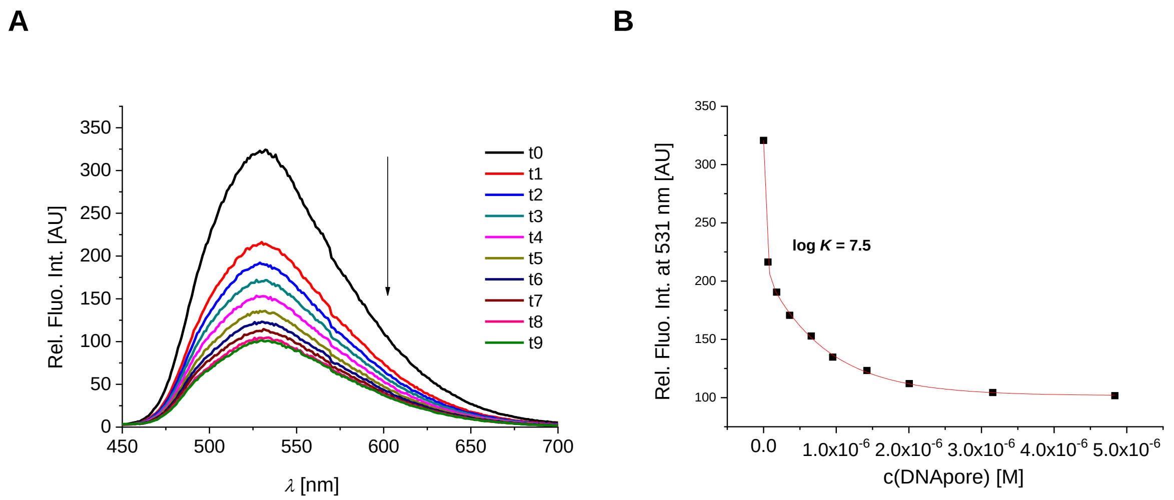

Figure S138: **A)** Fluorimetric titration of **Cat^{3+}** (c = 5 × 10^{-7} M; λ_{exc} = 421 nm; pH 8, 15 mM Tris-HCl, 300 mM KCl) with DNApore. **B)** Dependence of fluorescence of **Cat^{3+}** at λ_{max} = 531 nm on c(DNApore).

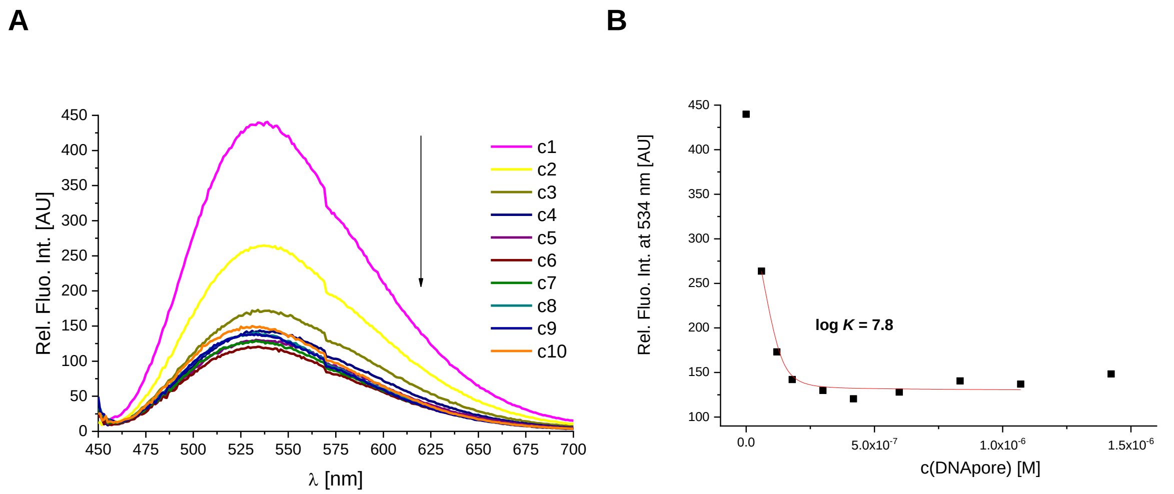

Figure S139: **A)** Fluorimetric titration of **Cat^{4+}** (c = 4 × 10^{-7} M; λ_{exc} = 425 nm; pH 8, 15 mM Tris-HCl, 300 mM KCl) with DNApore. **B)** Dependence of fluorescence of **Cat^{4+}** at λ_{max} = 534 nm on c(DNApore).

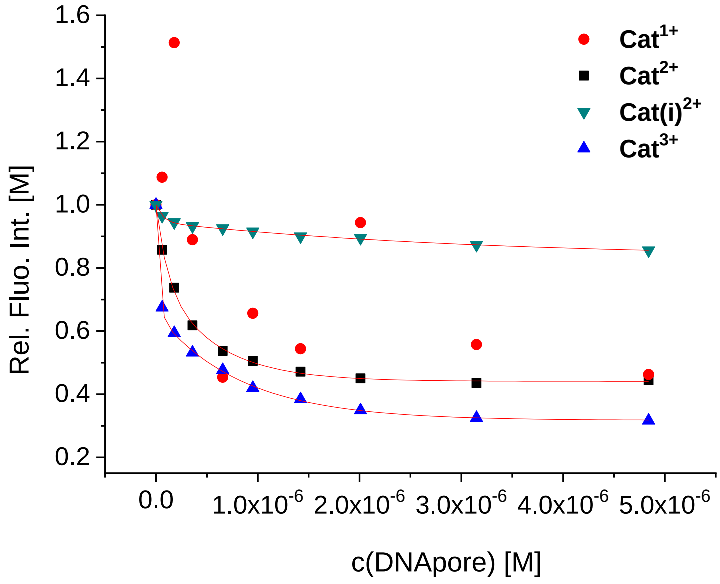

Figure S140: Normalized fluorescence intensities of *bis*-triarylboranes (c(compound) = 5×10^{-7} M; pH 8, 15 mM Tris-HCl, 300 mM KCl) upon addition of DNApore.

Circular Dichroism Experiments

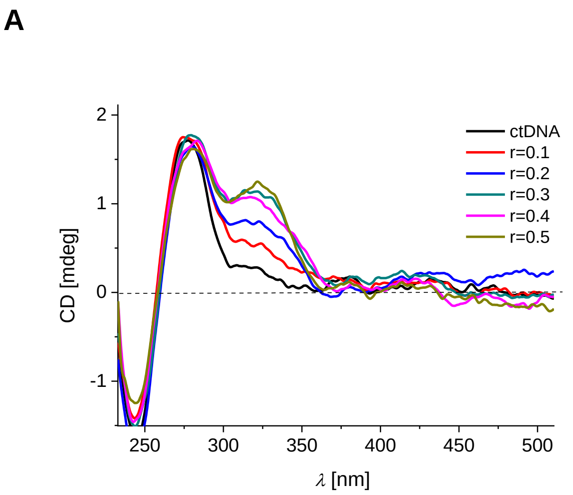

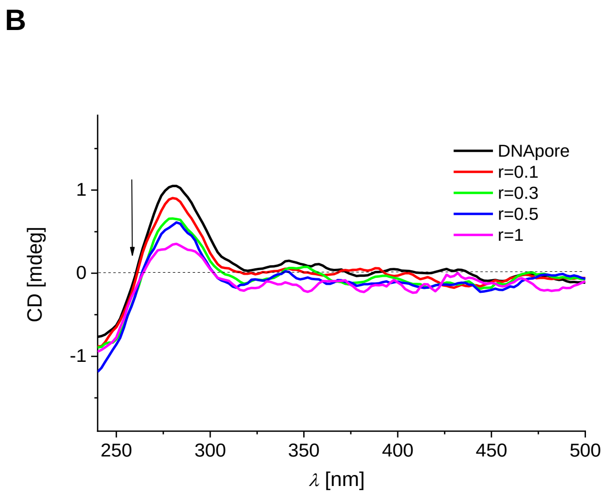

Figure S141: **A)** CD titration of ctDNA (c(ctDNA) = 2 × 10^{-5} M; pH 8, 15 mM Tris-HCl, 300 mM KCl) with **Cat^{1+}** at molar ratios $r_{[compound]/[polynucleotide]}$ = 0.1 – 0.5. **B)** CD titration of DNApore (c(DNApore) = 2.5 × 10^{-5} M; pH 8, 15 mM Tris-HCl, 300 mM KCl) with **Cat^{1+}** at molar ratios $r_{[compound]/[polynucleotide]}$ = 0.1 – 1.

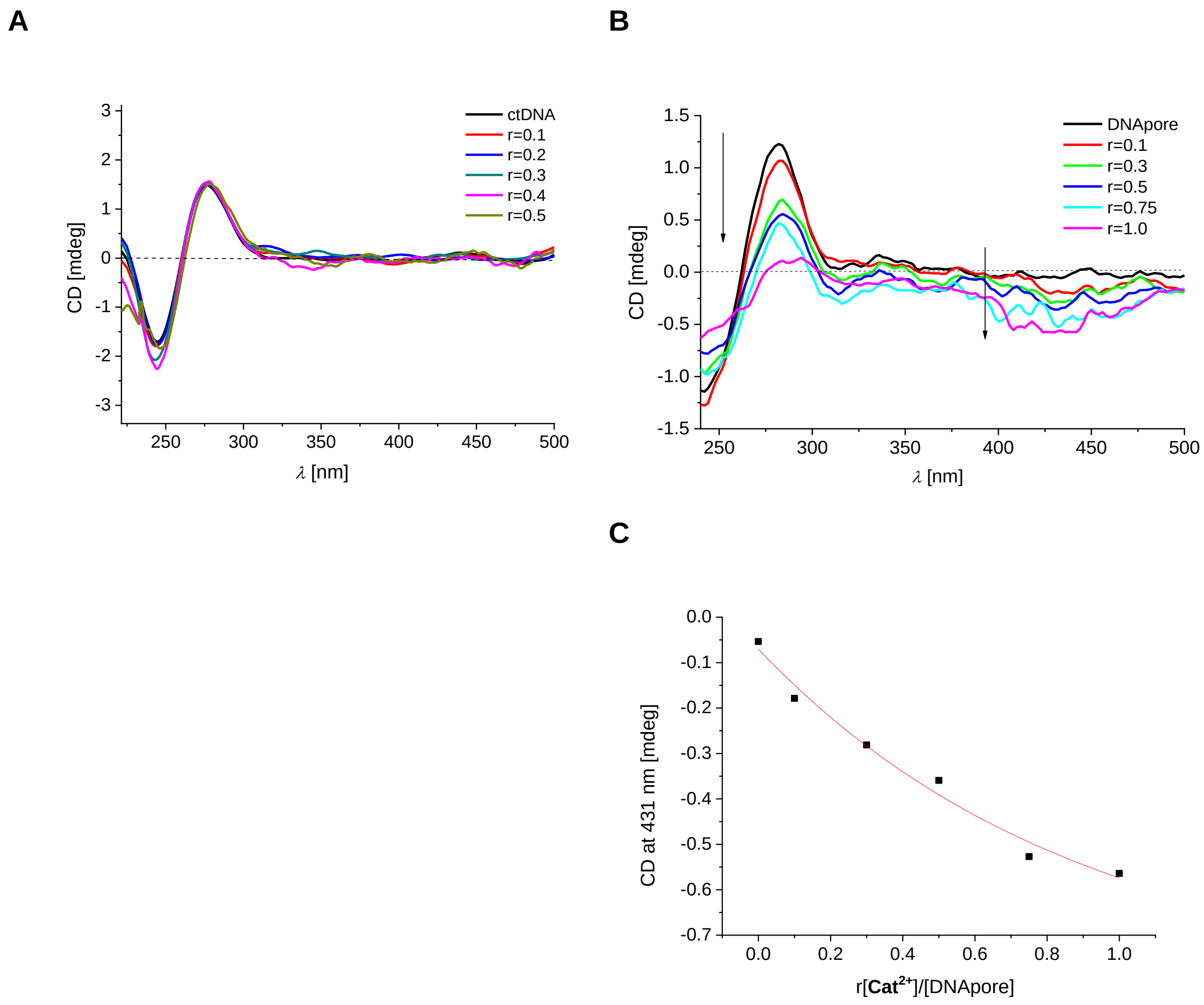

Figure S142: **A)** CD titration of ctDNA (c(ctDNA) = 2 × 10^{-5} M; pH 8, 15 mM Tris-HCl, 300 mM KCl) with **Cat^{2+}** at molar ratios $r_{[compound]/[polynucleotide]}$ = 0.1 – 0.5. **B)** CD titration of DNApore (c(DNApore) = 5 × 10^{-5} M; pH 8, 15 mM Tris-HCl, 300 mM KCl) with **Cat^{2+}** at molar ratios $r_{[compound]/[polynucleotide]}$ = 0.1 – 1. **C)** Dependence of CD signal of DNApore at λ_{max} = 431 nm on c(**Cat^{2+}**).

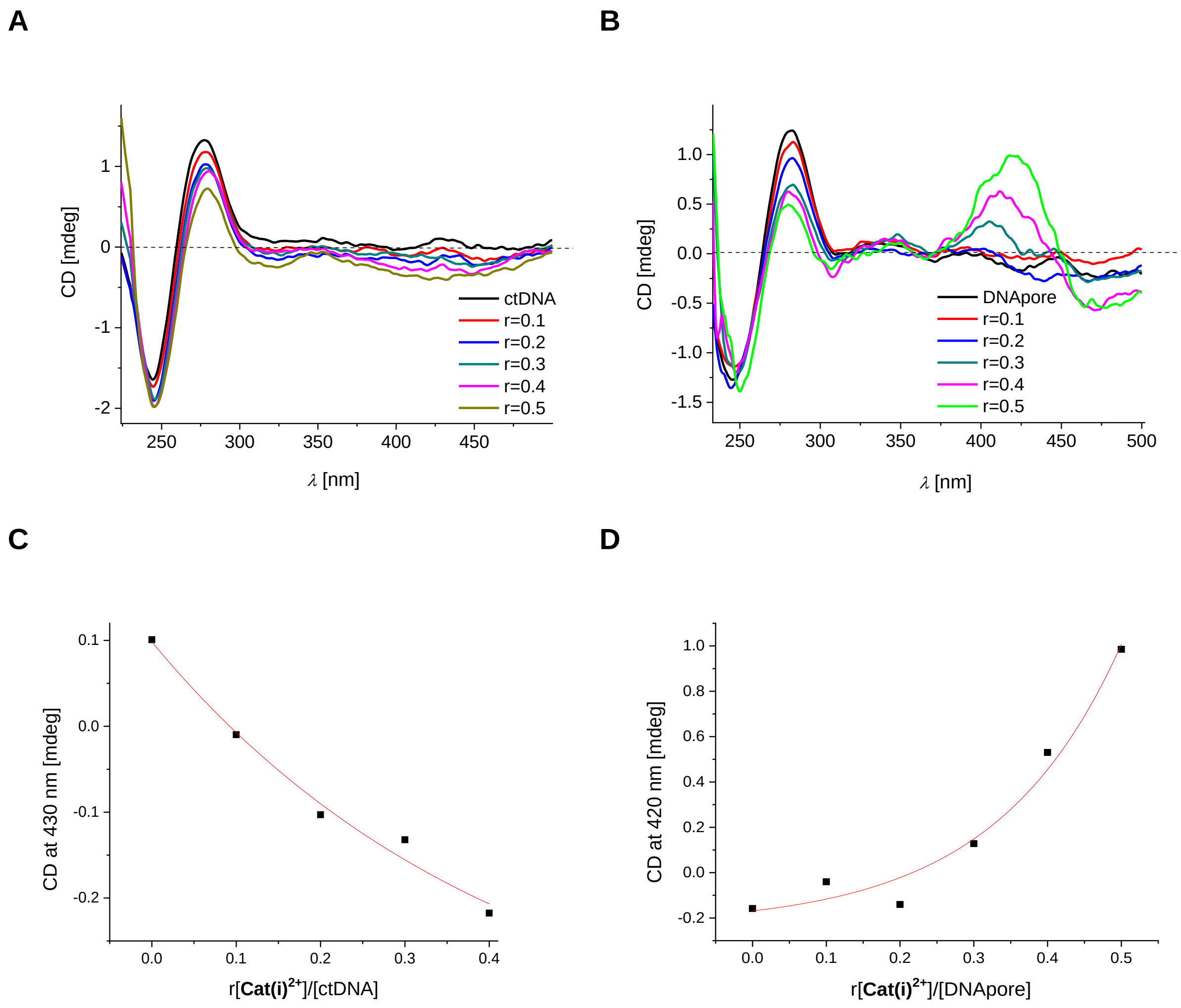

Figure S143: **A)** CD titration of ctDNA (c(ctDNA) = 2 × 10^{-5} M; pH 8, 15 mM Tris-HCl, 300 mM KCl) with **Cat(i)**$^{2+}$ at molar ratios $r_{[compound]/[polynucleotide]}$ = 0.1 – 0.5. **B)** CD titration of DNApore (c(DNApore) = 5 × 10^{-5} M; pH 8, 15 mM Tris-HCl, 300 mM KCl) with **Cat(i)**$^{2+}$ at molar ratios $r_{[compound]/[polynucleotide]}$ = 0.1 – 0.5. **C)** Dependence of CD signal of ctDNA at λ_{max} = 430 nm on c(**Cat(i)**$^{2+}$). **D)** Dependence of CD signal of DNApore at λ_{max} = 420 nm on c(**Cat(i)**$^{2+}$).

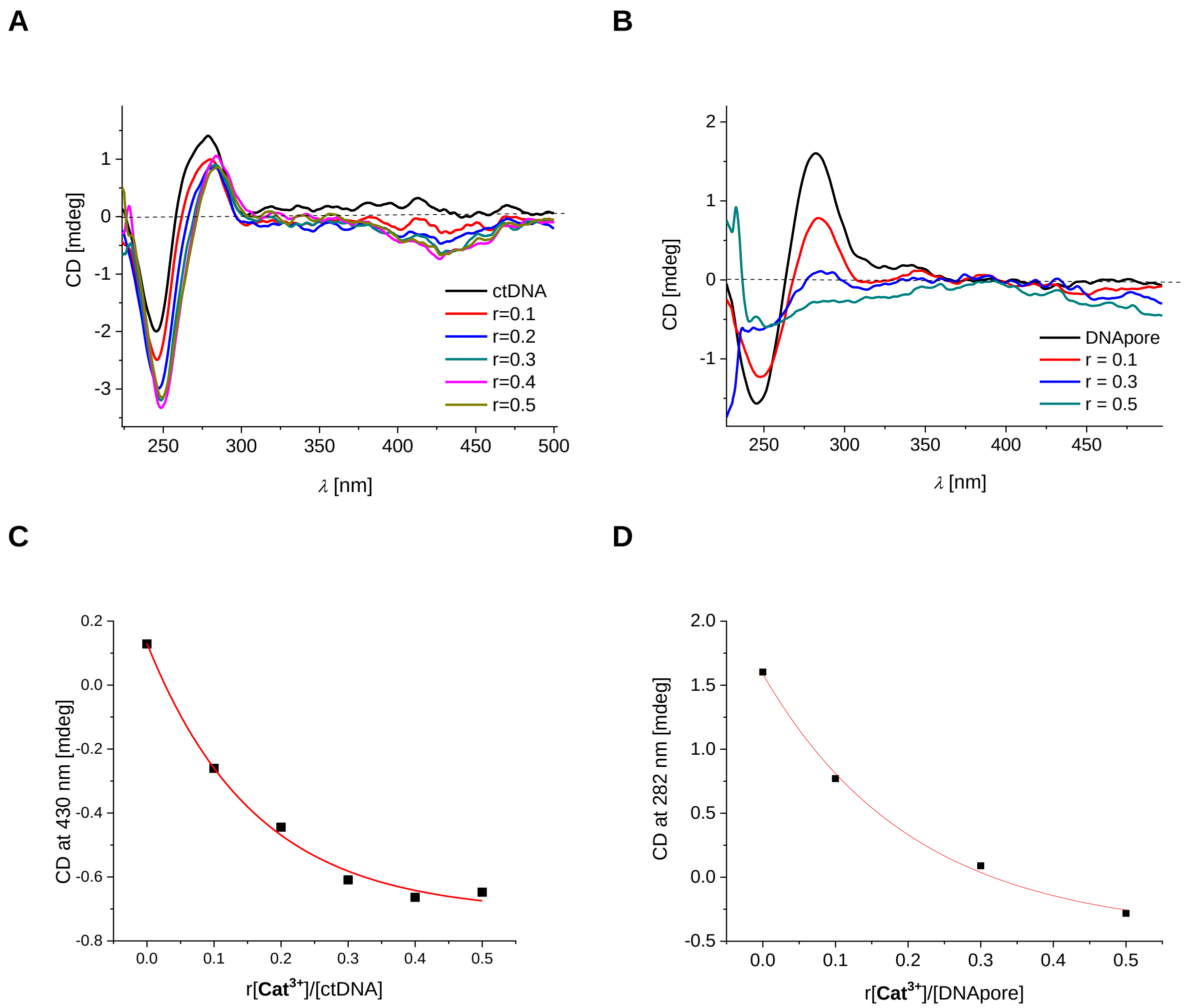

Figure S144: **A)** CD titration of ctDNA (c(ctDNA) = 2×10^{-5} M; pH 8, 15 mM Tris-HCl, 300 mM KCl) with **Cat^{3+}** at molar ratios $r_{[compound]/[polynucleotide]}$ = 0.1 – 0.5. **B)** CD titration of DNApore (c(DNApore) = 5×10^{-5} M; pH 8, 15 mM Tris-HCl, 300 mM KCl) with **Cat^{3+}** at molar ratios $r_{[compound]/[polynucleotide]}$ = 0.1 – 0.5. **C)** Dependence of CD signal of ctDNA at λ_{max} = 430 nm on c(**Cat^{3+}**). **D)** Dependence of CD signal of DNApore at λ_{max} = 282 nm on c(**Cat^{3+}**).

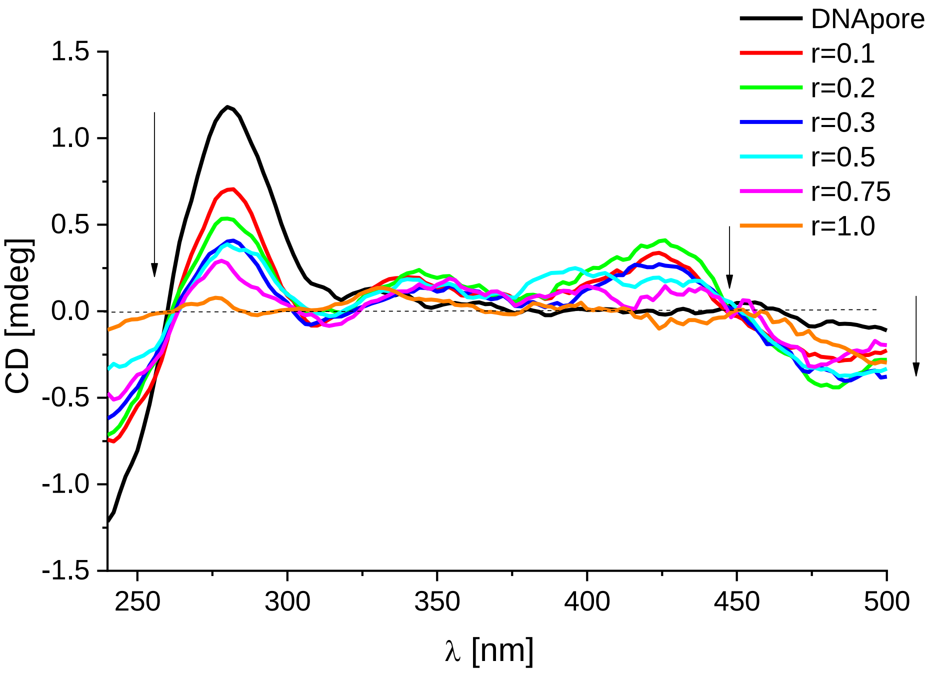

Figure S145: CD titration of DNApore (c(DNApore) = 5 × 10^{-5} M; pH 8, 15 mM Tris-HCl, 300 mM KCl) with **Cat^{4+}** at molar ratios $r_{[compound]/[polynucleotide]}$ = 0.1 – 1.

Cell Studies

MTT Assay

Compounds **Cat(i)$^{2+}$** and **Cat^{3+}** were screened by the MTT assay for anti-proliferative activity against human lung carcinoma (A549) and normal lung (WI38) cell line (Figure S146). Both compounds do not show significant cell toxicity in human A549 and WI38 cells at concentrations of 0.1 µM and 1.0 µM. The cell toxicity of 10 µM **Cat^{3+}** is more pronounced in WI38 cells. The light irradiation increases cell toxicity of both **Cat(i)$^{2+}$** and **Cat^{3+}** at concentrations of 1.0 µM and 10 µM.

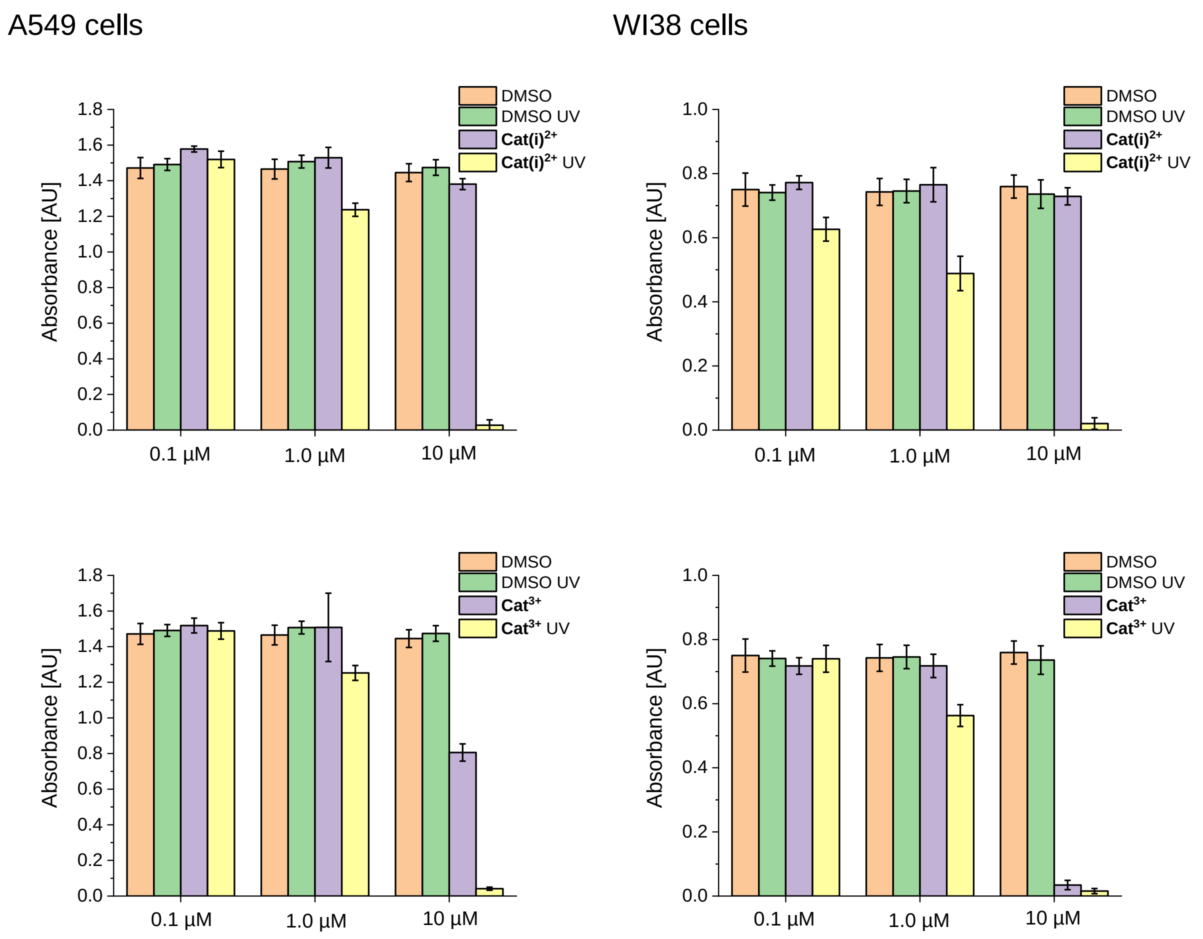

Figure S146: Cell survival of A549 and WI38 cells exposed to **Cat(i)$^{2+}$** and **Cat^{3+}**, with or without UV exposure. Data are presented as mean ±SD made in four replicates, relative to the control samples. Control samples are cells treated with DMSO in same concentration as compound tested. Representative data of three independent experiments which yielded similar results are shown.

Photoinduced Cell Damage

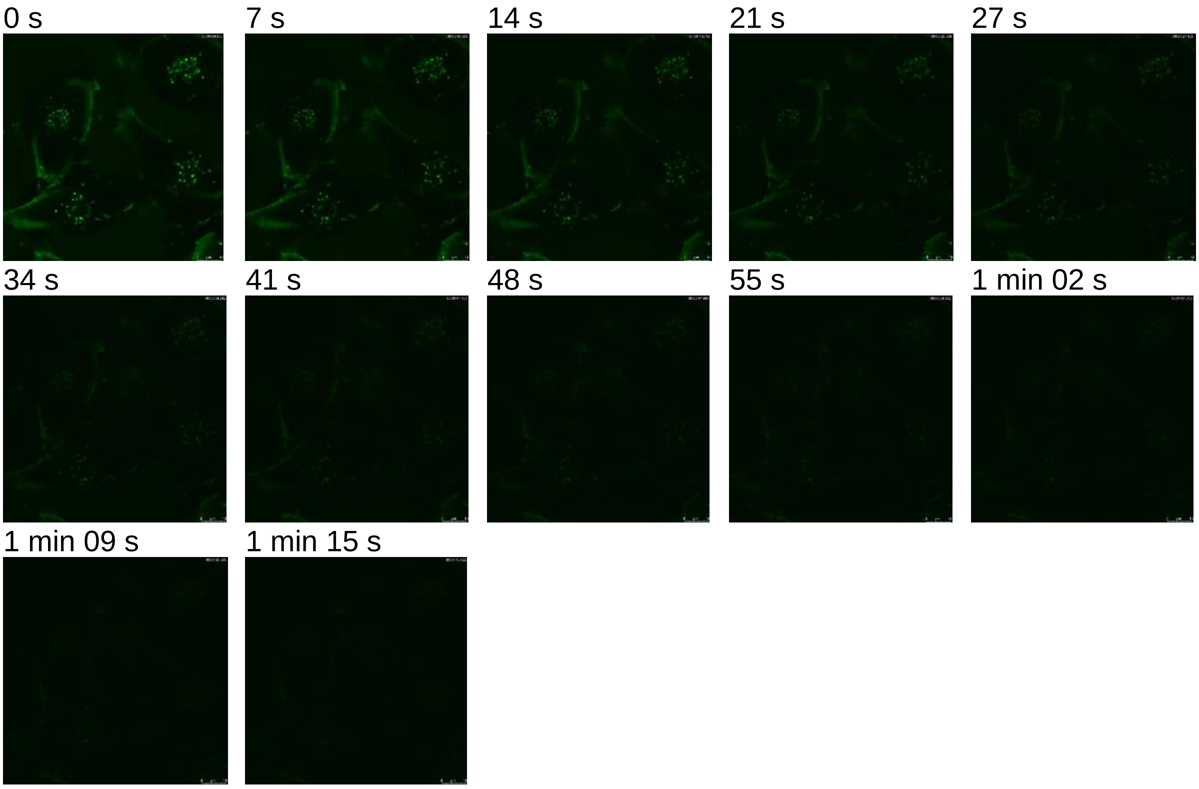

Figure S147: Emission of A549 cells stained with **Cat^{2+}**. Images were taken at times indicated showing fast bleaching of the emission of **Cat^{2+}**.

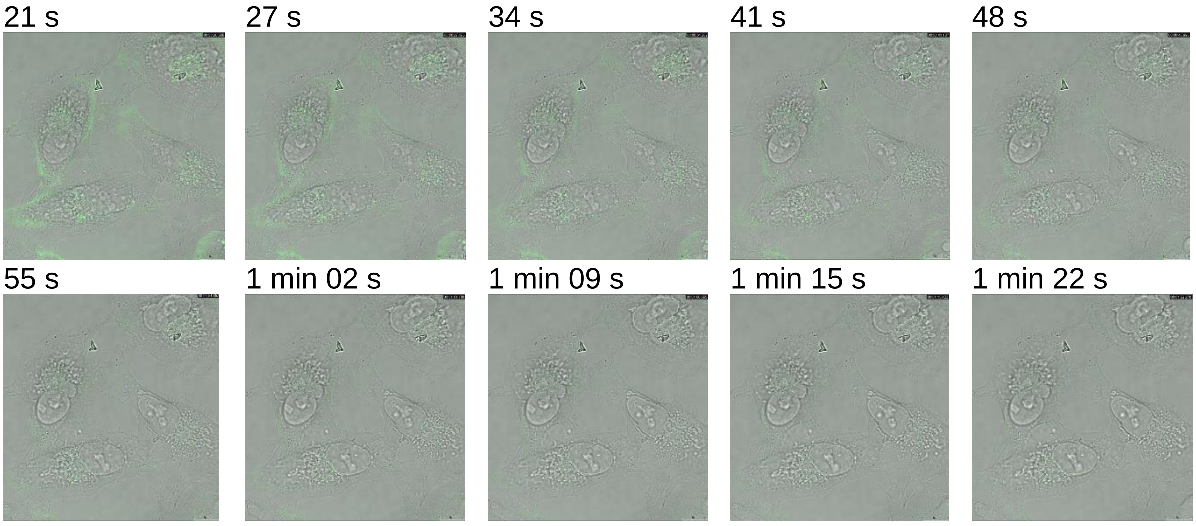

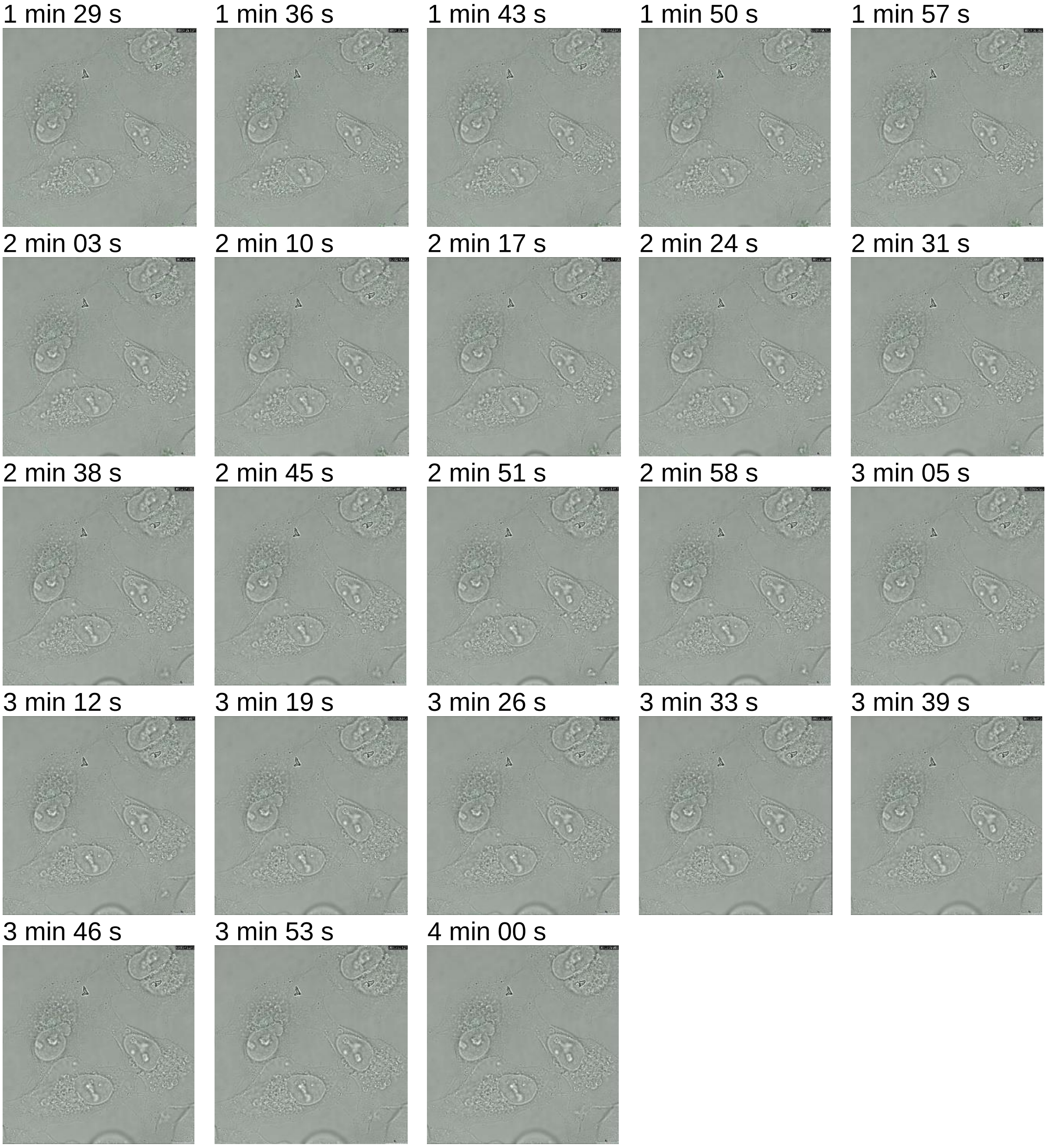

Figure S148: Emission of A549 cells stained with **Cat^{2+}** overlayed with bright field images. Images were taken at times indicated showing fast bleaching of the emission of **Cat^{2+}** and simultaneous cell blebbing.

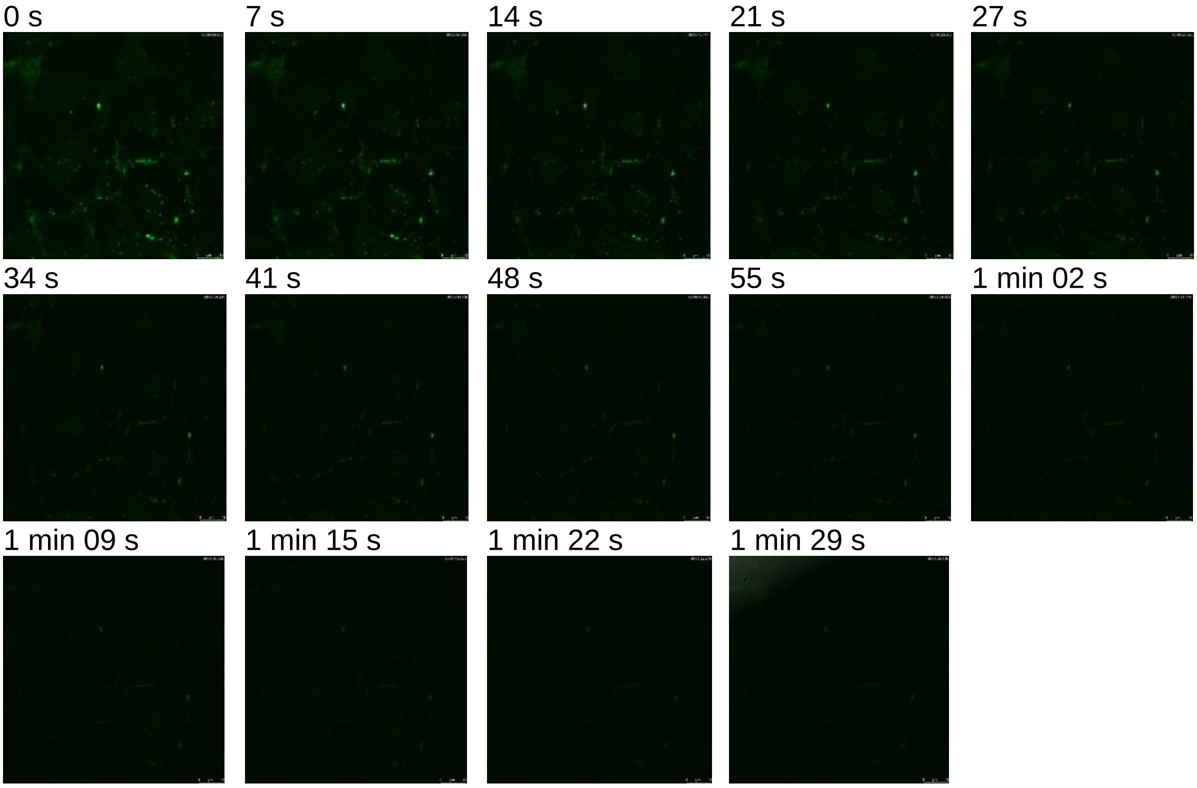

Figure S149: Emission of A549 cells stained with **Cat(i)$^{2+}$**. Images were taken at times indicated showing fast bleaching of the emission of **Cat(i)$^{2+}$**.

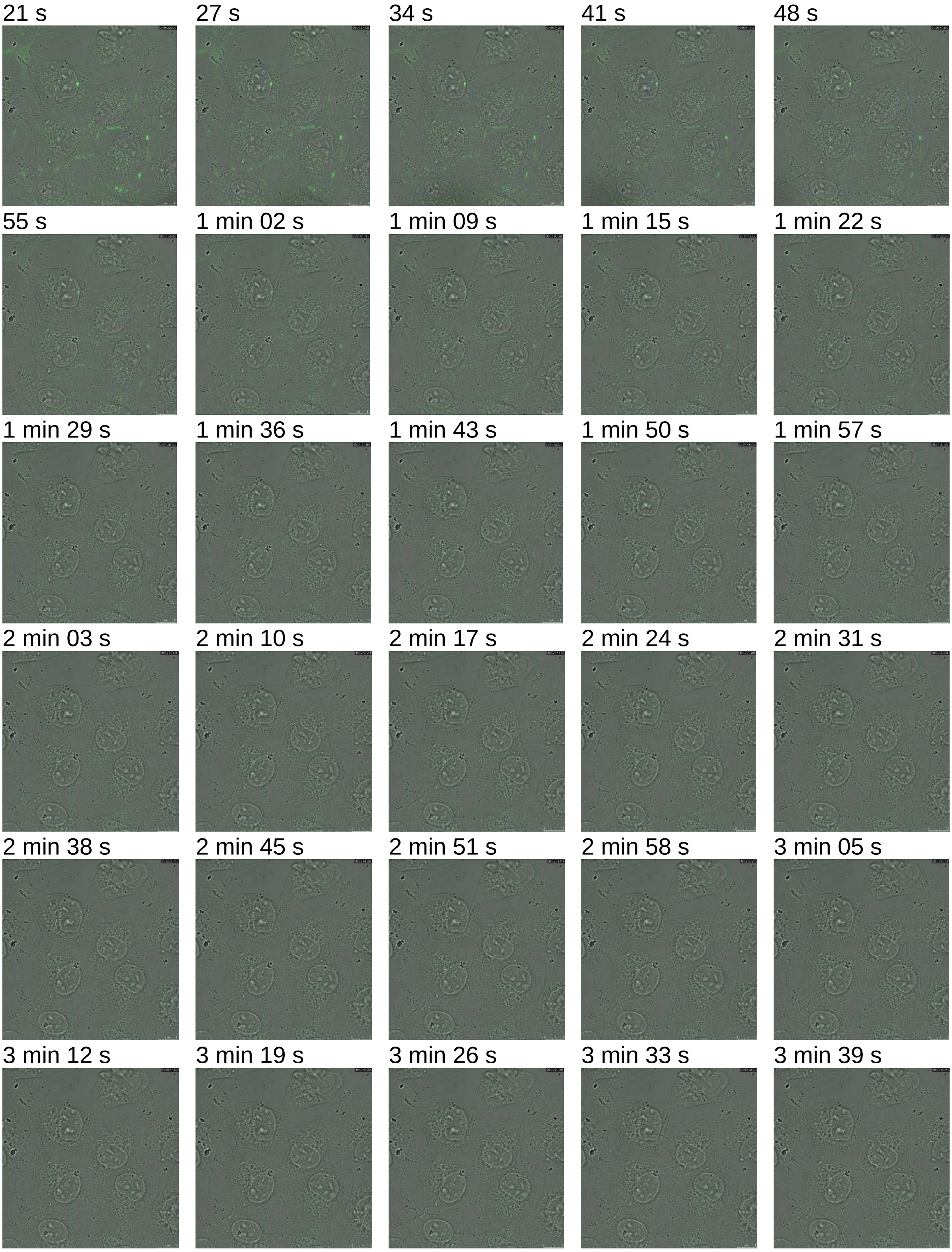

Figure S150: Emission of A549 cells stained with **Cat(i)$^{2+}$** overlayed with bright field images. Images were taken at times indicated showing fast bleaching of the emission of **Cat(i)$^{2+}$** and simultaneous cell blebbing.

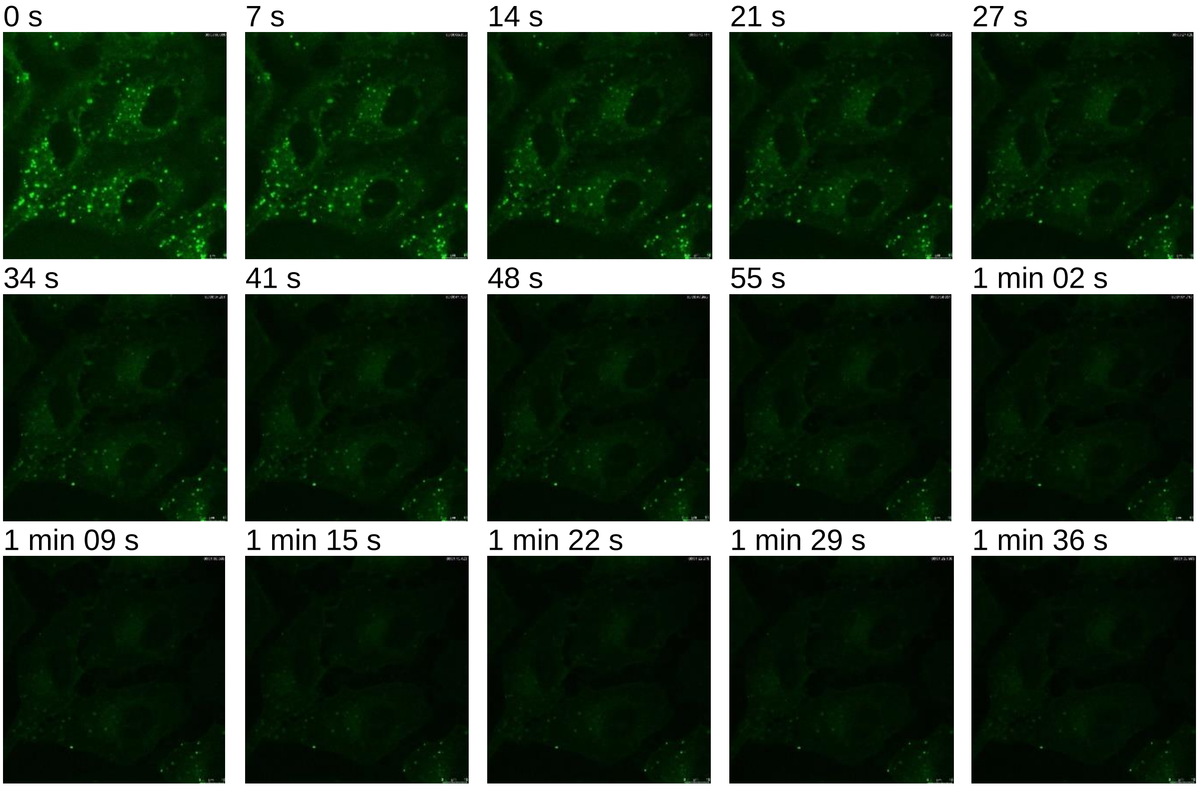

Figure S151: Emission of A549 cells stained with **Cat^{3+}**. Images were taken at times indicated showing fast bleaching of the emission of **Cat^{3+}**.

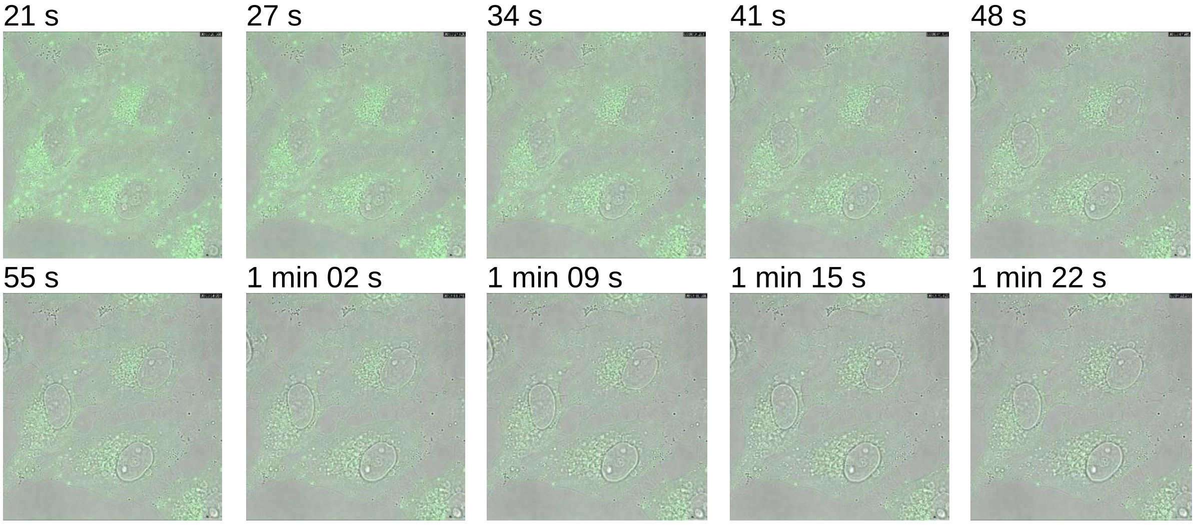

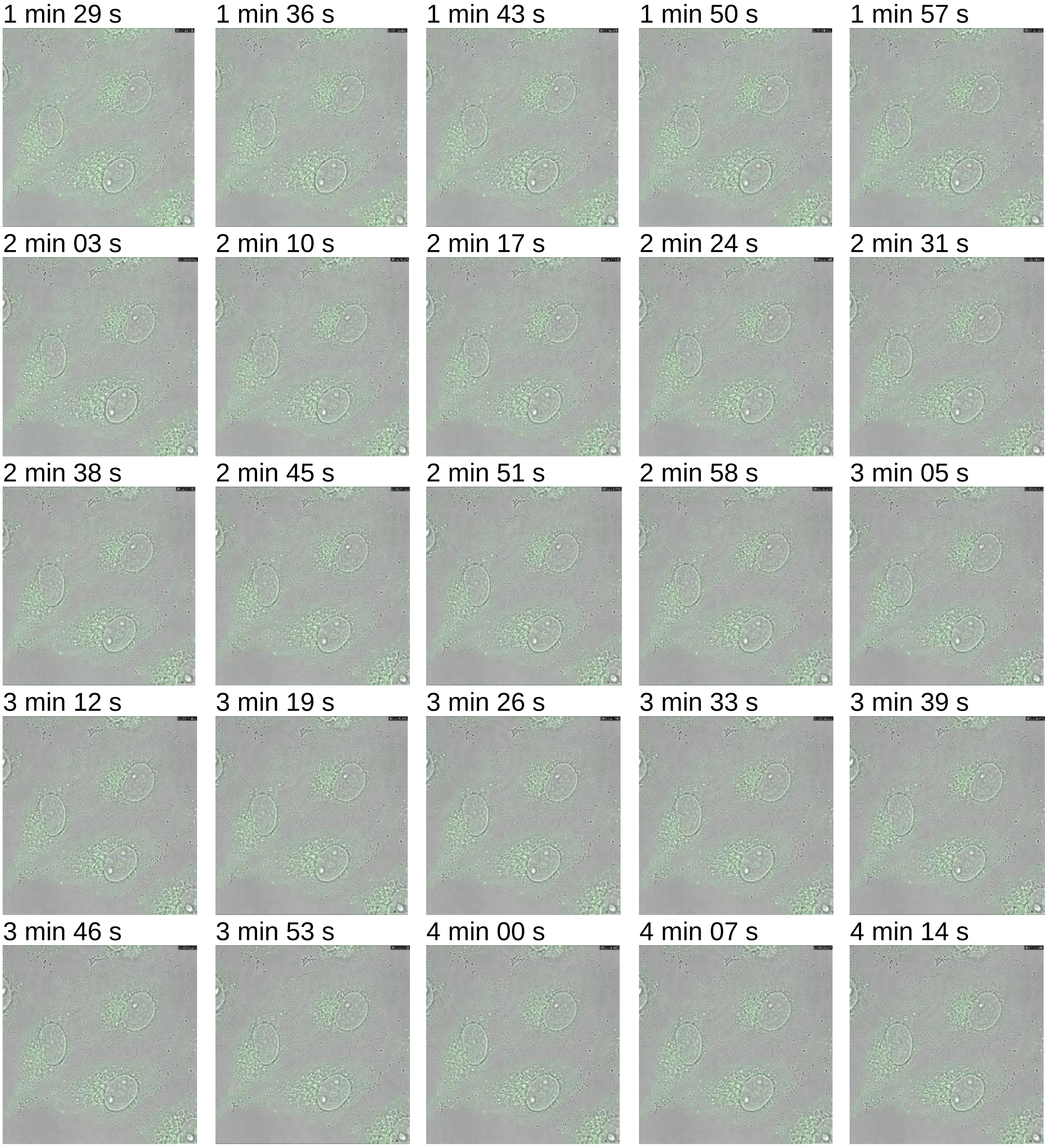

Figure S152: Emission of A549 cells stained with **Cat^{3+}** overlayed with bright field images. Images were taken at times indicated showing fast bleaching of the emission of **Cat^{3+}** and simultaneous cell blebbing.

Co-localization by Confocal Microscopy

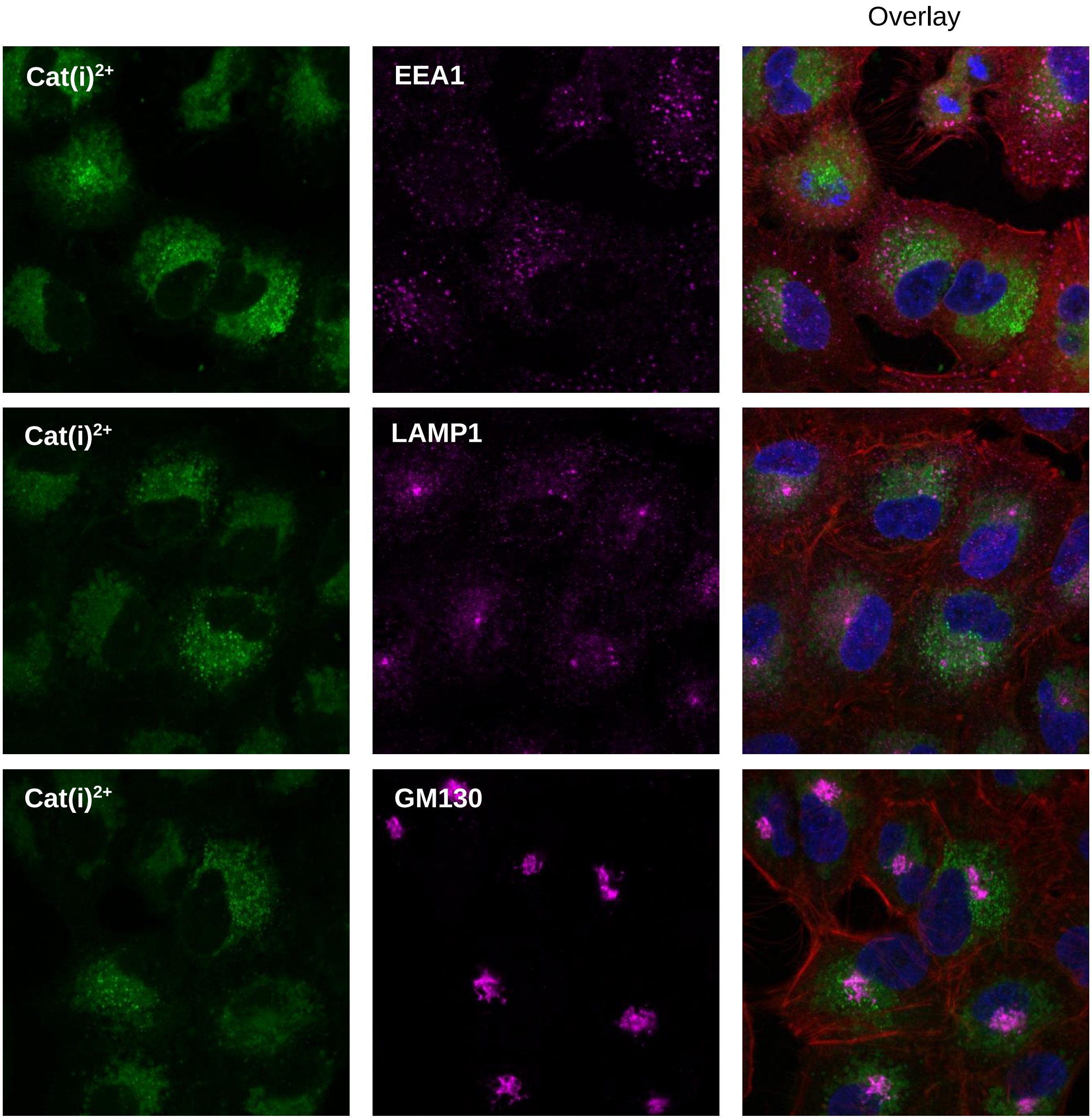

Figure S153: Intracellular localization of **Cat(i)**$^{2+}$ in A549 cells. Co-localization of **Cat(i)**$^{2+}$ with early endosomes (EEA1), golgi (GM130) or lysosomes (LAMP1) observed by confocal microscopy. Cells were treated with 10 µM of **Cat(i)**$^{2+}$ for 90 min at 37 ºC. Nuclei were stained with DAPI. Co-localization was assessed by determining Pearson correlation coefficient. The result is +1 for perfect correlation, 0 for no correlation, and -1 for perfect anti-correlation.

Table S23: Summary of Pearson coefficients determined for **Cat(i)**$^{2+}$.

Commercial Staining Dye	Pearson Coefficient
EEA1	0.24
LAMP1	0.46
GM130	0.37

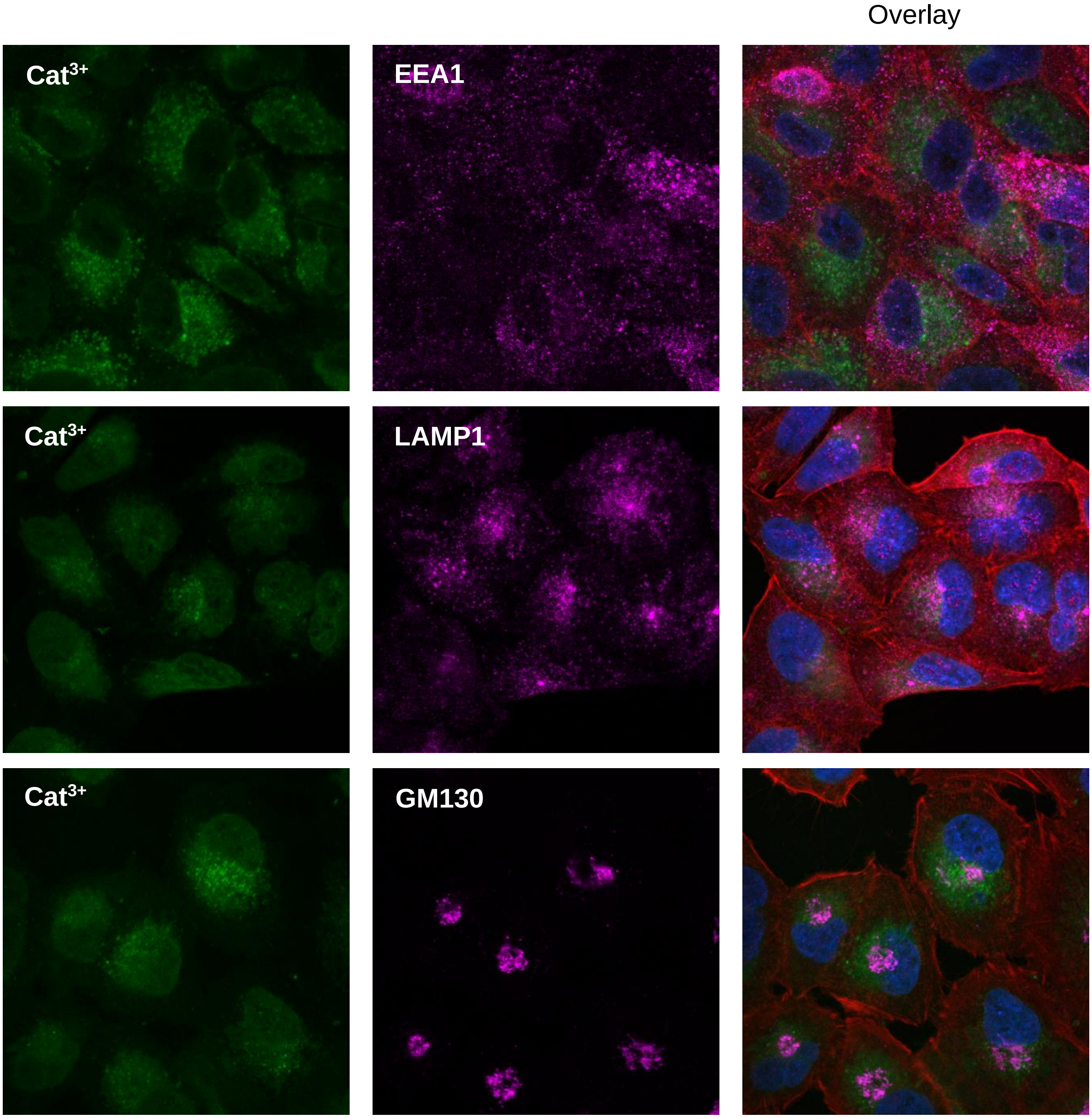

Figure S154: Intracellular localization of **Cat^{3+}** in A549 cells. Co-localization of **Cat^{3+}** with early endosomes (EEA1), lysosomes (LAMP1) or golgi (GM130) observed by confocal microscopy. Cells were treated with 10 µM of **Cat^{3+}** for 90 min at 37 ºC. Nuclei were stained with DAPI. Co-localization was assessed by determining Pearson correlation coefficient. The result is +1 for perfect correlation, 0 for no correlation, and -1 for perfect anti-correlation.

Table S24: Summary of Pearson's coefficients determined for **Cat^{3+}**.

Commercial Staining Dye	Pearson Coefficient
EEA1	0.08
LAMP1	0.54
GM130	0.39

DFT and TD-DFT Calculations

DFT (B3LYP, 6-31G(d,p)) and TD-DFT (CAM-B3LYP, 6-31G(d,p)) calculations were performed for all neutral and cationic triarylboranes and *bis*-triarylboranes which were investigated photophysically. If not stated otherwise, calculations were performed in the gas phase.

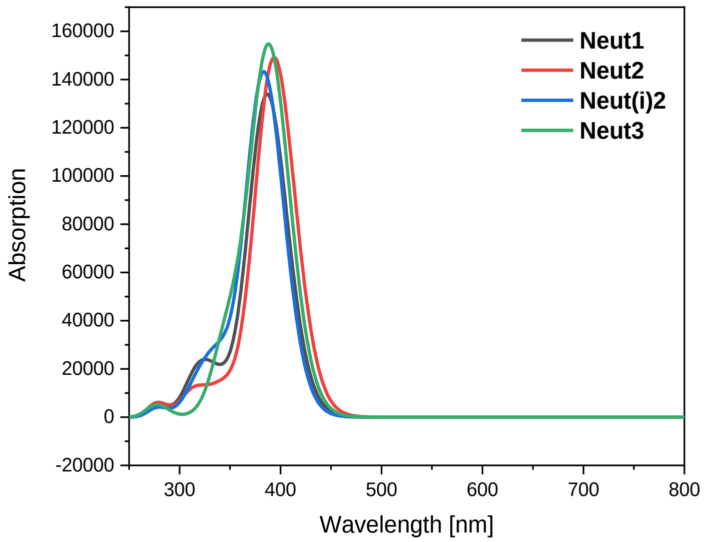

Figure S155: Calculated UV-Vis spectra of neutral compounds **Neut1**-**Neut3** calculated in the gas phase using the CAM-B3LYP functional and the 6-31G(d,p) basis set.

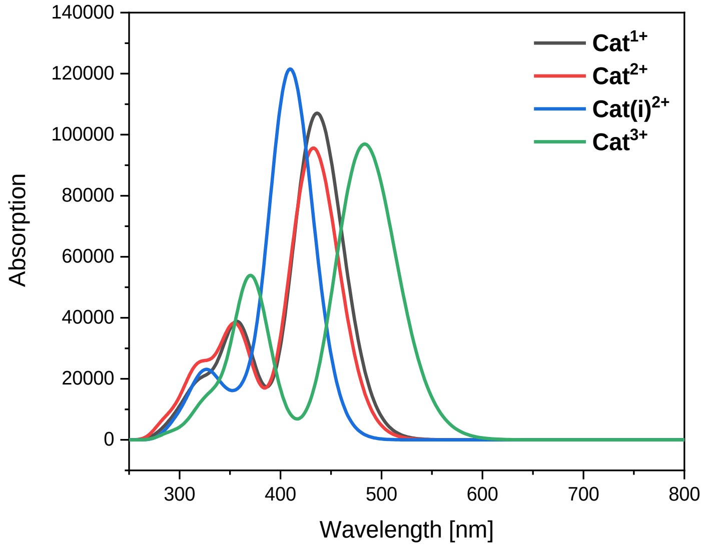

Figure S156: Calculated UV-Vis spectra of cationic compounds **Cat^{1+}**-**Cat^{3+}** calculated in the gas phase using the CAM-B3LYP functional and the 6-31G(d,p) basis set.

Triarylboranes

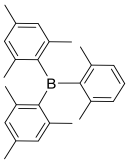

4a

Functional used: TD-DFT CAM-B3LYP 6-31G(d,p), gas phase

Calculated Absorption Spectrum

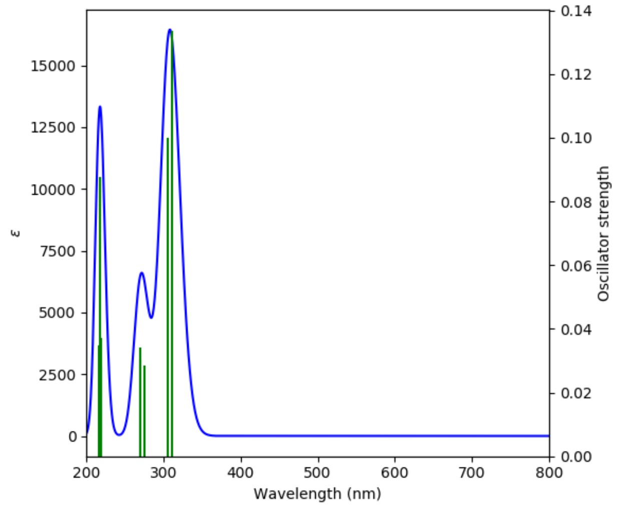

Orbital	Energy [eV]	Symmetry
L+4	0.39	B
L+3	0.33	A
L+2	0.00	A
L+1	-0.01	B
LUMO	-1.60	B
HOMO	-5.94	A
H-1	-6.02	B
H-2	-6.17	A
H-3	-6.26	A
H-4	-6.26	B

Orbitals Relevant for $S_1 \leftarrow S_0$ Transition

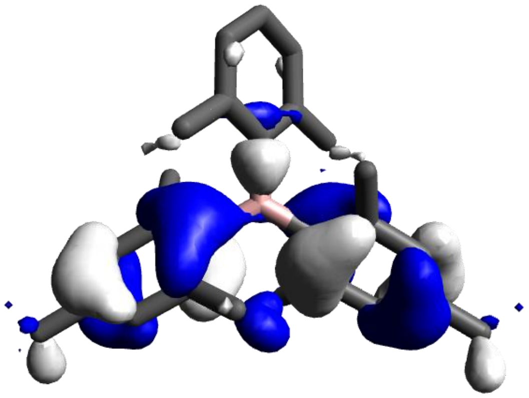

HOMO

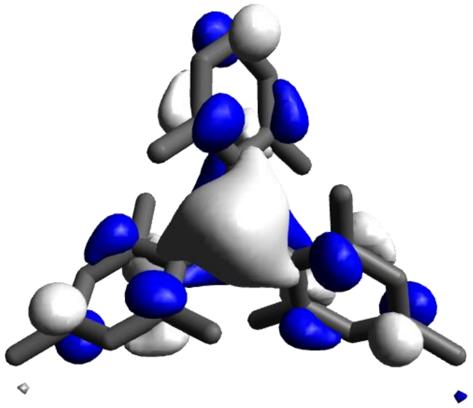

LUMO

Other Relevant Orbitals

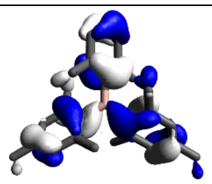

HOMO-1

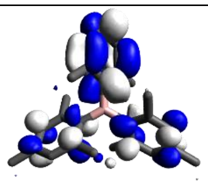

LUMO+1

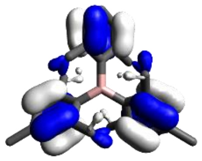

HOMO-2

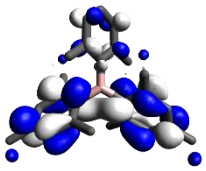

LUMO+2

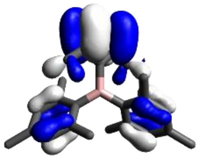

HOMO-3

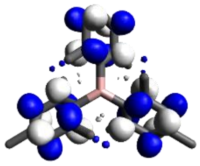

LUMO+3

Table S25: Lowest energy singlet electronic transitions of **4a** (TD-DFT CAM-B3LYP 6-31G(d,p), gas phase).

State	E [eV]	λ [nm]	*f*	Symmetry	Major Contributions	Λ
1	3.98	310	0.134	B	HOMO->LUMO (93%)	0.583
2	4.07	305	0.010	A	H-1->LUMO (91%)	0.634
3	4.51	275	0.028	B	H-2->LUMO (86%)	0.521
4	4.59	270	0.031	A	H-4->LUMO (82%)	0.494
5	4.60	269	0.034	B	H-3->LUMO (84%)	0.506
6	4.74	261	0.000	A	H-5->LUMO (90%)	0.682
7	5.66	219	0.037	B	H-4->L+2 (17%), H-1->L+3 (10%), HOMO->L+1 (12%), HOMO->L+4 (19%)	0.639
8	5.66	219	0.028	A	H-4->LUMO (14%), H-2->L+2 (12%), HOMO->L+3 (18%)	0.655
9	5.71	217	0.088	B	H-6->LUMO (40%)	0.625
10	5.74	216	0.035	B	H-6->LUMO (28%), H-3->L+1 (12%), H-1->L+2 (15%)	0.651

4a

TD-DFT CAM-B3LYP 6-31G(d,p), gas phase, S_0

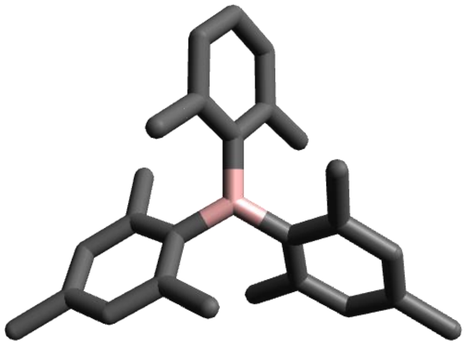

Point group: C_2

Total energy: -648639.32 kcal/mol

Dipole moment: 0.55 D

Imaginary frequencies: 0

Optimized x, y, z coordinates

```
C     0.         0.         4.64884
C    -0.92274   0.77092   3.94848
C    -0.9257    0.79423   2.55046
C     0.         0.         1.82789
C     0.9257   -0.79423   2.55046
C     0.92274  -0.77092   3.94848
B     0.         0.         0.25435
C    -0.00469  -1.36079  -0.52917
C     0.00469   1.36079  -0.52917
C    -0.90551   1.58952  -1.59185
C    -0.90433   2.81482  -2.26233
C     0.         3.82913  -1.94322
C     0.9035    3.5958   -0.90492
C     0.91092   2.39671  -0.18901
C     0.90551  -1.58952  -1.59185
C     0.90433  -2.81482  -2.26233
C     0.        -3.82913  -1.94322
C    -0.9035   -3.5958   -0.90492
```

C	-0.91092	-2.39671	-0.18901
C	1.92213	2.24979	0.9286
C	-1.91939	0.55122	-2.02528
C	1.91939	-0.55122	-2.02528
C	-1.92213	-2.24979	0.9286
C	0.01916	5.12506	-2.71344
C	-1.94973	1.66915	1.85841
C	1.94973	-1.66915	1.85841
C	-0.01916	-5.12506	-2.71344
H	0.	0.	5.73497
H	-1.64982	1.36932	4.49081
H	1.64982	-1.36932	4.49081
H	-1.62666	2.98088	-3.05839
H	1.61801	4.37223	-0.64084
H	1.62666	-2.98088	-3.05839
H	-1.61801	-4.37223	-0.64084
H	2.66274	3.05267	0.88123
H	1.44383	2.28772	1.91137
H	2.46085	1.2991	0.88068
H	-2.45189	0.10841	-1.17878
H	-2.66454	0.99942	-2.68808
H	-1.44385	-0.27574	-2.56015
H	2.45189	-0.10841	-1.17878
H	2.66454	-0.99942	-2.68808
H	1.44385	0.27574	-2.56015
H	-2.66274	-3.05267	0.88123
H	-1.44383	-2.28772	1.91137
H	-2.46085	-1.2991	0.88068
H	-0.97249	5.37322	-3.10273
H	0.35973	5.95609	-2.08885
H	0.69976	5.06297	-3.57171
H	-2.70343	2.0121	2.57241
H	-1.48557	2.55018	1.40614
H	-2.4714	1.14428	1.05293
H	2.4714	-1.14428	1.05293
H	2.70343	-2.0121	2.57241
H	1.48557	-2.55018	1.40614
H	0.97249	-5.37322	-3.10273
H	-0.35973	-5.95609	-2.08885
H	-0.69976	-5.06297	-3.57171

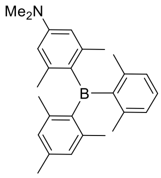

5a

Functional used: TD-DFT CAM-B3LYP 6-31G(d,p), gas phase

Calculated Absorption Spectrum

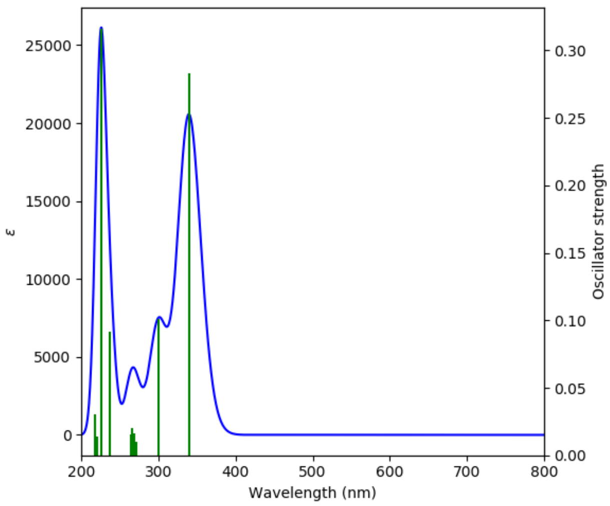

Orbital	Energy [eV]	Symmetry
L+4	0.52	A
L+3	0.48	A
L+2	0.30	A
L+1	0.12	A
LUMO	-1.36	A
HOMO	-5.02	A
H-1	-5.83	A
H-2	-6.03	A
H-3	-6.05	A
H-4	-6.12	A

Orbitals Relevant for $S_1 \leftarrow S_0$ Transition

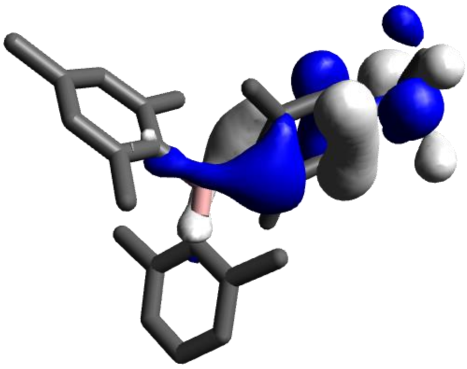

HOMO

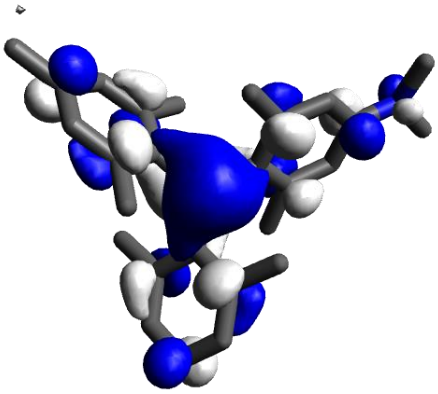

LUMO

Other Relevant Orbitals

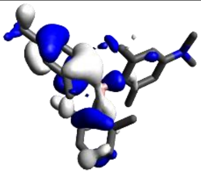

HOMO-1

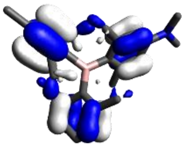

HOMO-2

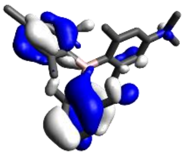

HOMO-3

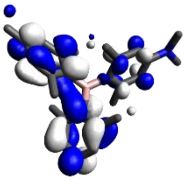

LUMO+1

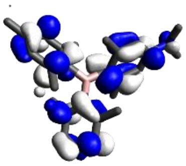

LUMO+2

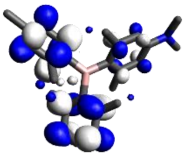

LUMO+3

Table S26: Lowest energy singlet electronic transitions of **5a** (TD-DFT CAM-B3LYP 6-31G(d,p), gas phase).

State	E [eV]	λ [nm]	*f*	Symmetry	Major Contributions	Λ
1	3.65	339	0.283	A	HOMO->LUMO (87%)	0.495
2	4.13	300	0.101	A	H-1->LUMO (87%)	0.572
3	4.57	271	0.010	A	H-2->LUMO (66%), HOMO->L+4 (11%)	0.511
4	4.61	269	0.016	A	H-3->LUMO (79%)	0.576
5	4.68	265	0.020	A	H-5->LUMO (56%), H-2->LUMO (13%)	0.521
6	4.69	264	0.015	A	H-4->LUMO (83%)	0.580
7	5.24	237	0.091	A	H-5->LUMO (26%), HOMO->L+1 (24%), HOMO->L+4 (33%)	0.495
8	5.50	225	0.316	A	H-7->LUMO (11%), H-6->LUMO (24%), HOMO->L+2 (36%), HOMO->L+5 (11%)	0.565
9	5.64	220	0.014	A	H-1->L+3 (15%), HOMO->L+2 (13%)	0.631
10	5.71	217	0.030	A	H-4->L+1 (14%), HOMO->L+2 (10%)	0.621

5a

TD-DFT CAM-B3LYP 6-31G(d,p), gas phase, S_0

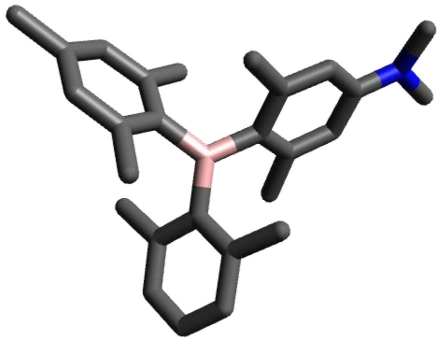

Point group: C_1

Total energy: -708017.08 kcal/mol

Dipole moment: 2.73 D

Imaginary frequencies: 0

Optimized x, y, z coordinates

C	1.97458	4.47391	0.12215
C	2.46312	3.59744	-0.84191
C	2.02787	2.26941	-0.89143
C	1.05428	1.80799	0.02827
C	0.55188	2.71232	0.99726
C	1.02896	4.0262	1.03982
B	0.55137	0.31343	0.00103
C	-0.97347	-0.00834	-0.0186
C	1.61602	-0.84622	-0.00604
C	1.53668	-1.88591	-0.96622
C	2.50962	-2.88818	-0.99462

C	3.55867	-2.92406	-0.07443
C	3.62729	-1.90493	0.87694
C	2.69123	-0.86889	0.91603
C	-1.52425	-1.03316	0.79779
C	-2.89025	-1.30025	0.79243
C	-3.77907	-0.60477	-0.05134
C	-3.23385	0.40752	-0.86597
C	-1.87634	0.71368	-0.84542
C	2.85963	0.19701	1.97787
C	0.43034	-1.9494	-1.99914
C	-0.67354	-1.85798	1.74259
C	-1.41509	1.82475	-1.76688
C	4.56985	-4.04258	-0.09024
C	2.61154	1.3682	-1.95873
C	-0.49087	2.30315	2.01685
N	-5.12878	-0.90585	-0.0831
C	-6.03969	-0.02945	-0.79676
C	-5.67845	-1.83168	0.89
H	2.32903	5.50002	0.15854
H	3.19699	3.94305	-1.56518
H	0.64888	4.70462	1.79908
H	2.44494	-3.66464	-1.75377
H	4.43605	-1.91323	1.60443
H	-3.26511	-2.06385	1.46223
H	-3.87575	0.9713	-1.53118
H	3.18256	1.14802	1.5452
H	1.92744	0.39833	2.514
H	3.60564	-0.11101	2.71559

H	0.2545	-0.98467	-2.4832
H	0.6785	-2.67431	-2.77942
H	-0.52198	-2.24669	-1.55044
H	0.01764	-1.24229	2.32383
H	-1.30603	-2.40591	2.44644
H	-0.06127	-2.58444	1.20145
H	-1.1518	2.72735	-1.20891
H	-0.52815	1.5452	-2.34129
H	-2.20443	2.08418	-2.47764
H	4.70823	-4.44188	-1.09927
H	5.5427	-3.70709	0.28111
H	4.24785	-4.87512	0.5478
H	3.16768	1.95565	-2.69448
H	3.29248	0.62719	-1.53057
H	1.84016	0.80891	-2.49664
H	-0.25507	1.34824	2.49533
H	-0.57247	3.05866	2.80333
H	-1.4765	2.18264	1.55785
H	-7.05237	-0.42631	-0.71795
H	-6.03857	0.99689	-0.40022
H	-5.78519	0.02052	-1.86121
H	-6.74087	-1.97287	0.68823
H	-5.19369	-2.81106	0.81606
H	-5.5677	-1.47639	1.92581

6a 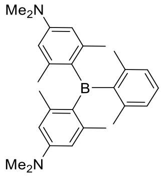

Functional used: TD-DFT CAM-B3LYP 6-31G(d,p), gas phase

Calculated Absorption Spectrum

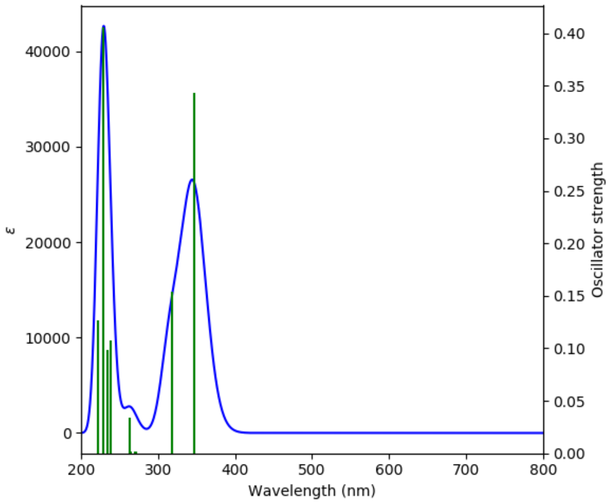

Orbital	Energy [eV]	Symmetry
L+4	0.67	A
L+3	0.61	A
L+2	0.46	A
L+1	0.31	A
LUMO	-1.16	A
HOMO	-4.79	A
H-1	-4.98	A
H-2	-5.89	A
H-3	-5.91	A
H-4	-5.99	A

Orbitals Relevant for $S_1 \leftarrow S_0$ Transition

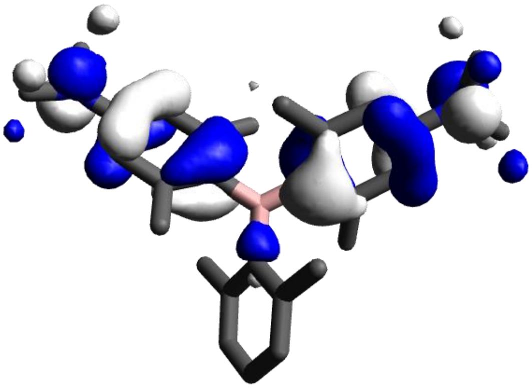

HOMO

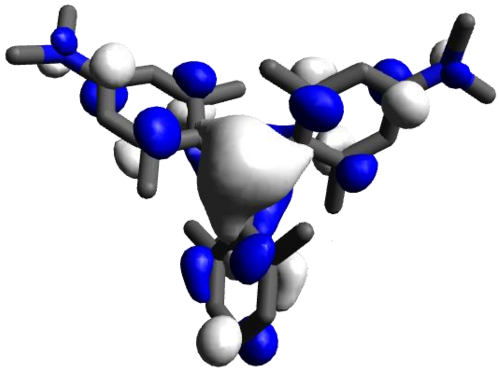

LUMO

Other Relevant Orbitals

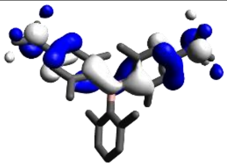

HOMO-1

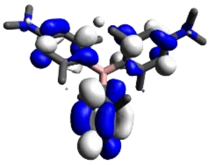

LUMO+1

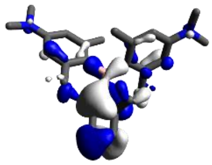

HOMO-2

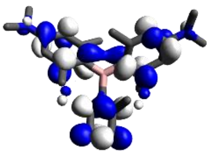

LUMO+2

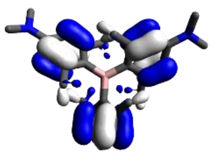

HOMO-3

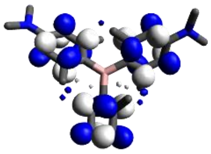

LUMO+3

Table S27: Lowest energy singlet electronic transitions of **6a** (TD-DFT CAM-B3LYP 6-31G(d,p), gas phase).

State	E [eV]	λ [nm]	*f*	Symmetry	Major Contributions	Λ
1	3.58	346	0.343	A	HOMO->LUMO (90%)	0.517
2	3.90	318	0.154	A	H-2->LUMO (12%), H-1->LUMO (81%)	0.575
3	4.57	271	0.001	A	H-2->LUMO (81%), H-1->LUMO (12%)	0.567
4	4.60	270	0.002	A	H-3->LUMO (56%)	0.527
5	4.69	265	0.001	A	H-5->LUMO (52%), H-1->L+4 (10%), HOMO->L+3 (12%)	0.545
6	4.72	263	0.034	A	H-4->LUMO (62%), H-3->LUMO (16%)	0.514
7	5.21	238	0.108	A	H-4->LUMO (14%), H-3->LUMO (19%), H-1->L+3 (12%), HOMO->L+1 (24%), HOMO->L+4 (16%)	0.553
8	5.30	234	0.100	A	H-5->LUMO (44%), H-1->L+1 (14%), H-1->L+4 (11%), HOMO->L+3 (16%)	0.552
9	5.43	229	0.406	A	H-8->LUMO (11%), H-6->LUMO (24%), H-1->L+2 (14%), HOMO->L+1 (25%)	0.606
10	5.61	221	0.126	A	H-1->L+1 (14%), HOMO->L+2 (43%), HOMO->L+5 (21%)	0.641

6a

TD-DFT CAM-B3LYP 6-31G(d,p), gas phase, S_0

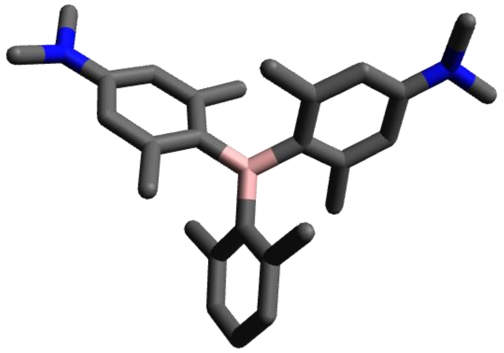

Point group: C_1

Total energy: -767388.41 kcal/mol

Dipole moment: 2.65 D

Imaginary frequencies: 0

Optimized x, y, z coordinates

C	0.07324	5.06539	-0.04675
C	-0.67274	4.39003	0.91463
C	-0.717	2.99235	0.93791
C	0.02686	2.24408	-0.0071
C	0.79481	2.94071	-0.97226
C	0.79637	4.33915	-0.98835
B	0.00032	0.66532	0.01506
C	1.34712	-0.12862	0.03011
C	-1.37287	-0.08217	0.02094
C	-1.61425	-1.16465	0.90791
C	-2.84846	-1.8092	0.93599
C	-3.89934	-1.43898	0.07419
C	-3.65176	-0.38342	-0.82477
C	-2.43589	0.29551	-0.84024
C	1.55225	-1.23784	-0.8323
C	2.76528	-1.92209	-0.84757
C	3.82214	-1.5779	0.01772
C	3.61868	-0.47961	0.87567
C	2.42533	0.23809	0.87722
C	-2.29393	1.42263	-1.84263
C	-0.56514	-1.65899	1.88412
C	0.48912	-1.71326	-1.80264
C	2.32804	1.40266	1.84146
C	-1.56222	2.32376	2.00184
C	1.61815	2.21536	-2.01598
N	5.00984	-2.29258	0.02907
C	6.15138	-1.76017	0.75032
C	5.25357	-3.28204	-1.00399
H	0.09102	6.15141	-0.06206
H	-1.23433	4.95179	1.65655
H	1.37587	4.86114	-1.7454
H	-2.98867	-2.61471	1.64597
H	-4.41601	-0.08042	-1.52941
H	2.88431	-2.73644	-1.55121
H	4.40193	-0.17178	1.55708
H	-2.29313	2.39897	-1.35046
H	-1.36017	1.35824	-2.40784
H	-3.11927	1.40526	-2.55982
H	-0.06918	-0.83907	2.40991
H	-1.01947	-2.31251	2.63417
H	0.22425	-2.22142	1.37771
H	0.02541	-0.88715	-2.34793
H	0.9212	-2.39945	-2.53655
H	-0.32201	-2.23505	-1.28709
H	2.35868	2.36171	1.31728
H	1.39531	1.38951	2.41177
H	3.15546	1.3798	2.55603
H	-1.86463	3.04894	2.76269
H	-2.4667	1.87985	1.57594
H	-1.02906	1.51316	2.5075
H	1.05855	1.40979	-2.5006
H	1.94556	2.90911	-2.79557
H	2.50668	1.75298	-1.57636
H	6.98852	-2.45287	0.65498
H	6.47162	-0.77602	0.37542
H	5.92785	-1.657	1.81762
H	6.21511	-3.7625	-0.8184
H	4.48515	-4.06207	-0.98658
H	5.2729	-2.84873	-2.01606
C	-6.1086	-1.8056	-0.91676
H	-5.7586	-2.07881	-1.92437
H	-7.01775	-2.36988	-0.70489
H	-6.37407	-0.74331	-0.9293
C	-5.25908	-3.3109	0.87554
H	-5.0466	-3.14153	1.93666
H	-6.28642	-3.66924	0.79891
H	-4.58714	-4.10665	0.5192
N	-5.12701	-2.08131	0.11578

5c

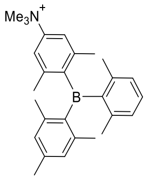

Functional used: TD-DFT CAM-B3LYP 6-31G(d,p), gas phase

Calculated Absorption Spectrum

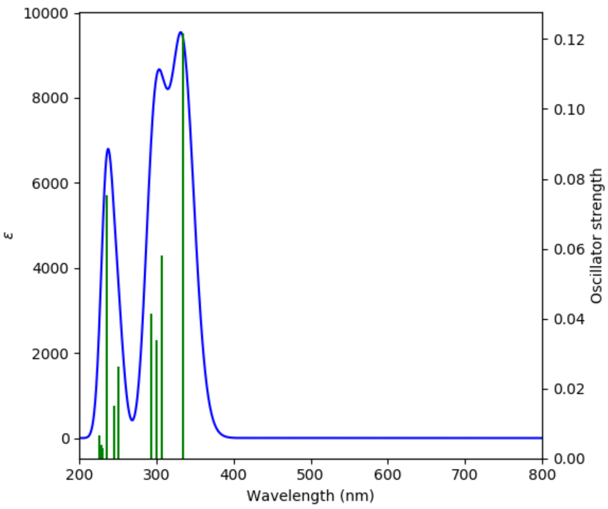

Orbital	Energy [eV]	Symmetry
L+4	-2.17	A
L+3	-2.37	A
L+2	-3.25	A
L+1	-3.36	A
LUMO	-4.36	A
HOMO	-8.23	A
H-1	-8.37	A
H-2	-8.46	A
H-3	-8.55	A
H-4	-9.58	A

Orbitals Relevant for $S_1 \leftarrow S_0$ Transition

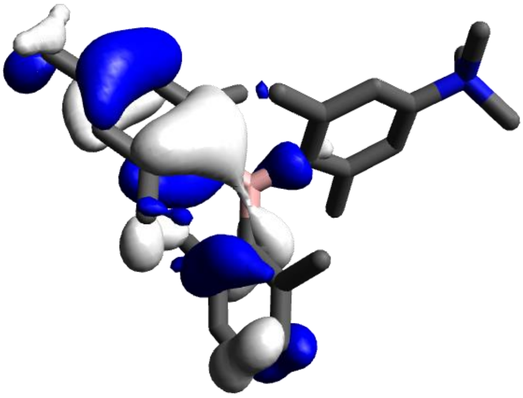

HOMO

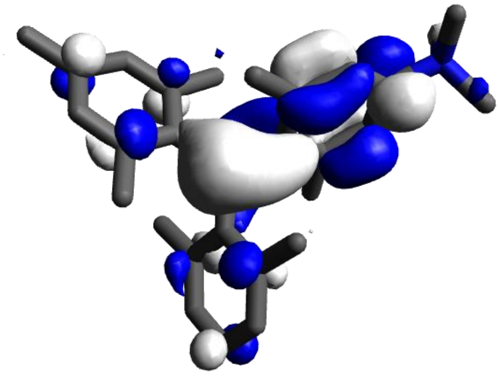

LUMO

Other Relevant Orbitals

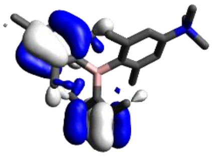

HOMO-1

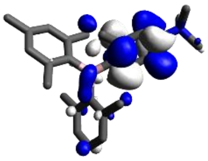

LUMO+1

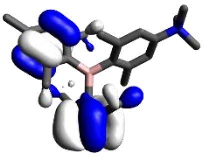

HOMO-2

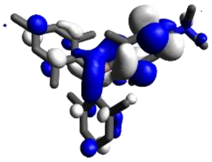

LUMO+2

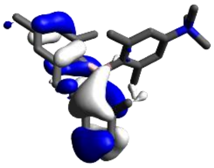

HOMO-3

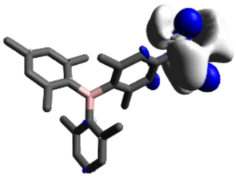

LUMO+3

Table S28: Lowest energy singlet electronic transitions of **5c** (TD-DFT CAM-B3LYP 6-31G(d,p), gas phase).

State	E [eV]	λ [nm]	*f*	Symmetry	Major Contributions	Λ
1	3.71	334	0.122	A	HOMO->LUMO (87%)	0.462
2	4.03	308	0.058	A	H-3->LUMO (54%), H-2->LUMO (17%), H-1->LUMO (14%)	0.473
3	4.14	300	0.034	A	H-3->LUMO (13%), H-1->LUMO (72%)	0.405
4	4.24	293	0.041	A	H-3->LUMO (17%), H-2->LUMO (68%)	0.397
5	4.96	250	0.026	A	H-4->LUMO (83%)	0.695
6	5.05	246	0.015	A	H-5->LUMO (62%), H-4->L+1 (14%)	0.565
7	5.25	236	0.075	A	HOMO->L+1 (31%), HOMO->L+2 (43%)	0.411
8	5.40	230	0.003	A	H-1->L+1 (10%), H-1->L+2 (21%)	0.481
9	5.41	229	0.004	A	H-2->LUMO (10%), H-2->L+2 (23%)	0.511
10	5.48	226	0.006	A	HOMO->L+1 (51%), HOMO->L+2 (32%)	0.364

5c

TD-DFT CAM-B3LYP 6-31G(d,p), gas phase, S_0

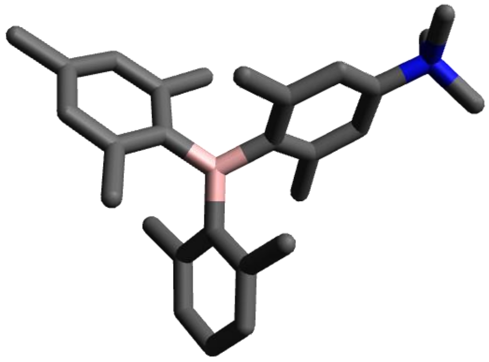

Point group: C_1

Total energy: -732910.16 kcal/mol

Dipole moment: 17.30 D

Imaginary frequencies: 0

Optimized x, y, z coordinates

C	-2.21131	4.49974	0.03195
C	-2.74042	3.57547	0.92721
C	-2.33727	2.23695	0.90467
C	-1.34575	1.81668	-0.0219
C	-0.80232	2.77117	-0.92032
C	-1.2533	4.09299	-0.89217
B	-0.84906	0.33344	-0.00098
C	0.7183	0.05813	-0.00078
C	-1.83234	-0.87505	0.01969
C	-1.63509	-1.98472	0.88342
C	-2.52696	-3.05701	0.86122

C	-3.61164	-3.09526	-0.01847
C	-3.79225	-2.01157	-0.88071
C	-2.94454	-0.90274	-0.86546
C	1.29516	-0.8019	-0.96032
C	2.67985	-1.02315	-0.97548
C	3.48479	-0.41299	-0.02464
C	2.9366	0.42731	0.93927
C	1.56242	0.67784	0.9516
C	-3.24742	0.22055	-1.83524
C	-0.49381	-2.04625	1.8777
C	0.46424	-1.51521	-2.00255
C	1.02563	1.61626	2.00715
C	-4.57355	-4.25412	-0.01594
C	-2.97781	1.29315	1.90112
C	0.24264	2.40492	-1.95357
N	4.9793	-0.63597	-0.00175
C	5.44689	-1.56307	-1.09128
C	5.68917	0.68605	-0.18821
C	5.37827	-1.24694	1.32252
H	-2.54638	5.5321	0.05203
H	-3.48491	3.89229	1.65162
H	-0.84925	4.80917	-1.60187
H	-2.37448	-3.88643	1.54736
H	-4.62307	-2.02841	-1.58137
H	3.0807	-1.67918	-1.73578
H	3.55415	0.90883	1.68963
H	-3.65134	1.09713	-1.32109
H	-2.36196	0.56055	-2.37925
H	-3.98492	-0.10431	-2.57289
H	-0.36707	-1.10869	2.42638
H	-0.67281	-2.83301	2.61443
H	0.46116	-2.26336	1.38814
H	-0.24818	-0.84429	-2.48796
H	1.09849	-1.94917	-2.77962
H	-0.12377	-2.31784	-1.5496
H	0.70835	2.5629	1.5625
H	0.15121	1.20119	2.51429
H	1.78298	1.82778	2.76609
H	-4.08904	-5.17645	0.31562
H	-5.41046	-4.06142	0.66606
H	-4.99716	-4.42427	-1.00938
H	-3.51952	1.85768	2.66373
H	-3.68823	0.61836	1.41513
H	-2.24935	0.65851	2.41346
H	-0.00121	1.47992	-2.48407
H	0.33171	3.19517	-2.70288
H	1.23007	2.26524	-1.50149
H	6.52725	-1.66679	-1.00044
H	4.9683	-2.53234	-0.96629
H	5.1946	-1.13414	-2.05901
H	6.76516	0.50946	-0.18151
H	5.41374	1.3575	0.6208
H	5.37322	1.10787	-1.14116
H	4.84188	-2.18783	1.43603
H	6.45576	-1.41408	1.32007
H	5.10548	-0.56852	2.12654

6c

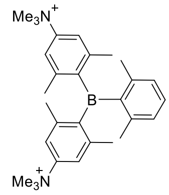

Functional used: TD-DFT CAM-B3LYP 6-31G(d,p), gas phase

Calculated Absorption Spectrum

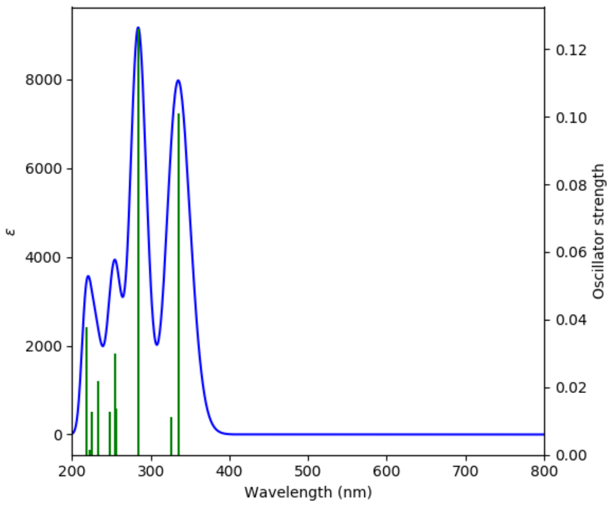

Orbital	Energy [eV]	Symmetry
L+4	-5.01	A
L+3	-5.44	A
L+2	-5.50	A
L+1	-5.83	A
LUMO	-6.90	A
HOMO	-10.62	A
H-1	-10.79	A
H-2	-11.69	A
H-3	-11.9	A
H-4	-11.92	A

Orbitals Relevant for $S_1 \leftarrow S_0$ Transition

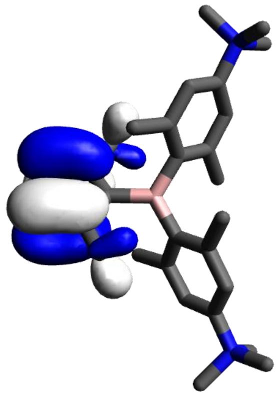

HOMO

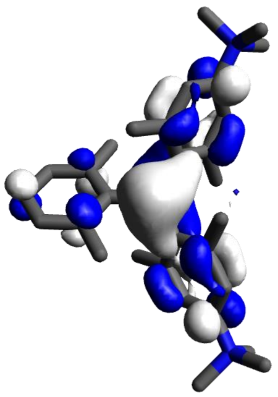

LUMO

Other Relevant Orbitals

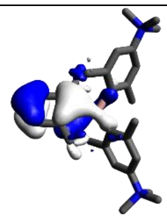

HOMO-1

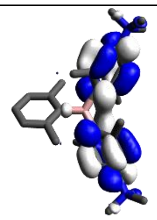

LUMO+1

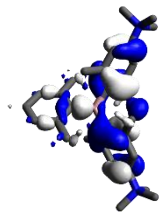

HOMO-2

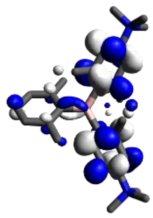

LUMO+2

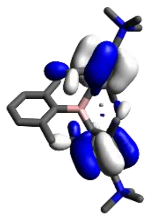

HOMO-3

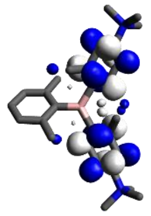

LUMO+3

Table S29: Lowest energy singlet electronic transitions of **6c** (TD-DFT CAM-B3LYP 6-31G(d,p), gas phase).

State	E [eV]	*λ* [nm]	*f*	Symmetry	Major Contributions	Λ
1	3.69	336	0.101	A	H-1->LUMO (92%)	0.472
2	3.80	326	0.011	A	HOMO->LUMO (92%)	0.286
3	4.36	284	0.126	A	H-2->LUMO (90%)	0.654
4	4.85	256	0.014	A	H-4->L+1 (11%),<br>H-3->LUMO (73%)	0.580
5	4.86	255	0.030	A	H-4->LUMO (70%),<br>H-3->L+1 (12%)	0.568
6	4.99	249	0.013	A	H-5->LUMO (87%)	0.692
7	5.32	233	0.022	A	H-1->L+1 (52%),<br>H-1->L+5 (10%),<br>HOMO->L+2 (10%),<br>HOMO->L+4 (15%)	0.342
8	5.50	225	0.013	A	H-1->L+1 (37%),<br>H-1->L+5 (11%),<br>HOMO->L+2 (19%),<br>HOMO->L+4 (16%)	0.349
9	5.56	223	0.001	A	HOMO->L+1 (94%)	0.136
10	5.69	218	0.038	A	H-1->L+2 (61%),<br>H-1->L+4 (12%)	0.468

6c

TD-DFT CAM-B3LYP 6-31G(d,p), gas phase, S_0

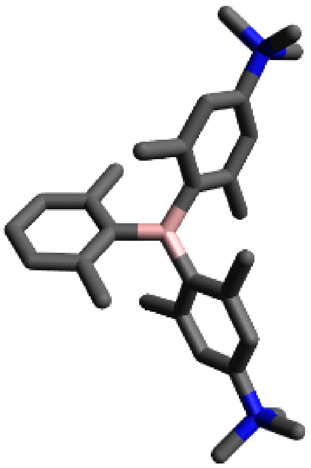

Point group: C_1

Total energy: -817135.15 kcal/mol

Dipole moment: 12.83 D

Imaginary frequencies: 0

Optimized x, y, z coordinates

C	-0.0006	5.15561	-0.00051
C	0.85111	4.45681	-0.8509
C	0.87988	3.06062	-0.85172
C	-0.00007	2.33455	0.0001
C	-0.88022	3.06068	0.85172
C	-0.85199	4.45685	0.85027
B	0.00002	0.78	0.00015
C	-1.37342	-0.0113	-0.01976
C	1.37345	-0.0113	0.02001

C	1.66414	-1.01341	-0.93109
C	2.91796	-1.64156	-0.94464
C	3.87197	-1.29814	0.0029
C	3.59259	-0.34099	0.97327
C	2.35871	0.31478	0.98573
C	-1.6643	-1.01309	0.93156
C	-2.91812	-1.64128	0.94504
C	-3.87187	-1.29822	-0.00286
C	-3.59226	-0.34139	-0.97351
C	-2.35844	0.31444	-0.98588
C	2.1402	1.37045	2.04563
C	0.66298	-1.42616	-1.98585
C	-0.66328	-1.42547	1.98661
C	-2.13968	1.36973	-2.04611
C	1.84003	2.38753	-1.81115
C	-1.83972	2.38755	1.81176
N	-5.23935	-1.93781	-0.01026
C	-5.41674	-2.95279	1.0897
C	-5.46425	-2.64664	-1.329
C	-6.29607	-0.8687	0.17903
N	5.23947	-1.93767	0.01013
C	5.41655	-2.95316	-1.08942
C	5.46485	-2.6459	1.32911
C	6.29607	-0.8686	-0.17999
H	-0.00084	6.24079	-0.00077
H	1.50539	5.00259	-1.52371
H	-1.5064	5.00269	1.5229
H	3.10678	-2.38438	-1.70703
H	4.32383	-0.06981	1.72627
H	-3.10709	-2.38381	1.70767
H	-4.32334	-0.07055	-1.7268
H	2.87855	1.27879	2.84543
H	2.22313	2.37382	1.61915
H	1.14869	1.30541	2.49861
H	0.28112	-0.56552	-2.54215
H	1.11026	-2.11036	-2.70995
H	-0.19925	-1.926	-1.53647
H	-0.28142	-0.56463	2.54258
H	-1.11063	-2.10939	2.71092
H	0.19897	-1.92551	1.53747
H	-2.87797	1.27792	-2.84594
H	-2.22239	2.37326	-1.61997
H	-1.14813	1.30438	-2.49898
H	2.19706	3.10533	-2.55267
H	2.71729	1.98718	-1.29304
H	1.3831	1.55765	-2.35704
H	-1.38175	1.55886	2.35864
H	-2.19765	3.10573	2.55247
H	-2.71643	1.98543	1.29412
H	-5.29183	-2.46323	2.05361
H	-6.42421	-3.35803	1.00903
H	-4.68669	-3.75011	0.96396
H	-5.40782	-1.92514	-2.13995
H	-6.45041	-3.11063	-1.3098
H	-4.68739	-3.40071	-1.4466
H	-7.27556	-1.34698	0.1881
H	-6.10469	-0.36596	1.1259
H	-6.23623	-0.1552	-0.63877
H	5.29125	-2.46405	-2.05352
H	6.42409	-3.35826	-1.00893
H	4.68662	-3.75047	-0.96305
H	5.40873	-1.92403	2.13975
H	6.451	-3.1099	1.30977
H	4.68802	-3.39991	1.44735
H	7.27559	-1.34682	-0.18914
H	6.10437	-0.36632	-1.12705
H	6.23643	-0.15471	0.63749

bis-Triarylboranes

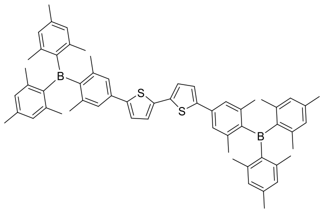

Neut0

Functional used: TD-DFT CAM-B3LYP 6-31G(d,p), gas phase

Calculated Absorption Spectrum

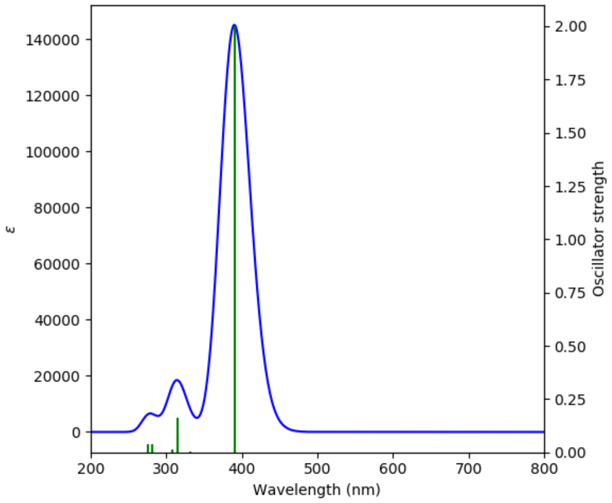

Orbital	Energy [eV]	Symmetry
L+4	-0.06	A
L+3	-0.49	B
L+2	-1.25	A
L+1	-1.69	B
LUMO	-1.96	A
HOMO	-4.97	B
H-1	-5.93	A
H-2	-5.97	A
H-3	-5.97	B
H-4	-6.16	B

Orbitals Relevant for $S_1 \leftarrow S_0$ Transition

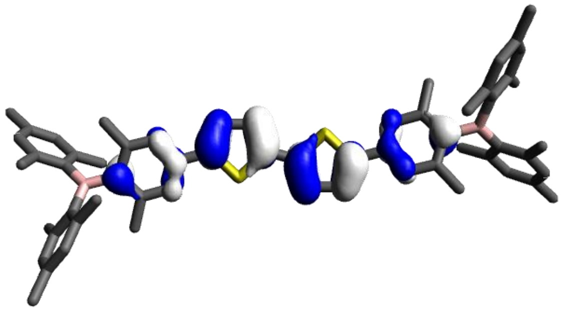

HOMO

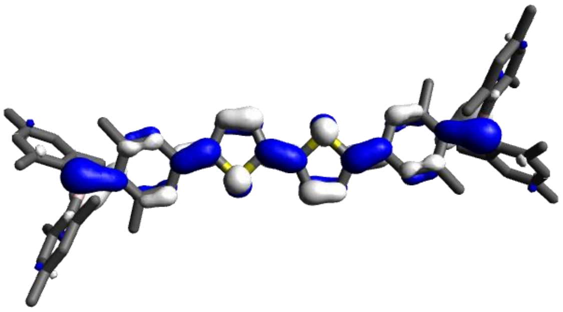

LUMO

Other Relevant Orbitals

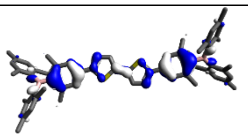

HOMO-1

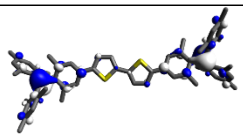

LUMO+1

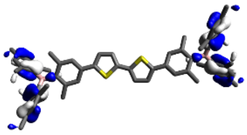

HOMO-2

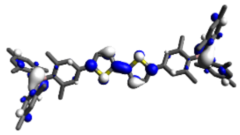

LUMO+2

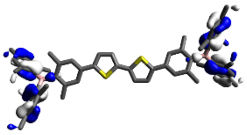

HOMO-3

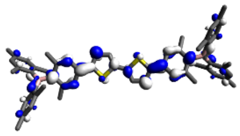

LUMO+3

Table S30: Lowest energy singlet electronic transitions of **Neut0** (TD-DFT CAM-B3LYP 6-31G(d,p), gas phase).

State	E [eV]	λ [nm]	*f*	Symmetry	Major Contributions	Λ
1	3.18	390	2.002	B	HOMO->LUMO (81%)	0.690
2	3.74	331	0.002	A	H-1->LUMO (26%), HOMO->L+1 (49%)	0.553
3	3.94	315	0.077	A	H-3->L+1 (44%), H-2->LUMO (34%), H-2->L+2 (15%)	0.485
4	3.94	315	0.165	B	H-3->LUMO (34%), H-3->L+2 (15%), H-2->L+1 (44%)	0.484
5	4.03	308	0.014	B	H-4->LUMO (13%), H-1->L+1 (27%), HOMO->L+2 (37%)	0.608
6	4.40	282	0.000	A	H-7->LUMO (19%), H-4->L+1 (32%), HOMO->L+1 (21%)	0.589
7	4.41	281	0.011	A	H-11->L+1 (17%), H-10->LUMO (28%), H-6->L+1 (16%), H-5->LUMO (16%)	0.452
8	4.41	281	0.036	B	H-11->LUMO (28%), H-10->L+1 (16%), H-6->LUMO (17%), H-5->L+1 (17%)	0.447
9	4.50	276	0.005	B	H-7->L+1 (21%), H-4->LUMO (16%), HOMO->L+2 (17%)	0.563
10	4.50	276	0.040	A	H-11->L+1 (14%), H-10->LUMO (18%), H-6->L+1 (24%), H-5->LUMO (15%), H-5->L+2 (12%)	0.444

Neut0

TD-DFT CAM-B3LYP 6-31G(d,p), gas phase, S_0

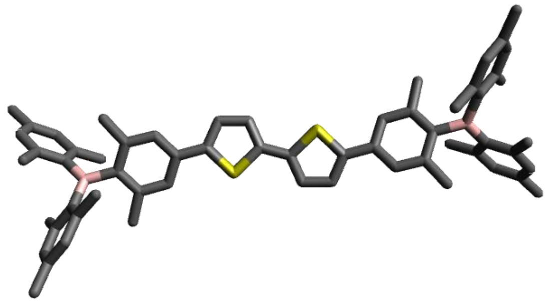

Point group: C_2

Total energy: -1989087.35 kcal/mol

Dipole moment: 0.15 D

Imaginary frequencies: 0

Optimized x, y, z coordinates

C	0.18473	0.69669	-0.31627
C	1.43729	1.26254	-0.44564
C	1.42634	2.67795	-0.40302
C	0.16965	3.22238	-0.24378
S	-1.02967	1.94681	-0.12052
H	2.33355	0.67133	-0.59358
H	2.31483	3.28563	-0.5233
C	-0.18473	-0.69669	-0.31627
C	-1.43729	-1.26254	-0.44564
S	1.02967	-1.94681	-0.12052
C	-1.42634	-2.67795	-0.40302
H	-2.33355	-0.67133	-0.59358
C	-0.16965	-3.22238	-0.24378
H	-2.31483	-3.28563	-0.5233
C	-0.21129	4.62953	-0.17033
C	-1.51208	5.05767	-0.46943
C	0.72784	5.60163	0.20549
C	-1.88093	6.3992	-0.38525
H	-2.25069	4.329	-0.79132
C	0.40001	6.95461	0.25679
H	1.73072	5.2914	0.48147
C	-0.9225	7.38177	-0.02955
C	-3.3149	6.75519	-0.71626
C	1.48873	7.92665	0.66009
B	-1.31485	8.90194	0.04653
H	-3.82204	5.90595	-1.1817
H	-3.87445	7.03516	0.18054
H	-3.38469	7.60224	-1.40444
H	1.15477	8.62857	1.42948
H	2.35489	7.38883	1.05465
H	1.82091	8.52999	-0.18922
C	-0.41888	9.9773	-0.66495
C	-2.60099	9.33999	0.83309
C	-0.00591	11.14735	0.02097
C	0.00591	9.80175	-2.00612
C	-3.5439	10.22328	0.24938
C	-2.8555	8.85757	2.14172
C	0.812	12.08118	-0.61917
C	-0.39985	11.42307	1.45702
C	0.79624	10.77429	-2.62254
C	-0.38393	8.58839	-2.82423
C	-4.69462	10.58305	0.95446
C	-3.36785	10.78524	-1.14589
C	-4.00137	9.26644	2.82739
C	-1.90941	7.91194	2.85175
C	1.21642	11.92063	-1.9456
H	1.13934	12.96039	-0.06887
H	-0.25659	10.55293	2.10395
H	0.19738	12.24251	1.86593
H	-1.4549	11.69963	1.53747
H	1.09793	10.63057	-3.65763
H	-0.16201	8.75374	-3.88194
H	0.15706	7.69417	-2.50167
H	-1.44927	8.35502	-2.74191
C	-4.93863	10.12531	2.2505
H	-5.41898	11.24177	0.48076
H	-3.09041	10.01711	-1.87341
H	-4.29621	11.24864	-1.49053
H	-2.57919	11.54228	-1.17734
H	-4.17099	8.89946	3.83715
H	-2.15693	7.851	3.9149
H	-1.96142	6.90209	2.43485
H	-0.86565	8.22893	2.77229
C	2.05681	12.9657	-2.63447
C	-6.1615	10.56655	3.01392
H	2.698	13.49504	-1.92373
H	2.69341	12.52201	-3.40553
H	1.42599	13.71684	-3.12595
H	-6.99493	10.78688	2.34064
H	-6.48761	9.80045	3.72344
H	-5.95832	11.47799	3.58984
C	0.21129	-4.62953	-0.17033
C	1.51208	-5.05767	-0.46943
C	-0.72784	-5.60163	0.20549
C	1.88093	-6.3992	-0.38525
H	2.25069	-4.329	-0.79132
C	-0.40001	-6.95461	0.25679
H	-1.73072	-5.2914	0.48147
C	0.9225	-7.38177	-0.02955
C	3.3149	-6.75519	-0.71626
C	-1.48873	-7.92665	0.66009
B	1.31485	-8.90194	0.04653
H	3.82204	-5.90595	-1.1817

H	3.87445	-7.03516	0.18054
H	3.38469	-7.60224	-1.40444
H	-1.15477	-8.62857	1.42948
H	-2.35489	-7.38883	1.05465
H	-1.82091	-8.52999	-0.18922
C	0.41888	-9.9773	-0.66495
C	2.60099	-9.33999	0.83309
C	0.00591	-11.14735	0.02097
C	-0.00591	-9.80175	-2.00612
C	3.5439	-10.22328	0.24938
C	2.8555	-8.85757	2.14172
C	-0.812	-12.08118	-0.61917
C	0.39985	-11.42307	1.45702
C	-0.79624	-10.77429	-2.62254
C	0.38393	-8.58839	-2.82423
C	4.69462	-10.58305	0.95446
C	3.36785	-10.78524	-1.14589
C	4.00137	-9.26644	2.82739
C	1.90941	-7.91194	2.85175
C	-1.21642	-11.92063	-1.9456
H	-1.13934	-12.96039	-0.06887
H	0.25659	-10.55293	2.10395
H	-0.19738	-12.24251	1.86593
H	1.4549	-11.69963	1.53747
H	-1.09793	-10.63057	-3.65763
H	0.16201	-8.75374	-3.88194
H	-0.15706	-7.69417	-2.50167
H	1.44927	-8.35502	-2.74191
C	4.93863	-10.12531	2.2505
H	5.41898	-11.24177	0.48076
H	3.09041	-10.01711	-1.87341
H	4.29621	-11.24864	-1.49053
H	2.57919	-11.54228	-1.17734
H	4.17099	-8.89946	3.83715
H	2.15693	-7.851	3.9149
H	1.96142	-6.90209	2.43485
H	0.86565	-8.22893	2.77229
C	-2.05681	-12.9657	-2.63447
C	6.1615	-10.56655	3.01392
H	-2.698	-13.49504	-1.92373
H	-2.69341	-12.52201	-3.40553
H	-1.42599	-13.71684	-3.12595
H	6.99493	-10.78688	2.34064
H	6.48761	-9.80045	3.72344
H	5.95832	-11.47799	3.58984

Neut1

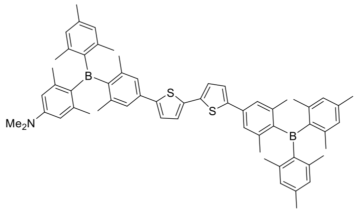

Functional used: TD-DFT CAM-B3LYP 6-31G(d,p), gas phase

Calculated Absorption Spectrum

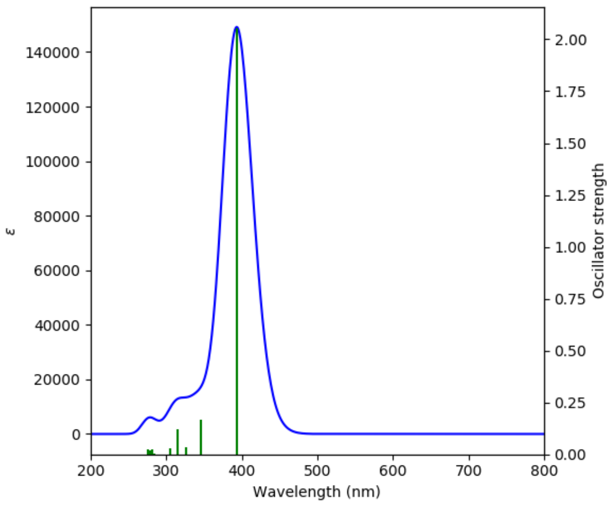

Orbital	Energy [eV]	Symmetry
L+4	-0.03	A
L+3	-0.42	A
L+2	-1.16	A
L+1	-1.55	A
LUMO	-1.88	A
HOMO	-4.87	A
H-1	-5.07	A
H-2	-5.84	A
H-3	-5.94	A
H-4	-5.96	A

Orbitals Relevant for $S_1 \leftarrow S_0$ Transition

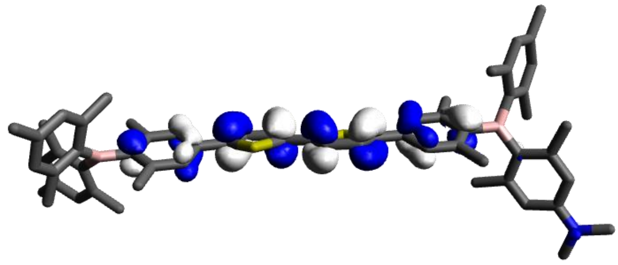

HOMO

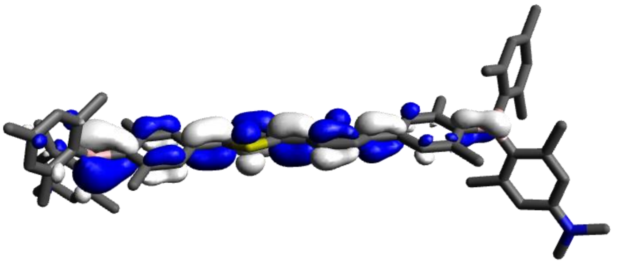

LUMO

Other Relevant Orbitals

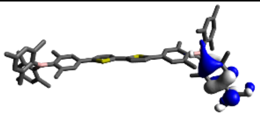

HOMO-1

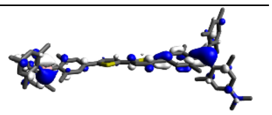

LUMO+1

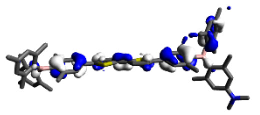

HOMO-2

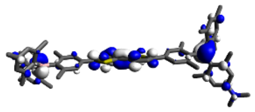

LUMO+2

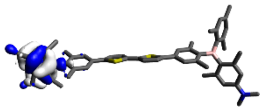

HOMO-3

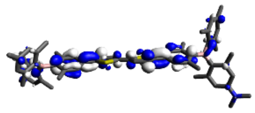

LUMO+3

Table S31: Lowest energy singlet electronic transitions of **Neut1** (TD-DFT CAM-B3LYP 6-31G(d,p), gas phase).

State	E [eV]	λ [nm]	*f*	Symmetry	Major Contributions	Λ
1	3.15	394	2.055	A	HOMO->LUMO (78%)	0.680
2	3.58	346	0.169	A	H-1->LUMO (20%), H-1->L+1 (34%), H-1->L+2 (17%), HOMO->L+1 (10%)	0.376
3	3.80	326	0.032	A	H-2->LUMO (22%), HOMO->L+1 (38%)	0.540
4	3.94	314	0.120	A	H-3->LUMO (46%), H-3->L+1 (35%), H-3->L+2 (11%)	0.357
5	4.05	306	0.027	A	H-2->L+1 (29%), HOMO->L+2 (32%)	0.592
6	4.36	284	0.002	A	H-6->LUMO (11%), H-4->L+1 (27%), HOMO->L+1 (16%)	0.530
7	4.41	281	0.023	A	H-11->LUMO (37%), H-11->L+1 (10%),H-7->LUMO (21%), H-7->L+1 (12%)	0.365
8	4.46	278	0.019	A	H-6->L+1 (21%), HOMO->L+2 (14%)	0.522
9	4.48	277	0.014	A	H-10->LUMO (11%), H-10->L+1 (12%), H-8->L+1 (12%), H-5->L+1 (10%)	0.401
10	4.50	276	0.026	A	H-11->LUMO (22%), H-7->LUMO (22%), H-7->L+1 (21%)	0.350

Neut1

TD-DFT CAM-B3LYP 6-31G(d,p), gas phase, S_0

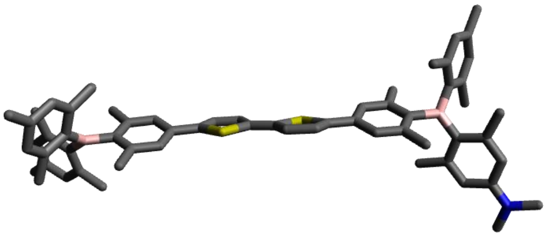

Point group: C_1

Total energy: -2048457.89 kcal/mol

Dipole moment: 2.68 D

Imaginary frequencies: 0

Optimized x, y, z coordinates

C	-2.74756	-0.3049	0.56457
C	-2.02546	-0.35756	1.73777
C	-0.62241	-0.32098	1.55019
C	-0.24306	-0.23868	0.2253
S	-1.65998	-0.19544	-0.80887
C	-4.19602	-0.32397	0.38359
C	1.08345	-0.18656	-0.33597
S	2.4982	-0.15499	0.70117
C	3.58755	-0.09367	-0.67449
C	2.86673	-0.10423	-1.84965
C	1.46423	-0.15632	-1.66284
C	5.03482	-0.04466	-0.49274
C	5.61322	0.3887	0.70849
C	6.99486	0.42118	0.88889
C	7.86195	0.05057	-0.17014
C	7.28094	-0.37038	-1.39447
C	5.89601	-0.43361	-1.52991
C	-4.78374	-0.73052	-0.82201
C	-6.16748	-0.76591	-0.98762
C	-7.02417	-0.36217	0.06616
C	-6.43286	0.06273	1.28369
C	-5.04697	0.06761	1.42774
B	-8.59154	-0.4015	-0.09059
B	9.42237	0.10088	0.0084
C	10.31335	0.79884	-1.08004
C	10.0865	-0.54724	1.27494
C	-9.45196	0.84494	0.27447
C	-9.26733	-1.72008	-0.6215
C	-8.95176	-2.98581	-0.06957
C	-9.55659	-4.14053	-0.57153
C	-10.45687	-4.09631	-1.63738
C	-10.75127	-2.84942	-2.19193
C	-10.19045	-1.66998	-1.69591
C	-10.66261	0.72058	1.00896
C	-11.41331	1.84129	1.35131
C	-11.02845	3.14013	0.96264
C	-9.84471	3.262	0.20768
C	-9.06188	2.15625	-0.11074
C	11.4628	0.1533	-1.60146
C	12.23052	0.77983	-2.58579
C	11.92336	2.05675	-3.05896
C	10.79858	2.69516	-2.53346
C	9.9873	2.08764	-1.5726
C	11.04519	0.16592	2.03795
C	11.60789	-0.41732	3.17547
C	11.28237	-1.7141	3.57693
C	10.34837	-2.41871	2.81535
C	9.73922	-1.85733	1.69095
C	11.88975	-1.22688	-1.14713
C	11.93946	-2.34432	4.77861
C	12.79456	2.73657	-4.08456
C	8.72832	-2.70083	0.94255
C	11.47632	1.57316	1.68109
C	8.78192	2.85955	-1.07798
C	-10.58878	-0.36702	-2.35803
C	-7.97807	-3.13914	1.07968
C	-11.1843	-0.61977	1.48654
C	-7.80651	2.42045	-0.9174
C	-6.70648	-1.23431	-2.32231
C	-7.26372	0.52086	2.46429
C	8.12266	-0.79807	-2.57829
C	7.51656	0.89187	2.23015
N	-11.77833	4.24788	1.3122
C	-11.44017	5.54602	0.75824
C	-13.09136	4.0655	1.90361
C	-11.11133	-5.35222	-2.15514
H	-2.4958	-0.45143	2.70885
H	0.09334	-0.3672	2.36289
H	3.33657	-0.04978	-2.82401
H	0.74977	-0.16623	-2.47782
H	4.97064	0.72313	1.51805
H	5.47452	-0.81062	-2.45634
H	-4.14806	-1.05292	-1.64211
H	-4.6156	0.4109	2.36304
H	-9.3134	-5.10141	-0.12331
H	-11.43961	-2.79342	-3.0324
H	-12.31486	1.6956	1.93301
H	-9.52408	4.23469	-0.14345
H	13.09457	0.2587	-2.99184
H	10.54231	3.69149	-2.88663
H	12.32294	0.15462	3.7625
H	10.08189	-3.43148	3.10922
H	11.05601	-1.93429	-1.12077
H	12.64816	-1.63303	-1.82185
H	12.31068	-1.20509	-0.13792

H	12.8563	-2.87488	4.49292
H	12.21803	-1.59194	5.52212
H	11.27944	-3.07258	5.25888
H	13.58791	3.32094	-3.60206
H	13.28074	2.00917	-4.7411
H	12.21611	3.42616	-4.70629
H	7.70713	-2.34415	1.10458
H	8.8949	-2.68949	-0.13833
H	8.77877	-3.74129	1.2745
H	10.62748	2.22656	1.46024
H	12.03461	2.02223	2.50694
H	12.11594	1.58413	0.79402
H	7.84844	2.43835	-1.46205
H	8.7015	2.85261	0.01277
H	8.83864	3.90307	-1.39918
H	-11.11551	-0.56149	-3.29644
H	-11.24705	0.22543	-1.71589
H	-9.72704	0.26735	-2.58402
H	-8.1712	-2.42356	1.88439
H	-8.04399	-4.14343	1.50701
H	-6.94579	-2.9761	0.75698
H	-11.58986	-1.21151	0.66142
H	-10.40259	-1.22804	1.94876
H	-11.97846	-0.47924	2.22486
H	-6.90549	2.3092	-0.30786
H	-7.70055	1.73031	-1.75835
H	-7.81725	3.43709	-1.31966
H	-5.90293	-1.29516	-3.0611
H	-7.17178	-2.2205	-2.24227
H	-7.47094	-0.55911	-2.71776
H	-8.09209	-0.1605	2.6775
H	-6.64561	0.58778	3.36378
H	-7.71023	1.50276	2.28389
H	8.91877	-1.49169	-2.29329
H	7.50247	-1.29473	-3.3292
H	8.61037	0.0582	-3.05226
H	6.71186	1.34356	2.81622
H	7.93495	0.06394	2.80911
H	8.3122	1.63533	2.12924
H	-10.417	5.83167	1.0253
H	-11.52463	5.57543	-0.33863
H	-12.11229	6.29772	1.17353
H	-13.77946	3.51494	1.24498
H	-13.02458	3.52066	2.85191
H	-13.52709	5.04267	2.11428
H	-11.35424	-5.267	-3.21836
H	-12.04848	-5.55736	-1.62268
H	-10.46459	-6.2243	-2.02098

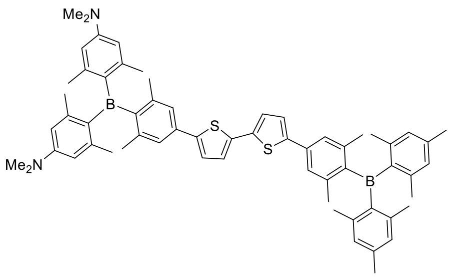

Neut2

Functional used: TD-DFT CAM-B3LYP 6-31G(d,p), gas phase

Calculated Absorption Spectrum

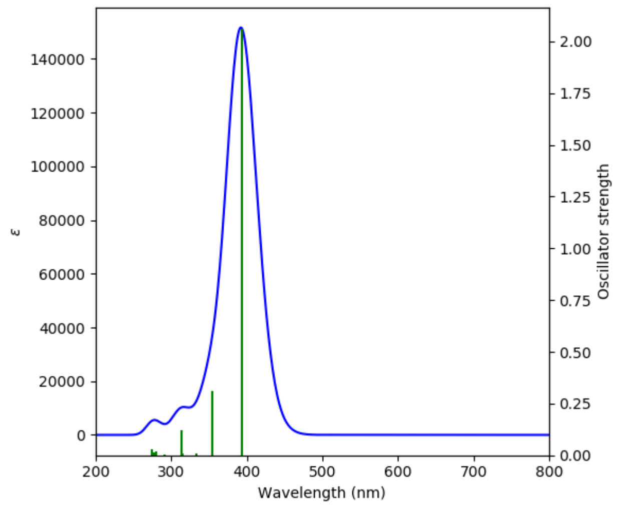

Orbital	Energy [eV]	Symmetry
L+4	-0.01	A
L+3	-0.35	A
L+2	-1.06	A
L+1	-1.46	A
LUMO	-1.83	A
HOMO	-4.82	A
H-1	-4.84	A
H-2	-5.05	A
H-3	-5.82	A
H-4	-5.93	A

Orbitals Relevant for $S_1 \leftarrow S_0$ Transition

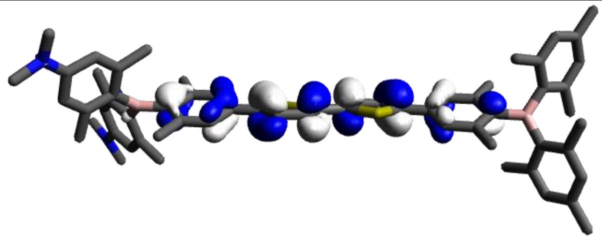

HOMO

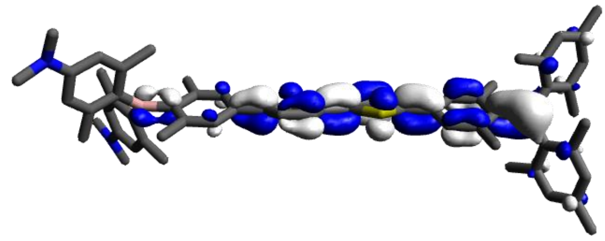

LUMO

Other Relevant Orbitals

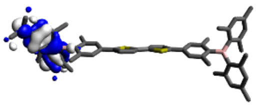

HOMO-1

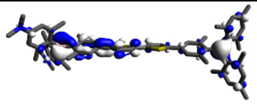

LUMO+1

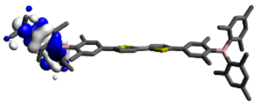

HOMO-2

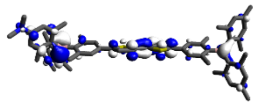

LUMO+2

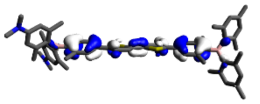

HOMO-3

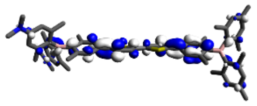

LUMO+3

Table S32: Lowest energy singlet electronic transitions of **Neut2** (TD-DFT CAM-B3LYP 6-31G(d,p), gas phase).

State	E [eV]	λ [nm]	*f*	Symmetry	Major Contributions	Λ
1	3.16	393	2.062	A	HOMO->LUMO (76%)	0.664
2	3.50	354	0.309	A	H-1->LUMO (16%), H-1->L+1 (43%), H-1->L+2 (31%)	0.307
3	3.72	333	0.001	A	H-3->LUMO (16%), H-2->L+1 (10%), H-2->L+2 (10%), HOMO->L+1 (34%)	0.527
4	3.93	315	0.001	A	H-2->L+1 (23%), HOMO->L+1 (12%), HOMO->L+2 (15%)	0.510
5	3.95	314	0.123	A	H-4->LUMO (52%), H-4->L+1 (34%)	0.367
6	4.25	292	0.004	A	H-3->L+1 (16%), H-2->L+1 (11%), HOMO->L+2 (24%)	0.515
7	4.42	281	0.021	A	H-11->LUMO (37%), H-8->LUMO (25%), H-8->L+1 (11%)	0.382
8	4.45	279	0.015	A	H-7->LUMO (15%), H-7->L+1 (11%), H-6->L+1 (11%), HOMO->L+2 (13%)	0.544
9	4.49	276	0.014	A	H-9->LUMO (21%), H-9->L+1 (29%), H-5->L+1 (12%)	0.336
10	4.51	275	0.027	A	H-11->LUMO (25%), H-8->LUMO (23%), H-8->L+1 (20%)	0.363

Neut2

TD-DFT CAM-B3LYP 6-31G(d,p), gas phase, S_0

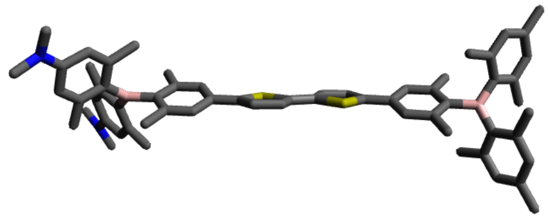

Point group: C_1

Total energy: -2107827.53 kcal/mol

Dipole moment: 2.36 D

Imaginary frequencies: 0

Optimized x, y, z coordinates

C	2.39264	-0.56318	0.36365
C	1.66064	-1.51957	1.03444
C	0.25884	-1.36155	0.91012
C	-0.10986	-0.27732	0.13932
S	1.31566	0.57023	-0.43452
C	3.84301	-0.42044	0.27389
C	-1.43174	0.18319	-0.204
S	-2.85713	-0.62997	0.41693
C	-3.93426	0.48154	-0.41228
C	-3.20188	1.40899	-1.12224
C	-1.80032	1.2441	-1.00727
C	-5.38391	0.35377	-0.30389
C	-5.98709	-0.3228	0.76544
C	-7.372	-0.42994	0.87957
C	-8.21587	0.13014	-0.113
C	-7.60886	0.80363	-1.20481
C	-6.22232	0.91366	-1.27959
C	4.45154	0.28029	-0.77657
C	5.83583	0.42772	-0.85423
C	6.67331	-0.16868	0.12014
C	6.06246	-0.8929	1.17444
C	4.6744	-0.99065	1.24944
B	8.24301	-0.02677	0.03708
B	-9.77829	0.00314	-0.00912
C	-10.60908	-0.48363	-1.24961
C	-10.50574	0.36235	1.33532
C	9.13674	-1.3061	0.12151
C	8.87839	1.39138	-0.12762
C	8.43747	2.50465	0.635
C	9.01204	3.76235	0.47159
C	10.03132	3.99475	-0.47253
C	10.44736	2.89803	-1.25274
C	9.90867	1.62595	-1.07651
C	10.28578	-1.34233	0.95539
C	11.06068	-2.49462	1.06139
C	10.77051	-3.65516	0.31735
C	9.63267	-3.61927	-0.51231
C	8.82649	-2.48755	-0.60173
C	-11.76181	0.22343	-1.67513
C	-12.47503	-0.21098	-2.7947
C	-12.10939	-1.35713	-3.50277
C	-10.9817	-2.05875	-3.07277
C	-10.2236	-1.63707	-1.97825
C	-11.46296	-0.51701	1.90133
C	-12.08269	-0.19177	3.10999
C	-11.8164	1.00562	3.77643
C	-10.8834	1.87544	3.20926
C	-10.2185	1.57017	2.01961
C	-12.24981	1.47126	-0.96923
C	-12.53422	1.36115	5.05368
C	-12.92264	-1.8382	-4.6776
C	-9.21306	2.57472	1.49665
C	-11.83146	-1.8349	1.25254
C	-9.00938	-2.46057	-1.60285
C	10.44917	0.52691	-1.96963
C	7.3472	2.38154	1.68006
C	10.70923	-0.15581	1.79823
C	7.6277	-2.57279	-1.5245
C	6.39874	1.21618	-2.0178
C	6.87825	-1.55498	2.26477
C	-8.42384	1.43199	-2.31575
C	-7.92167	-1.16938	2.08123
N	11.57266	-4.7823	0.39343
C	11.10947	-6.02524	-0.19604
C	12.61542	-4.84712	1.40053
N	10.6037	5.24755	-0.62255
C	11.50614	5.48927	-1.73296
C	9.98648	6.39248	0.02187
H	2.12393	-2.3331	1.57903
H	-0.46322	-2.03146	1.36274
H	-3.66357	2.20662	-1.69124
H	-1.07777	1.88856	-1.49443
H	-5.36212	-0.75498	1.54196
H	-5.78011	1.42201	-2.13072
H	3.83218	0.70703	-1.56072
H	4.22615	-1.50474	2.09406
H	8.65461	4.57326	1.09382
H	11.2033	3.03205	-2.01628
H	11.90398	-2.48312	1.74043
H	9.36427	-4.48695	-1.10191
H	-13.34229	0.35857	-3.12108
H	-10.68022	-2.95576	-3.60901
H	-12.79589	-0.89039	3.54165
H	-10.66231	2.81479	3.7111
H	-11.44176	2.18056	-0.7687
H	-12.99913	1.98531	-1.57727

H	-12.70245	1.23436	-0.00221
H	-13.46083	1.91026	4.84487
H	-12.80779	0.46654	5.62056
H	-11.91735	1.99756	5.69485
H	-13.70987	-2.53066	-4.35439
H	-13.41291	-1.00637	-5.19161
H	-12.30045	-2.37026	-5.40318
H	-8.18606	2.22123	1.62606
H	-9.3415	2.77582	0.42931
H	-9.31191	3.52551	2.02729
H	-10.95261	-2.40817	0.94369
H	-12.40453	-2.45506	1.94708
H	-12.43619	-1.68482	0.35376
H	-8.07887	-1.94708	-1.86139
H	-8.96275	-2.66916	-0.53026
H	-9.02189	-3.42008	-2.12678
H	11.0387	0.95347	-2.78605
H	11.08965	-0.16432	-1.41467
H	9.65334	-0.07868	-2.4109
H	7.49176	1.51245	2.32737
H	7.32364	3.2702	2.31675
H	6.36082	2.26846	1.22174
H	11.15583	0.63321	1.1867
H	9.86712	0.30214	2.32342
H	11.4457	-0.46211	2.54648
H	6.69128	-2.63438	-0.96309
H	7.54398	-1.69821	-2.1751
H	7.69712	-3.45739	-2.16351
H	5.62564	1.39568	-2.77001
H	6.78963	2.18413	-1.69171
H	7.2276	0.69646	-2.50716
H	7.64541	-0.88845	2.66966
H	6.23408	-1.86483	3.09235
H	7.40266	-2.43841	1.88995
H	-9.24841	2.03937	-1.9318
H	-7.79442	2.07872	-2.9328
H	-8.87091	0.67354	-2.96412
H	-7.12182	-1.70585	2.59839
H	-8.38852	-0.48467	2.79456
H	-8.68668	-1.90005	1.80388
H	11.86691	-6.79553	-0.046
H	10.16421	-6.37881	0.24319
H	10.95867	-5.91571	-1.27544
H	13.16185	-5.78449	1.28884
H	13.33226	-4.02899	1.27375
H	12.22206	-4.79627	2.42761
H	11.88255	6.51114	-1.67114
H	12.36848	4.81569	-1.6891
H	11.02308	5.35725	-2.71348
H	10.57161	7.28564	-0.20058
H	8.9522	6.56361	-0.31331
H	9.97152	6.26969	1.11023

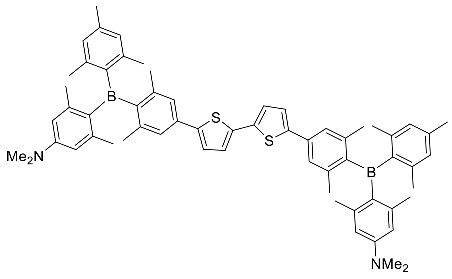

Neut(i)2

Functional used: TD-DFT CAM-B3LYP 6-31G(d,p), gas phase

Calculated Absorption Spectrum

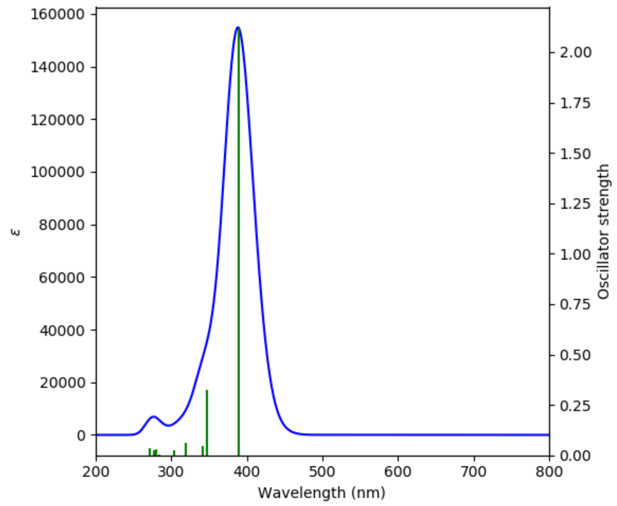

Orbital	Energy [eV]	Symmetry
L+4	0.16	A
L+3	-0.33	B
L+2	-1.06	A
L+1	-1.44	B
LUMO	-1.74	A
HOMO	-4.81	B
H-1	-5.04	A
H-2	-5.06	B
H-3	-5.78	A
H-4	-5.90	B

Orbitals Relevant for $S_1 \leftarrow S_0$ Transition

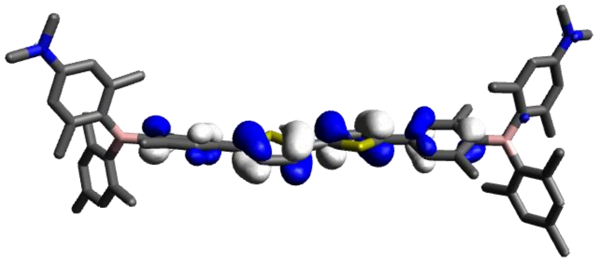

HOMO

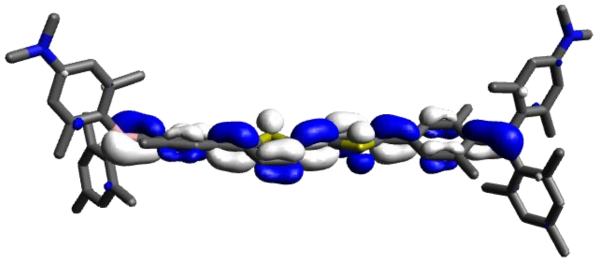

LUMO

Other Relevant Orbitals

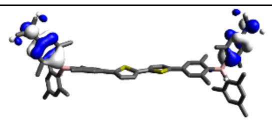

HOMO-1

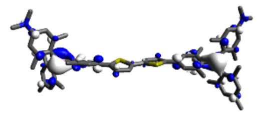

LUMO+1

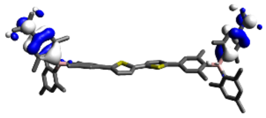

HOMO-2

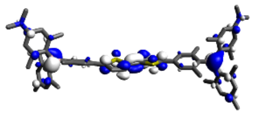

LUMO+2

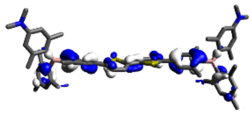

HOMO-3

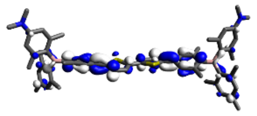

LUMO+3

Table S33: Lowest energy singlet electronic transitions of **Neut(i)2** (TD-DFT CAM-B3LYP 6-31G(d,p), gas phase).

State	E [eV]	λ [nm]	*f*	Sym-metry	Major Contributions	Λ
1	3.19	389	2.117	B	HOMO->LUMO (80%)	0.700
2	3.57	347	0.322	A	H-2->L+1 (30%),<br>H-1->LUMO (30%),<br>H-1->L+2 (14%),<br>HOMO->L+1 (13%)	0.425
3	3.63	342	0.044	B	H-2->LUMO (32%),<br>H-2->L+2 (12%),<br>H-1->L+1 (37%)	0.459
4	3.88	320	0.064	A	H-4->L+1 (11%),<br>H-3->LUMO (24%),<br>HOMO->L+1 (34%)	0.553
5	4.08	304	0.024	B	H-4->LUMO (15%),<br>H-3->L+1 (29%),<br>HOMO->L+2 (30%)	0.570
6	4.36	284	0.006	A	H-5->LUMO (21%),<br>H-5->L+2 (10%),<br>H-4->L+1 (31%),<br>HOMO->L+1 (16%)	0.530
7	4.43	280	0.030	B	H-5->L+1 (29%),<br>H-4->LUMO (16%),<br>HOMO->L+2 (21%)	0.545
8	4.47	277	0.003	A	H-11->L+1 (10%),<br>H-10->LUMO (20%),<br>H-9->LUMO (15%),<br>H-8->L+1 (11%)	0.456
9	4.48	277	0.026	B	H-11->LUMO (19%),<br>H-9->L+1 (10%),<br>H-8->LUMO (12%),<br>H-7->L+1 (10%)	0.452
10	4.56	272	0.037	A	H-11->L+1 (10%),<br>H-10->LUMO (12%),<br>H-7->LUMO (13%),<br>H-7->L+2 (13%),<br>H-6->L+1 (24%)	0.441

Neut(i)2

TD-DFT CAM-B3LYP 6-31G(d,p), gas phase, S_0

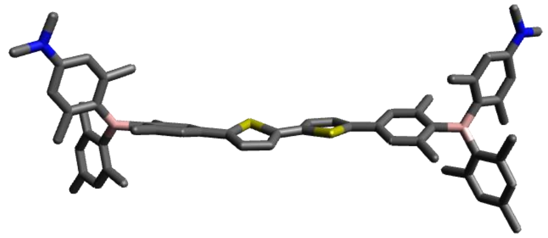

Point group: C_2

Total energy: -2107827.20 kcal/mol

Dipole moment: 3.83 D

Imaginary frequencies: 0

Optimized x, y, z coordinates

C	0.47212	4.61157	-0.45084
C	1.79357	4.96514	-0.75561
C	2.22704	6.28937	-0.71158
C	1.33503	7.3213	-0.32878
C	0.00052	6.96565	-0.00417
C	-0.41012	5.6362	-0.07692
B	1.79357	8.82787	-0.28892
C	1.52334	9.70597	0.96953
C	2.52951	9.42846	-1.54411
C	3.75535	10.12356	-1.39199
C	4.42428	10.61637	-2.51512
C	3.90484	10.47658	-3.80331
C	2.69171	9.80143	-3.94819
C	2.01015	9.26735	-2.8521
C	1.04708	11.04013	0.85375
C	0.78998	11.812	1.98299
C	1.03242	11.32758	3.28397
C	1.51058	10.00708	3.40047
C	1.73729	9.20825	2.28368
C	0.70849	8.53983	-3.11449
C	4.39695	10.34214	-0.03725
C	0.75439	11.67956	-0.48866
C	2.24919	7.80564	2.54296
C	4.61757	11.05975	-4.99731
C	3.66968	6.57178	-1.0728
C	-1.02357	7.99558	0.4254
N	0.8153	12.11555	4.39939
C	0.87153	11.51619	5.72034
C	0.16804	13.40585	4.2498
H	2.49409	4.19284	-1.06122
H	-1.43216	5.3848	0.18947
H	5.37565	11.12645	-2.38074
H	2.26803	9.68133	-4.94288
H	0.39026	12.80829	1.84196
H	1.71192	9.58879	4.37865
H	0.81683	7.45984	-2.97962
H	-0.08936	8.86113	-2.43841
H	0.36784	8.72021	-4.13772
H	4.40879	9.43329	0.57093
H	5.43071	10.67893	-0.1547
H	3.85891	11.09537	0.54562
H	0.19247	11.01606	-1.15093
H	0.16956	12.59403	-0.3567
H	1.67451	11.94009	-1.01881
H	1.478	7.05368	2.35461
H	3.09693	7.54861	1.90271
H	2.5732	7.70304	3.58214
H	5.69966	11.09221	-4.84012
H	4.42089	10.47868	-5.90313
H	4.28602	12.08741	-5.19188
H	4.24172	5.64155	-1.12473
H	3.74913	7.07551	-2.04001
H	4.15794	7.22166	-0.34072
H	-1.03411	8.86889	-0.23295
H	-2.02722	7.56167	0.42134
H	-0.81809	8.3704	1.43199
H	0.68161	12.28517	6.46984
H	0.13086	10.71357	5.85305
H	1.86331	11.09576	5.92049
H	0.11672	13.89475	5.22328
H	0.74165	14.05681	3.58123
H	-0.85394	13.32537	3.84938
C	0.01207	3.22781	-0.51878
C	-1.27265	2.75442	-0.68048
S	1.13687	1.88652	-0.38723
C	-1.3634	1.34146	-0.71912
H	-2.12462	3.41114	-0.80716
C	-0.14546	0.70597	-0.5846
H	-2.29091	0.80128	-0.86976
C	0.14546	-0.70597	-0.5846
C	1.3634	-1.34146	-0.71912
S	-1.13687	-1.88652	-0.38723
C	1.27265	-2.75442	-0.68048
H	2.29091	-0.80128	-0.86976
C	-0.01207	-3.22781	-0.51878
H	2.12462	-3.41114	-0.80716
C	-0.47212	-4.61157	-0.45084
C	-1.79357	-4.96514	-0.75561
C	0.41012	-5.6362	-0.07692
C	-2.22704	-6.28937	-0.71158

H	-2.49409	-4.19284	-1.06122
C	-0.00052	-6.96565	-0.00417
H	1.43216	-5.3848	0.18947
C	-1.33503	-7.3213	-0.32878
C	-3.66968	-6.57178	-1.0728
C	1.02357	-7.99558	0.4254
B	-1.79357	-8.82787	-0.28892
H	-4.24172	-5.64155	-1.12473
H	-3.74913	-7.07551	-2.04001
H	-4.15794	-7.22166	-0.34072
H	1.03411	-8.86889	-0.23295
H	2.02722	-7.56167	0.42134
H	0.81809	-8.3704	1.43199
C	-1.52334	-9.70597	0.96953
C	-2.52951	-9.42846	-1.54411
C	-1.04708	-11.04013	0.85375
C	-1.73729	-9.20825	2.28368
C	-3.75535	-10.12356	-1.39199
C	-2.01015	-9.26735	-2.8521
C	-0.78998	-11.812	1.98299
C	-0.75439	-11.67956	-0.48866
C	-1.51058	-10.00708	3.40047
C	-2.24919	-7.80564	2.54296
C	-4.42428	-10.61637	-2.51512
C	-4.39695	-10.34214	-0.03725
C	-2.69171	-9.80143	-3.94819
C	-0.70849	-8.53983	-3.11449
C	-1.03242	-11.32758	3.28397
H	-0.39026	-12.80829	1.84196
H	-0.19247	-11.01606	-1.15093
H	-0.16956	-12.59403	-0.3567
H	-1.67451	-11.94009	-1.01881
H	-1.71192	-9.58879	4.37865
H	-1.478	-7.05368	2.35461
H	-3.09693	-7.54861	1.90271
H	-2.5732	-7.70304	3.58214
C	-3.90484	-10.47658	-3.80331
H	-5.37565	-11.12645	-2.38074
H	-4.40879	-9.43329	0.57093
H	-5.43071	-10.67893	-0.1547
H	-3.85891	-11.09537	0.54562
H	-2.26803	-9.68133	-4.94288
H	-0.81683	-7.45984	-2.97962
H	0.08936	-8.86113	-2.43841
H	-0.36784	-8.72021	-4.13772
N	-0.8153	-12.11555	4.39939
C	-4.61757	-11.05975	-4.99731
C	-0.87153	-11.51619	5.72034
C	-0.16804	-13.40585	4.2498
H	-5.69966	-11.09221	-4.84012
H	-4.42089	-10.47868	-5.90313
H	-4.28602	-12.08741	-5.19188
H	-0.68161	-12.28517	6.46984
H	-0.13086	-10.71357	5.85305
H	-1.86331	-11.09576	5.92049
H	-0.11672	-13.89475	5.22328
H	-0.74165	-14.05681	3.58123
H	0.85394	-13.32537	3.84938

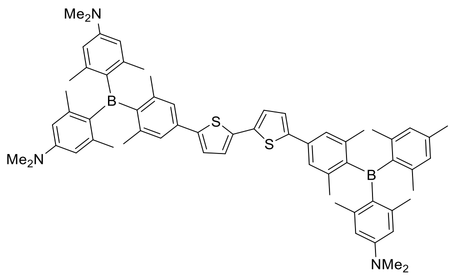

Neut3

Functional used: TD-DFT CAM-B3LYP 6-31G(d,p), gas phase

Calculated Absorption Spectrum

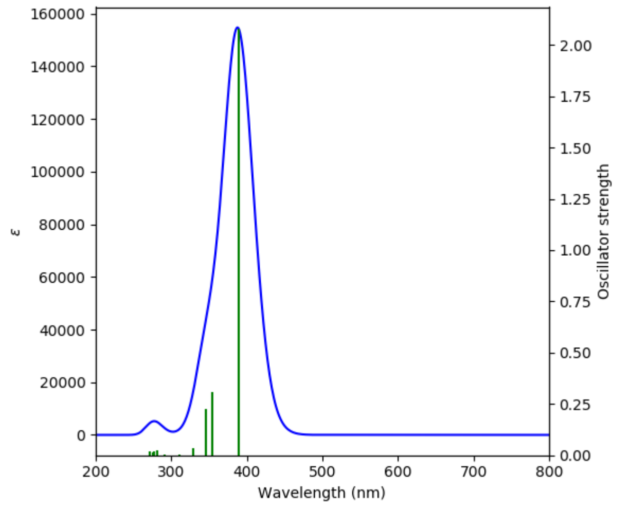

Orbital	Energy [eV]	Symmetry
L+4	0.18	A
L+3	-0.26	A
L+2	-0.97	A
L+1	-1.35	A
LUMO	-1.69	A
HOMO	-4.76	A
H-1	-4.82	A
H-2	-5.02	A
H-3	-5.04	A
H-4	-5.74	A

Orbitals Relevant for $S_1 \leftarrow S_0$ Transition

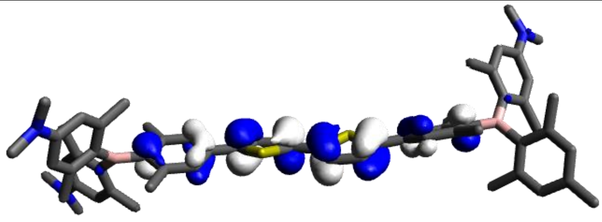

HOMO

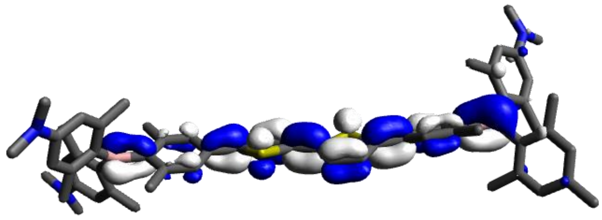

LUMO

Other Relevant Orbitals

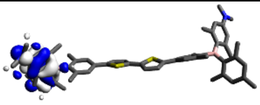

HOMO-1

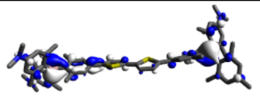

LUMO+1

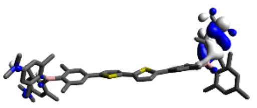

HOMO-2

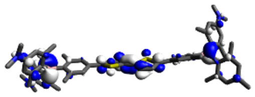

LUMO+2

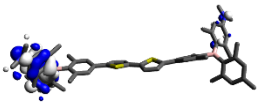

HOMO-3

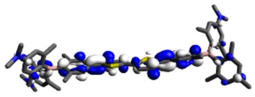

LUMO+3

Table S34: Lowest energy singlet electronic transitions of **Neut3** (TD-DFT CAM-B3LYP 6-31G(d,p), gas phase).

State	E [eV]	λ [nm]	*f*	Symmetry	Major Contributions	Λ
1	3.19	389	2.080	A	HOMO->LUMO (80%)	0.703
2	3.50	354	0.309	A	H-1->LUMO (23%), H-1->L+1 (43%), H-1->L+2 (24%)	0.295
3	3.58	346	0.226	A	H-2->LUMO (36%), H-2->L+1 (23%), H-2->L+2 (10%), HOMO->L+1 (10%)	0.403
4	3.77	328	0.034	A	H-4->LUMO (11%), H-3->LUMO (17%), H-2->L+1 (10%), HOMO->L+1 (21%)	0.501
5	3.99	311	0.004	A	H-4->LUMO (12%), H-3->L+1 (18%), HOMO->L+1 (15%), HOMO->L+2 (12%)	0.545
6	4.25	291	0.003	A	H-5->LUMO (11%), H-4->L+1 (16%), H-3->L+1 (11%), HOMO->L+1 (11%), HOMO->L+2 (18%)	0.515
7	4.41	281	0.026	A	H-5->LUMO (17%), H-5->L+1 (25%), HOMO->L+2 (19%)	0.507
8	4.46	278	0.018	A	H-11->LUMO (23%), H-9->LUMO (15%), H-7->LUMO (11%)	0.375
9	4.50	276	0.014	A	H-10->LUMO (27%), H-10->L+1 (22%), H-6->L+1 (12%)	0.364
10	4.58	271	0.019	A	H-6->L+1 (19%), H-6->L+2 (16%)	0.360

Neut3

TD-DFT CAM-B3LYP 6-31G(d,p), gas phase, S_0

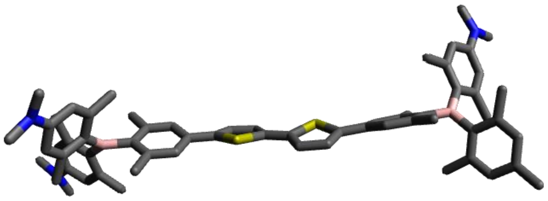

Point group: C_1

Total energy: -2167196.94 kcal/mol

Dipole moment: 2.47 D

Imaginary frequencies: 0

Optimized x, y, z coordinates

C	-3.52784	0.77948	-0.57225
C	-2.77529	1.87572	-0.9361
C	-1.37775	1.64539	-0.91665
C	-1.03448	0.36449	-0.53367
S	-2.47571	-0.56785	-0.17216
C	-4.98015	0.65564	-0.49106
C	0.27533	-0.22375	-0.40443
S	1.71128	0.78294	-0.35719
C	2.76605	-0.61338	-0.21757
C	2.01764	-1.77079	-0.18787
C	0.62166	-1.55518	-0.29369
C	4.21654	-0.46299	-0.1434
C	4.86657	0.67492	-0.64081
C	6.25058	0.82302	-0.55889
C	7.04702	-0.20539	0.00193
C	6.39465	-1.36462	0.49177
C	5.00627	-1.46863	0.43379
C	-5.61867	-0.59046	-0.55896
C	-7.00427	-0.7117	-0.46446
C	-7.81487	0.44413	-0.33565
C	-7.17382	1.70785	-0.28691
C	-5.78432	1.79531	-0.34152
B	-9.38373	0.31724	-0.2691
B	8.61683	-0.06481	0.07863
C	9.51836	-1.21588	-0.47404
C	9.24543	1.22144	0.70493
C	-10.25968	1.2069	-1.22765
C	-10.05333	-0.67323	0.73022
C	-9.60233	-0.79383	2.07276
C	-10.21674	-1.67139	2.9611
C	-11.28765	-2.4981	2.56674
C	-11.71644	-2.40135	1.22782
C	-11.13866	-1.50211	0.33638
C	-11.33445	1.97442	-0.71291
C	-12.08521	2.78714	-1.56574
C	-11.82992	2.85226	-2.93672
C	-10.78228	2.08122	-3.44297
C	-9.98981	1.28127	-2.61618
C	10.62369	-1.70225	0.27311
C	11.40266	-2.75574	-0.19917
C	11.16128	-3.35375	-1.4516
C	10.06745	-2.86932	-2.19526
C	9.25665	-1.8414	-1.72106
C	10.31738	1.89833	0.06502
C	10.85144	3.06737	0.60136
C	10.38816	3.60463	1.81844
C	9.32744	2.93176	2.45595
C	8.75784	1.78247	1.91436
C	10.99228	-1.13593	1.62992
N	10.95488	4.74311	2.36874
N	11.96853	-4.37124	-1.9349
C	7.61982	1.15176	2.69087
C	10.91003	1.41562	-1.24394
C	8.1082	-1.40999	-2.61038
C	-11.69682	-1.48663	-1.07251
C	-8.46048	0.03922	2.61955
C	-11.70489	1.96141	0.75575
C	-8.86969	0.49073	-3.25867
C	-7.59336	-2.10542	-0.5292
C	-7.95681	2.99482	-0.13758
C	7.16391	-2.50917	1.11721
C	6.85914	2.0953	-1.10987
C	-12.64189	3.7489	-3.837
N	-11.89515	-3.36342	3.45821
C	11.5449	-5.11868	-3.10486
C	12.95578	-4.97294	-1.0579
C	11.90339	5.51445	1.58688
C	10.29561	5.39602	3.48508
C	-12.87879	-4.31484	2.97504
C	-11.29167	-3.58973	4.75894
H	-3.22149	2.81298	-1.2455
H	-0.64071	2.38719	-1.20167
H	2.46731	-2.7544	-0.12866
H	-0.11126	-2.3534	-0.3168
H	4.28097	1.45588	-1.11803
H	4.52364	-2.34154	0.86245
H	-5.02144	-1.48628	-0.70445
H	-5.31284	2.76856	-0.24706
H	-9.85115	-1.70846	3.97959
H	-12.51375	-3.03897	0.86724
H	-12.89605	3.38128	-1.14982
H	-10.57394	2.10625	-4.51033
H	12.21008	-3.11512	0.42639
H	9.83753	-3.29761	-3.16285
H	11.64139	3.56661	0.05442
H	8.93261	3.30636	3.39206

H	10.12058	-1.00713	2.2768
H	11.69587	-1.7982	2.14219
H	11.45773	-0.1503	1.54157
H	6.65808	1.30913	2.19482
H	7.74218	0.07063	2.79793
H	7.55602	1.58041	3.69478
H	10.14173	1.13937	-1.9707
H	11.53193	2.19548	-1.69241
H	11.53161	0.52724	-1.10102
H	7.1443	-1.73535	-2.20935
H	8.0517	-0.32306	-2.71383
H	8.2179	-1.8342	-3.61223
H	-12.34368	-2.35282	-1.23661
H	-12.28501	-0.58459	-1.26225
H	-10.9102	-1.50881	-1.83109
H	-8.54745	1.09335	2.34402
H	-8.43365	-0.01877	3.71104
H	-7.49374	-0.30275	2.23995
H	-12.19298	1.02438	1.03911
H	-10.83214	2.06277	1.40694
H	-12.38936	2.78319	0.9839
H	-7.88893	0.89857	-2.99802
H	-8.86663	-0.55658	-2.94228
H	-8.96422	0.50703	-4.34786
H	-6.84032	-2.82339	-0.86535
H	-7.96029	-2.4299	0.44875
H	-8.44372	-2.16643	-1.21391
H	-8.706	2.9328	0.6572
H	-7.28796	3.82624	0.10073
H	-8.49581	3.24318	-1.05597
H	7.9055	-2.16166	1.84239
H	6.48393	-3.19124	1.63521
H	7.71257	-3.08065	0.36322
H	6.12176	2.65154	-1.69518
H	7.21911	2.74536	-0.30742
H	7.71882	1.89656	-1.75641
H	-13.66331	3.87026	-3.46435
H	-12.19809	4.75028	-3.90123
H	-12.69418	3.35146	-4.85485
H	10.58207	-5.63134	-2.95759
H	11.4465	-4.46185	-3.97584
H	12.30042	-5.86873	-3.34208
H	13.67159	-4.22376	-0.70358
H	12.50715	-5.45602	-0.17606
H	13.51531	-5.7263	-1.61393
H	12.76998	4.90427	1.31135
H	11.46597	5.9188	0.66092
H	12.26591	6.34942	2.18785
H	9.27751	5.73489	3.23992
H	10.22816	4.72488	4.34806
H	10.88262	6.26386	3.78825
H	-12.46113	-5.02552	2.24581
H	-13.27184	-4.88214	3.81944
H	-13.72102	-3.8004	2.49992
H	-11.23832	-2.65977	5.33579
H	-11.90797	-4.29199	5.32126
H	-10.27432	-4.00249	4.68785

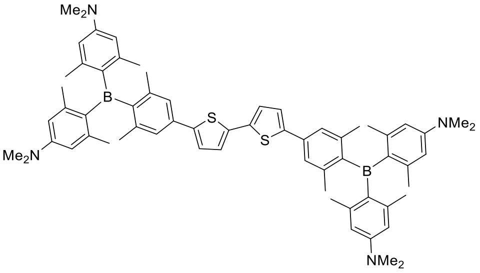

Neut4

Functional used: TD-DFT CAM-B3LYP 6-31G(d,p), gas phase

Calculated Absorption Spectrum

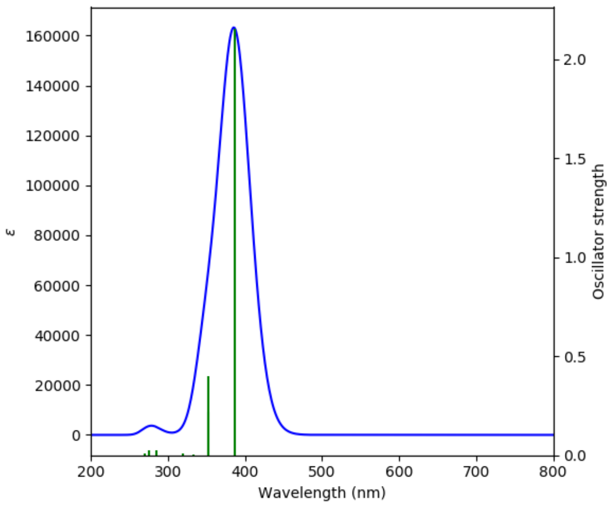

Orbital	Energy [eV]	Symmetry
L+4	0.34	A
L+3	-0.18	B
L+2	-0.89	A
L+1	-1.25	B
LUMO	-1.60	A
HOMO	-4.70	B
H-1	-4.80	A
H-2	-4.80	B
H-3	-4.99	A
H-4	-5.02	B

Orbitals Relevant for $S_1 \leftarrow S_0$ Transition

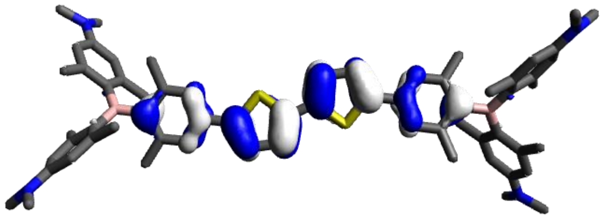

HOMO

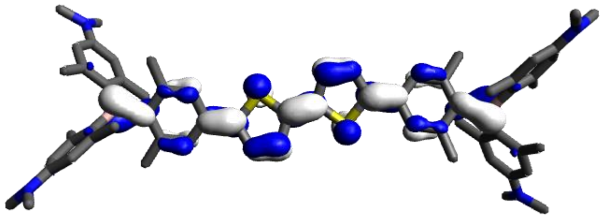

LUMO

Other Relevant Orbitals

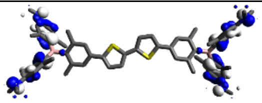

HOMO-1

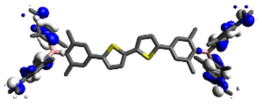

HOMO-2

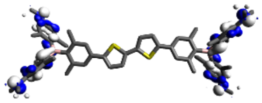

HOMO-3

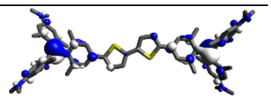

LUMO+1

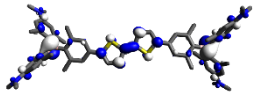

LUMO+2

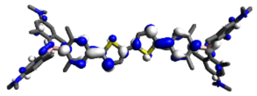

LUMO+3

Table S35: Lowest energy singlet electronic transitions of **Neut4** (TD-DFT CAM-B3LYP 6-31G(d,p), gas phase).

State	E [eV]	λ [nm]	*f*	Symmetry	Major Contributions	Λ
1	3.2	387	2.156	A	HOMO->LUMO (82%)	0.717
2	3.51	353	0.219	B	H-2->L+1 (42%), H-1->LUMO (31%), H-1->L+2 (18%)	0.416
3	3.51	353	0.400	A	H-2->LUMO (31%), H-2->L+2 (18%), H-1->L+1 (42%)	0.416
4	3.72	334	0.003	B	H-4->L+1 (15%), H-3->LUMO (21%), H-3->L+2 (10%), HOMO->L+1 (31%)	0.509
5	3.88	319	0.009	A	H-4->LUMO (28%), H-3->L+1 (34%), HOMO->L+2 (15%)	0.528
6	4.17	298	0.000	B	H-5->LUMO (12%), H-4->L+1 (27%), H-3->LUMO (15%), HOMO->L+1 (29%)	0.533
7	4.34	286	0.024	A	H-5->L+1 (23%), H-4->LUMO (11%), HOMO->L+2 (39%)	0.581
8	4.50	276	0.001	B	H-11->L+1 (18%), H-10->LUMO (33%), H-7->L+1 (10%), H-6->LUMO (11%)	0.463
9	4.50	276	0.026	A	H-11->LUMO (33%), H-10->L+1 (17%), H-7->LUMO (11%), H-6->L+1 (11%)	0.462
10	4.58	271	0.008	B	H-10->LUMO (10%), H-7->L+1 (20%), H-6->LUMO (10%), H-6->L+2 (13%)	0.456

Neut4

TD-DFT CAM-B3LYP 6-31G(d,p), gas phase, S_0

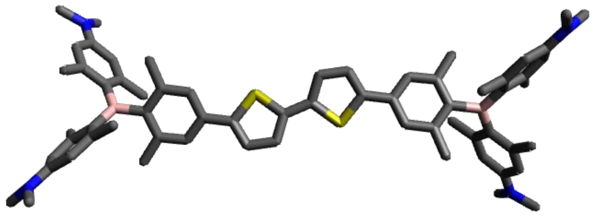

Point group: C_2

Total energy: -2226566.36 kcal/mol

Dipole moment: 0.6266 D

Imaginary frequencies: 0

Optimized x, y, z coordinates

C	0.28414	0.66252	-0.47675
C	1.6026	1.04216	-0.62621
C	1.79771	2.44444	-0.5762
C	0.63687	3.16442	-0.39011
S	-0.73213	2.07423	-0.24997
H	2.40027	0.3281	-0.79544
H	2.76256	2.91815	-0.71006
C	-0.28414	-0.66252	-0.47675
C	-1.6026	-1.04216	-0.62621
S	0.73213	-2.07423	-0.24997
C	-1.79771	-2.44444	-0.5762
H	-2.40027	-0.3281	-0.79544
C	-0.63687	-3.16442	-0.39011
H	-2.76256	-2.91815	-0.71006
C	0.46442	4.61135	-0.29731
C	-0.76731	5.22553	-0.56273
C	1.54133	5.43412	0.0655
C	-0.93714	6.60517	-0.45482
H	-1.60971	4.61455	-0.87521
C	1.41011	6.81941	0.14073
H	2.4959	4.98003	0.31296
C	0.1573	7.43468	-0.10739
C	-2.3106	7.17263	-0.74525
C	2.63348	7.62536	0.52391
B	-0.01639	8.99832	0.01149
H	-2.94503	6.41864	-1.21931
H	-2.80557	7.50835	0.17038
H	-2.26766	8.04006	-1.41025
H	2.41871	8.35556	1.3096
H	3.4283	6.96783	0.88693
H	3.02093	8.19229	-0.32732
C	0.97117	9.94494	-0.7445
C	-1.17529	9.57459	0.8878
C	1.55397	11.06504	-0.09472
C	1.34882	9.71212	-2.09282
C	-2.00316	10.62474	0.40962
C	-1.46163	9.06471	2.18108
C	2.46468	11.88565	-0.75567
C	1.25199	11.40647	1.35086
C	2.23304	10.56348	-2.75027
C	0.79411	8.55076	-2.89234
C	-3.05921	11.11235	1.17527
C	-1.80605	11.24978	-0.95722
C	-2.49906	9.58835	2.94837
C	-0.63687	7.95946	2.80877
C	2.81337	11.67226	-2.10379
H	2.91012	12.70382	-0.20376
H	1.2861	10.52815	2.00066
H	1.97508	12.13376	1.73076
H	0.25131	11.8331	1.46298
H	2.47134	10.35374	-3.78553
H	1.22709	7.59895	-2.5724
H	-0.28911	8.4529	-2.78141
H	1.00955	8.67933	-3.95665
C	-3.33782	10.60988	2.46153
H	-3.67466	11.89854	0.75609
H	-1.65541	10.49981	-1.73803
H	-2.67799	11.84992	-1.23211
H	-0.92639	11.89908	-0.98097
H	-2.65085	9.19003	3.9437
H	-0.8503	6.98942	2.35133
H	0.43716	8.12887	2.69422
H	-0.84938	7.88184	3.87861
N	3.68549	12.52001	-2.76821
N	-4.39684	11.09524	3.21193
C	4.20404	12.12953	-4.06654
C	4.41155	13.52566	-2.01499
C	-4.53045	10.69024	4.59899
C	-5.07984	12.30088	2.78017
H	4.86402	12.91445	-4.43802
H	4.7714	11.18691	-4.03312
H	3.3931	12.00856	-4.79278
H	5.01961	14.11605	-2.70165
H	3.72107	14.21069	-1.51185
H	5.07497	13.09203	-1.25069
H	-3.67319	10.996	5.21855
H	-5.43265	11.13899	5.01664
H	-4.63359	9.60306	4.68009
H	-5.53519	12.16251	1.79355
H	-5.88169	12.5283	3.48369
H	-4.41056	13.17296	2.72414
C	-0.46442	-4.61135	-0.29731
C	0.76731	-5.22553	-0.56273
C	-1.54133	-5.43412	0.0655
C	0.93714	-6.60517	-0.45482
H	1.60971	-4.61455	-0.87521

C	-1.41011	-6.81941	0.14073
H	-2.4959	-4.98003	0.31296
C	-0.1573	-7.43468	-0.10739
C	2.3106	-7.17263	-0.74525
C	-2.63348	-7.62536	0.52391
B	0.01639	-8.99832	0.01149
H	2.94503	-6.41864	-1.21931
H	2.80557	-7.50835	0.17038
H	2.26766	-8.04006	-1.41025
H	-2.41871	-8.35556	1.3096
H	-3.4283	-6.96783	0.88693
H	-3.02093	-8.19229	-0.32732
C	-0.97117	-9.94494	-0.7445
C	1.17529	-9.57459	0.8878
C	-1.55397	-11.06504	-0.09472
C	-1.34882	-9.71212	-2.09282
C	2.00316	-10.62474	0.40962
C	1.46163	-9.06471	2.18108
C	-2.46468	-11.88565	-0.75567
C	-1.25199	-11.40647	1.35086
C	-2.23304	-10.56348	-2.75027
C	-0.79411	-8.55076	-2.89234
C	3.05921	-11.11235	1.17527
C	1.80605	-11.24978	-0.95722
C	2.49906	-9.58835	2.94837
C	0.63687	-7.95946	2.80877
C	-2.81337	-11.67226	-2.10379
H	-2.91012	-12.70382	-0.20376
H	-1.2861	-10.52815	2.00066
H	-1.97508	-12.13376	1.73076
H	-0.25131	-11.8331	1.46298
H	-2.47134	-10.35374	-3.78553
H	-1.22709	-7.59895	-2.5724
H	0.28911	-8.4529	-2.78141
H	-1.00955	-8.67933	-3.95665
C	3.33782	-10.60988	2.46153
H	3.67466	-11.89854	0.75609
H	1.65541	-10.49981	-1.73803
H	2.67799	-11.84992	-1.23211
H	0.92639	-11.89908	-0.98097
H	2.65085	-9.19003	3.9437
H	0.8503	-6.98942	2.35133
H	-0.43716	-8.12887	2.69422
H	0.84938	-7.88184	3.87861
N	-3.68549	-12.52001	-2.76821
N	4.39684	-11.09524	3.21193
C	-4.20404	-12.12953	-4.06654
C	-4.41155	-13.52566	-2.01499
C	4.53045	-10.69024	4.59899
C	5.07984	-12.30088	2.78017
H	-4.86402	-12.91445	-4.43802
H	-4.7714	-11.18691	-4.03312
H	-3.3931	-12.00856	-4.79278
H	-5.01961	-14.11605	-2.70165
H	-3.72107	-14.21069	-1.51185
H	-5.07497	-13.09203	-1.25069
H	3.67319	-10.996	5.21855
H	5.43265	-11.13899	5.01664
H	4.63359	-9.60306	4.68009
H	5.53519	-12.16251	1.79355
H	5.88169	-12.5283	3.48369
H	4.41056	-13.17296	2.72414

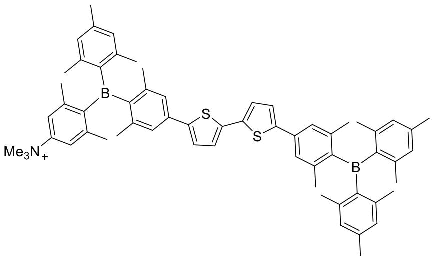

Cat[1+]

Functional used: TD-DFT CAM-B3LYP 6-31G(d,p), gas phase

Calculated Absorption Spectrum

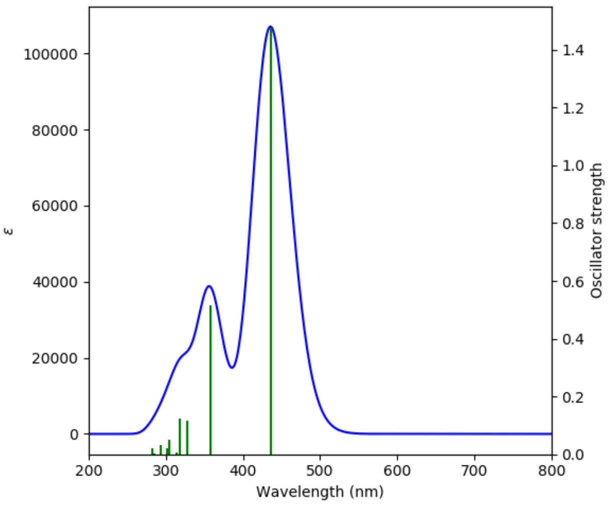

Orbital	Energy [eV]	Symmetry
L+4	-2.49	A
L+3	-2.87	A
L+2	-3.22	A
L+1	-3.36	A
LUMO	-4.24	A
HOMO	-6.33	A
H-1	-6.78	A
H-2	-6.98	A
H-3	-7.02	A
H-4	-7.10	A

Orbitals Relevant for $S_1 \leftarrow S_0$ Transition

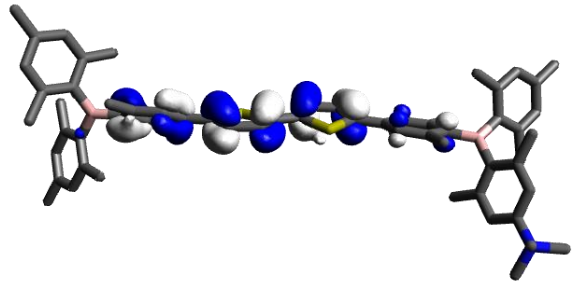

HOMO

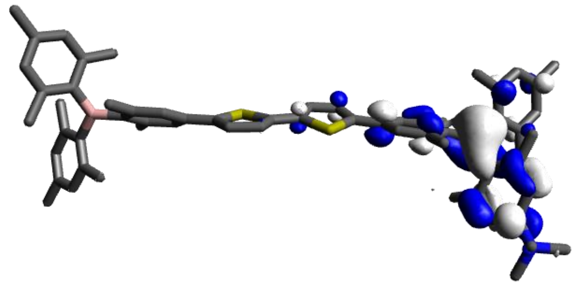

LUMO

Other Relevant Orbitals

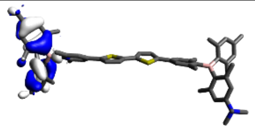

HOMO-1

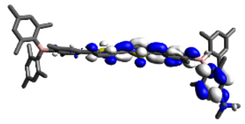

LUMO+1

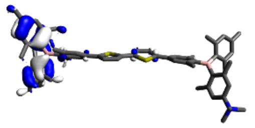

HOMO-2

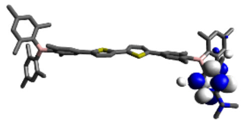

LUMO+2

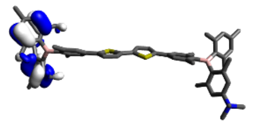

HOMO-3

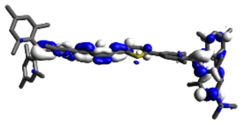

LUMO+3

Table S36: Lowest energy singlet electronic transitions of **Cat^{1+}** (TD-DFT CAM-B3LYP 6-31G(d,p), gas phase).

State	E [eV]	λ [nm]	*f*	Symmetry	Major Contributions	Λ
1	2.85	436	1.477	A	HOMO->LUMO (60%), HOMO->L+1 (18%)	0.355
2	3.47	358	0.515	A	HOMO->LUMO (10%), HOMO->L+1 (42%), HOMO->L+3 (22%)	0.518
3	3.78	328	0.115	A	H-7->LUMO (62%), HOMO->LUMO (10%)	0.425
4	3.89	319	0.123	A	H-1->L+3 (27%), H-1-> L+4 (49%)	0.345
5	3.95	314	0.005	A	H-2->L+3 (10%), H-2-> L+4 (15%), HOMO-> L+4 (27%)	0.499
6	4.07	305	0.051	A	H-10->LUMO (14%), H-9->LUMO (20%), H-5->LUMO (20%), HOMO->LUMO (14%)	0.377
7	4.11	302	0.023	A	H-9->LUMO (53%)	0.424
8	4.21	294	0.033	A	H-12->LUMO (16%), H-11->LUMO (45%)	0.423
9	4.36	285	0.003	A	H-2->L+3 (13%), H-2->L+4 (15%), HOMO->L+3 (29%)	0.541
10	4.39	283	0.021	A	H-6->L+3 (13%), H-3->L+3 (17%), H-3->L+4 (27%)	0.261

Cat^{1+}

TD-DFT CAM-B3LYP 6-31G(d,p), gas phase, S_0

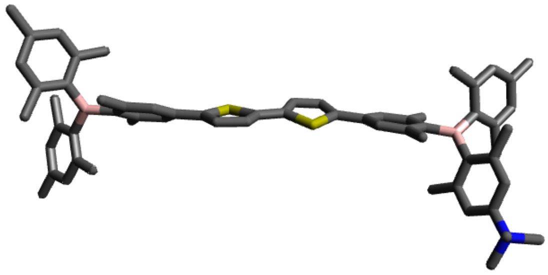

Point group: C_1

Total energy: -2073359.69 kcal/mol

Dipole moment: 47.70 D

Imaginary frequencies: 0

Optimized x, y, z coordinates

C	-2.54897	-3.58563	-1.71223
C	-2.38839	-2.33078	-1.15741
C	-1.04564	-1.89622	-1.09767
C	-0.14404	-2.81837	-1.59872
S	-0.98431	-4.25366	-2.14685
C	-3.77867	-4.32568	-1.94545
C	1.29093	-2.70074	-1.67331
S	2.18495	-1.5498	-0.69987
C	3.71762	-2.07182	-1.37194
C	3.51392	-3.0809	-2.29151
C	2.15505	-3.43283	-2.46478
C	4.97569	-1.46092	-0.95259
C	5.10063	-0.80425	0.27899
C	6.30707	-0.23803	0.68756
C	7.44278	-0.29376	-0.15847
C	7.31645	-0.94498	-1.41195
C	6.10348	-1.52135	-1.78377
C	-3.82514	-5.42352	-2.82046
C	-4.99342	-6.14855	-3.02888
C	-6.20293	-5.76832	-2.38553
C	-6.16768	-4.63799	-1.51892
C	-4.97157	-3.963	-1.2973
B	-7.52048	-6.57556	-2.56544
B	8.80979	0.35412	0.28136
C	9.56043	1.32385	-0.69647
C	9.4077	0.02478	1.69324
C	-8.9053	-5.88913	-2.78928
C	-7.45768	-8.16577	-2.51891
C	-6.85405	-8.82764	-1.42786
C	-6.81	-10.2285	-1.3816
C	-7.34237	-10.9683	-2.4273
C	-7.9347	-10.33783	-3.5172
C	-8.00809	-8.94378	-3.56541
C	-10.06701	-6.29046	-2.08082
C	-11.29152	-5.67055	-2.33267
C	-11.42544	-4.66974	-3.29774
C	-10.28424	-4.29053	-4.00813
C	-9.03412	-4.86106	-3.76179
C	10.94643	1.16231	-0.95201
C	11.59537	2.01501	-1.84739
C	10.92788	3.06248	-2.48521
C	9.56697	3.22648	-2.22123
C	8.87523	2.3736	-1.35848
C	9.87074	1.0621	2.54244
C	10.37448	0.75574	3.80847
C	10.47529	-0.55999	4.26425
C	10.03049	-1.57953	3.42045
C	9.48933	-1.31123	2.16156
C	11.76666	0.06632	-0.30424
C	11.07201	-0.8725	5.61274
C	11.66071	4.00256	-3.40793
C	9.0258	-2.48843	1.32926
C	9.81924	2.52153	2.14067
C	7.39716	2.63042	-1.15085
C	-8.68064	-8.31228	-4.76199
C	-6.23296	-8.07289	-0.27447
C	-10.03106	-7.36456	-1.01358
C	-7.86252	-4.35928	-4.58031
C	-4.92196	-7.31024	-3.99875
C	-7.38875	-4.14614	-0.76957
C	8.47323	-1.04818	-2.38292
C	6.34793	0.4321	2.0443
C	-12.75161	-3.99915	-3.54424
N	-7.2982	-12.47904	-2.42177
C	-6.63385	-13.0378	-1.19249
C	-8.70687	-13.02552	-2.47058
C	-6.5205	-12.96465	-3.62391
H	-3.22298	-1.71924	-0.83661
H	-0.74231	-0.92414	-0.72946
H	4.32851	-3.56795	-2.81281
H	1.81356	-4.19255	-3.15748
H	4.24628	-0.75854	0.94889
H	6.02479	-2.00257	-2.75362
H	-2.92927	-5.70445	-3.36613
H	-4.95976	-3.14114	-0.58947
H	-6.34711	-10.69376	-0.52226
H	-8.35365	-10.90436	-4.34179
H	-12.16586	-5.97457	-1.76233
H	-10.37012	-3.52232	-4.77248
H	12.65263	1.86056	-2.04998
H	9.02839	4.03782	-2.70559
H	10.70255	1.566	4.45548
H	10.10282	-2.61197	3.75464
H	11.27248	-0.9083	-0.34905
H	12.73511	-0.02968	-0.80206
H	11.94937	0.27361	0.7539
H	12.15517	-1.02802	5.53585
H	10.91088	-0.05443	6.32057
H	10.64286	-1.78418	6.03835
H	12.11161	4.83082	-2.84747
H	12.46999	3.49202	-3.93791
H	10.98725	4.44015	-4.15033
H	7.93443	-2.54067	1.27673
H	9.38756	-2.43767	0.29834
H	9.38699	-3.42606	1.75978
H	8.85398	2.80384	1.71084
H	9.99634	3.16194	3.00882
H	10.57563	2.75753	1.38681
H	6.78251	1.87287	-1.6457
H	7.11698	2.61972	-0.0936
H	7.11922	3.60695	-1.55641

H	-8.85808	-9.0506	-5.54807
H	-9.63919	-7.86838	-4.48138
H	-8.07795	-7.50669	-5.18861
H	-6.87426	-7.26095	0.07552
H	-6.0413	-8.73823	0.57129
H	-5.28607	-7.6145	-0.57245
H	-9.94233	-8.36603	-1.4472
H	-9.19104	-7.23812	-0.32446
H	-10.94711	-7.34265	-0.4183
H	-7.19128	-3.7367	-3.98186
H	-7.25118	-5.1702	-4.98579
H	-8.21657	-3.75749	-5.42074
H	-4.00319	-7.25856	-4.58775
H	-4.92742	-8.27367	-3.47903
H	-5.76066	-7.31856	-4.70026
H	-7.93826	-4.95601	-0.28211
H	-7.09711	-3.43	0.00226
H	-8.0975	-3.65023	-1.4382
H	9.39263	-1.38502	-1.89581
H	8.24175	-1.75766	-3.18166
H	8.69902	-0.08117	-2.84034
H	5.33686	0.55581	2.44139
H	6.92689	-0.15493	2.76233
H	6.81299	1.42111	2.00354
H	-13.58694	-4.65765	-3.29124
H	-12.8501	-3.09573	-2.93038
H	-12.85715	-3.69402	-4.58895
H	-6.64514	-14.12403	-1.27132
H	-5.60772	-12.67863	-1.14582
H	-7.18912	-12.72288	-0.31124
H	-8.66058	-14.11486	-2.45522
H	-9.19226	-12.68294	-3.38061
H	-9.24538	-12.6497	-1.60189
H	-5.51739	-12.54429	-3.56972
H	-6.48495	-14.05425	-3.60104
H	-7.01346	-12.62378	-4.53049

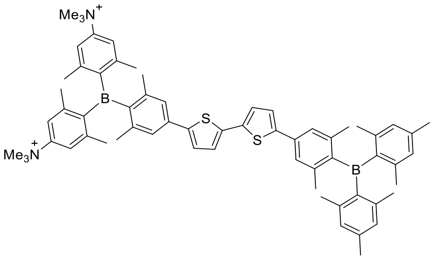

Cat^{2+}

Functional used: TD-DFT CAM-B3LYP 6-31G(d,p), gas phase

Calculated Absorption Spectrum

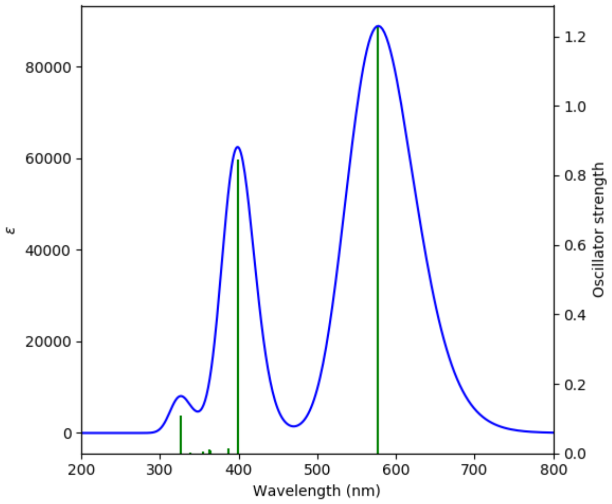

Orbital	Energy [eV]	Symmetry
L+4	-4.87	A
L+3	-5.00	A
L+2	-5.12	A
L+1	-5.40	A
LUMO	-6.34	A
HOMO	-7.66	A
H-1	-7.66	A
H-2	-7.88	A
H-3	-7.92	A
H-4	-7.97	A

Orbitals Relevant for $S_1 \leftarrow S_0$ Transition

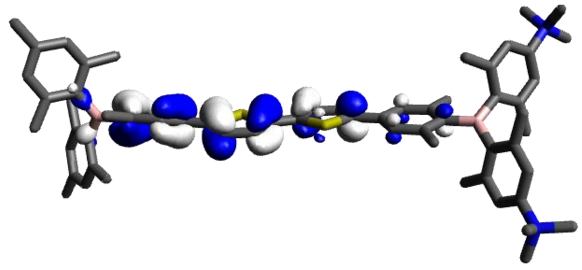

HOMO

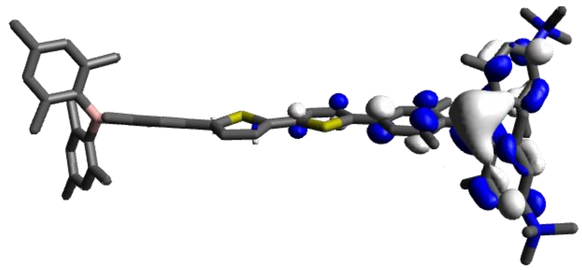

LUMO

Other Relevant Orbitals

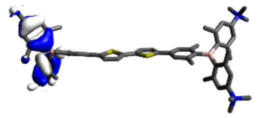

HOMO-1

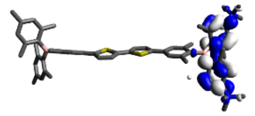

LUMO+1

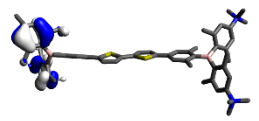

HOMO-2

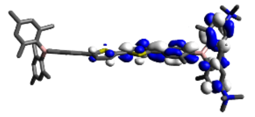

LUMO+2

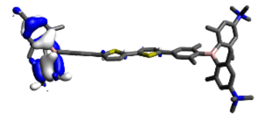

HOMO-3

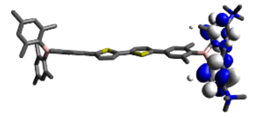

LUMO+3

Table S37: Lowest energy singlet electronic transitions of **Cat^{2+}** (TD-DFT CAM-B3LYP 6-31G(d,p), gas phase).

State	E [eV]	λ [nm]	*f*	Symmetry	Major Contributions	Λ
1	2.15	577	1.227	A	HOMO->LUMO (78%)	0.297
2	3.03	409	5E-4	A	H-1->LUMO (98%)	0.017
3	3.11	399	0.845	A	HOMO->LUMO (13%), HOMO->L+2 (47%), HOMO->L+4 (12%), HOMO->L+5 (12%)	0.411
4	3.20	387	0.016	A	H-6->LUMO (21%), H-3->LUMO (48%)	0.208
5	3.30	375	1E-4	A	H-2->LUMO (90%)	0.044
6	3.40	364	0.007	A	H-6->LUMO (18%), H-4->LUMO (51%), H-3->LUMO (18%)	0.111
7	3.41	363	0.010	A	H-6->LUMO (21%), H-4->LUMO (48%), H-3->LUMO (18%)	0.121
8	3.49	355	0.005	A	HOMO->L+1 (88%)	0.081
9	3.67	338	9E-4	A	H-5->LUMO (90%)	0.064
10	3.80	326	0.109	A	H-1->L+2 (10%), H-1->L+5 (18%), H-1->L+9 (50%)	0.301

Cat^{2+}

TD-DFT CAM-B3LYP 6-31G(d,p), gas phase, S_0

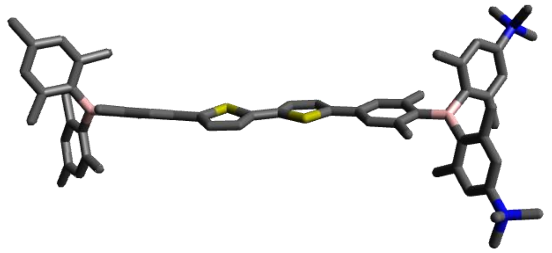

Point group: C_1

Total energy: -2157602.57 kcal/mol

Dipole moment: 85.40 D

Imaginary frequencies: 0

Optimized x, y, z coordinates

C	1.98343	-0.3578	0.56799
C	1.23503	-1.05531	1.50745
C	-0.15307	-0.92284	1.35165
C	-0.51904	-0.11475	0.27948
S	0.90691	0.48161	-0.54563
C	3.41587	-0.2726	0.44423
C	-1.83304	0.2323	-0.16698
S	-3.25705	-0.4671	0.58546
C	-4.33025	0.43364	-0.4635
C	-3.59892	1.21069	-1.34784
C	-2.20503	1.09981	-1.1824
C	-5.77713	0.31053	-0.34824

C	-6.38459	-0.13345	0.83501	C	9.95588	6.27601	0.04394
C	-7.76938	-0.23292	0.95085	C	11.8528	5.08162	-0.9118
C	-8.59939	0.09384	-0.15061	C	9.90527	5.67437	-2.31748
C	-7.98859	0.52763	-1.35439	H	1.68164	-1.6389	2.30294
C	-6.60243	0.64047	-1.43409	H	-0.88021	-1.39151	2.0033
C	4.03476	0.57324	-0.49904	H	-4.06688	1.86623	-2.07079
C	5.41199	0.6795	-0.6131	H	-1.48558	1.65283	-1.77582
C	6.27593	-0.11363	0.20974	H	-5.76762	-0.37507	1.69631
C	5.6478	-0.99111	1.15474	H	-6.15356	0.95932	-2.36953
C	4.26823	-1.04011	1.26683	H	3.41286	1.16624	-1.16236
B	7.80366	-0.02904	0.08384	H	3.83212	-1.6946	2.0131
B	-10.17185	-0.02035	-0.0385	H	8.6241	4.51442	1.11192
C	-10.96449	-0.8263	-1.12185	H	10.66924	2.91546	-2.33671
C	-10.90905	0.67614	1.15457	H	11.34043	-2.60278	1.97512
C	8.70747	-1.3302	0.21989	H	8.95047	-4.46731	-1.09781
C	8.522	1.36352	-0.18578	H	-13.6743	-0.53091	-3.18371
C	8.25238	2.46077	0.66521	H	-10.936	-3.79696	-2.81348
C	8.85918	3.70449	0.43536	H	-13.23118	0.0205	3.57526
C	9.71416	3.87071	-0.64419	H	-11.06366	3.63789	2.85514
C	10.00329	2.80234	-1.48849	H	-11.87184	1.85567	-1.30377
C	9.42551	1.55134	-1.2592	H	-13.39574	1.42924	-2.08646
C	9.78844	-1.40584	1.12476	H	-13.13983	1.08649	-0.3705
C	10.53888	-2.58391	1.24973	H	-13.89215	3.03795	4.14292
C	10.23461	-3.68163	0.45705	H	-13.23311	1.84848	5.26585
C	9.19558	-3.62063	-0.46629	H	-12.35598	3.35677	4.94969
C	8.42314	-2.4624	-0.58625	H	-13.99344	-3.59286	-3.68736
C	-12.12405	-0.27116	-1.72384	H	-13.63129	-2.35768	-4.8925
C	-12.80232	-0.98174	-2.71601	H	-12.54418	-3.74265	-4.68273
C	-12.39859	-2.25601	-3.11999	H	-8.57476	2.53855	1.00009
C	-11.26513	-2.80396	-2.51645	H	-9.72514	2.7958	-0.30197
C	-10.53928	-2.11111	-1.54574	H	-9.69322	3.90557	1.06801
C	-11.88135	-0.03156	1.90859	H	-11.39333	-2.10503	1.4329
C	-12.50662	0.58562	2.9939	H	-12.83442	-1.89154	2.4303
C	-12.23485	1.90817	3.3496	H	-12.87771	-1.53189	0.69932
C	-11.28707	2.60521	2.59766	H	-8.39142	-2.33593	-1.31734
C	-10.61473	2.01265	1.52728	H	-9.29789	-2.74145	0.13136
C	-12.6577	1.09672	-1.35209	H	-9.30204	-3.84889	-1.24094
C	-12.9626	2.57099	4.49072	H	10.37719	0.78458	-3.04353
C	-13.17883	-3.02585	-4.15423	H	10.3071	-0.3706	-1.7051
C	-9.59948	2.85452	0.78296	H	8.85973	-0.01746	-2.63855
C	-12.26354	-1.46469	1.60175	H	7.40693	1.43461	2.38697
C	-9.3197	-2.79242	-0.96101	H	7.44657	3.19685	2.52824
C	9.76285	0.42975	-2.21301	H	6.25746	2.39814	1.48836
C	7.29545	2.36798	1.83237	H	10.53015	0.60044	1.43714
C	10.15374	-0.2445	2.01973	H	9.28733	0.11528	2.58203
C	7.28245	-2.48604	-1.57789	H	10.9218	-0.52617	2.74347
C	5.92796	1.61961	-1.68436	H	6.32287	-2.59959	-1.06558
C	6.43381	-1.85865	2.11771	H	7.21476	-1.56041	-2.15268
C	-8.80111	0.88265	-2.58047	H	7.39307	-3.31507	-2.28106
C	-8.34001	-0.69637	2.27319	H	5.11947	1.89287	-2.36574
N	11.00435	-4.97557	0.57454	H	6.32605	2.54588	-1.25923
C	12.09962	-4.91186	1.60668	H	6.72595	1.17354	-2.28324
C	10.05962	-6.0881	0.97784	H	7.26065	-1.31885	2.58642
C	11.64553	-5.31062	-0.75436	H	5.78258	-2.21952	2.91668
N	10.346	5.20882	-0.94599	H	6.86357	-2.73525	1.62362

H	-9.63471	1.54943	-2.34335
H	-8.17657	1.38267	-3.32533
H	-9.23628	-0.00838	-3.04063
H	-7.55018	-1.0952	2.91513
H	-8.83095	0.12402	2.80335
H	-9.0925	-1.47941	2.14556
H	12.59839	-5.87974	1.62376
H	12.8089	-4.13447	1.32928
H	11.66232	-4.7038	2.58123
H	10.63068	-7.01166	1.07489
H	9.29216	-6.20059	0.21645
H	9.60211	-5.81633	1.92794
H	12.31103	-4.49255	-1.026
H	12.2035	-6.241	-0.64713
H	10.87126	-5.42471	-1.50854
H	10.45347	7.19933	-0.24888
H	8.87664	6.41277	0.01741
H	10.28156	5.97897	1.03894
H	12.29002	6.05778	-1.12248
H	12.1695	4.3613	-1.6616
H	12.14184	4.73748	0.08015
H	8.81853	5.74399	-2.31854
H	10.35412	6.64787	-2.51587
H	10.23041	4.95301	-3.0627

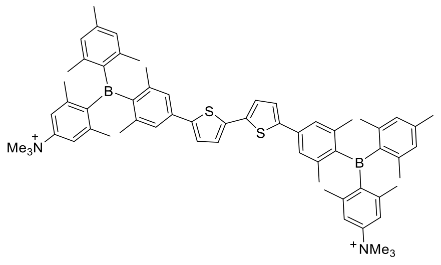

Cat(i)$^{2+}$

Functional used: TD-DFT CAM-B3LYP 6-31G(d,p), gas phase

Calculated Absorption Spectrum

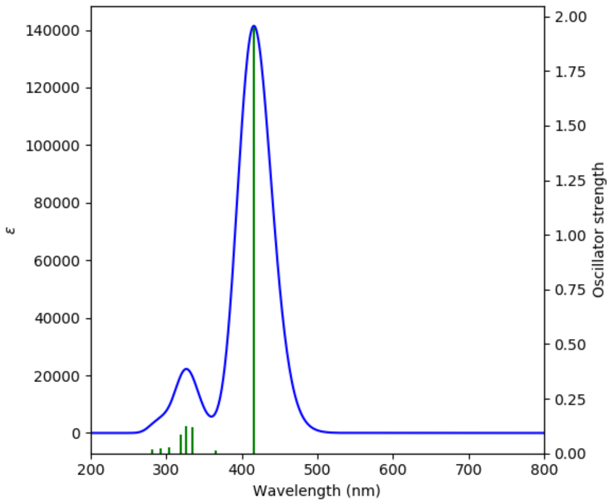

Orbital	Energy [eV]	Symmetry
L+4	-3.99	A
L+3	-4.05	B
L+2	-4.41	A
L+1	-5.04	B
LUMO	-5.14	A
HOMO	-7.72	B
H-1	-8.79	A
H-2	-8.93	B
H-3	-9.01	A
H-4	-9.04	B

Orbitals Relevant for $S_1 \leftarrow S_0$ Transition

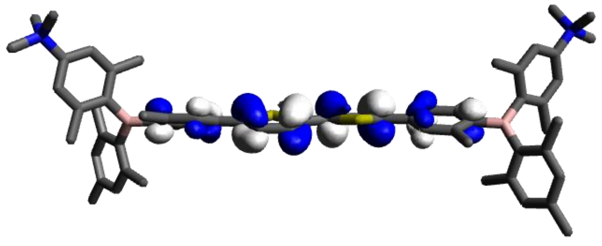

HOMO

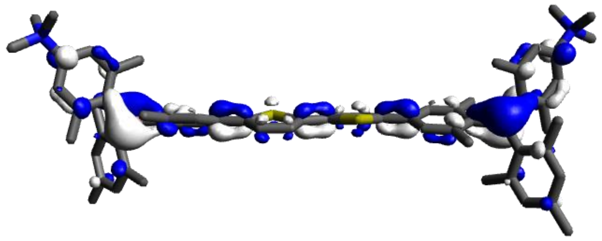

LUMO

Other Relevant Orbitals

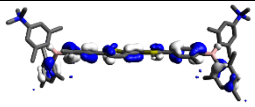

HOMO-1

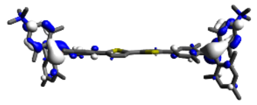

LUMO+1

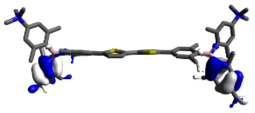

HOMO-2

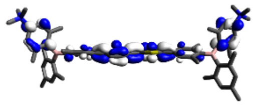

LUMO+2

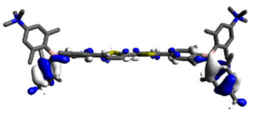

HOMO-3

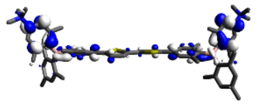

LUMO+3

Table S38: Lowest energy singlet electronic transitions of **Cat(i)$^{2+}$** (TD-DFT CAM-B3LYP 6-31G(d,p), gas phase).

State	E [eV]	λ [nm]	*f*	Symmetry	Major Contributions	Λ
1	2.98	416	1.952	B	HOMO->LUMO (67%), HOMO->L+2 (17%)	0.530
2	3.39	366	0.011	A	H-1->LUMO (24%), HOMO->L+1 (57%)	0.423
3	3.71	335	0.121	B	H-3->L+1 (12%), H-2->LUMO (30%), H-1->L+1 (21%), HOMO->L+2 (22%)	0.516
4	3.80	326	0.126	A	H-3->LUMO (33%), H-2->L+1 (36%), HOMO->L+1 (11%)	0.489
5	3.89	319	0.084	B	H-3->L+1 (22%), H-2->LUMO (13%), HOMO->L+2 (33%)	0.564
6	4.08	304	0.002	A	H-5->L+1 (41%), H-4->LUMO (43%)	0.328
7	4.08	304	0.027	B	H-5->LUMO (42%), H-4->L+1 (40%)	0.326
8	4.23	293	0.014	A	H-7->L+1 (33%), H-6->LUMO (42%)	0.418
9	4.23	293	0.022	B	H-7->LUMO (46%), H-6->L+1 (31%)	0.405
10	4.39	282	0.020	A	H-10->L+1 (15%), H-3->LUMO (12%), H-1->LUMO (28%), HOMO->L+1 (21%)	0.517

Cat(i)$^{2+}$

TD-DFT CAM-B3LYP 6-31G(d,p), gas phase, S_0

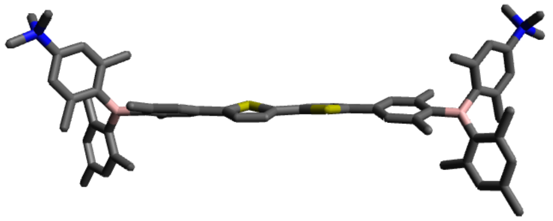

Point group: C_2

Total energy: -2157605.56 kcal/mol

Dipole moment: 25.77 D

Imaginary frequencies: 0

Optimized x, y, z coordinates

C	1.3291	2.8835	2.18084
C	0.97884	2.76766	3.5113
C	0.3817	1.52911	3.84089
C	0.27178	0.66876	2.76541
S	0.92976	1.40771	1.32093
H	1.10941	3.57189	4.22482
H	0.00804	1.28174	4.82683
C	-0.27178	-0.66876	2.76541
C	-0.3817	-1.52911	3.84089
S	-0.92976	-1.40771	1.32093
C	-0.97884	-2.76766	3.5113
H	-0.00804	-1.28174	4.82683
C	-1.3291	-2.8835	2.18084
H	-1.10941	-3.57189	4.22482
C	1.94026	4.01937	1.50077
C	1.87829	4.16791	0.10771
C	2.61426	5.00974	2.23317
C	2.43548	5.26611	-0.54379
H	1.35751	3.42051	-0.48372
C	3.20248	6.10667	1.61119
H	2.69597	4.91021	3.31057
C	3.11696	6.26678	0.2016
C	2.2902	5.32601	-2.05014
C	3.90012	7.11803	2.49685
B	3.75604	7.48877	-0.5302
H	1.97064	4.35643	-2.43945
H	1.54934	6.07123	-2.35243
H	3.22327	5.59522	-2.55181
H	3.60164	8.14488	2.26743
H	3.66197	6.9358	3.54738
H	4.98811	7.06879	2.3897
C	5.23457	7.94022	-0.15402
C	2.98676	8.29172	-1.62449
C	5.51005	9.27953	0.19715
C	6.30251	7.01164	-0.18118
C	3.59768	8.65741	-2.85249
C	1.64707	8.70583	-1.3919
C	6.81677	9.67669	0.51555
C	4.4293	10.33535	0.24647
C	7.60283	7.42805	0.11422
C	6.0877	5.55713	-0.5303
C	2.88205	9.38915	-3.80059
C	5.01111	8.24508	-3.20877
C	0.97884	9.46738	-2.35202
C	0.89725	8.37575	-0.11766
C	7.84995	8.75256	0.46233
H	6.98093	10.71015	0.78784
H	3.5356	9.98181	0.76581
H	4.78164	11.2334	0.75996
H	4.11306	10.61661	-0.76148
H	8.40102	6.69517	0.06946
H	5.54741	5.03995	0.26689
H	5.49063	5.44003	-1.43811
H	7.04054	5.04584	-0.68817
C	1.57267	9.81643	-3.5671
H	3.35565	9.63276	-4.74832
H	5.20932	7.19362	-2.98179
H	5.19201	8.38725	-4.27685
H	5.75572	8.83655	-2.66619
H	-0.03865	9.79259	-2.15045
H	0.58417	7.32793	-0.09406
H	1.4975	8.54051	0.78132
H	0.0004	8.99422	-0.0349
C	0.83272	10.64721	-4.58237
C	9.7788	8.33631	1.9474
C	9.40757	10.60403	1.1396
H	1.18042	10.44111	-5.59807
H	-0.24391	10.46121	-4.54225
H	0.98502	11.71713	-4.39537
H	10.80196	8.64095	2.16913
H	9.12663	8.52754	2.79829
H	9.74917	7.28078	1.69016
H	10.45822	10.80051	1.34851
H	9.07489	11.21414	0.30222
H	8.8081	10.8089	2.02438
C	-1.94026	-4.01937	1.50077
C	-1.87829	-4.16791	0.10771
C	-2.61426	-5.00974	2.23317
C	-2.43548	-5.26611	-0.54379
H	-1.35751	-3.42051	-0.48372
C	-3.20248	-6.10667	1.61119
H	-2.69597	-4.91021	3.31057
C	-3.11696	-6.26678	0.2016
C	-2.2902	-5.32601	-2.05014
C	-3.90012	-7.11803	2.49685
B	-3.75604	-7.48877	-0.5302
H	-1.97064	-4.35643	-2.43945
H	-1.54934	-6.07123	-2.35243
H	-3.22327	-5.59522	-2.55181
H	-3.60164	-8.14488	2.26743
H	-3.66197	-6.9358	3.54738
H	-4.98811	-7.06879	2.3897
C	-5.23457	-7.94022	-0.15402
C	-2.98676	-8.29172	-1.62449
C	-5.51005	-9.27953	0.19715
C	-6.30251	-7.01164	-0.18118
C	-3.59768	-8.65741	-2.85249
C	-1.64707	-8.70583	-1.3919
C	-6.81677	-9.67669	0.51555
C	-4.4293	-10.33535	0.24647
C	-7.60283	-7.42805	0.11422

C	-6.0877	-5.55713	-0.5303
C	-2.88205	-9.38915	-3.80059
C	-5.01111	-8.24508	-3.20877
C	-0.97884	-9.46738	-2.35202
C	-0.89725	-8.37575	-0.11766
C	-7.84995	-8.75256	0.46233
H	-6.98093	-10.71015	0.78784
H	-3.5356	-9.98181	0.76581
H	-4.78164	-11.2334	0.75996
H	-4.11306	-10.61661	-0.76148
H	-8.40102	-6.69517	0.06946
H	-5.54741	-5.03995	0.26689
H	-5.49063	-5.44003	-1.43811
H	-7.04054	-5.04584	-0.68817
C	-1.57267	-9.81643	-3.5671
H	-3.35565	-9.63276	-4.74832
H	-5.20932	-7.19362	-2.98179
H	-5.19201	-8.38725	-4.27685
H	-5.75572	-8.83655	-2.66619
H	0.03865	-9.79259	-2.15045
H	-0.58417	-7.32793	-0.09406
H	-1.4975	-8.54051	0.78132
H	-0.0004	-8.99422	-0.0349
C	-0.83272	-10.64721	-4.58237
C	-9.7788	-8.33631	1.9474
C	-9.40757	-10.60403	1.1396
H	-1.18042	-10.44111	-5.59807
H	0.24391	-10.46121	-4.54225
H	-0.98502-11.71713		-4.39537
H	-10.80196	-8.64095	2.16913
H	-9.12663	-8.52754	2.79829
H	-9.74917	-7.28078	1.69016
H	-10.45822	-10.80051	1.34851
H	-9.07489	-11.21414	0.30222
H	-8.8081	-10.8089	2.02438
C	-10.1457	-8.89688	-0.4332
H	-10.11562	-7.83936	-0.68207
H	-9.75124	-9.4825	-1.26225
H	-11.16639	-9.19953	-0.19773
N	-9.27384	-9.14904	0.7766
C	10.1457	8.89688	-0.4332
H	10.11562	7.83936	-0.68207
H	9.75124	9.4825	-1.26225
H	11.16639	9.19953	-0.19773
N	9.27384	9.14904	0.7766

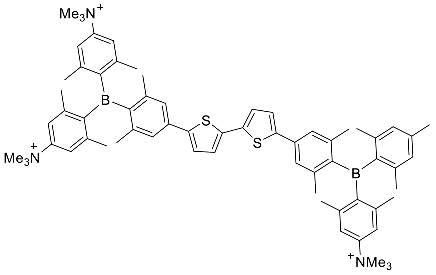

Cat^{3+}

Functional used: TD-DFT CAM-B3LYP 6-31G(d,p), gas phase

Calculated Absorption Spectrum

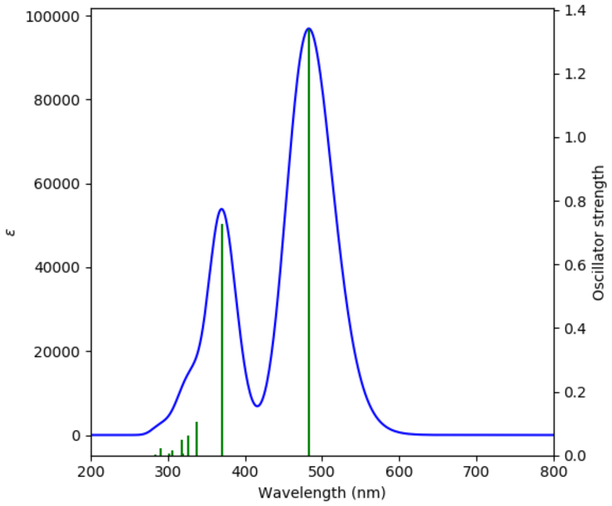

Orbital	Energy [eV]	Symmetry
L+4	-5.89	A
L+3	-5.94	A
L+2	-6.16	A
L+1	-6.28	A
LUMO	-7.31	A
HOMO	-9.14	A
H-1	-9.80	A
H-2	-9.90	A
H-3	-10.28	A
H-4	-10.31	A

Orbitals Relevant for $S_1 \leftarrow S_0$ Transition

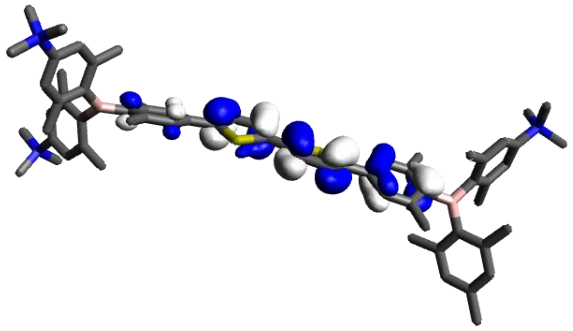

HOMO

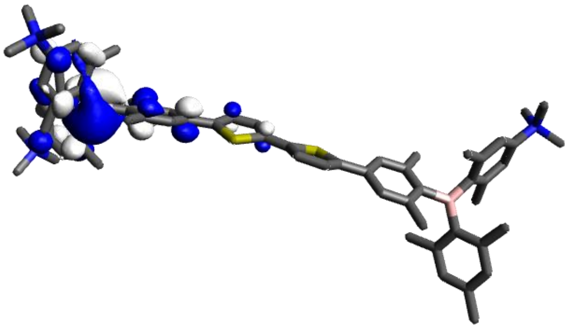

LUMO

Other Relevant Orbitals

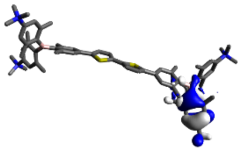

HOMO-1

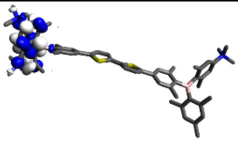

LUMO+1

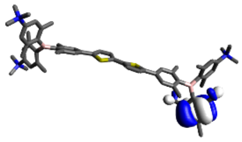

HOMO-2

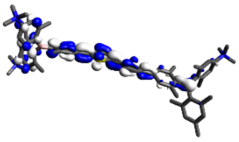

LUMO+2

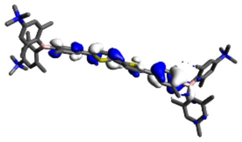

HOMO-3

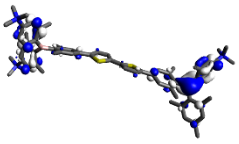

LUMO+3

Table S39: Lowest energy singlet electronic transitions of **Cat^{3+}** (TD-DFT CAM-B3LYP 6-31G(d,p), gas phase).

State	E [eV]	λ [nm]	f	Symmetry	Major Contributions	Λ
1	2.57	483	1.338	A	HOMO->LUMO (74%), HOMO->L+2 (10%)	0.345
2	3.35	371	0.729	A	HOMO->L+2 (57%)	0.547
3	3.67	338	0.107	A	H-1->L+2 (18%), H-1->L+3 (32%), HOMO->L+3 (11%)	0.385
4	3.80	327	0.063	A	H-6->LUMO (12%), H-3->LUMO (41%), HOMO->LUMO (16%)	0.336
5	3.88	319	0.007	A	H-10->LUMO (48%), H-7->LUMO (14%)	0.349
6	3.89	319	0.047	A	H-10->LUMO (12%), H-1->L+3 (12%), HOMO->L+3 (22%)	0.402
7	4.05	306	0.016	A	H-2->L+2 (22%), H-2->L+3 (50%)	0.211
8	4.11	302	0.004	A	HOMO->L+1 (87%)	0.087
9	4.27	291	0.023	A	H-4->L+2 (33%), H-4->L+3 (36%)	0.265
10	4.33	284	0.004	A	H-1->LUMO (82%)	0.121

Cat^{3+}

TD-DFT CAM-B3LYP 6-31G(d,p), gas phase, S_0

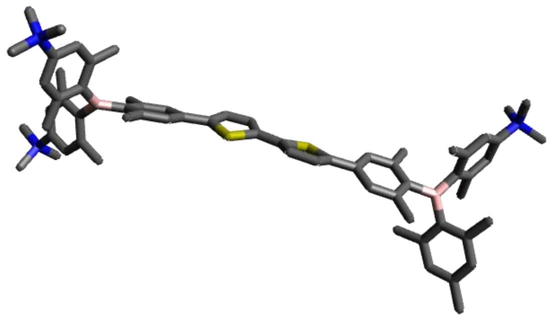

Point group: C_1

Total energy: -2241826.32 kcal/mol

Dipole moment: 40.27 D

Imaginary frequencies: 0

Optimized x, y, z coordinates

C	-3.66446	0.45801	-0.66012
C	-2.92193	1.08263	-1.64318
C	-1.53109	1.07764	-1.39525
C	-1.1826	0.44466	-0.21636
S	-2.61324	-0.16385	0.59554
C	-5.11104	0.26399	-0.60439
C	0.12794	0.23935	0.34125
S	1.56535	0.50626	-0.62281
C	2.62022	0.05066	0.70773
C	1.86307	-0.2965	1.81368

C	0.47223	-0.19051	1.6129
C	4.0632	0.06024	0.57038
C	4.7045	0.69408	-0.50928
C	6.08731	0.7171	-0.64115
C	6.91678	0.05154	0.31317
C	6.26601	-0.60623	1.40377
C	4.88325	-0.57764	1.52076
C	-5.78722	0.0715	0.60855
C	-7.16669	-0.1234	0.65166
C	-7.92151	-0.1627	-0.54854
C	-7.24049	0.03796	-1.77931
C	-5.86475	0.25726	-1.78722
B	-9.46354	-0.44491	-0.54748
B	8.45289	0.03213	0.16552
C	9.28461	-1.28288	0.48847
C	9.24501	1.32295	-0.31478
C	-10.0915	-1.51089	-1.49484
C	-10.41998	0.36133	0.43365
C	-10.41753	1.77271	0.45314
C	-11.32172	2.47543	1.26269
C	-12.20611	1.7779	2.07303
C	-12.20248	0.38676	2.09599
C	-11.32585	-0.328	1.27596
C	-11.28177	-1.25028	-2.22634
C	-11.82547	-2.23415	-3.05167
C	-11.25185	-3.50339	-3.16464
C	-10.09277	-3.76311	-2.43012
C	-9.49443	-2.79523	-1.62174
C	10.38094	-1.27569	1.37782
C	11.08063	-2.4576	1.66152
C	10.71095	-3.64358	1.0432
C	9.65017	-3.66989	0.14305
C	8.92593	-2.50666	-0.13186
C	10.14978	1.28595	-1.39774
C	10.81956	2.44681	-1.81102
C	10.61295	3.64048	-1.13428
C	9.74465	3.69563	-0.04824
C	9.04986	2.55425	0.36095
C	10.82489	-0.01455	2.08263
N	11.31031	4.91283	-1.55213
N	11.43396	-4.93822	1.32775
C	8.09134	2.69897	1.52115
C	10.41055	0.01481	-2.17261
C	7.76004	-2.62106	-1.0877
C	-11.39542	-1.83713	1.3202
C	-9.46761	2.57991	-0.40171
C	-11.98857	0.08915	-2.18327
C	-8.23305	-3.19166	-0.88207
C	-7.80929	-0.32134	2.00759
C	-7.95233	0.0202	-3.11556
C	7.02633	-1.32287	2.50145
C	6.64202	1.43242	-1.85637
C	-11.84882	-4.54655	-4.07156
N	-13.20421	2.49784	2.94943
C	-13.11177	3.99588	2.82984
C	-14.60727	2.09273	2.55234
C	-12.9612	2.13717	4.39798
C	12.55883	-4.77491	2.31741
C	10.45974	-5.94421	1.90459
C	12.02701	-5.48316	0.0459
C	12.2185	4.71953	-2.73927
C	10.27752	5.95369	-1.93108
C	12.15588	5.42973	-0.40775
H	-3.3737	1.559	-2.50414
H	-0.8034	1.54322	-2.04987
H	2.30012	-0.59222	2.75942
H	-0.26432	-0.40244	2.37843
H	4.10252	1.19439	-1.26157
H	4.42227	-1.07952	2.36435
H	-5.22904	0.09344	1.54017
H	-5.36009	0.38644	-2.73917
H	-11.30045	3.55591	1.22943
H	-12.88178	-0.17112	2.73107
H	-12.72218	-2.00842	-3.62321
H	-9.63971	-4.74905	-2.49308
H	11.89908	-2.40793	2.36614
H	9.35275	-4.58739	-0.35211
H	11.48634	2.37519	-2.65906
H	9.57635	4.61945	0.49371
H	9.98826	0.48735	2.57654
H	11.57452	-0.23028	2.84699
H	11.25719	0.70036	1.37743
H	7.05484	2.70102	1.17236
H	8.17513	1.87588	2.23385
H	8.27003	3.63129	2.06183
H	9.4793	-0.4611	-2.49195
H	11.00231	0.21108	-3.06929
H	10.95143	-0.7145	-1.56356
H	6.80939	-2.60093	-0.54736
H	7.72606	-1.79587	-1.80194
H	7.80927	-3.55408	-1.65389
H	-11.95205	-2.17755	2.19683
H	-11.88743	-2.23045	0.42665
H	-10.40336	-2.29259	1.35535
H	-9.49676	2.26601	-1.44856
H	-9.71343	3.64392	-0.36753
H	-8.4353	2.45456	-0.06526
H	-12.5554	0.22071	-1.25584
H	-11.29518	0.93143	-2.25781
H	-12.69321	0.17328	-3.01383
H	-7.34464	-2.73065	-1.32336
H	-8.24849	-2.89651	0.17064
H	-8.09881	-4.27524	-0.91838
H	-7.04848	-0.41641	2.78571
H	-8.4563	0.51981	2.27331
H	-8.42678	-1.22401	2.04281
H	-8.88881	0.58363	-3.09967
H	-7.31729	0.45319	-3.8921
H	-8.208	-0.99905	-3.41772
H	7.88554	-0.74971	2.85893

H	6.37336	-1.50194	3.35826
H	7.4049	-2.29386	2.16823
H	5.84275	1.63693	-2.57173
H	7.09924	2.39028	-1.59094
H	7.40469	0.84683	-2.37604
H	-12.93478	-4.44154	-4.14228
H	-11.4463	-4.45216	-5.08727
H	-11.62142	-5.55734	-3.72336
H	-13.86227	4.43009	3.48879
H	-12.11838	4.31904	3.13477
H	-13.3101	4.28578	1.79987
H	-15.31686	2.63073	3.18139
H	-14.72311	1.02073	2.68882
H	-14.74969	2.3495	1.50371
H	-11.94332	2.42809	4.65329
H	-13.68206	2.67226	5.01663
H	-13.0819	1.06463	4.52569
H	13.02027	-5.75074	2.46099
H	13.29037	-4.07555	1.91723
H	12.15655	-4.41406	3.26204
H	10.99945	-6.8667	2.11913
H	9.66902	-6.13305	1.1831
H	10.03839	-5.52411	2.81667
H	12.71498	-4.73938	-0.35336
H	12.5532	-6.4104	0.27304
H	11.2293	-5.67235	-0.66773
H	12.67535	5.68094	-2.96892
H	12.99	3.99471	-2.48671
H	11.63088	4.37737	-3.58896
H	10.79632	6.85961	-2.24488
H	9.64649	6.16326	-1.07125
H	9.6754	5.55357	-2.7455
H	12.88247	4.66129	-0.14798
H	12.65938	6.34076	-0.73145
H	11.51643	5.64011	0.44563

References

[1] R. Uson, L. A. Oro, J. A. Cabeza, H. E. B. Bryndza, M. P. Stepro, *Inorg. Synth.* **1985**, *23*, 126-130.

[2] S. S. Zalesskiy, V. P. Ananikov, *Organometallics* **2012**, *31*, 2302-2309.

[3] S. Colella, M. Mazzeo, R. Grisorio, E. Fabiano, G. Melcarne, S. Carallo, M. D. Angione, L. Torsi, G. P. Suranna, F. della Sala, P. Mastrorilli, G. Gigli, *Chem. Commun*. **2010**, *46*, 6273-6275.

[4] S. Griesbeck, Z. Zhang, M. Gutmann, T. Lühmann, R. M. Edkins, G. Clermont, A. N. Lazar, M. Haehnel, K. Edkins, A. Eichhorn, M. Blanchard-Desce, L. Meinel, T. B. Marder, *Chem. Eur. J*. **2016**, *22*, 14701-14706.

[5] M. Ferger, S. M. Berger, F. Rauch, M. Schönitz, J. Rühe, J. Krebs, A. Friedrich, T. B. Marder, *Chem. Eur. J.* **2021**, *27*, 9094-9101.

[6] J. C. Collings, S.-Y. Poon, C. Le Droumaguet, M. Charlot, C. Katan, L.-O. Pålsson, A. Beeby, J. A. Mosely, H. M. Kaiser, D. Kaufmann, W.-Y. Wong, M. Blanchard-Desce, T. B. Marder, *Chem. Eur. J*. **2009**, *15*, 198-208.

[7a] G. M. Sheldrick, *Acta Crystallogr*. **2015**, *A71*, 3-8.

[7b] G. M. Sheldrick, *Acta Crystallogr*. **2015**, *C71*, 3–8.

[8] C. B. Hübschle, G. M. Sheldrick, B. Dittrich, *J. Appl. Crystallogr*. **2011**, *44*, 1281-1284.

[9] Brandenburg, K. Diamond (version 4.4.0), Crystal and Molecular Structure Visualization, Crystal Impact H. Putz & K. Brandenburg GbR, Bonn (Germany), **2017**.

[10] R. Schmidt, C. Tanielian, R. Dunsbach, C. Wolff, *J. Photochem. Photobio. A Chem.* **1994**, *79*, 11-17.

[11] M. J. Frisch, G. W. Trucks, H. B. Schlegel, G. E. Scuseria, M. A. Robb, J. R. Cheeseman, G. Scalmani, V. Barone, B. Mennucci, G. A. Petersson, H. Nakatsuji, M. Caricato, X. Li, H. P. Hratchian, A. F. Izmaylov, J. Bloino, G. Zheng, J. L. Sonnenberg, M. Hada, M. Ehara, K. Toyota, R. Fukuda, J. Hasegawa, M. Ishida, T. Nakajima, Y. Honda, O. Kitao, H. Nakai, T. Vreven, J. A. Montgomery Jr, J. E. Peralta, F. Ogliaro, M. Bearpark, J. J. Heyd, E. Brothers, K. N. Kudin, V. N. Staroverov, R. Kobayashi, J. Normand, K. Raghavachari, A. Rendell, J. C. Burant, S. S. Iyengar, J. Tomasi, M. Cossi, N. Rega, J. M. Millam, M. Klene, J. E. Knox, J. B. Cross, V. Bakken, C. Adamo, J. Jaramillo, R. Gomperts, R. E. Stratmann, O. Yazyev, A. J. Austin, R. Cammi, C. Pomelli, J. W. Ochterski, R. L. Martin, K. Morokuma, V. G. Zakrzewski, G. A. Voth, P. Salvador, J. J. Dannenberg, S. Dapprich, A. D. Daniels, O. Farkas, J. B. Foresman, J. V. Ortiz, J. Cioslowski, D. J. Fox, *Gaussian 09, Revision E.01*, Gaussian, Inc., Wallingford CT, **2009**.

[12] T. Lu, F. Chen, *J. Comput. Chem.* **2012**, *33*, 580-592.
[13] A. D. Becke, *J. Chem. Phys.* **1993**, *98*, 5648-5652.
[14] C. Lee, W. Yang, R. G. Parr, *Phys. Rev. B Condens. Matter.* **1988**, *37*, 785-789.
[15] P. J. Stephens, F. J. Devlin, C. F. Chabalowski, M. J. Frisch, *J. Phys. Chem. A* **1994**, *98*, 11623-11627.
[16] G. A. Petersson, A. Bennett, T. G. Tensfeldt, M. A. Al-Laham, W. A. Shirley, *J. Chem. Phys.* **1988**, *89*, 2193-2218.
[17] G. A. Petersson, M. A. Al-Laham, *J. Chem. Phys.* **1991**, *94*, 6081-6090.
[18] T. Yanai, D. P. Tew, N. C. Handy, *Chem. Phys. Lett.* **2004**, *393*, 51-57.
[19] J. B. Chaires, N. Dattagupta, D. M. Crothers, *Biochemistry* **1982**, *21*, 3933-3940.
[20] L.-M. Tumir, I. Piantanida, I. J. Cindrić, T. Hrenar, Z. Meić, M. Žinić, *J. Phys. Org. Chem*. **2003**, *16*, 891-899.
[21] J.-L. Mergny, L. Lacroix, *Oligonucleotides* **2003**, *13*, 515-537.
[22] G. Scatchard, *Ann. N.Y. Acad. Sci*. **1949**, *51*, 660-672.
[23] J. D. McGhee, P. H. von Hippel, *J. Mol. Biol*. **1974**, *86*, 469-489.
[24] T. Mosmann, *J. Immunol. Methods* **1983**. *65*, 55-63.
[25] C. Chazalette, M. Riviere-Baudet, A. Scozzafava, F. Abbate, Z. B. Maarouf, C. T. Supuran, *J. Enzym. Inhib. Med. Chem.* **2001**, *16*, 125-133.
[26] N. Suzuki, K. Suda, D. Yokogawa, H. Kitoh-Nishioka, S. Irle, A. Ando, L. M. G. Abegao, K. Kamada, A. Fukazawa, S. Yamaguchi, *Chem. Sci*. **2018**, *9*, 2666-2673.
[27] S. Hotta, T. Katagiri, *J. Heterocyclic Chem*. **2003**, *40*, 845-850.
[28] S. Colella, M. Mazzeo, R. Grisorio, E. Fabiano, G. Melcarne, S. Carallo, M. D. Angione, L. Torsi, G. P. Suranna, F. della Sala, P. Mastrorilli, G. Gigli, *Chem. Commun*. **2010**, *46*, 6273-6275.
[29] S. Ahn, K. Yabumoto, Y. Jeong, K. Akagi, *Polym. Chem*. **2014**, *5*, 6977-6989.
[30] M. J. Peach, P. Benfield, T. Helgaker, D. J. Tozer, *J. Chem. Phys*. **2008**, *128*, 044118.
[31] S. Griesbeck, M. Ferger, C. Czernetzi, C. Wang, R. Bertermann, A. Friedrich, M. Haehnel, D. Sieh, M. Taki, S. Yamaguchi, T. B. Marder, *Chem. Eur. J.* **2019**, *25*, 7679-7688.
[32] D. Reitzenstein, T. Quast, F. Kanal, M. Kullmann, S. Ruetzel, M. S. Hammer, C. Deibel, V. Dyakonov, T. Brixner, C. Lambert, *Chem. Mater.* **2010**, *22*, 6641-6655.
[33] D. Tsiplakides, D. Archonta, C. G. Vayenas, *Top. Catal.* **2007**, *44*, 469-479.
[34] N. G. Connelly, W. E. Geiger, *Chem. Rev.* **1996**, *96*, 877-910.
[35] D. T. Sawyer, J. L. Roberts, *Experimental Electrochemistry for Chemists*, Wiley-Interscience, New York, **1974**.
